# Effect of the µ-Diphosphine Bridging Ligand on the Cytotoxic Activity of Homodinuclear Ruthenium Complexes

**DOI:** 10.3390/molecules31172968

**Published:** 2026-08-25

**Authors:** Basile Roufosse, Kathia L. Jiménez-Monroy, Christoph Marschner, Jill van de Laarschot, Oscar Loftus, Thomas J. Cleij, Sharon Prince, Burgert Blom

**Affiliations:** 1 Maastricht Science Programme, Faculty of Science and Engineering, Maastricht University, Paul-Henri Spaaklaan 1, 6229 EN Maastricht, The Netherlands; k.jimenezmonroy@maastrichtuniversity.nl (K.L.J.-M.); thomas.cleij@maastrichtuniversity.nl (T.J.C.); 2 Institut für Anorganische Chemie, Technische Universität Graz, Stremayrgasse 9, A-8010 Graz, Austria; christoph.marschner@tugraz.at; 3 Department of Human Biology, University of Cape Town, Observatory 7925, South Africa; sharon.prince@uct.ac.za

**Keywords:** diphosphine, bridging ligands, bimetallic, cancer, ruthenium, cytotoxicity

## Abstract

Herein we report the synthesis and full characterisation of Ru(II)-based homobimetallic complexes bridged by diphosphines of the type [{(*p*-cym)RuCl_2_}_2_(µ-dppx)], where *p*-cym = η^6^-1-isopropyl-4-methylbenzene, dppx = dppe (1,2-bis(diphenylphosphino)ethane, **1**), tdppe (*trans*-1,2-bis(diphenylphosphino)ethylene, **2**), dppa (bis(diphenylphosphino)acetylene, **3**), dcpe (1,2-bis(dicyclohexylphosphino)ethane, **4**), and 1,4dppb (1,4-bis(diphenylphosphino)benzene, **5**). We also report the mononuclear complexes [(*p*-cym)RuCl_2_(κ^1^-dppx)], where dppx = dppe (**6**) and tdppe (**7**). Complexes **2**, **4**, **5**, **6** and **7** were further characterised *via* single crystal X-ray diffraction analysis. The bimetallic complexes were synthesised in a straightforward fashion and isolated in good to excellent yields. The mononuclear complexes **6** and **7** are stable in air but exhibit reactions in solution, hindering their use as precursors for the synthesis of bimetallic complexes. The in vitro cytotoxic (anticancer) activities of complexes **1**–**5** were determined via cell viability studies and their IC_50_ values were derived on HeLa and Ect1/E6E7 cell lines. All complexes displayed only low to moderate activity and selectivity. Strikingly, the cytotoxic profiles of the different complexes varied substantially, highlighting the importance of the bridging ligand itself in the cytotoxic behaviour of the complexes, further demonstrating the importance of ligand design in bimetallic complexes.

## 1. Introduction

**Scheme 1:**
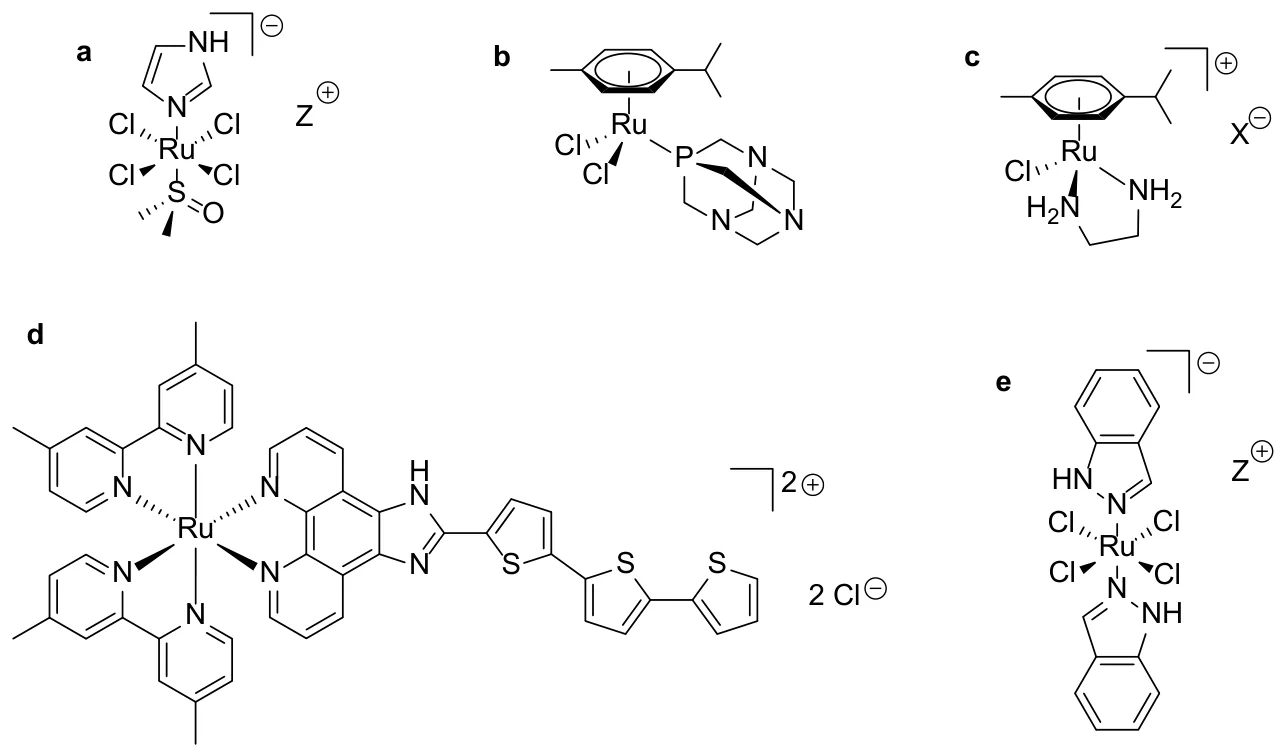
Some Ru-based complexes which reached (pre)clinical trials. (**a**): NAMI (Z = Na^+^) or NAMI-A (Z = imidazoleH^+^), (**b**): RAPTA-C, (**c**): RAED-C, (**d**): TLD1433 and (**e**): KP1339 (Z = Na^+^) or KP1019 (Z = indazoleH^+^).

Since the discovery of the anticancer properties of NAMI by Sava et al. in 1992 (Scheme 1a) [1,2], a number of Ru-based complexes have been investigated for their anticancer properties [3]. A plethora of Ru-based molecular designs have been explored over the years, including photodynamic systems [4,5,6,7,8], photoactivated systems [9,10,11,12], photothermal systems [13,14,15], theranostics [16,17], and nanoparticles [18,19]. Some in-depth reviews highlight the predominance of Ru-based coordination complexes as potential alternatives to the Pt-based drugs currently in use [20,21,22,23,24], and some of those Ru-based complexes have undergone (pre)clinical trials (Scheme 1b–e) [25,26,27,28,29,30,31].

**Scheme 2:**
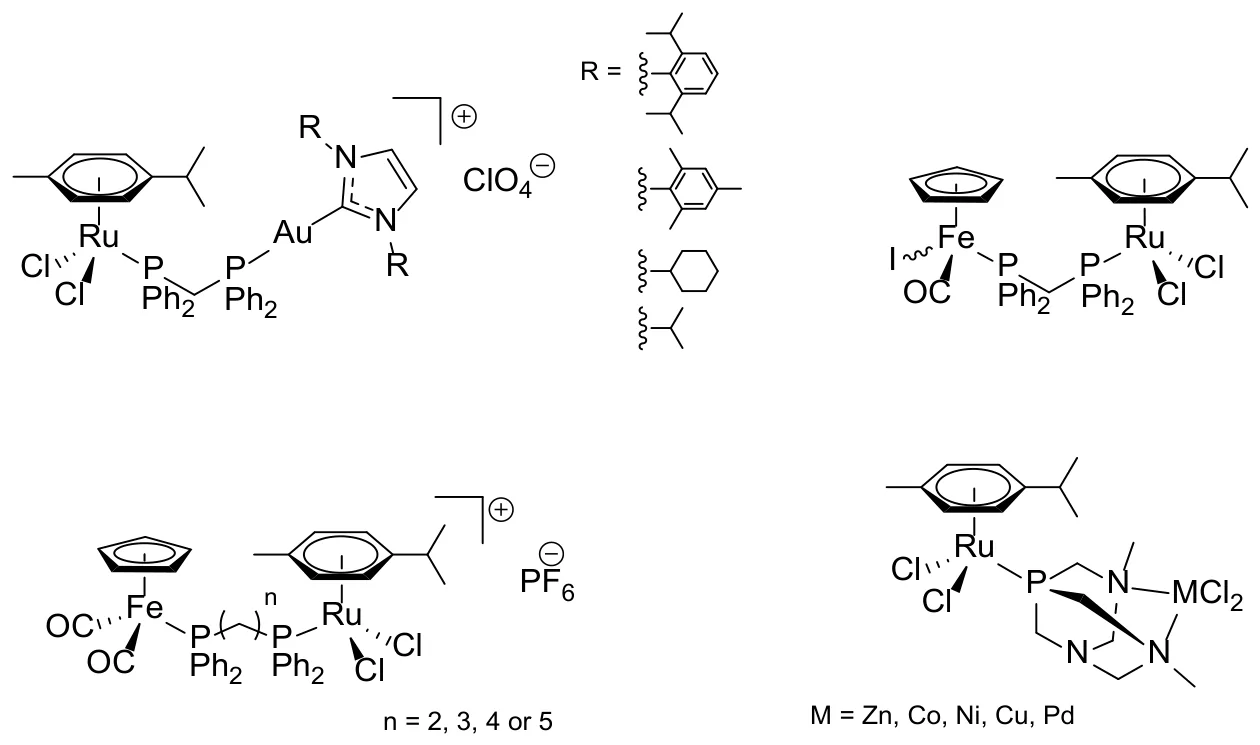
Examples of biologically active bimetallic complexes of the general formula [(*p*-cym)RuCl_2_PR_2_R′M] reported in the literature.

More recently, bimetallic Ru-based candidates have been studied, with the rationale that the presence of two metal centres within a well-defined complex could enhance anticancer activity, possibly by inter alia synergistic effects. Some examples of [(*p*-cym)RuCl_2_PR_2_R′M] bimetallic complexes exhibiting promising anticancer activities include [(*p*-cym)RuCl_2_(µ-dppm)Au(NHC)](ClO_4_) [32], [(η^6^-arene)RuCl_2_(µ-dppm)FeI(η^5^-Cp)(CO)] [33], and [(*p*-cym)RuCl_2_(µ-dppx)Fe(CO)_2_(η^5^-Cp)](PF_6_) [34] (Scheme 2) (dppm = 1,1-bis(diphenylphosphino)methane). Other examples of highly active bimetallic complexes include complexes of the type [(*p*-cym)RuCl_2_-μ-dmoPTA-1κ*P*:2κ^2^*N*,*N′*-MCl_2_] (M = Zn, Co, Ni), where a significant increase in cytotoxic activity was observed upon binding of a second metal to the Ru-based monomeric fragment, surpassing cisplatin in Caco-2/TC7 colon cancer cell line [35]. More recently, the same group reported the Cu and Pd analogue complexes, further showing the versatile tuneability of such systems (Scheme 2). However these two novel examples performed suboptimally, presumably due to their exceptional stability in water and DMSO, which the authors link to the lack of activity [36].

**Scheme 3:**
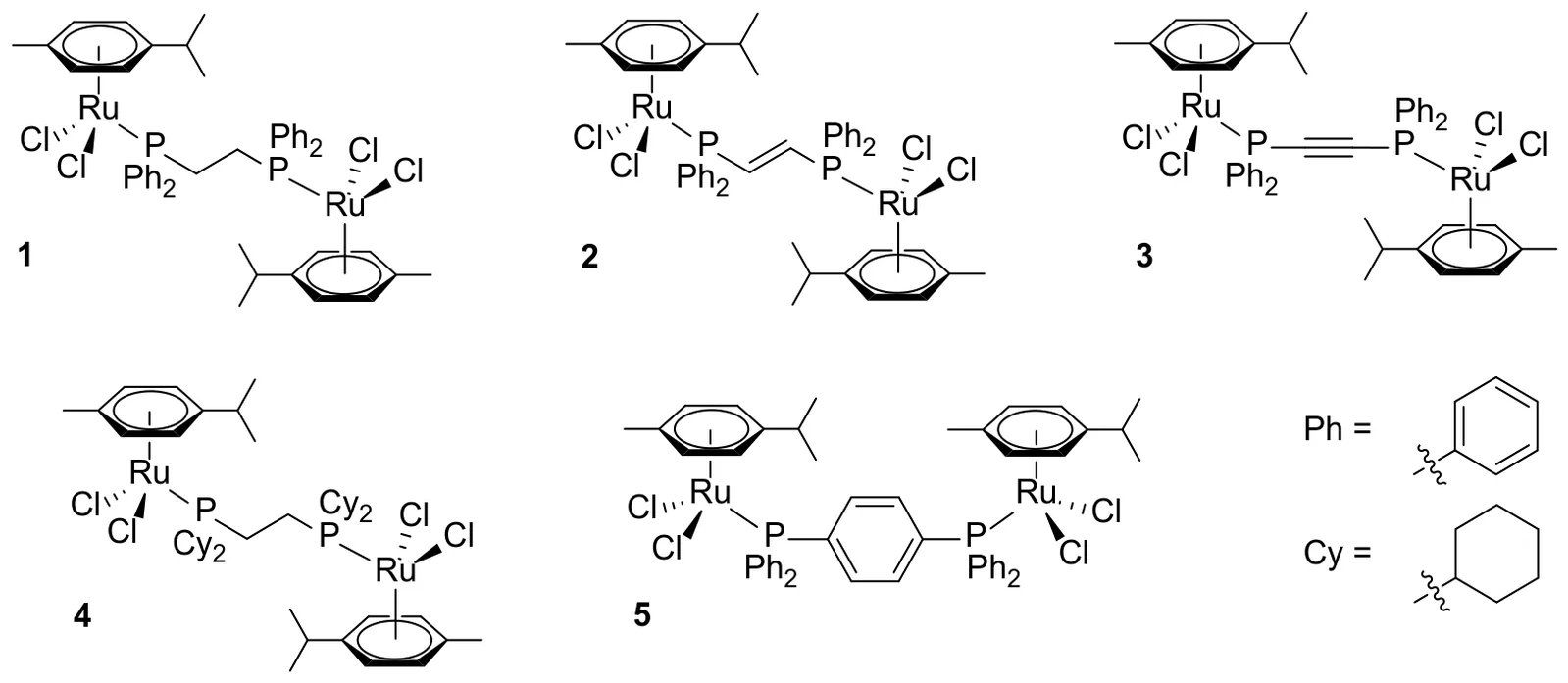
Schematic representation of the bimetallic complexes **1**–**5** studied herein.

A versatile synthetic strategy for such bimetallic molecular designs consists of using a µ-diphosphine bridging ligand, where each P atom can ligate to a different metal centre in k^1^ fashion, enabling facile access to a range of bimetallic complexes. We have recently reviewed all bimetallic complexes containing a group 8 metal (Fe, Ru, Os) and a bridging diphosphine ligand in the context of anticancer research, highlighting the relevance of these systems in the pursuit of the next generation of anticancer agents [37]. Within this scope, we noticed that several structural variations have been carefully studied regarding the ligand spheres around the metal atoms; however, few studies have highlighted the effects of the bridging ligands *themselves* on the biological (anticancer) activity of the complexes. In fact, only two reports studied complexes of the type [{(*p*-cym)RuCl_2_}_2_(µ-dppx)], where dppx is a µ-diphosphine bridging ligand in the context of anticancer studies to date [38,39]. In an attempt to delineate the effects the µ-bridging diphosphine ligand itself could have on cytotoxic profiles of complexes of this type, we hereby present a series of homodinuclear Ru(II)-based complexes of the general formula [{(*p*-cym)RuCl_2_}_2_(µ-dppx)], where the only structural differences lie within the µ-bridging diphosphine ligand (Scheme 3). These serve as model complexes to show the dramatic differences in the in vitro anticancer biological activity, which can solely be attributed to the nature of the ligand present.

**Scheme 4:**
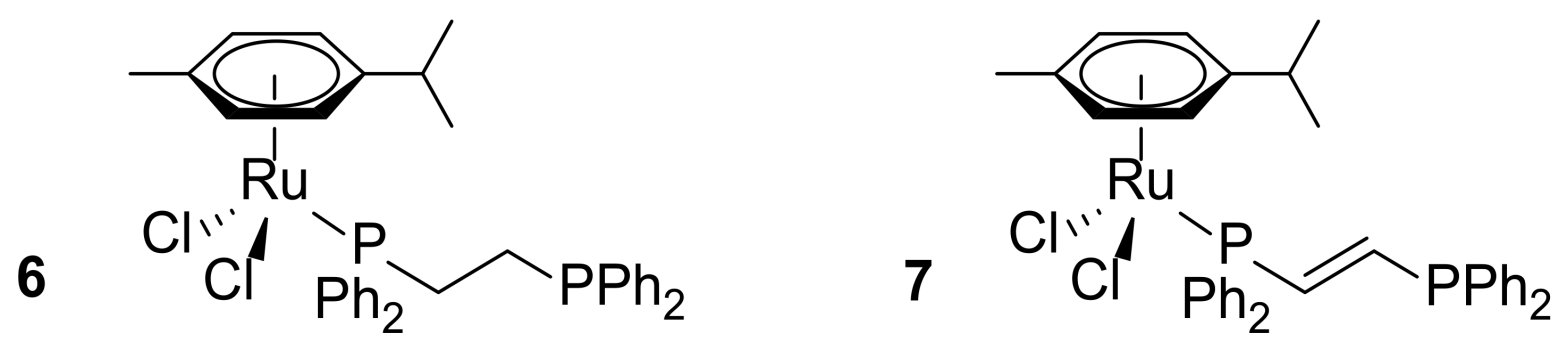
Schematic representation of the monometallic complexes **6** and **7** studied herein.

The synthesis of monometallic precursors has also been investigated, and the complexes [(*p*-cym)RuCl_2_(κ^1^-dppx)] (dppx = dppe (**6**) and tdppe (**7**)) are reported (Scheme 4) herein. These complexes undergo further reactions in solution, and hence their use as precursors towards heterobimetallic systems is impractical. Mechanistic studies of similar complexes have been described by Chaplin et al., where the Z-isomer of complex **7** is described and characterised [40,41]. Nevertheless, complexes **6** and **7** represent novel entities and were fully characterised, and their solid-state crystal structures are reported for the sake of completeness.

All complexes were fully characterised via (multinuclear) NMR, FT-IR, and UV–Vis spectroscopy, as well as high-resolution mass spectrometry (HRMS). The solid-state crystal structures of complexes **2**, **4**, **5**, **6** and **7** were determined via means of single crystal X-ray diffraction analysis. Finally, in vitro anticancer cytotoxic studies were performed through multidose treatment of malignant cell line HeLa, and healthy cell line Ect1/E6E7 with complexes **1**–**5**.

## 2. Results and Discussion

### 2.1. Synthesis and Spectroscopic Characterisation of the Bimetallic Complexes ***1*–*5***

**Scheme 5:**
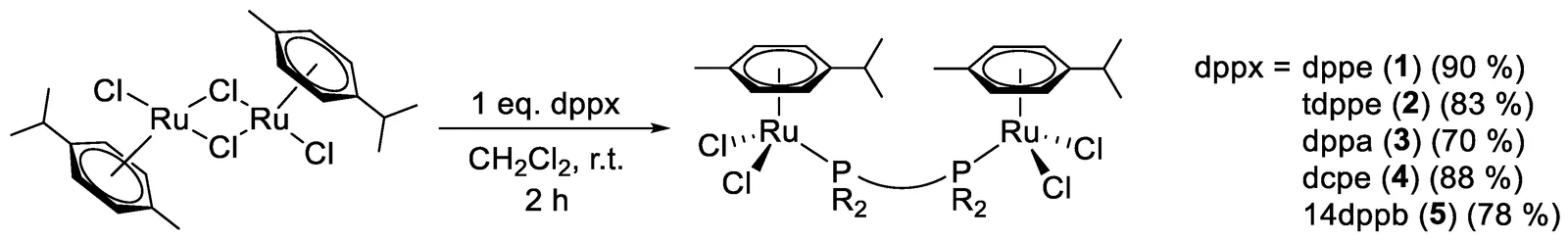
Synthesis of the bimetallic complexes **1**–**5**.

The homobimetallic complexes were synthesised in a straightforward fashion, stirring equimolar amounts of [(*p*-cym)RuCl_2_]_2_ and the respective diphosphine in CH_2_Cl_2_ for 2 h at room temperature. Each P atom binds to a Ru(II) centre, thereby inducing dimer cleavage to form the homodinuclear complexes (Scheme 5). The products were obtained as orange/red powders in good to excellent yields ranging from 70% to 90%.

The FT-IR spectra of all complexes are, as expected, very similar, with the major difference being the much more pronounced C-H stretching vibrations in complex **4** between 2830 and 3040 cm^−1^ (Figures S40–S44). Regarding UV–Vis, all spectra exhibit a strong absorption at around 225 nm, which is assigned to a π-π* LLCT transition. Complexes **1** and **5** exhibit a clearly defined absorption at 255 and 251 nm, respectively, whereas complex **2** exhibits an absorption in the form of a shoulder at 249 nm, which is absent in complexes **3** and **4**. Seeing as the diphosphine bridging ligand is the only structural difference between the complexes, this electronic transition might be assigned to a diphosphine-centred LLCT, or a LMCT transition between the bridging ligand and the Ru centre. The minor absorption maxima, observed in all complexes, presented the most wavelength shift (Table 1). These electronic transitions are tentatively assigned to MLCT transitions, more specifically electronic transitions between the Ru(II) centred molecular orbital (HOMO), and ligand-centred orbitals (LUMO) (Figures S47–S56).

The ^1^H NMR spectra of all complexes, except for **4**, display aromatic multiplets in the region d = 8–7 ppm, corresponding to the aromatic Ph substituents on both P atoms, integrating to 20 H. Complex **4**, in contrast, exhibits cyclohexyl signals in the aliphatic region, integrating to the expected total of 44 H. Complex **1** has the bridging ethylene protons displayed as a doublet signal at d = 2.43 ppm integrating to 4 H, while complex **2** has the olefinic protons displayed as a triplet signal at d = 6.93 ppm due to coupling to both equivalent P atoms. The geminal H atoms do not couple to one another as they are magnetically equivalent. The aromatic protons on the η^6^-*p*-cymene moiety are observed as a pair of doublets in the region from δ = 5.5–5 ppm, integrating to 8 H in all cases, with the roofing effect observed. Finally, the expected septet, singlet and doublet resonance signals arising from the *p*-cymene substituents are observed in the aliphatic region and integrate to the expected values in all cases (Figures S1–S5). The ^31^P{^1^H} NMR spectra further support the bimetallic nature of these complexes, as only one singlet resonance signal is observed in all cases, shifted downfield compared to the respective naked diphosphines due to the deshielding effect of Ru (Figures S15–S19). The ^13^C{^1^H} NMR spectra display the expected signals, with the aromatic signals corresponding to the phenyl rings on P from δ = 140–130 ppm for **2** and **5**, followed by four signals corresponding to the four C environments within the *p*-cymene aromatic ring. In all cases the signals corresponding to the aliphatic substituent on *p*-cymene were assigned unambiguously through two-dimensional ^1^H-^13^C HSQC NMR spectroscopy. Complex **2** has the olefinic C downfield at 140 ppm, while complex **5** has the bridging aromatic C of the 1,4-dppb ligand at 136 and 133 ppm, within the phenyl region (Figures S8–S12). Furthermore, high-resolution mass spectrometry was performed, and all molecular peaks were observed either as the Na adduct [M + Na]^+^ or the [M-Cl]^+^ fragment. The mass spectra and theoretical isotope patterns comparisons can be found in the Supporting Information (Figures S57–S61).

### 2.2. Synthesis and Spectroscopic Characterisation of the Monometallic Complexes ***6*** and ***7***

Our initial starting point to access the dinuclear complexes was to first prepare and isolate the mononuclear complexes [(*p*-cym)RuCl_2_(κ^1^-dppx)] (dppx = dppe (**6**) or tdppe (**7**)) as building blocks for subsequent reactions to prepare the target dinuclear complexes. As it turns out, and as was observed by Chaplin et al. [40,41], complexes bearing κ^1^-diphosphines tend to undergo further reactions in solution, thereby hindering their use as precursors. Nevertheless, we managed to isolate complexes **6** and **7** in pure form, albeit at very low yields, and we decided to include the synthesis and characterisation of these two monometallic complexes herein. Complex **6** was synthesised using a 1:2 ratio [(*p*-cym)RuCl_2_]_2_: dppe in CH_2_Cl_2_ at −78 °C. A low temperature was utilised in order to minimise the dppe ligand to form a more stable bidentate salt complex with Cl as a counteranion, due to the flexibility of the ligand backbone, for which it is prone. After purification via column chromatography on silica gel, a yield of 10% was obtained. Complex **7** was synthesised in a similar fashion; however, room temperature was used due to the more rigid ligand backbone of tdppe, preventing κ^2^-product formation. The yield of this reaction was however even lower with 4% desired product obtained. Similar to the bimetallic complexes discussed above, the ^1^H NMR spectra of complexes **6** and **7** display multiplet signals in the aromatic region integrating to 20 H, attributed to the Ph rings on both P atoms, as well as the pair of doublets from the *p*-cymene aromatic protons integrating to 4 H. In addition, the septet, singlet and doublet signals integrating to 1, 3 and 6 H, respectively, corresponding to the *p*-cymene methyl and isopropyl groups, are observed in the aliphatic region. The main difference stems from the chemically inequivalent H within the bridging ligand. In complex **6**, these are displayed as multiplets, due to each H atom coupling to both inequivalent P atoms, as well as the neighbouring H atoms, each signal integrating to 2 H in the aliphatic region. Complex **7** has the bridging olefinic H signals in the range 6.9–6.3 ppm region, displayed as two multiplets integrating to 1 H each (Figures S6 and S7). Both ^31^P{^1^H} spectra display two sets of doublets with equivalent coupling constants, as expected. The downfield doublet is attributed to the P atom coordinated to Ru, as it has a deshielding effect. The upfield signal corresponds to the pendant phosphine, which matches the typical chemical shift of free phosphines (Figures S20 and S21). The ^13^C{^1^H} NMR spectra resemble closely those of the bimetallic complexes discussed prior. The main difference being that each C atom within the diphosphine has a distinct signal, which is observed as a (pseudo) doublet of doublet, due to coupling to both chemically inequivalent P atoms. These bridging ligand signals in **7** were assigned through ^1^H-^13^C HSQC spectroscopy, as the corresponding signals were low intensity and within the aromatic signals of the Ph groups (Figures S13, S14, S29 and S31). The [M − Cl]^+^ fragment with the expected isotope pattern was observed in both cases in the mass spectra (Figures S62 and S63).

### 2.3. X-Ray Diffraction Analyses

**Figure 1:**
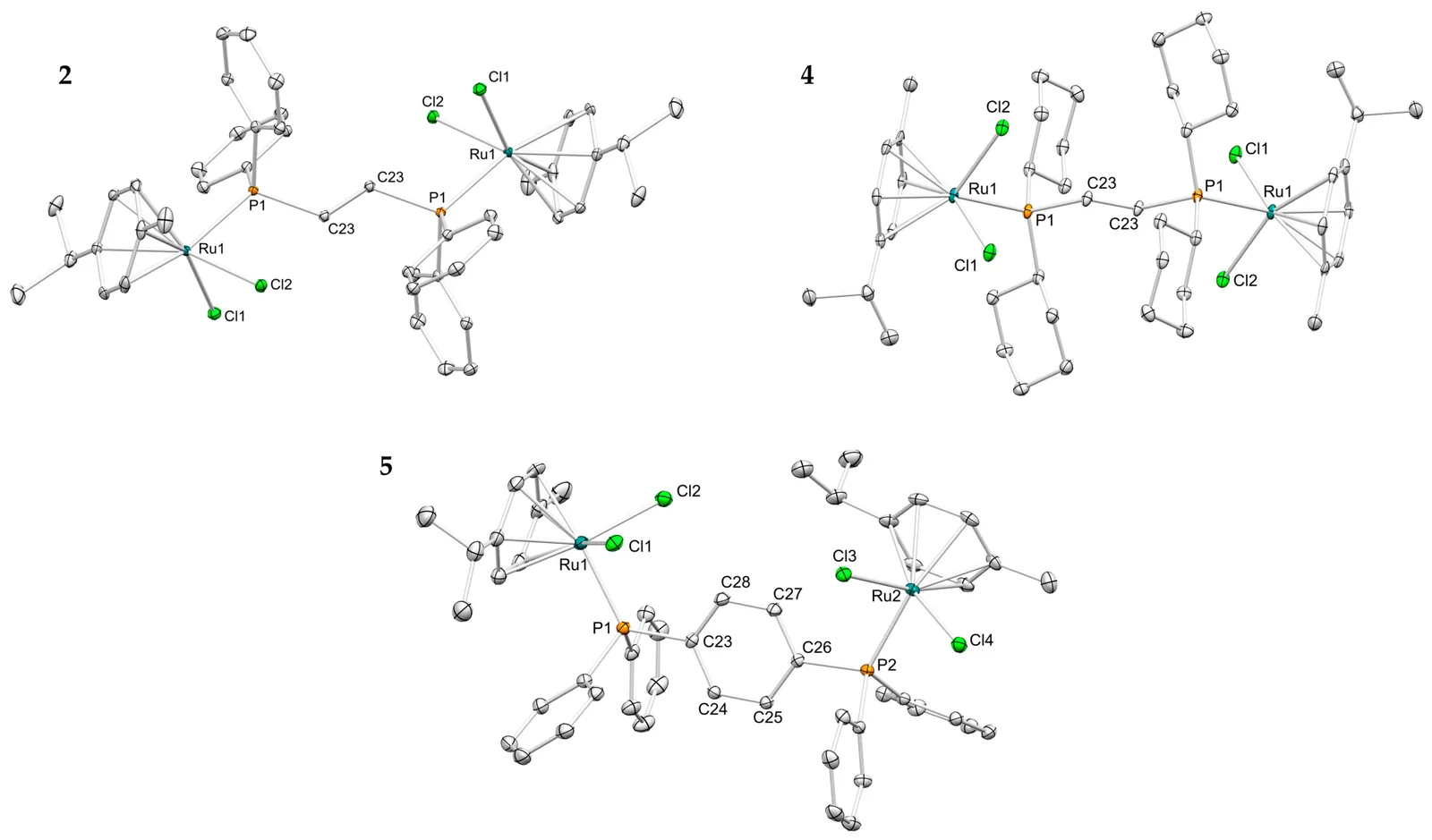
ORTEP representation of **2** (**top left**), **4** (**top right**) and **5** (**bottom**) in the solid state, determined by X-ray diffraction analyses. Thermal ellipsoids represented at the 30% probability level. H atoms and solvent molecules omitted for clarity.

**Figure 2:**
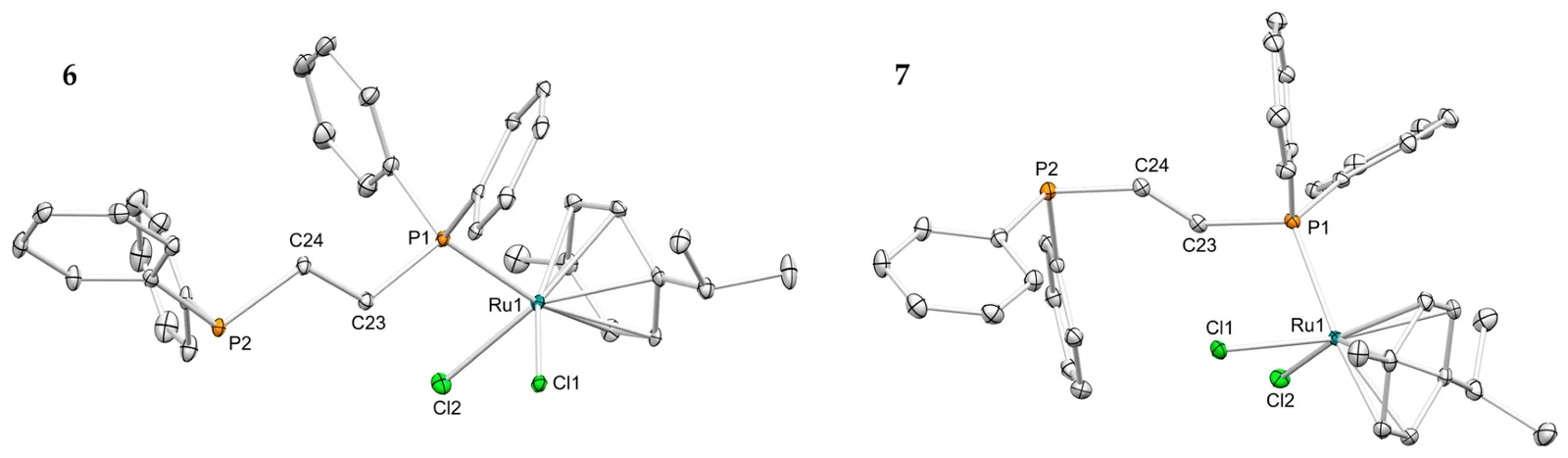
ORTEP representation of **6** (**left**), and **7** (**right**) in the solid state determined by X-ray diffraction analyses. Thermal ellipsoids represented at the 30% probability level. H atoms omitted for clarity.

The solid-state crystal structures of complexes **2**, **4** and **5** (Figure 1), as well as monomer complexes **6** and **7** (Figure 2), were determined via means of single-crystal X-ray diffraction analyses (see Table 2 for selected metrical parameters, bond lengths, and angles. See SI for complete data). Common to all complexes is the characteristic piano-stool configuration at Ru, where three binding sites are occupied by the η^6^-*p*-cymene moiety, whereas the remaining binding sites are occupied by two chlorides and a P atom from the bridging diphosphine. Complex **2** crystallised in the triclinic crystal system with the P-1 space group. A centre of inversion is observed between the two C atoms within the bridging tdppe. Two similar entities of **4** crystallised in the asymmetric unit, with the *P*2_1_/*c* space group of the monoclinic crystal system. Each of the two independent entities also has a centre of inversion in between the two C atoms of the bridging diphosphine. Complex **5** does not have a centre of inversion and crystallised in the triclinic crystal system and P-1 space group. Interestingly, the Ru atoms in **5** are *syn* to one another and lie within the C_6_H_4_ plane of 1,4dppb, with a dihedral angle of 10.60° between the Ru2-P2-C26 and Ru1-P1-C23 planes (Figure S71). In the case of the similar complex [(*p*-cym)RuCl_2_Ph_2_PCH_2_(*p*-C_6_H_4_)CH_2_Ph_2_P·BH_3_] reported by McQuade et al., which has extra methylene groups between *p*-C_6_H_4_ and P, the two P atoms are also *syn* to one another, and display a dihedral angle between the C_C6H4_-C_methylene_-P planes of 4.5°. However those planes are perpendicular to the C_6_H_4_ ring, which contrasts to complex **5**, for which both Ru-P-C planes are coplanar with each other, as well as with the C_6_H_4_ ring [42].

The average bond length between Ru and the six C of the η^6^-*p*-cymene unit is of 2.219 Å (**2**), 2.224 Å (**4**) and 2.220 Å (**5**), whereas the average Ru-Cl bond lengths are 2.410 (**2**), 2.419 (**4**) and 2.412 (**5**). The average Ru-P bond length is 2.340 (**2**), 2.395 (**4**) and 2.360 (**5**). These bond lengths are comparable within narrow margins to the structurally similar complexes [{(*p*-cym)RuCl_2_}_2_(µ-1,2-bis(diphenylphosphino)propane)] and [{(*p*-cym)RuCl_2_}_2_(µ-2,4-bis(diphenylphosphino)pentane)] reported by Klaimanee et al. [39], as well as the complex [{(C_6_H_6_)RuCl_2_}_2_(µ-1,1-bis(diphenylphosphino)methane)] reported by Herry et al. [33]. The Ru-Cl bond lengths of complex **3**, as reported by Chaplin et al. are also strikingly close to those of complexes **2**, **4** and **5** reporting herein, whereas the Ru-P bond is notably shorter than all complexes herein (Ru1-P1 (**3**), 2.319(11)) [41]. The nature of the diphosphine bridge seems therefore to only influence the Ru-P bond length, which was expected, while having minimal effect on the other ligands bound to Ru.

The bond angle Ru1-P1-C23 (see Figure 1 for atom labelling) varies greatly between the complexes, and follows the trend **4** < **2** < **5**, with angles of 110.53(9) (**4**) < 116.33(5) (**2**) < 121.75(9) (**5**). This trend can be tentatively explained by the increased rigidity along the series from the least strained diphosphine bridging ligand of complex **4**, which has *sp*^3^ P*C*H_2_*C*H_2_P bridging carbon atoms allowing for a low-strain conformation around P. Complex **2**, in contrast, experiences some strain due to the *sp*^2^ carbon atoms within the bridge, which renders the PCHCHP rigid and planar. Finally, the *para*-substituted benzene bridge in complex **5** introduces even more rigidity to the system, while also representing a more sterically bulky ligand. This might explain the increase in Ru1-P1-C23 bond angles along this series. The Ru1-P1-C23 bond angle in complex **3** is reported as 109.21(13) by Chaplin et al. [41], attributed to the linear PC≡CP bridge that allows for low steric strain at P. Although the solid state structures give insight into the molecular geometries of the complexes, no clear structure–activity relationships can be derived solely from the data obtained from single crystal X-ray diffraction analyses (see Section 2.3.).

The crystallographic Ru-Ru spatial distance follows the expected trend **3** (7.539 Å) [41] < **2** (8.238 Å) < **4** (8.446 Å) < **5** (8.648 Å). For complex **4**, the average of the two entities within the unit cell was calculated. The is no relationship between the Ru-Ru distance obtained via means of single crystal X-ray crystallography for these compounds and their respective in vitro cytotoxic activity. Although the molecules in solution would exhibit flexibility, the solid state Ru-Ru intramolecular distances do not explain the different biological activities, where complexes **3** and **5** are more active than the other complexes, while having the two Ru atoms the closest and furthest apart, respectively.

Monometallic complex **6** crystallised in the monoclinic crystal system with a *P*2_1_/*n* space group, while complex **7** crystallised in the monoclinic crystal system with a *P*2_1_/*c* space group. The geometry around Ru displays highly similar features as complexes **2**, **4** and **5**, with the characteristic piano-stool conformation. The main difference stems from the pendant P atom at the other end of the diphosphine, which shows the ability for this intermediate complex to bind another metal through the lone pair available on the pendant P. The borane-protected analogue **6·BH_3_** has been previously characterised by single crystal X-ray diffraction analysis [41], and present strikingly similar features as **6**.

### 2.4. In Vitro Cell Viability in HeLa and Ect1/E6E7

The in vitro cytotoxic effects in HeLa and Ect1/E6E7 cells were investigated in the range of 0–80 µM for each of the five complexes and compared against cisplatin as positive control (Figures S64–S67). From this data, complex **3** and **5** showed the lowest cell survival at 32 µM concentration, while complex **4** required a higher concentration (80 µM) to produce the same effect.

**Figure 3:**
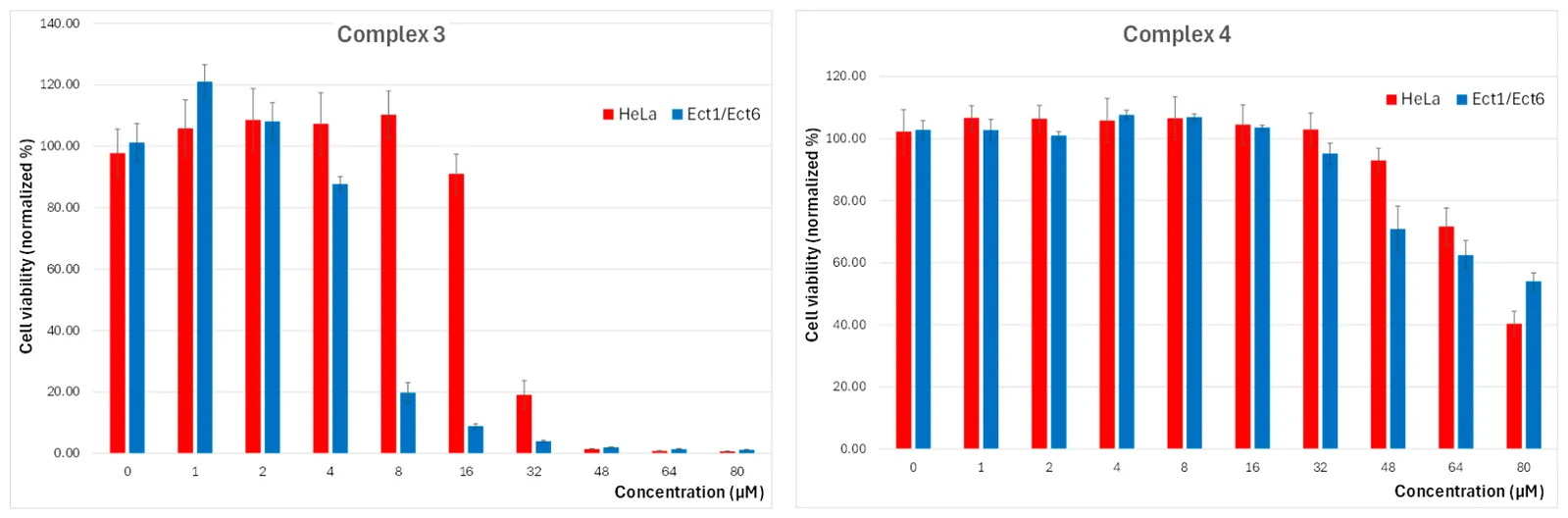
Presto blue cell cytotoxicity assay for Hela (red column) and Ect1/Ect6 (blue column) cells in presence of complex 3 (**left graph**) and complex 4 (**right graph**). Data are presented as mean ± SD (standard deviation) (n = 3).

Moreover, Ect1/E6E7 cells were included as non-malignant control cells due to their similarity to normal ectocervical tissue. Our aim was to identify the drug that has a higher specificity for cancer cells (HeLa) and less effect in the control cells (Ect1/Ect6) (Figure 3). Primarily we were concerned with studying the effect of changing the nature of the bridging ligand on the cytotoxicity of the different complexes.

IC_50_ values were calculated and are tabulated in Table 3. Complexes **3** and **5** displayed the most encouraging results against HeLa, with higher activity than cisplatin. On the other hand, these complexes also exhibit high cytotoxic activity against the Ect1/E6E7 healthy cell line, which is not desirable. Interestingly, complex **4**, while exhibiting a two-fold lower activity against HeLa cells, displayed a much lower cytotoxic profile in Ect1/E6E7 cells compared to cisplatin. Structurally, complexes **3** and **5** both have a rigid, linear bridging ligand, with dppa (**3**) and 1,4dppb (**5**). These ligands are also on the less basic side of the series, which might result in easier complex activation if the in vitro cytotoxic activity observed results from Ru-P bond breaking to form monomeric entities in solution (see Section 2.4).

In addition, a higher cell survival was observed in both cell lines after incubation with the five complexes at low concentration. This can be explained through the biphasic (hormetic) dose–response effect, where low-dose stimulates and high-dose inhibits cell survival [43,44]. Furthermore, hormesis is reported as a cellular response to “drug stressors”, where at low concentration it can trigger transcription of proteins involved in cell proliferation, survival and adaptive oxidative response pathways such as MAPK/ERK1/2 and PI3K/AKT [45,46,47,48]. Nevertheless, the IC_50_ values of these diruthenium complexes are suboptimal. The disparities in their values, however, clearly indicate a significant structure–activity relationship, solely based on the changing µ-diphosphine ligand. Complex **1**, as described by Das et al., was reported to have a suboptimal IC_50_ value of 61.3 µM against lung cancer H460 cell line [38]. The diruthenium complex [{(η^6^-C_6_H_6_)RuCl_2_}_2_(µ-bis(diphenylphosphino)methane)] was also reported to have a lower activity than cisplatin on ovarian cancer cell line A2780, even though it displayed slightly higher activity on cisplatin-resistant A2780cisR cell line [33]. The only complexes of the type [{(*p*-cym)RuCl_2_}_2_(µ-dppx)] that exhibited promising anticancer activities were reported by Klaimanee et al., which exhibited much higher cytotoxic activity than cisplatin in the breast cancer cell lines MCF-7, HCC1937 and MDA-MB-231 [39]. These previously reported findings, in addition to the ones we report herein, highlight the effect that the bridging diphosphine ligand itself has on the cytotoxic profiles of the complexes.

**Figure 4:**
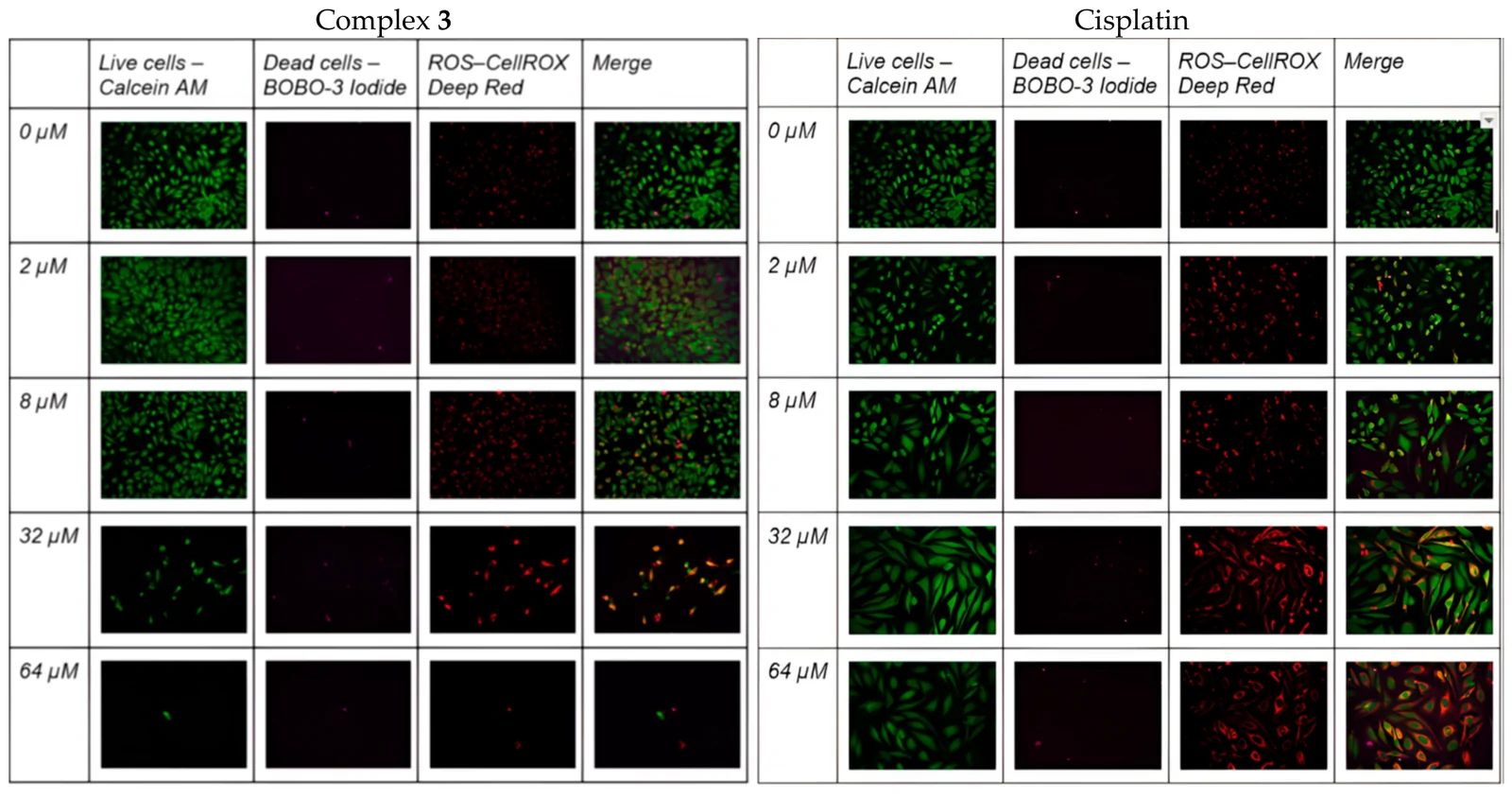
Fluorescence microscope imaging (objective magnification of 20×) for cells treated with complex **3** (**left**) and cisplatin (**right**) at various drug concentrations. HeLa cells were stained with Calcein AM (live cells), BOBO-3 Iodide (dead cells) and CellROX Deep Red (Reactive Oxygen Species, ROS). Each stain is individually observed for each condition and a composite (merge) image is presented of all stains in the Merge column.

To further investigate the potential mechanism of action of the complexes, HeLa cells were treated with complex **3** and then stained with Calcein AM (stains live cells), BOBO-3 iodide (stains dead cells) and CELLRox (stains Reactive Oxygen Species (ROS)) and visualised by fluorescence microscopy. For this experiment we selected complex **3** due to its apparent low-dose stimulating effect on cell viability of HeLa cells at concentrations lower than 8 µM and high-dose inhibition at concentrations above it. At concentrations above 8 µM, the number of dead cells does not rise substantially, but there is a decrease in live cells and an increase in ROS within the cells (Figure 4). Noteworthy, there is also ROS when the cells are exposed to only DMSO (0 µM: 5% DMSO in 95% cell medium) and their cell viability is slightly lower (~98%) compared to the cells exposed to complex **3** (at 8 µM, cell viability ~110%). This is consistent with predictions that ROS are produced in cells treated with complex **3** and may be responsible for inducing cellular stress. The observed higher cell count between 1 and 8 μM treatment compared to that of the control (0 µM) may be indicative of the hormetic effect. This is supported by data from the cell viability assays that at this concentration HeLa cells exhibit a hormetic response to the complexes. Based on the apparent cell shrinking morphology and high levels of ROS within the cells (Figure 4), it is tempting to speculate that complex **3** is inducing cytotoxicity by apoptosis [49]. Moreover, above 8 µM cisplatin treatment, HeLa cells exhibit an increased size morphology, reminiscent of cellular senescence rather than apoptosis and no hormetic effect was observed. Senescent cells cannot divide but are metabolically active and may be involved in accelerating tumorigenesis. Indeed, senescent cells secrete biomolecules that can modulate the tumour microenvironment, known as the senescence-associated secretory phenotype (SASP), giving rise to chemoresistant cancer cells [50]. In addition, chronic cellular senescence induced by low-dose cisplatin has also been reported to induce renal fibrosis and nephrotoxicity in kidney tubules [51,52]. Therefore, we demonstrated that Ruthenium complex **3** contributes to cellular apoptosis and has the advantage of not inducing cellular senescence, a known shortcoming of cisplatin [52,53,54,55,56].

### 2.5. Solution Stability Studies

In order to evaluate whether the complexes retain their bimetallic nature in solution, DMSO stability studies were performed via means of ^31^P{^1^H} NMR spectroscopy over time. After 48 h in pure DMSO-*d_6_*, a drop of PBS buffer solution in milliQ^®^ water was added to each sample and the ^31^P{^1^H} NMR spectra were recorded over time as well. The spectra can be found in Figures S32–S39. It is worth noting that the solubility in DMSO of complex **2** is very low, precluding NMR analysis. Interestingly, in pure DMSO-*d_6_*, complexes **1** and **4** did not display any change over the time frame of the experiment, whereas some minor changes were observed in the case of complexes **3** and **5**. Complex **5** displays a new signal at *δ* = −6.3 ppm which does not seem to increase in intensity after 6 h. Additionally, after 12 h, a minor signal is observed at *δ* = 25.3 ppm, likely from another species containing a P directly bound to Ru. Addition of PBS solution does not seem to further alter the identity of these species over time, nor the intensity of the signals. Complex **3**, however, displays the most striking changes over the course of the experiment. After 6 h in pure DMSO-*d_6_*, the most intense signal appears at *δ* = −33.3 ppm and appears as a doublet. In addition, a low intensity doublet signal appears at *δ* = 3.2 ppm, indicating the possible formation of [(*p*-cym)RuCl_2_(κ^1^-dppa)]. Interestingly, upon addition of PBS buffer solution, it seems like this process is reversed and after 48 h, only the bimetallic complex **3** is observed in solution again. This experiment shows that the higher in vitro cytotoxic activity of complexes **3** and **5** might result from activation of the complexes in solution. The low cytotoxic activity observed for complexes **1** and **4** might stem from their high stability in solution, inhibiting the formation of biologically active species. This goes to show the importance of careful design of the ligand sphere around the metal centre in the context of in vitro cytotoxic studies, as slight changes in electronic and steric structures can induce broadly different mechanisms of action.

In order to gain further insight into the differences in activity between the complexes, lipophilicity measurements were attempted. However, upon attempting to dissolve the complexes in either *n*-octanol or PBS buffer in MilliQ^®^ water, it was noted that solubility in those solvents was not high enough to allow for reliable data to be obtained. This rendered the “shake-flask” method impracticable for the complexes reported herein, and obtaining data from HPLC was not possible due to the nature of the complexes. Moreover, computational methods to determine lipophilicity are typically unreliable for metal-based complexes.

## 3. Conclusions

The synthesis and spectroscopic characterisation of five bimetallic complexes of the type [{(*p*-cym)RuCl_2_}_2_(µ-dppx)] have been reported, in addition to two novel monometallic analogous complexes. Five of those complexes were further characterised via single crystal X-ray diffraction analyses. The in vitro cytotoxic (anticancer) activity of all bimetallic complexes was tested against cervical cancer cell line HeLa, as well as healthy cell line Ect1/E6E7 to determine the potential change in activity and selectivity would be observed from modifying solely the bridging ligand structure of the complexes. Even though the complexes did not display encouraging anticancer activities or selectivities, fluctuations in IC_50_ values demonstrate that the bridging ligand itself plays a crucial role in the overall anticancer activity of these bimetallic complexes. It was demonstrated that even minor changes in ligand sphere induces different solution behaviours for seemingly analogous complexes, which leads invariably to different cytotoxic profiles. These findings highlight the importance of ligand design for µ-bridging diphosphine ligands in these and other bi- and multimetallic complexes.

## 4. Experimental Section

### 4.1. General Considerations

Standard Schlenk techniques were used unless otherwise stated. The chemicals, dichloromethane (99.8%, extra dry over molecular sieves, Thermo Fisher), diethyl ether (99.5%, extra dry over molecular sieves, stab BHT, Thermo Fisher), acetonitrile (LC-MS grade, Biosolve), ethyl acetate (AR grade, Biosolve), *n*-hexane (97%, extra dry over molecular sieves, Acros), pentane (98%, Aldrich), CDCl_3_ (99.8% D + 0.03% TMS *v*/*v*, VWR), [(*p*-cym)RuCl_2_]_2_ (Aldrich), 1,2-bis(diphenylphosphino)ethane (99%, Aldrich), *trans*-1,2-bis(diphenylphosphino)ethylene (97%, Aldrich), bis(diphenylphosphino)acetylene (97%, Strem), 1,2-bis(dicyclohexylphosphino)ethane (98%, Fisher), and 1,4-bis(diphenylphosphino)benzene (98%, BLD pharm), were used as received without further purification. The solvents used for synthetic procedures were degassed through N_2_(g) bubbling prior to use. NMR spectra were recorded on a JEOL JNM-ECZ400s FT NMR spectrometer. Raw NMR data was processed using Mnova version 14.3.0. The data is reported in ppm (parts per million) and referenced to TMS (^1^H and ^13^C) or phosphoric acid (^31^P). UV–Vis spectra were recorded on a Shimadzu UV-3600 iPlus-1 spectrometer, using 1 cm path length quartz cuvettes. FT-IR data was acquired on a Shimadzu IRSpirit FTIR spectrometer at 32 scans, 2 cm^−1^ resolution, Happ-Genzel apodization with a scan range of 4000–400 cm^−1^. FT-IR and UV–Vis data was processed using OriginPro 2018 SR1 b9.5.1.195. Abbreviations (NMR): s = singlet, d = doublet, t = triplet, sept = septet, m = multiplet, ps = pseudo, br = broad. Abbreviations (FT-IR): s = strong, m = medium, w = weak. Single crystal X-ray diffraction analysis was performed on a Rigaku XtaLAB Synergy, Dualflex, HyPix-Arc 100 diffractometer using Cu radiation (λ = 1.54184 Å). CrysAlisPro Software System (v1.171.43.92a, Rigaku Oxford Diffraction, 2026) was used to reduce data to F_o_^2^ and corrected for absorption effects. Structures were solved using SHELXT [57] and refined with SHELXL [58] as implemented in OLEX2 [59]. The cell lines used for the in vitro cytotoxic tests were purchased from ATCC (American Type Culture Collection; HeLa CCL-2, Ect1/E6E7 CRL-2614).

### 4.2. General Procedure for the Synthesis of the Novel Homobimetallic Complexes [{(p-cym)RuCl_2_}_2_(µ-Diphosphine)]

Complexes **1**–**5** were synthesised in an analogous manner. [(*p*-cym)RuCl_2_]_2_ and the respective diphosphine were dissolved in equimolar amounts in CH_2_Cl_2_ and stirred for 2 h at room temperature. The solvents were then evaporated in vacuo, and the resulting powder was washed with Et_2_O (5 × 5 mL). The product was subsequently dried in vacuo overnight. Characterisation of the known complexes **1** and **3** is included in the Supporting Information. The procedure used herein gives higher yields than previously reported methodologies for the synthesis of those known compounds [38,41,60,61,62,63].

#### 4.2.1. Characterisation of [{(p-cym)RuCl_2_}_2_(µ-tdppe)] (**2**)

In total, 300 mg of [(*p*-cym)RuCl_2_]_2_ (0.490 mmol) and 194 mg of tdppe (0.490 mmol) was used. A total of 410 mg (0.406 mmol, 83%) of dark red solid was obtained, air stable. Crystals suitable for X-ray diffraction analysis were grown via slow evaporation of a concentrated CH_2_Cl_2_ solution. ^1^H NMR (400.130 MHz, CDCl_3_, 293 K): *δ* 7.73–7.65 (8H, m, P*Ph*), 7.39–7.28 (12H, m, P*Ph*), 6.94 (2H, t, *J*_H-P_ = 18.9 Hz, PC*H* = C*H*P), 5.34 (4H, ps d, ^3^*J*_H-H_ = 6.06 Hz, C^2,6^*H*, *p*-cym), 5.31 (4H, ps d, ^3^*J*_H-H_ = 6.06 Hz, C^3,5^*H*, *p*-cym), 2.39 (2H, sept, ^3^*J*_H-H_ = 6.96 Hz, C*H*(CH_3_)_2_), 1.72 (6H, s, C*H*_3_), 0.93 (12H, d, ^3^*J*_H-H_ = 6.93 Hz, CH(C*H*_3_)_2_). ^13^C{^1^H} NMR (100.613 MHz, CDCl_3_, 293 K): *δ* 140.2–139.6 (m, P*C*H = *C*HP), 134.1 (t, ^2^*J*_C-P_ = 4.6 Hz, *C*^2^, Ph), 133.0–132.4 (m, *C*^1^, Ph), 130.5 (s, *C*^4^, Ph), 128.3 (t, ^3^*J*_C-P_ = 5.0 Hz, *C*^3^, Ph), 109.2 (s, *C*^1^, *p*-cym), 97.7 (s, *C*^4^, *p*-cym), 88.3 (s, *C*^2,6^, p-cym), 86.7 (s, *C*^3,5^, *p*-cym), 30.1 (s, *C*H(CH_3_)_2_), 21.9 (s, CH(*C*H_3_)_2_), 17.5 (s, *C*H_3_). ^31^P{^1^H} NMR (161.976 MHz, CDCl_3_, 293 K): *δ* 24.04 (s, tdppe-*P*). FT-IR (cm^−1^): *ν* 3036 (w), 2960 (w), 2863 (w), 1433 (m), 1191 (m), 1095 (m), 877 (m), 752 (m), 740 (m), 696 (s). UV–Vis *λ*_max_ (dichloromethane): 225 nm (major, ϵ = 66171 M^−1^cm^−1^), 249 nm (shoulder, ϵ = 44362 M^−1^cm^−1^), 375 (minor, ϵ = 4438 M^−1^cm^−1^). ESI-TOF-MS (ACN): *m*/*z* calcd. for [M + Na]^+^, 1031.0131; expt., 1031.0393 (100%). *m*/*z* calcd. for [M − Cl]^+^, 973.0549; expt., 973.0767 (1.3%).

#### 4.2.2. Characterisation of [{(p-cym)RuCl_2_}_2_(µ-dcpe)] (**4**)

In total, 250 mg of [(*p*-cym)RuCl_2_]_2_ (0.410 mmol) and 172.5 mg of dcpe (0.410 mmol) were used. A total of 375 mg (0.362 mmol, 88%) of dark orange powder was obtained, air stable. Crystals suitable for X-ray diffraction analysis were grown via slow diffusion of pentane into a concentrated THF solution. ^1^H NMR (400.130 MHz, CDCl_3_, 293 K): *δ* 5.55 (4H, ps d, ^3^*J*_H-H_ = 6.38 Hz, C^2,6 or 3,5^*H*, *p*-cym), 5.53 (4H, ps d, ^3^*J*_H-H_ = 6.40 Hz, C^2,6 or 3,5^*H*, *p*-cym), 2.79 (2H, sept, ^3^*J*_H-H_ = 6.95 Hz, C*H*(CH_3_)_2_), 2.30 (4H, d, ^2^*J*_H-P_ = 3.69 Hz, PC*H*_2_C*H*_2_P), 2.26–2.09 (8H, m, Cy), 2.06 (6H, s, C*H*_3_, *p*-cym), 1.89–1.76 (12H, m, Cy), 1.69 (4H, br s, Cy), 1.63–1.48 (6H, m, Cy), 1.31–1.23 (14H, m, Cy), 1.26 (12H, d, ^3^*J*_H-H_ = 6.96 Hz, CH(C*H*_3_)_2_). ^13^C{^1^H} NMR (100.613 MHz, CDCl_3_, 293 K): *δ* 108.9 (s, *C*^1^, *p*-cym), 94.2 (s, *C*^4^, *p*-cym), 88.5 (s, *C*^2,6 or 3,5^, *p*-cym), 82.6 (s, *C*^2,6 or 3,5^, *p*-cym), 37.6 (d, ^x^*J*_C-P_ = 21.5 Hz, Cy), 30.9 (s, *C*H(CH_3_)_2_), 29.5 (s, Cy), 28.6 (s, Cy), 27.7–27.4 (m, Cy), 26.6 (s, Cy), 22.5 (s, CH(*C*H_3_)_2_), 18.3 (s, *C*H_3_, *p*-cym), 14.5 (d, ^1^*J*_C-P_ = 9.66 Hz, P*C*H_2_*C*H_2_P). ^31^P{^1^H} NMR (161.976 MHz, CDCl_3_, 293 K): *δ* 30.40 (s, dcpe-*P*). FT-IR (cm^−1^): *ν* 3035 (w), 2932 (s), 2917 (s), 2851 (m), 2841 (m), 1443 (m), 1003 (m), 885 (m), 862 (m), 849 (m), 739 (m), 668 (w), 517 (m). UV–Vis *λ*_max_ (dichloromethane): 226 (major, ϵ = 32190 M^−1^cm^−1^), 359 (minor, ϵ = 2970 M^−1^cm^−1^). ESI-TOF-MS (ACN): *m*/*z* calcd. for [M − Cl]^+^, 999.2584; expt., 999.3219 (100%).

#### 4.2.3. Characterisation of [{(p-cym)RuCl_2_}_2_(µ-1,4dppb)] (**5**)

In total, 100 mg of [(*p*-cym)RuCl_2_]_2_ (0.163 mmol) and 73 mg of 1,4dppb (0.163 mmol) were used. A total of 135 mg (0.128 mmol, 78%) of brick-like powder was obtained, air stable. Crystals suitable for X-ray diffraction analysis were grown via slow diffusion of Et_2_O into a concentrated CH_2_Cl_2_ solution. ^1^H NMR (400.130 MHz, CDCl_3_, 293 K): *δ* 7.84–7.76 (8H, m, P*Ph*), 7.75–7.68 (4H, m, 1,4dppb), 7.43–7.32 (12H, m, P*Ph*), 5.18 (4H, ps d, ^3^*J*_H-H_ = 6.2 Hz, C^3,5^-*H*, *p*-cym), 4.97 (4H, ps d, ^3^*J*_H-H_ = 6.1 Hz, C^2,6^-*H*, *p*-cym), 2.81 (2H, sept, ^3^*J*_H-H_ = 7.1 Hz, C*H*(CH_3_)_2_), 1.82 (6H, s, C*H*_3_, *p*-cym), 1.09 (12H, d, ^3^*J*_H-H_ = 7.0 Hz, CH(C*H*_3_)_2_). ^13^C{^1^H} NMR (100.613 MHz, CDCl_3_, 293 K): *δ* 136.3 (d, ^1^*J*_C-P_ = 44.5 Hz, *C*^1,4^, 1,4dppb), 134.6 (d, ^3^*J*_C-P_ = 9.8 Hz, *C*^3^, Ph), 133.4–132.8 (m, *C*^1^, Ph + *C*^2,3,5,6^, 1,4dppb), 130.6 (s, *C*^4^, Ph), 128.2 (d, ^2^*J*_C-P_ = 16.1 Hz, *C*^2^, Ph), 111.3 (d, ^2^*J*_C-P_ = 3 Hz, *C*^1^, *p*-cym), 96.3 (s, *C*^4^, p-cym), 89.2 (d, ^2^*J*_C-P_ = 3 Hz, *C*^3,5 or 2,6^, *p*-cym), 87.3 (d, ^2^*J*_C-P_ = 5,2 Hz, *C*^3,5 or 2,6^, *p*-cym), 30.4 (s, *C*H(CH_3_)_2_), 22.0 (s, CH(*C*H_3_)_2_), 17.9 (s, *C*H_3_). ^31^P{^1^H} NMR (161.976 MHz, CDCl_3_, 293 K): *δ* 25.2 (s, 1,4dppb-*P*). FT-IR (cm^−1^): *ν* 3049 (w), 2963 (w), 2924 (w), 2870 (w), 1482 (w), 1433 (m), 1377 (w), 1094 (m), 747 (m), 697 (m), 550 (s), 539 (s), 520 (s), 438 (m). UV–Vis *λ*_max_ (dichloromethane): 225 (major, ϵ = n/a), 251 (major, ϵ = 34876 M^−1^cm^−1^), 379 (minor, ϵ = 3505 M^−1^cm^−1^). ESI-TOF-MS (ACN): *m*/*z* calcd. for [M + Na]^+^, 1081.0289; expt., 1081.0870 (0.7%).

### 4.3. Synthesis of [(p-cym)RuCl_2_(κ^1^-dppe)] (***6***)

In total, 260 mg of dppe (0.653 mmol, 2 eq.) were dissolved in 10 mL of dichloromethane and cooled down to −78 °C. A total of 200 mg of [(*p*-cym)RuCl_2_]_2_ (0.327 mmol, 1 eq.) were dissolved in 20 mL dichloromethane and added dropwise to the tdppe solution. The resulting solution was stirred for 5 min after which the solvents were removed *in vacuo*. The monodentate product was purified by column chromatography on a silica column using a 1:1 solvent mixture of EtOAc: *n*-hexane. The solvents were evaporated and the product was washed with Et_2_O (3 × 3 mL) and dried in vacuo to yield 45.7 mg of orange powder (0.065 mmol, 10%). Crystals suitable for X-ray diffraction analysis were grown via slow diffusion of degassed pentane in a concentrated degassed CH_2_Cl_2_ solution. Stability: stable in air, converts partially to dinuclear species **1** and bidentate species [(*p*-cym)RuCl(κ^2^-dppe)]^+^Cl^-^ in solution over time. Crystals suitable for X-ray diffraction analysis were grown via slow diffusion of pentane into a concentrated degassed CH_2_Cl_2_ solution. ^1^H NMR (400.130 MHz, CDCl_3_, 293 K): *δ* 7.72 (4H, m, P*Ph*), 7.49–7.37 (6H, m, P*Ph*), 7.23–7.10 (10H, m, P*Ph*), 5.18 (2H, dd, ^3^*J*_H-H_ = 6.3, 1.5 Hz, C^3,5^*H*, *p*-cym), 5.02 (2H, d, ^3^*J*_H-H_ = 6.1 Hz, C^2,6^*H*, *p*-cym), 2.68–2.58 (2H, m, PC*H*_2_CH_2_P), 2.51 (1H, p, ^3^*J*_H-H_ = 6.9 Hz, *p*-cym C*H*(CH_3_)_2_), 1.84 (3H, s, C*H*_3_), 1.79–1.71 (2H, m, PCH_2_C*H*_2_P), 0.80 (6H, d, ^3^*J*_H-H_ = 6.9 Hz, CH(C*H_3_*)_2_). ^13^C{^1^H} NMR (100.613 MHz, CDCl_3_, 293 K): *δ* 138.13–128.38 (m, P*Ph*), 108.57 (s, *C*^1^, *p*-cym), 94.02 (*C*^4^, *p*-cym), 90.55 (d, ^2^*J*_C-P_ = 4.36 Hz, *C*^3,5^, *p*-cym), 85.65 (d, ^2^*J*_C-P_ = 5.96 Hz, *C*^2,6^, *p*-cym), 30.02 (s, *C*H(CH_3_)_2_), 21.71 (m, P*C*H_2_CH_2_P, assigned via ^1^H-^13^C HSQC), 21.45 (s, CH(*C*H_3_)_2_), 20.76 (m, PCH_2_*C*H_2_P, assigned via ^1^H-^13^C HSQC), 17.45 (s, *C*H_3_). ^31^P{^1^H} NMR (161.976 MHz, CDCl_3_, 293 K): *δ* 26.16 (d, ^3^*J*_P-P_ = 34.98 Hz, Ru*P*Ph_2_), −12.05 (d, ^3^*J*_P-P_ = 34.98 Hz, *P*Ph_2_). FT-IR (cm^−1^): *ν* = 3047 (w), 2972 (w), 2949 (w), 2914 (w), 1483 (w), 1431 (m), 1414 (w), 1097 (m), 1029 (w), 885 (w), 868 (w), 742 (s), 696 (s), 673 (m), 523 (s), 477 (m), 454 (m). ESI-MS (ACN), *m*/*z* calculated for [M − Cl]^+^ 669.12. Found: 669.10.

### 4.4. Synthesis of [(p-cym)RuCl_2_(κ^1^-tdppe)] (***7***)

In total, 258.9 mg of tdppe (0.653 mmol, 2 eq.) and 200 mg of [(*p*-cym)RuCl_2_]_2_ (0.327 mmol, 1 eq.) were dissolved in 10 mL each in separate flasks. The [(*p*-cym)RuCl_2_]_2_ solution was added dropwise to the tdppe solution at room temperature and the resulting solution was stirred at room temperature for 1 h. The solvent was removed in vacuo and a column chromatography on silica gel was performed using a three solvent mixture of 5:3.5:1.5 mixture of dichloromethane: *n*-hexane: EtOAc. The solvents were removed in vacuo and the product was dried under reduced pressure to yield 17.7 mg (0.025 mmol, 8%). Crystals suitable for X-ray diffraction analysis were grown via slow diffusion of degassed pentane in a concentrated degassed CH_2_Cl_2_ solution. Stability: stable in air, converts partially to dinuclear species **2** in solution over time. Crystals suitable for X-ray diffraction analysis were grown via slow diffusion of pentane into a concentrated degassed CH_2_Cl_2_ solution. ^1^H NMR (400.130 MHz, CDCl_3_, 293 K): *δ* 7.79–7.71 (4H, m, P*Ph*), 7.46–7.35 (10H, m, P*Ph*), 7.29 (6H, m, P*Ph*), 6.76 (1H, m, PC*H* = CHP), 6.45 (1H, m, PCH = C*H*P), 5.16 (4H, d, ^3^*J*_H-H_ = 6.7 Hz, C^3,5 or 2,6^*H*, *p*-cym), 2.56 (1H, sept, ^3^*J*_H-H_ = 7.0 Hz, C*H*(CH_3_)_2_), 1.81 (3H, s, C*H*_3_), 0.95 (6H, d, ^3^*J*_H-H_ = 7.0 Hz, CH(C*H*_3_)_2_). ^13^C{^1^H} NMR (100.613 MHz, CDCl_3_, 293 K): *δ* 145.69–128.29 (m, P*Ph* + P*C*H = CHP (assigned via ^1^H-^13^C HSQC)), 109.14 (s, *C*^1^, *p*-cym), 95.33 (s, *C*^4^, *p*-cym), 89.44 (d, ^2^*J*_C-P_ = 4.43 Hz, *C*^3,5 or 2,6^, *p*-cym), 85.83 (d, ^2^*J*_C-P_ = 5.91 Hz, *C*^3,5 or 2,6^, *p*-cym), 30.20 (s, *C*H(CH_3_)_2_), 21.79 (s, CH(*C*H_3_)_2_), 17.59 (s, *C*H_3_) ppm. ^31^P{^1^H} NMR (161.976 MHz, CDCl_3_, 293 K): *δ* 22.65 (d, ^3^*J*_P-P_ = 8.1 Hz, Ru*P*PH_2_), −4.86 (d, ^3^*J*_P-P_ = 8.1 Hz, *P*PH_2_) ppm. FT-IR (cm^−1^): *ν* = 3052 (w), 3029 (w), 2960 (w), 2868 (w), 1730 (w), 1477 (w), 1431 (m), 1380 (w), 1242 (w), 1178 (w), 1098 (m), 1029 (w), 966 (w), 874 (w), 799 (w), 742 (s), 690 (s), 512 (s), 466 (s). ESI-MS (ACN), *m*/*z* calculated for [M − Cl]^+^ 667.10. Found: 667.10.

### 4.5. Cell Viability Assays

The two types of cells were cultured according to the ATCC protocols under aseptic conditions: Cervical Cancer Cells (HeLa) (ATCC CCL-2) and as a control an Ectocervix Epithelial Cell Line Ect1/E6E7 (ATCC CRL-2614). Cell viability assays were performed using Presto Blue HS cell viability reagent (Invitrogen, Cat# A13261, Thermo Fisher Scientific, Waltham, MA, USA), following the manufacturer’s instructions.

Briefly, cells were seeded in 96-well plates at 5000 cells/mL density for HeLa and for was 7000 cells/mL density for Ect1/E6E7. To allow cell adhesion, cells were incubated 24 h prior to drug treatment. The cells were then incubated with the corresponding complexes and cisplatin for 72 h. Afterwards, Presto Blue HS cell viability reagent was used. After 20 min incubation with 10 μL Presto Blue, the fluorescence intensity was measured in a Tecan (Cat# 30050303) microplate well reader (excitation/emission = 560/590 nm). Wells containing only cell medium were used as controls to Normalise the Absorbance values during the different conditions. At least three independent measurements were performed to determine the IC_50_ concentration of the respective complexes. The IC_50_ values were calculated from the sigmoidal plots with Quest Graph™ IC_50_ Calculator, AAT Bioquest. https://www.aatbio.com/tools/ic50-calculator (accessed on 27 December 2025).

Cells prepared for fluorescence microscopy were seeded as mentioned above. Imaging was performed by Dr. Anne Gemmink using the FEI Munich CorrSight Cryo inverted microscope, located in the Microscopy Core laboratories, Maastricht University. Images were taken of live cells at 20× magnification and in the 2D optical slice plane for quick, high-resolution images of the cell monolayer.

A Live/Dead cell staining kit was used (Invitrogen, Cat# R37601, Thermo Fisher Scientific) which included Calcein AM to stain live cells cytoplasm and BOBO-3 iodide was used to stain the nuclei of dead cells. In addition, CellROX™ deep red dye (Invitrogen, Cat# C10448, Thermo Fisher Scientific) was used in order to visualise ROS. Fluorescent imaging was observed using the following excitation/emission wavelengths; Calcein AM: 488 nm/515 nm, BOBO-3 iodide: 570 nm/602 nm, CellROX™deep red: 640 nm/665 nm. The CellROX™ deep red solution was prepared in a 5 μM solution by diluting the 2.5 mM stock solution with medium. After 72 h drug incubation, the medium was aspirated from the cells on the 96 well plate and 50 μL of the 5 μM dye solution was added to each well and left to incubate at 37 °C for 30 min. In the meantime, the live/dead stain was prepared by pipetting 1 mL of the live green stain into the vial containing 1 μL of the dead red stain, and the solution was homogenised by gently pipetting.

Once the 30 min incubation was over the dye-containing medium was aspirated and the wells were gently washed with 1× PBS. The live/dead stain solution was then added again at 50 mL per well and left to incubate for 15 min at room temperature. Finally, the cells were carefully washed with 1× PBS three times before being left in 80 μL PBS. Plates were imaged within 2 h of preparation.

## Figures and Tables

**Table 1:** UV–Vis absorption maxima and related molar extinction coefficients of complexes **1**–**5**.

Complex	Absorption Maxima λ_max_ in nm (Molar Extinction Coefficients ε in L·mol^−1^·cm^−1^)		
	λ_1_	λ_2_	λ_3_
**1**	226 (49,238)	245 (34,229)	370 (3619)
**2**	225 (66,171)	249 (44,362)	375 (4438)
**3**	226 (49,810)	n/a *	384 (3248)
**4**	226 (32,190)	n/a *	361 (2971)
**5**	225 (n.d. **)	251 (34,876)	378 (3505)

* n/a = not applicable; ** n.d. = not determined.

**Table 2:** Crystallographic data, selected bond lengths and angles for complexes **2**, **4**, **5**, **6** and **7**.

Complex Label	2	4	5	6	7
Empirical formula	C_24_H_27_Cl_4_PRu	C_46_H_76_Cl_4_P_2_Ru_2_	C_50.5_H_53_Cl_5.06_P_2_Ru_2_	C_72_H_76_Cl_4_P_4_Ru_2_	C_72_H_72_Cl_4_P_4_Ru_2_
Temperature/K	100.00 (10)	100.0 (3)	100.00 (10)	100.00	101 (1)
Crystal system	triclinic	monoclinic	triclinic	monoclinic	monoclinic
Space group	P-1	P2_1_/c	P-1	P2_1_/n	P2_1_/c
a/Å	11.0487 (3)	21.3803 (2)	10.09180 (10)	12.59299 (10)	9.93542 (4)
b/Å	11.3414 (3)	15.64630 (10)	14.2482 (2)	17.95904 (15)	15.54235 (7)
c/Å	11.9805 (3)	13.87320 (10)	18.8810 (3)	15.11860 (15)	20.93213 (10)
α/°	80.712 (2)	90	73.7490 (10)	90	90
β/°	64.941 (2)	96.8390 (10)	85.0770 (10)	106.4350 (9)	98.6808 (4)
γ/°	63.925 (2)	90	87.0960 (10)	90	90
Volume/Å^3^	1220.96 (6)	4607.88 (6)	2595.80 (6)	3279.49 (5)	3195.31 (3)
Z	2	4	2	2	2
ρ_calc_ g/cm^3^	1.603	1.492	1.412	1.427	1.460
F(000)	596.0	2152.0	1120.0	1448.0	1440.0
Radiation	Mo Kα (λ = 0.71073)	Cu Kα (λ = 1.54184)	Cu Kα (λ = 1.54184)	Cu Kα (λ = 1.54184)	Cu Kα (λ = 1.54184)
2Θ range for data collection/°	5.404 to 66.184	7.018 to 159.982	6.464 to 160.496	7.836 to 159.388	8.546 to 160.628
Index ranges	−16 ≤ h ≤ 16, −17 ≤ k ≤ 16, −18 ≤ l ≤ 17	−19 ≤ h ≤ 26, −19 ≤ k ≤ 19, −17 ≤ l ≤ 17	−12 ≤ h ≤ 12, −16 ≤ k ≤ 18, −23 ≤ l ≤ 24	−11 ≤ h ≤ 15, −22 ≤ k ≤ 22, −19 ≤ l ≤ 18	−11 ≤ h ≤ 12, −19 ≤ k ≤ 18, −26 ≤ l ≤ 26
Reflections collected	44,250	80,964	67,005	70,878	71,850
Independent reflections	8266 [R_int_ = 0.0391, R_sigma_ = 0.0320]	9882 [R_int_ = 0.0417, R_sigma_ = 0.0231]	11,124 [R_int_ = 0.0396, R_sigma_ = 0.0283]	7093 [R_int_ = 0.0577, R_sigma_ = 0.0250]	6989 [R_int_ = 0.0473, R_sigma_ = 0.0233]
Data/restraints/parameters	8266/0/278	9882/0/493	11,124/0/567	7093/0/374	6989/0/374
Goodness-of-fit on *F*2	1.051	1.056	1.045	1.125	1.057
Final R indexes [*I* ≥ 2σ (*I*)]	R_1_ = 0.0273, wR_2_ = 0.0586	R_1_ = 0.0378, wR_2_ = 0.0976	R_1_ = 0.0360, wR_2_ = 0.0914	R_1_ = 0.0363, wR_2_ = 0.0916	R_1_ = 0.0261, wR_2_ = 0.0649
Final R indexes [all data]	R_1_ = 0.0327, wR_2_ = 0.0602	R_1_ = 0.0401, wR_2_ = 0.0989	R_1_ = 0.0386, wR_2_ = 0.0930	R_1_ = 0.0367, wR_2_ = 0.0919	R_1_ = 0.0264, wR_2_ = 0.0651
Largest diff. peak/hole/eÅ^−3^	0.90/−0.73	1.09/−1.59	1.95/−1.08	1.30/−1.42	0.84/−0.49
Selected bond lengths (Å)					
Ru1-Cl1	2.4070 (4)	2.4181 (7)	2.4043 (8)	2.4157(6)	2.4027 (4)
Ru1-Cl2	2.4121 (4)	2.4266 (7)	2.4177 (8)	2.4157 (6)	2.4103 (4)
Ru1-P1	2.3404 (4)	2.3946 (7)	2.3578 (7)	2.3537 (6)	2.3490 (4)
P1-C23	1.8189 (14)	1.852 (3)	1.835 (3)	1.834 (3)	1.8113 (18)
C23-C23 ^1 a^	1.334 (3)	1.522 (5)	n/a *	n/a *	n/a *
C23-C24 ^b^	n/a *	n/a *	n/a *	1.532 (3)	1.324 (3)
C24-P2 ^b^	n/a *	n/a *	n/a *	1.860 (3)	1.8244 (18)
Selected bond angles (°)					
Cl1-Ru1-Cl2	87.967 (13)	86.48 (3)	89.30 (3)	88.36 (2)	88.846 (15)
Cl2-Ru1-P1	83.734 (12)	87.38 (2)	90.07 (3)	88.69 (2)	84.707 (15)
P1-Ru1-Cl1	85.914 (13)	87.88 (2)	85.44 (3)	82.97 (2)	83.944 (14)
Ru1-P1-C23	116.33 (5)	110.53 (9)	121.75 (9)	113.35 (8)	112.60 (6)

^a^ Applicable to complexes **2** and **4** only. ^b^ Applicable to complexes **6** and **7** only. * n/a = not applicable.

**Table 3:** IC_50_ values (µM) for cisplatin and complexes **1**–**5** calculated in Hela and Ect1/E6E7cells using Presto Blue assays after 72 h exposure. Data are presented as mean ± SD (standard deviation) (*n* = 3).

Drug	HeLa	Ect1/E6E7
Cisplatin	35.62 ± 7.47	8.46 ± 0.32
**1**	>80	>80
**2**	73.73 ± 16.05	38.95 ± 1.89
**3**	23.32 ± 1.27	5.55 ± 0.16
**4**	73.34 ± 5.21	77.92 ± 9.50
**5**	27.59 ± 0.61	7.98 ± 0.31

## Data Availability

Supplementary Information (SI): Experimental procedures and full characterisation of the complexes, including all spectra and X-ray data. CCDC 2521032-2521036 contain the supplementary crystallographic data for this paper.

## Supplementary Materials

The following supporting information can be downloaded at: https://www.mdpi.com/article/10.3390/molecules31172968/s1, Figures S1–S7: ^1^H NMR spectra; Figures S8–S14: ^13^C{^1^H} NMR spectra; Figures S15–S21: ^31^P{^1^H} NMR spectra; Figures S22–S31: 2D NMR spectra; Figures S32–S39: Solution stability studies; Figures S40–S46: FT–IR spectra; Figures S47–S56: UV–Vis spectra; Figures S57–S63: Mass Spectra; Figures S64–S66: Cell viability studies; Figure S67: IC_50_ plots; Figures S68–S73 and Tables S1–S37: Singles crystal X–ray diffraction data.
