# Supplementary material for: Effect of the µ-Diphosphine Bridging Ligand on the Cytotoxic Activity of Homodinuclear Ruthenium Complexes
Source: Molecules. 2026 Aug 25;31(17):2968. doi: 10.3390/molecules31172968 (PMC13566926; doi:10.3390/molecules31172968)
Supplement: Supplementary file 1 [file molecules-31-02968-s001.zip › molecules-4424137-supplementary.pdf]

SUPPORTING INFORMATION

# Effect of the μ-Diphosphine Bridging Ligand on the Cytotoxic Activity of Homodinuclear Ruthenium Complexes


1. Characterisation of complexes **1** and **3** ........ 2

2. $^{1}$H NMR spectra ........ 3

3. $^{13}C\{^{1}H\}$ NMR spectra ........ 6

4. $^{31}P\{^{1}H\}$ NMR spectra ........ 10

5. 2D NMR spectra ........ 13

6. Solution stability studies ........ 18

7. FT-IR spectra ........ 22

8. UV-Vis spectra ........ 26

9. Mass spectra ........ 35

10. Cell viability studies ........ 41

11. $IC_{50}$ plots ........ 43

12. Single crystal X-ray diffraction analyses ........ 44

13. References ........ 100

1. Characterisation of complexes **1** and **3**

$[(p\text{-cym})RuCl_2]_2(\mu\text{-dppe})$ (**1**)

200 mg of $[(p\text{-cym})RuCl_2]_2$ (0.327 mmol, 1 eq.) and 130 mg (0.327 mmol, 1 eq.) used, 298 mg (0.295 mmol, 90 %) of orange powder obtained, air stable. The NMR spectroscopic characterisation matches with the literature [1]. $^1H$ NMR (400.130 MHz, $CDCl_3$, 293 K): $\delta$ 7.62 – 7.54 (8 H, m, P*Ph*), 7.42 – 7.29 (12 H, m, P*Ph*), 5.24 (4 H, d, $^2J_{H\text{-}H}$ = 6.0 Hz, $C^{2,6\text{ or }3,5}H$, *p*-cym), 5.07 (4 H, d, $^2J_{H\text{-}H}$ = 6.0 Hz, $C^{2,6\text{ or }3,5}H$, *p*-cym), 2.45 (4 H, m, $PCH_2CH_2P$, dppe), 2.38 (2H, sept, $^3J_{H\text{-}H}$ = 7.0 Hz, $CH(CH_3)_2$), 1.77 (6H, s, $CH_3$), 0.85 (12H, d, $^3J_{H\text{-}H}$ = 7.0 Hz, $CH(CH_3)_2$). $^{31}P\{^1H\}$ NMR (161.976 MHz, $CDCl_3$, 293 K): $\delta$ 24.76 (s, dppe-*P*). FT-IR ($cm^{-1}$): $\nu$ 3031 (w), 2958 (w), 1433 (m), 1124 (w), 1099 (m), 874 (m), 721 (s), 693 (s), 515 (s), 484 (s), 464 (m). UV-Vis $\lambda_{max}$ (dichloromethane): 226 nm (major, $\epsilon$ = 49238 $M^{-1}cm^{-1}$), 245 nm ($\epsilon$ = 34229 $M^{-1}cm^{-1}$), 370 (minor, $\epsilon$ = 3619 $M^{-1}cm^{-1}$). ESI-TOF-MS (ACN): m/z calcd. for $[M + Na]^+$, 1033.0288; expt., 1033.0582 (49 %). m/z calcd. for $[M - Cl]^+$, 975.0706; expt., 975.0925 (6 %).

$[(p\text{-cym})RuCl_2]_2(\mu\text{-dppa})$ (**3**)

50 mg of $[(p\text{-cym})RuCl_2]_2$ (0.327 mmol, 1 eq.) and 32 mg (0.327 mmol, 1 eq.) used, 57 mg (0.179 mmol, 70 %) of red powder obtained, air stable. The NMR spectroscopic characterisation matches with the literature [2]. $^1H$ NMR (400.130 MHz, $CDCl_3$, 293 K): $\delta$ 8.03 – 7.94 (8 H, m, P*Ph*), 7.44 – 7.34 (12 H, m, P*Ph*), 5.37 (4 H, d, $^2J_{H\text{-}H}$ = 6.1 Hz, $C^{2,6\text{ or }3,5}H$, *p*-cym), 5.34 (4 H, d, $^2J_{H\text{-}H}$ = 6.1 Hz, $C^{2,6\text{ or }3,5}H$, *p*-cym), 2.45 (2H, sept, $^3J_{H\text{-}H}$ = 6.9 Hz, $CH(CH_3)_2$), 1.73 (6H, s, $CH_3$), 0.96 (12H, d, $^3J_{H\text{-}H}$ = 6.9 Hz, $CH(CH_3)_2$). $^{31}P\{^1H\}$ NMR (161.976 MHz, $CDCl_3$, 293 K): $\delta$ 10.64 (s, dppa-*P*). FT-IR ($cm^{-1}$): $\nu$ 3048 (w), 2963 (w), 1436 (w), 1263 (w), 1097 (w), 826 (m), 732 (m), 701 (m), 687 (m), 520 (s), 481 (m), 473 (m), 424 (m). UV-Vis $\lambda_{max}$ (dichloromethane): 225 nm (major, $\epsilon$ = 49810 $M^{-1}cm^{-1}$), 383 nm (minor, $\epsilon$ = 3251 $M^{-1}cm^{-1}$). ESI-TOF-MS (ACN): m/z calcd. for $[M + Na]^+$, 1028.9974; expt., 1029.0083 (7 %). m/z calcd. for $[M - Cl]^+$, 971.0392; expt., 971.0628 (3 %).

2. $^{1}$H NMR spectra

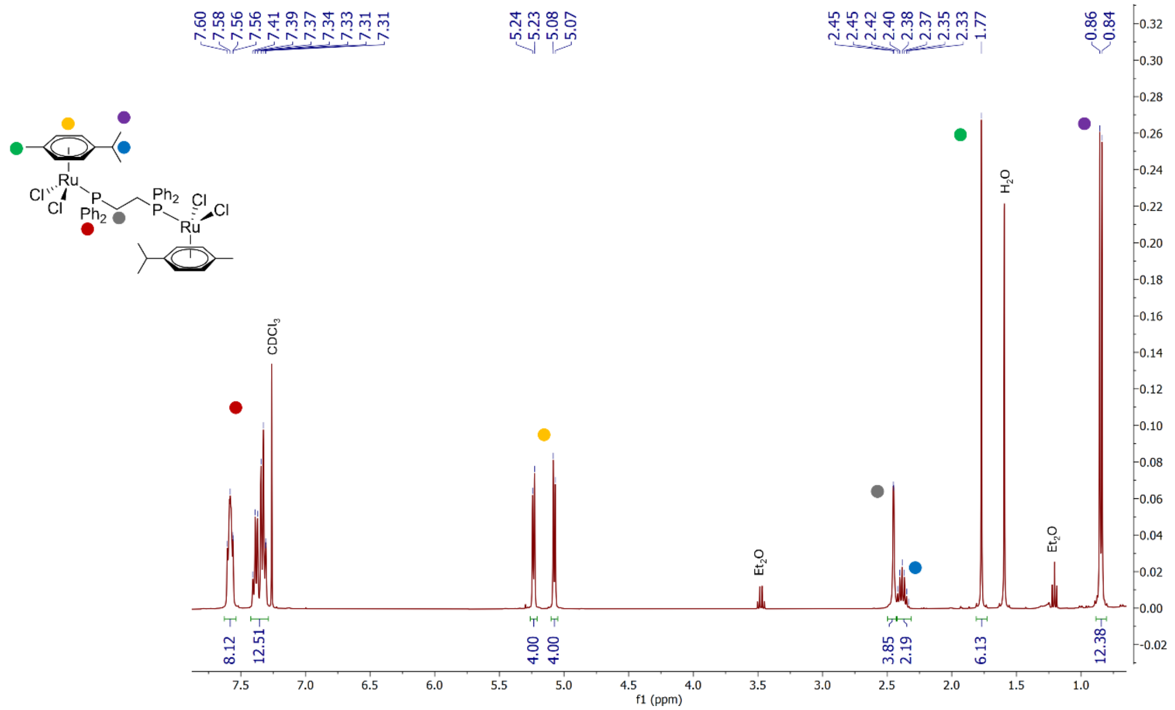


**Figure S1**. $^{1}$H NMR spectrum of **1** in $CDCl_3$ at 298 K

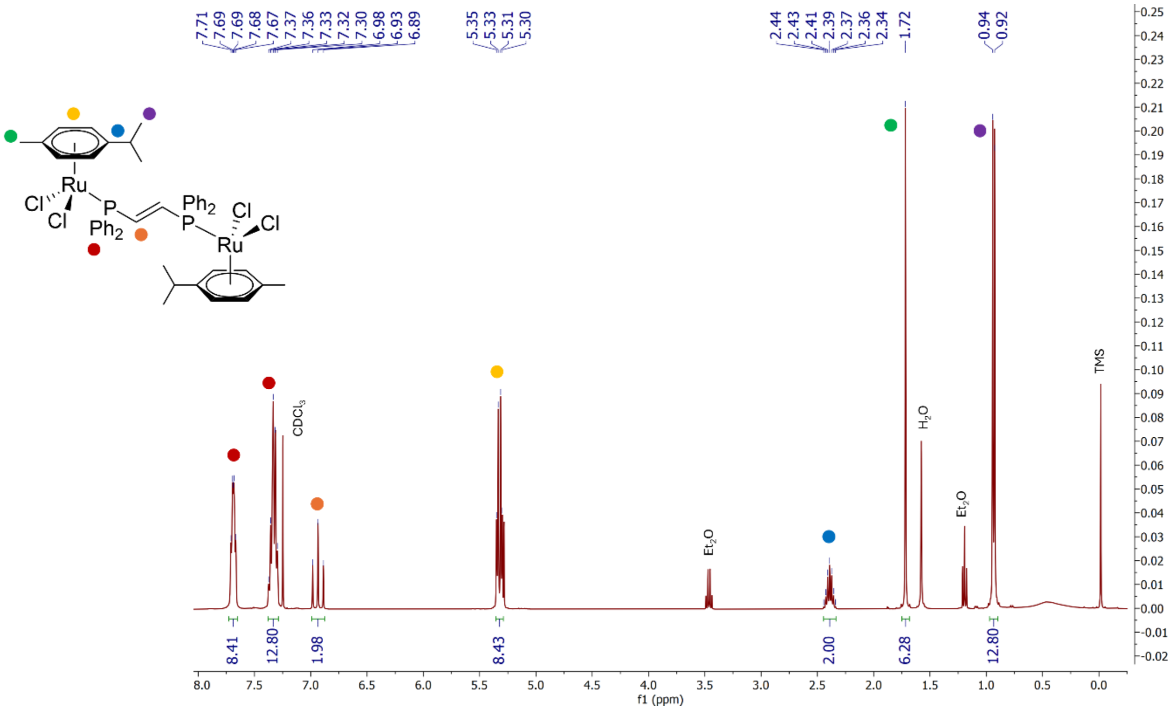


**Figure S2**. $^{1}$H NMR spectrum of **2** in $CDCl_3$ at 298 K

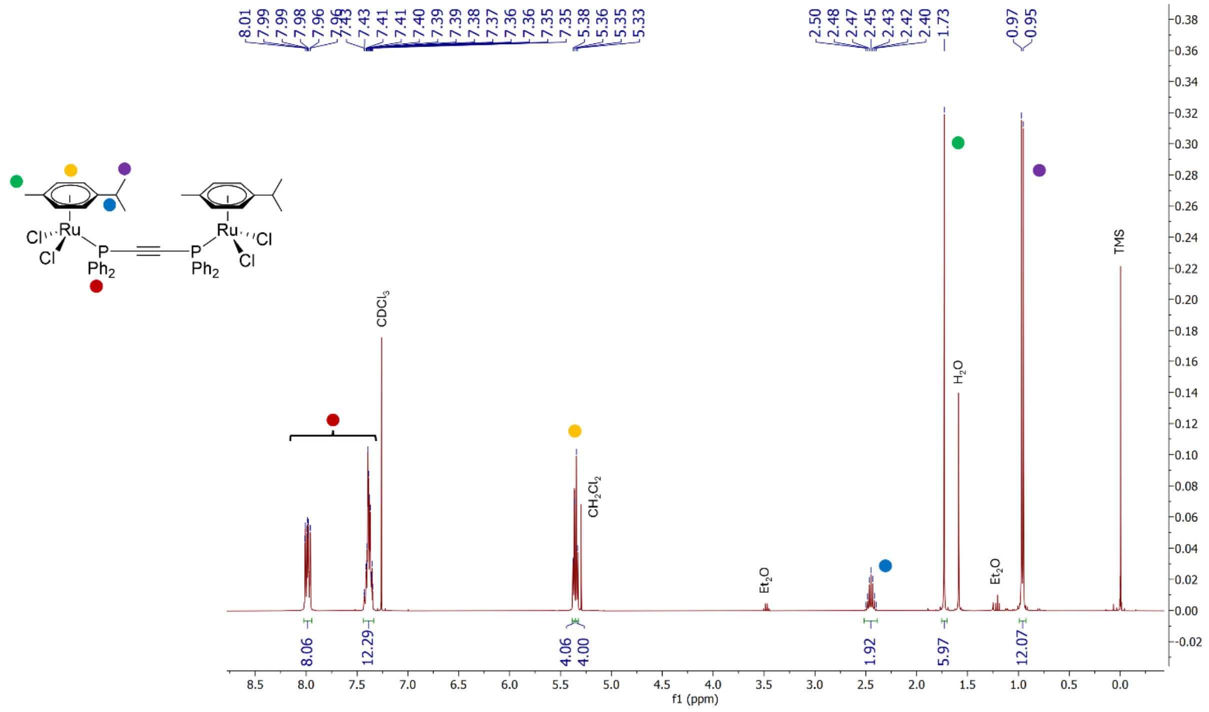


**Figure S3**. ¹H NMR spectrum of **3** in $CDCl_3$ at 298 K

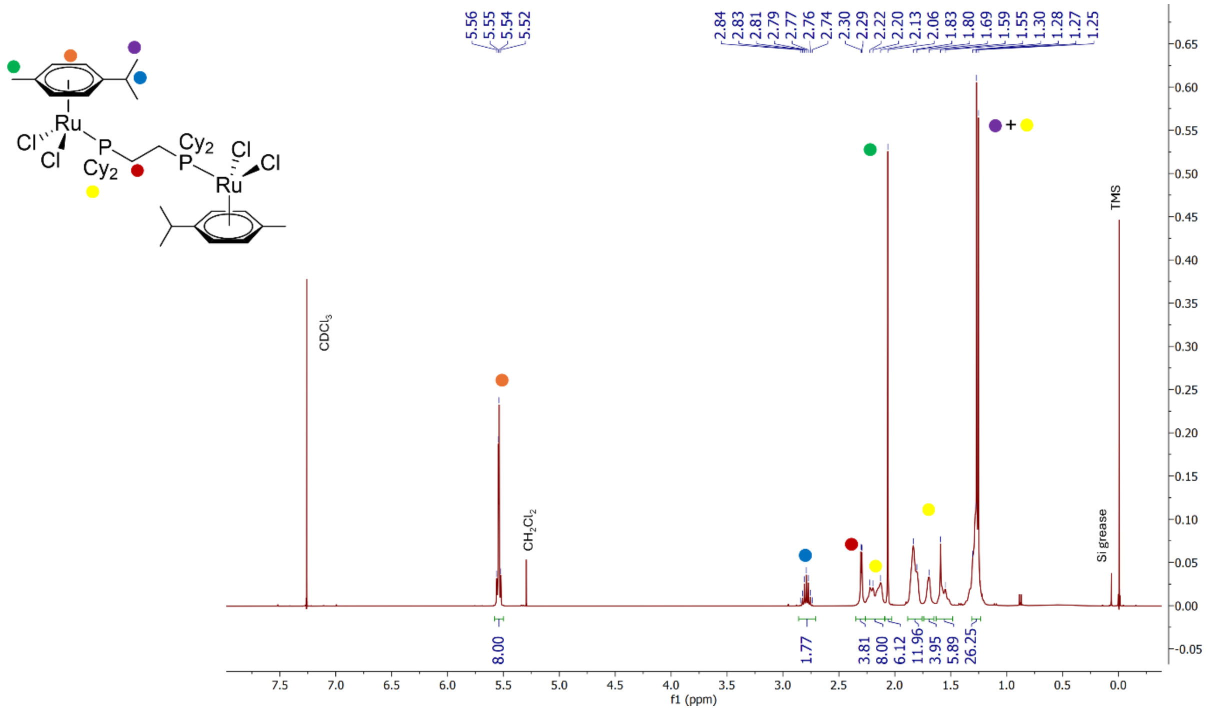


**Figure S4**. ¹H NMR spectrum of **4** in $CDCl_3$ at 298 K

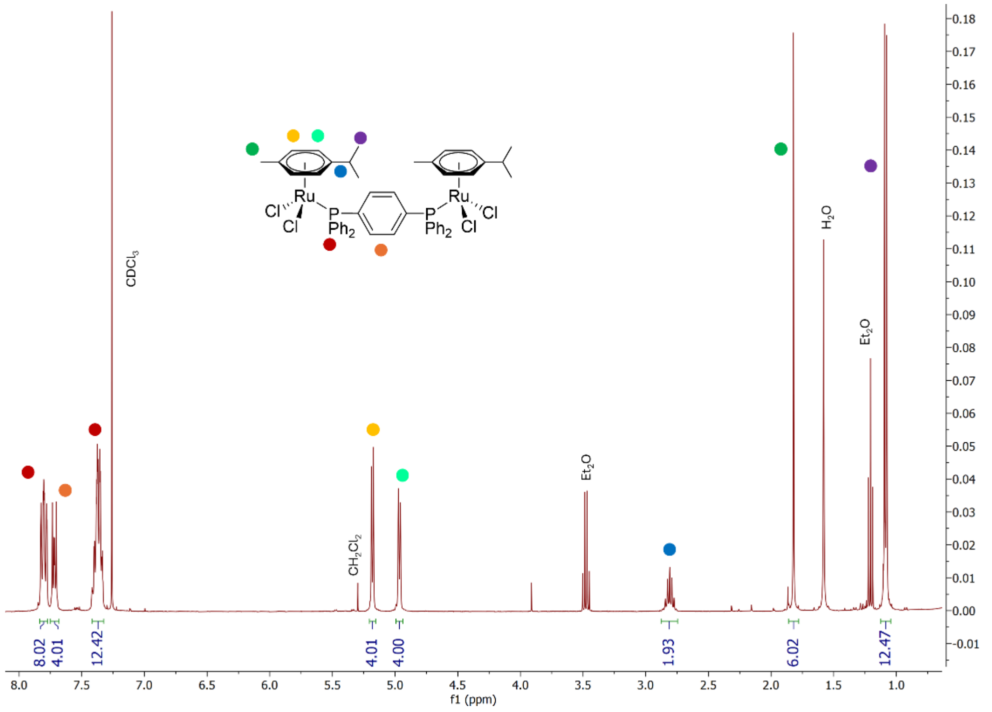


**Figure S5**. $^{1}$H NMR spectrum of **5** in $CDCl_3$ at 298 K. The singlet at 3.91 ppm corresponds to a minor unidentified impurity.

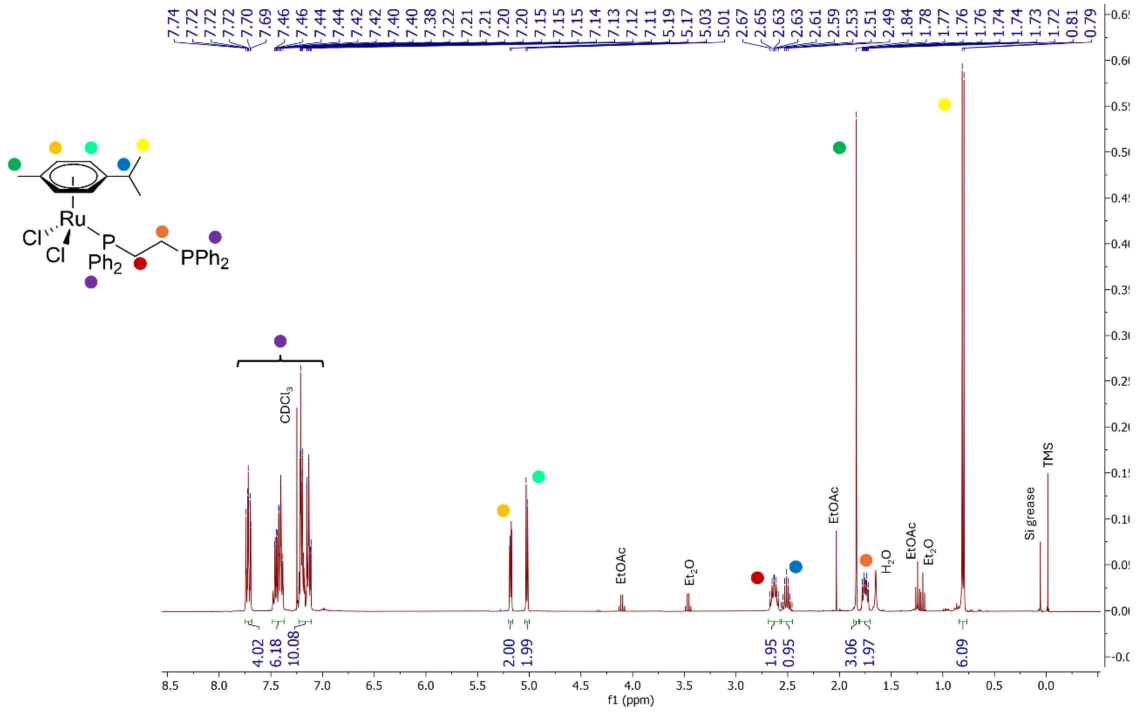


**Figure S6**. $^{1}$H NMR spectrum of **6** in $CDCl_3$ at 298 K

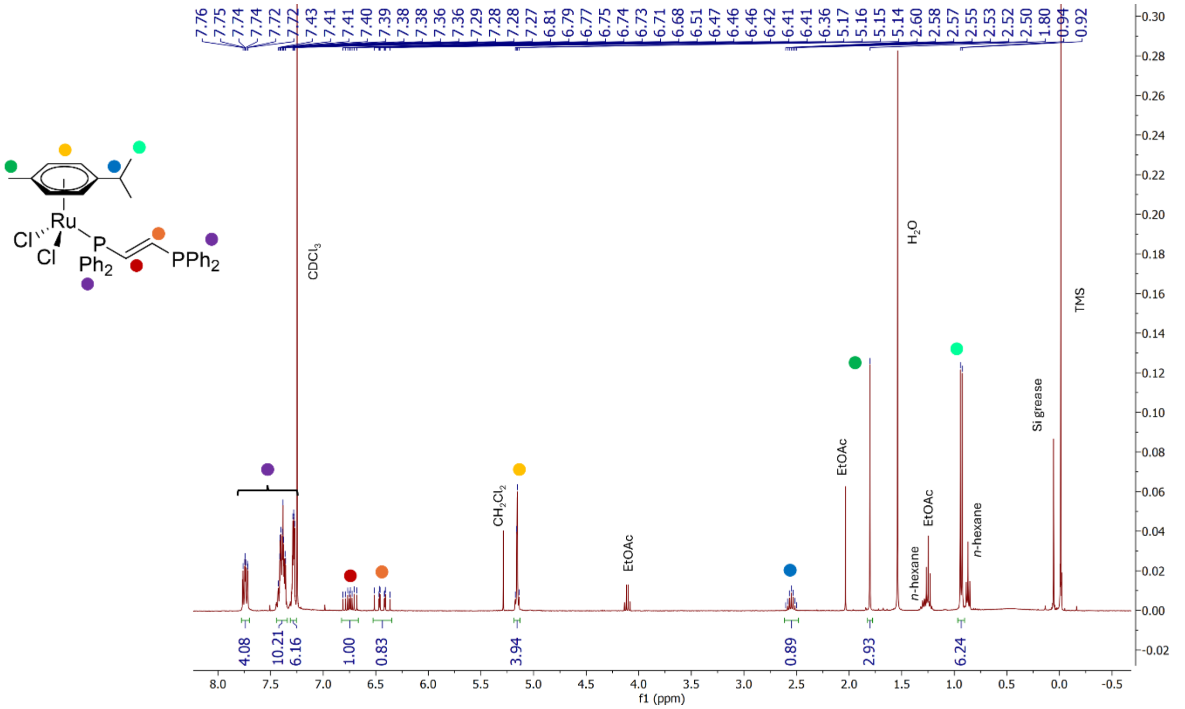


**Figure S7**. $^{1}H$ NMR spectrum of **7** in $CDCl_3$ at 298 K

3. $^{13}C\{^{1}H\}$ NMR spectra

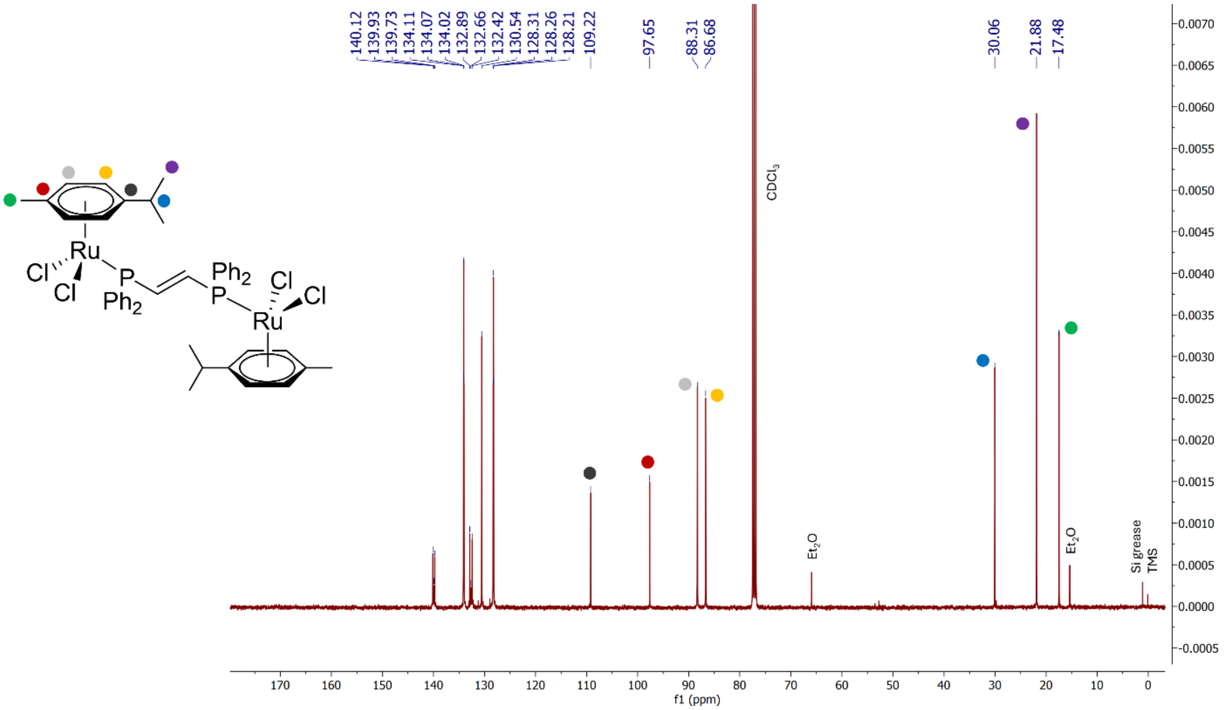


**Figure S8**. $^{13}C\{^{1}H\}$ NMR spectrum of **2** in $CDCl_3$ at 298 K

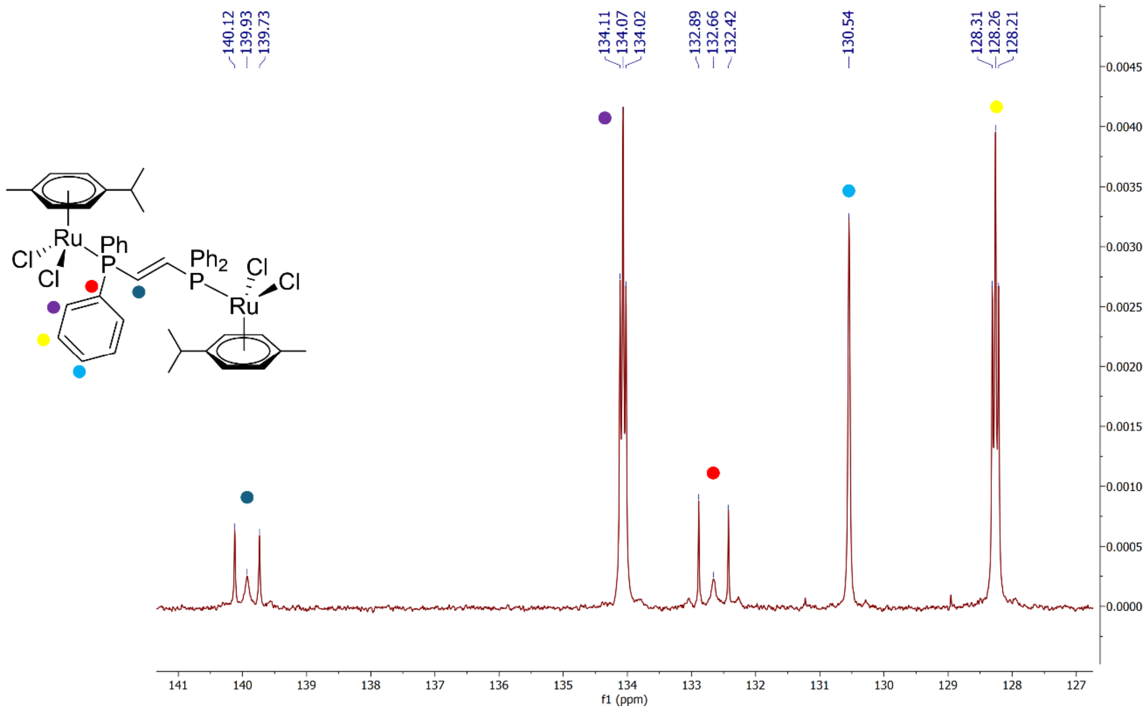


**Figure S9**. $^{13}C\{^1H\}$ NMR spectrum of **2** in $CDCl_3$ at 298 K (zoomed in)

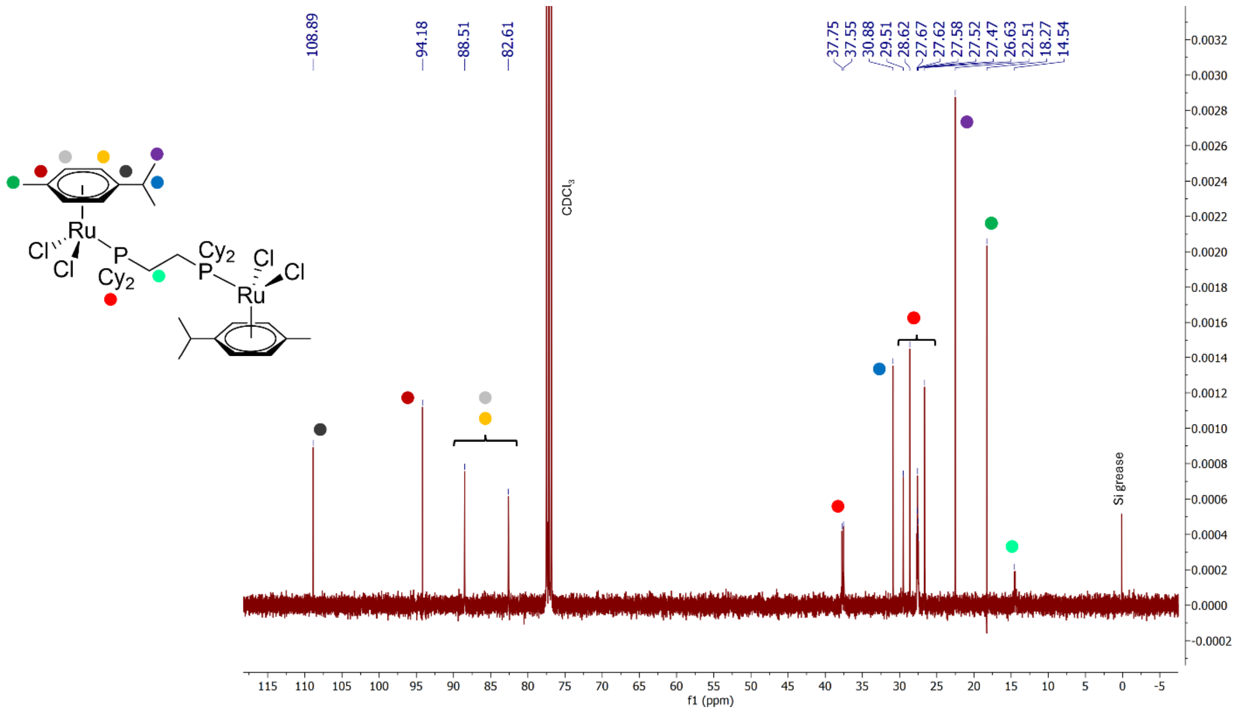


**Figure S10**. $^{13}C\{^1H\}$ NMR spectrum of **4** in $CDCl_3$ at 298 K

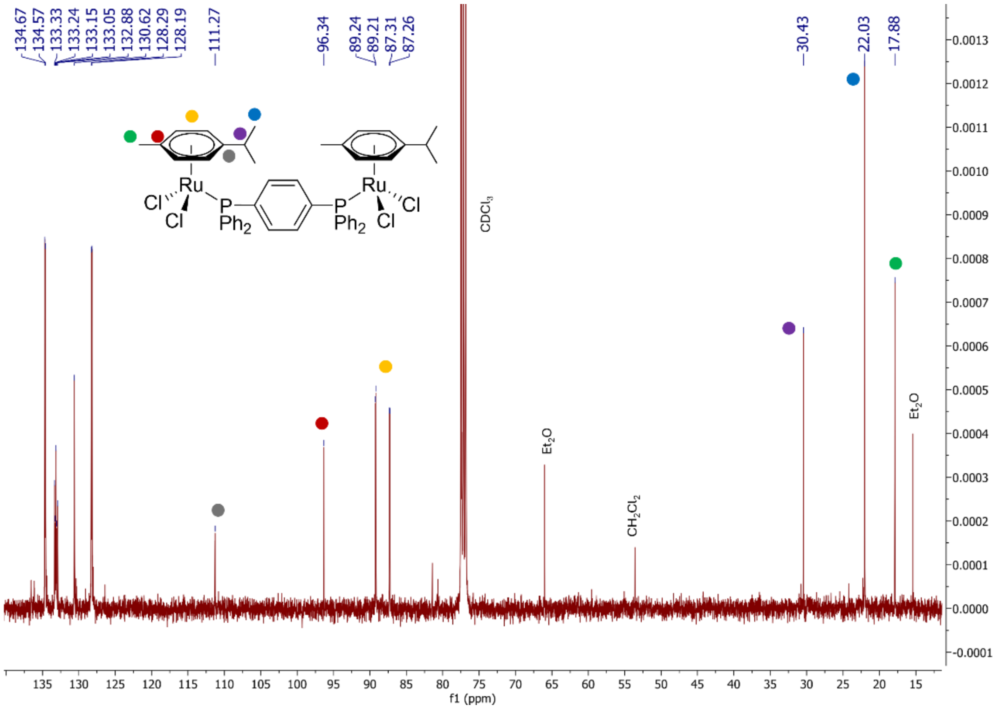


**Figure S11**. $^{13}C\{^{1}H\}$ NMR spectrum of **5** in $CDCl_3$ at 298 K. The signals at 81.4 and 80.7 ppm correspond to minor unidentified impurities.

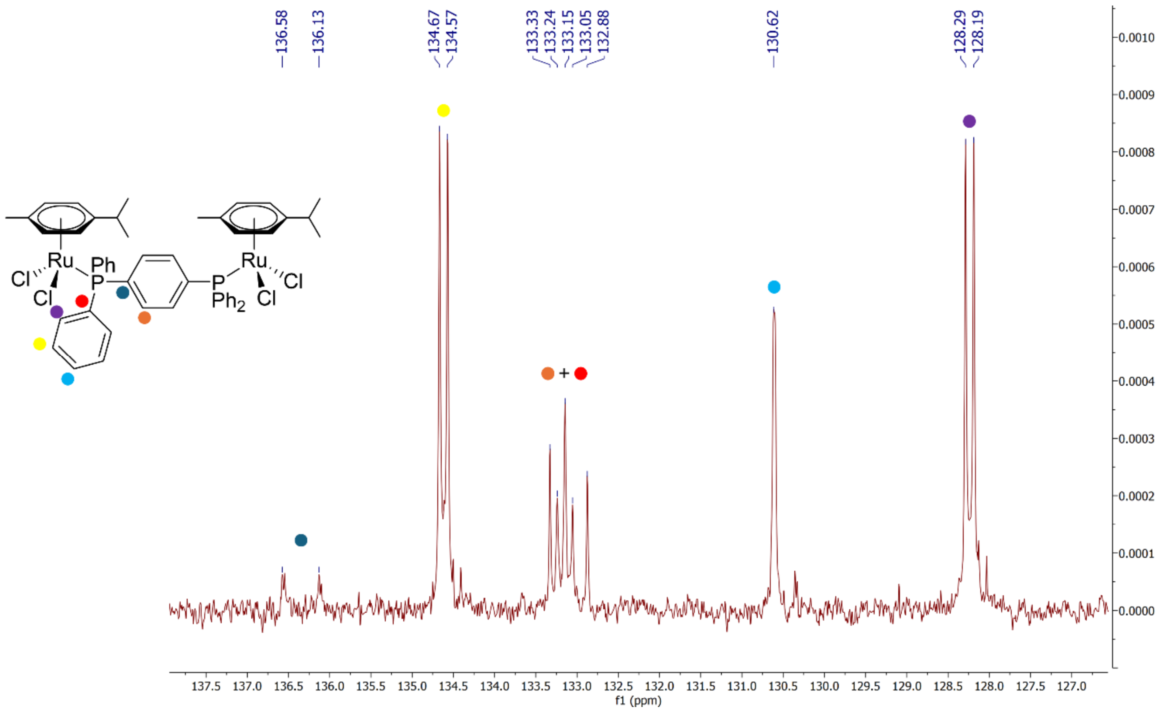


**Figure S12**. $^{13}C\{^{1}H\}$ NMR spectrum of **5** in $CDCl_3$ at 298 K (zoomed in)

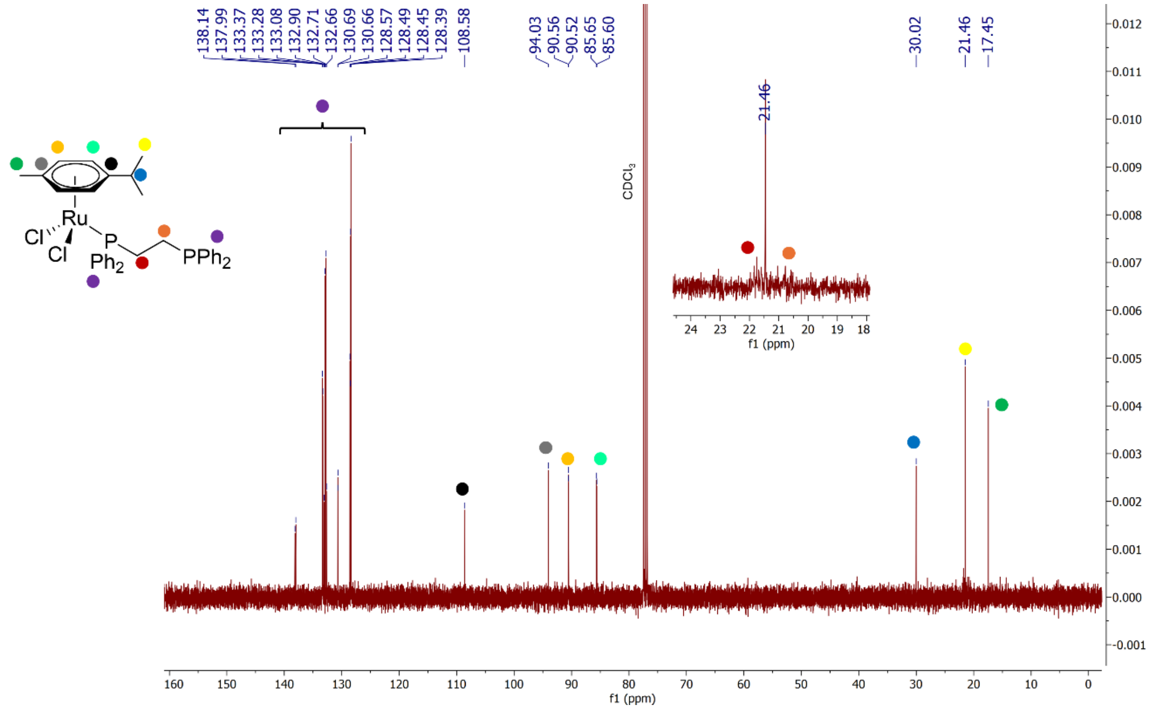


**Figure S13**. $^{13}C\{^1H\}$ NMR spectrum of **6** in $CDCl_3$ at 298 K

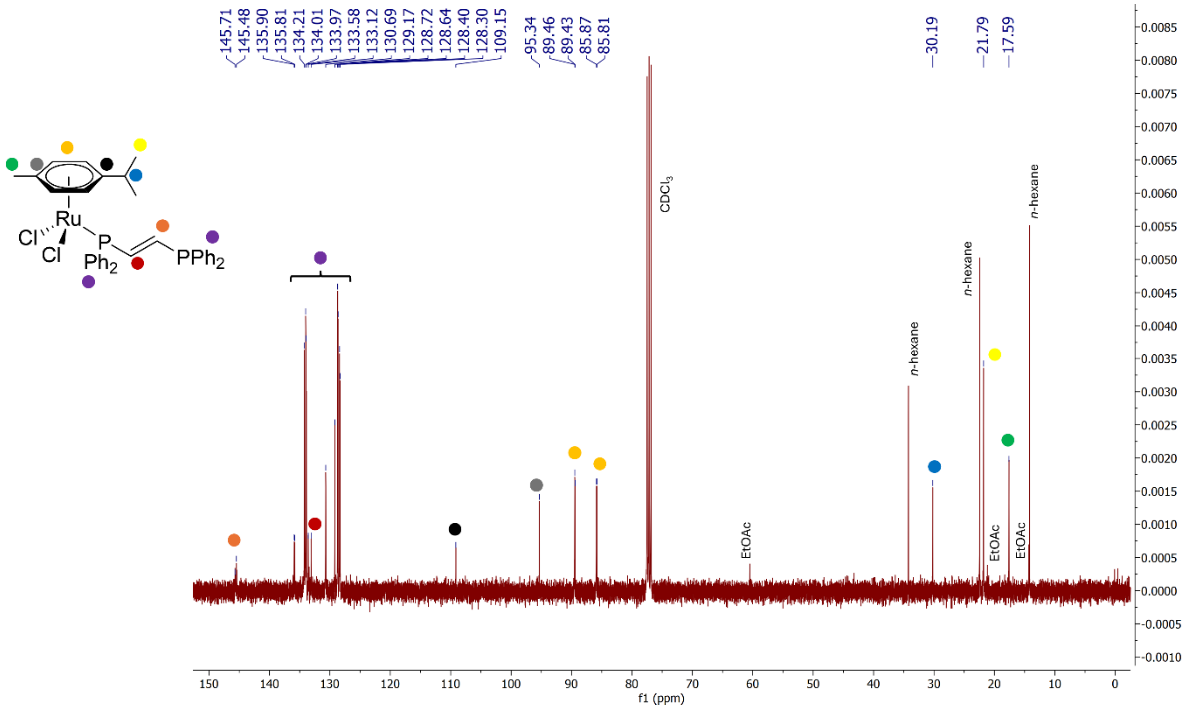


**Figure S14**. $^{13}C\{^1H\}$ NMR spectrum of **7** in $CDCl_3$ at 298 K

4. $^{31}P\{^{1}H\}$ NMR spectra

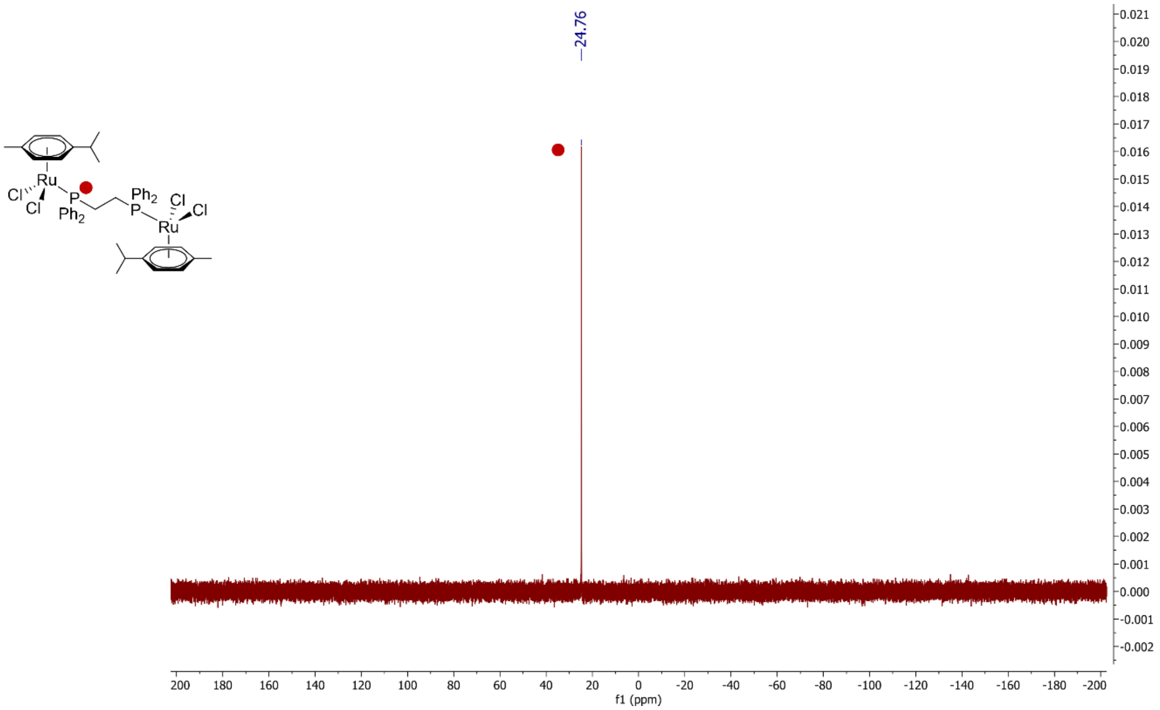


**Figure S15**. $^{31}P\{^{1}H\}$ NMR spectrum of **1** in $CDCl_3$ at 298 K

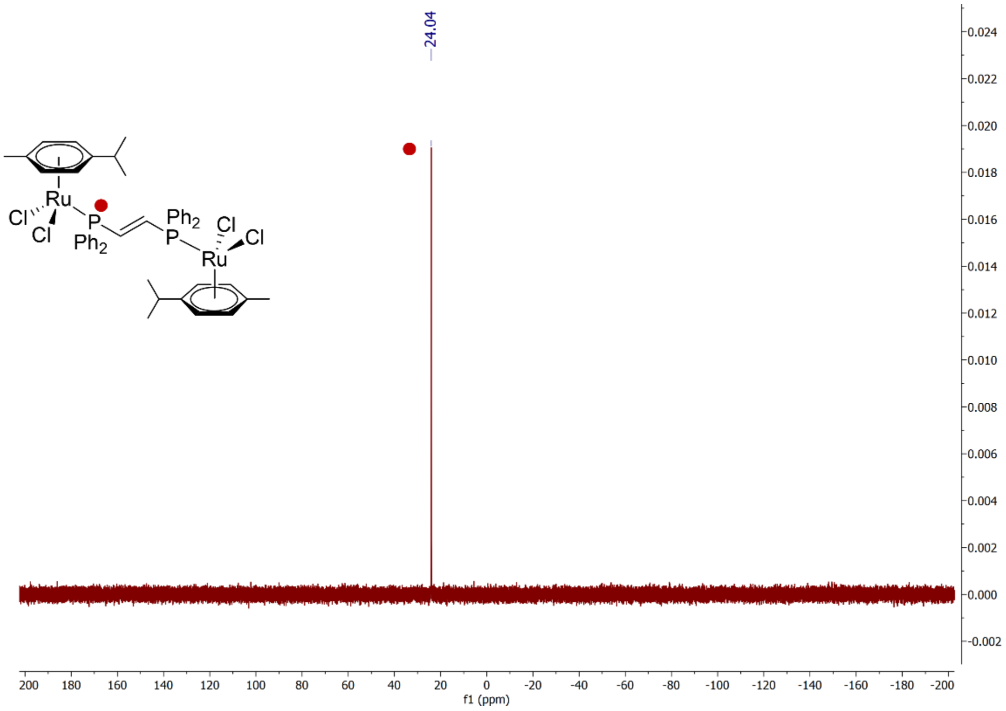


**Figure S16**. $^{31}P\{^{1}H\}$ NMR spectrum of **2** in $CDCl_3$ at 298 K

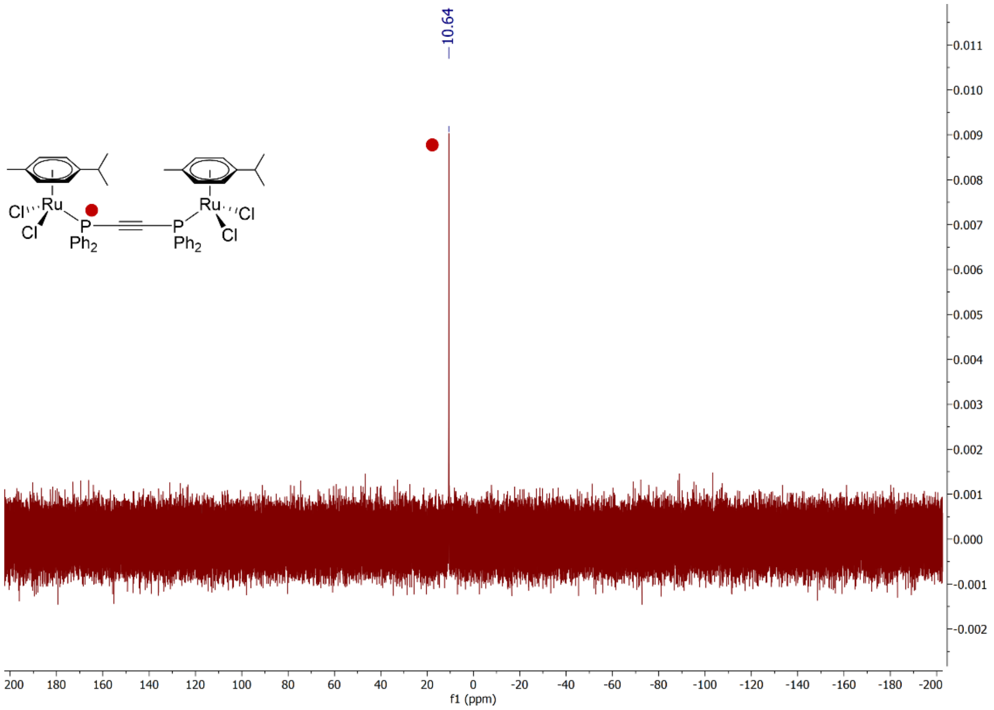


**Figure S17**. $^{31}P\{^{1}H\}$ NMR spectrum of **3** in $CDCl_3$ at 298 K

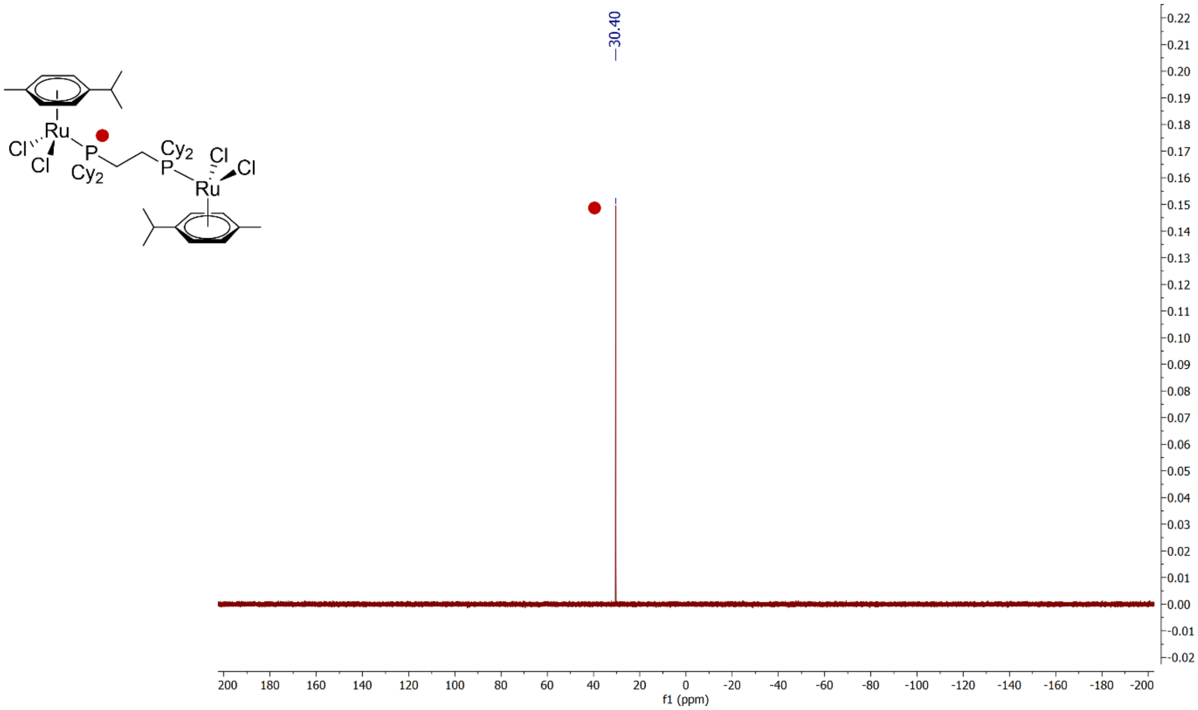


**Figure S18**. $^{31}P\{^{1}H\}$ NMR spectrum of **4** in $CDCl_3$ at 298 K

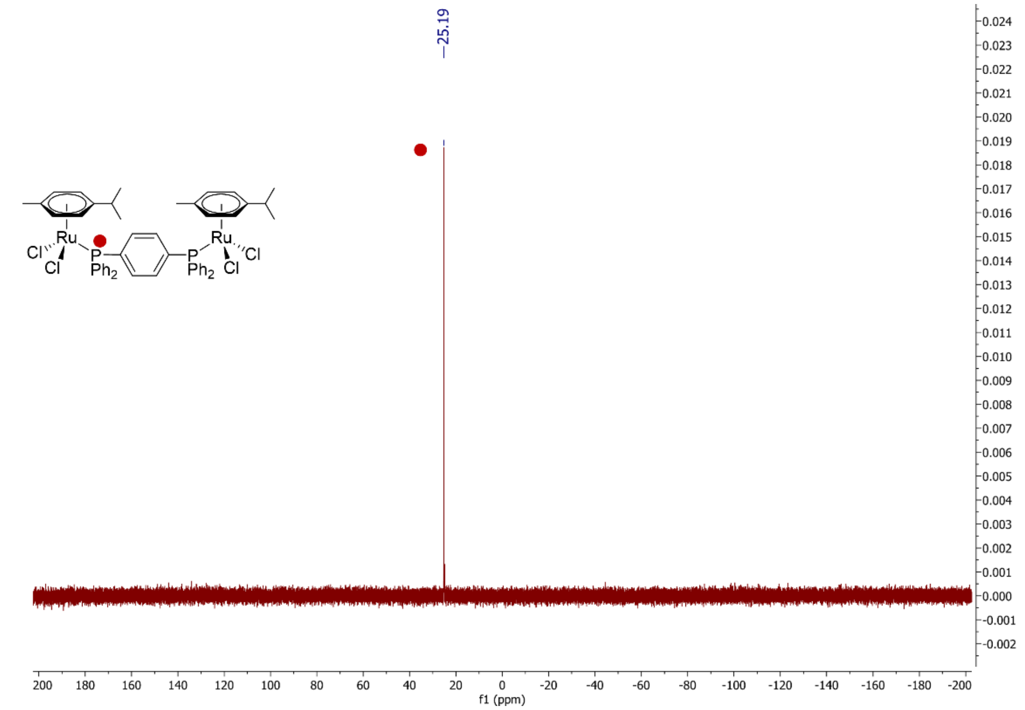


**Figure S19**. $^{31}P\{^{1}H\}$ NMR spectrum of **5** in $CDCl_3$ at 298 K

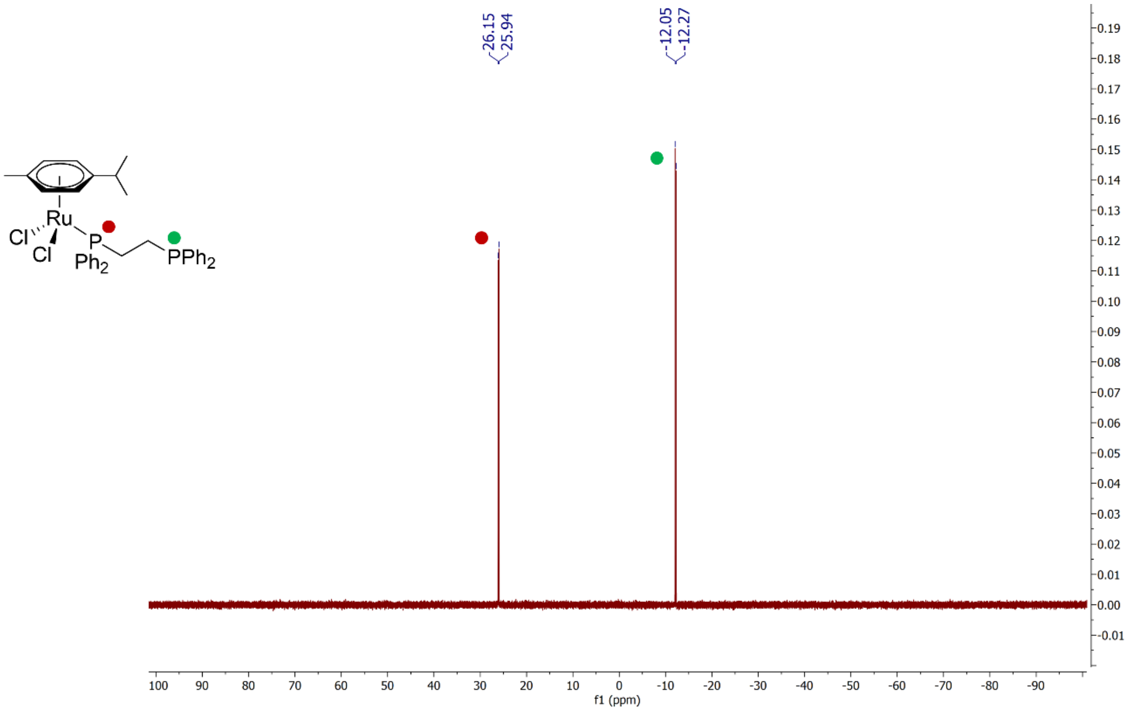


**Figure S20**. $^{31}P\{^{1}H\}$ NMR spectrum of **6** in $CDCl_3$ at 298 K

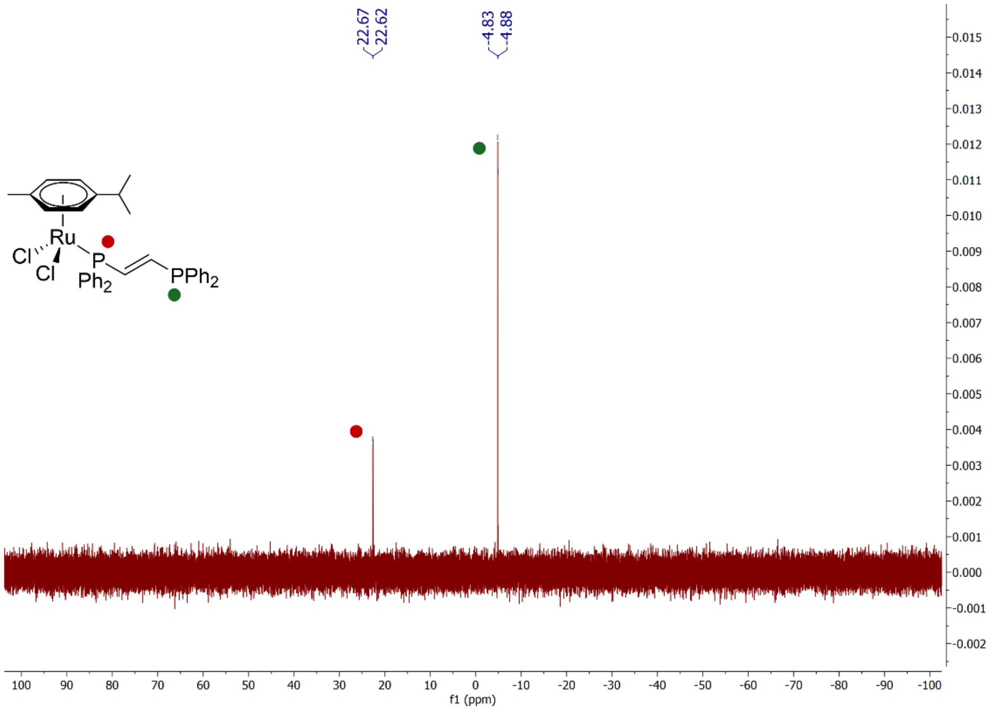


**Figure S21**. $^{31}P\{^{1}H\}$ NMR spectrum of **7** in $CDCl_3$ at 298 K

5. 2D NMR spectra

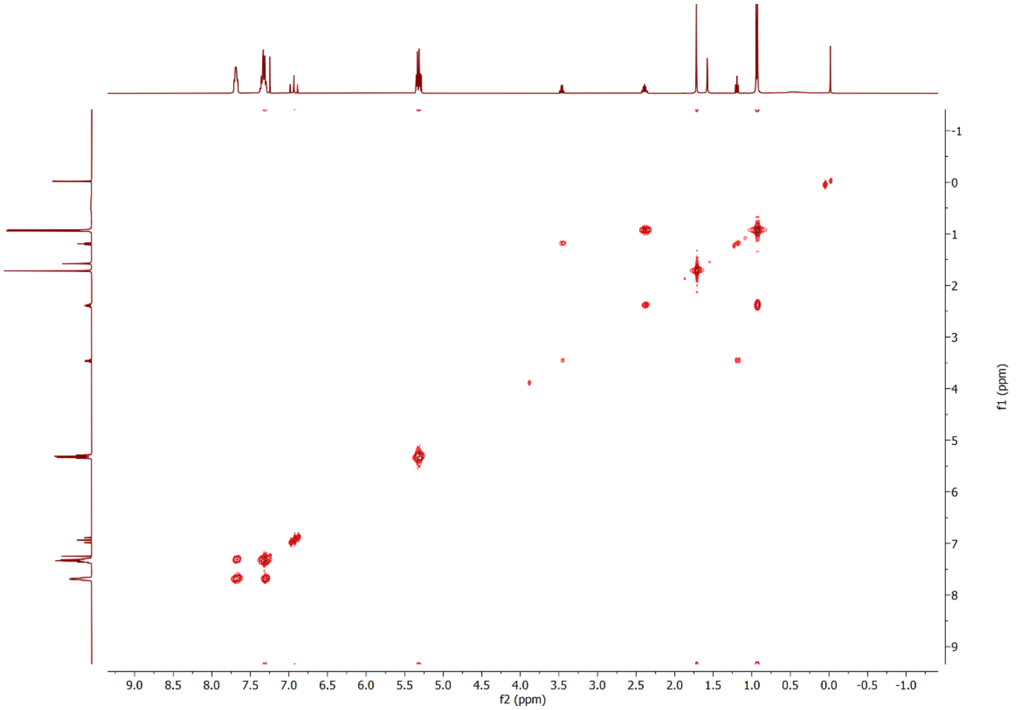


**Figure S22**. $^{1}H$-$^{1}H$ COSY NMR spectrum of **2** in $CDCl_3$ at 298 K

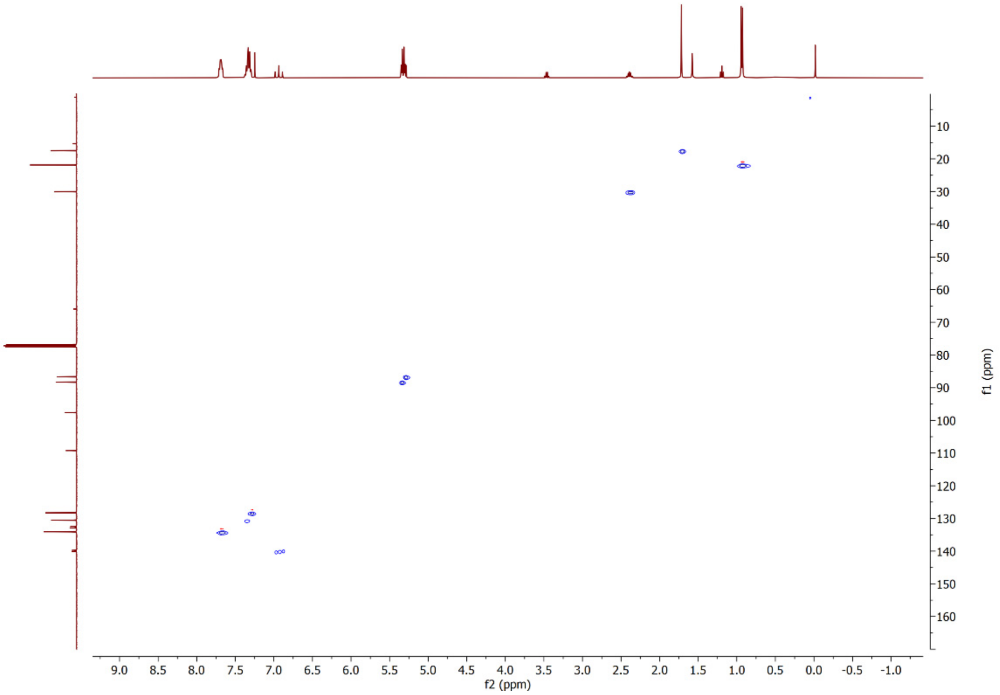


**Figure S23**. ¹H-¹³C HSQC NMR spectrum of **2** in $CDCl_3$ at 298 K

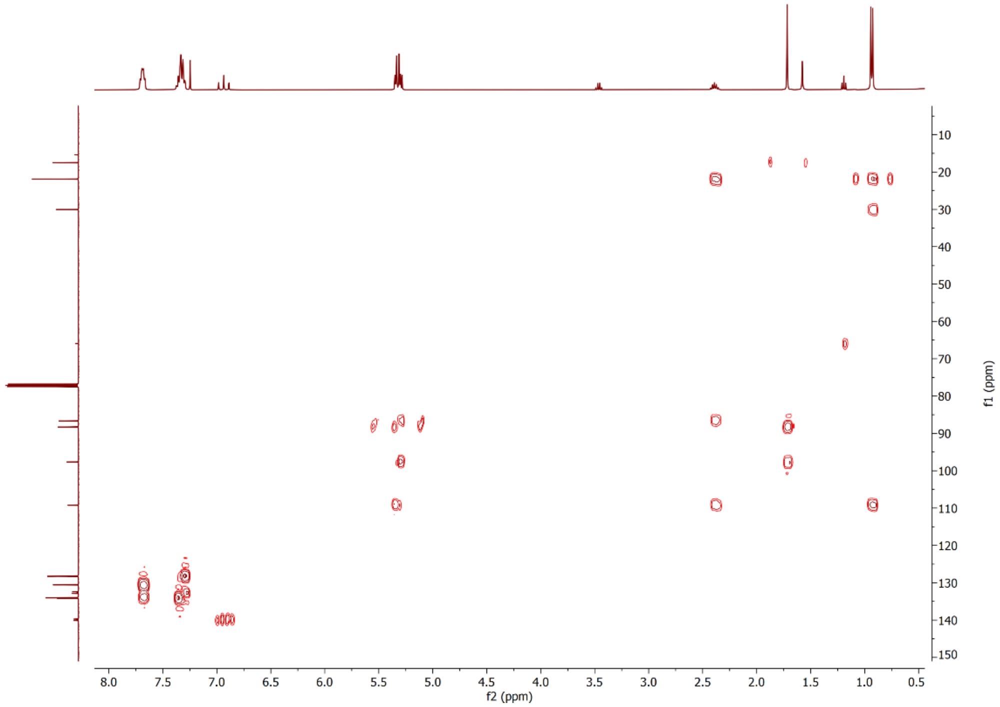


**Figure S24**. ¹H-¹³C HMBC NMR spectrum of **2** in $CDCl_3$ at 298 K

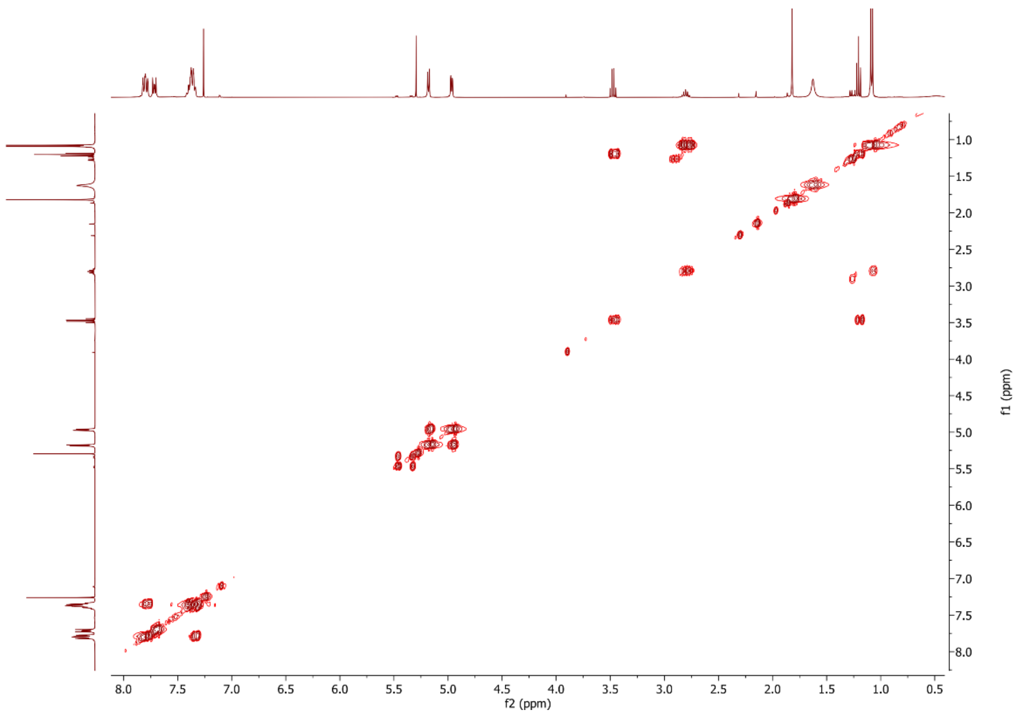


**Figure S25**. $^{1}$H-$^{1}$H COSY NMR spectrum of **5** in $CDCl_3$ at 298 K

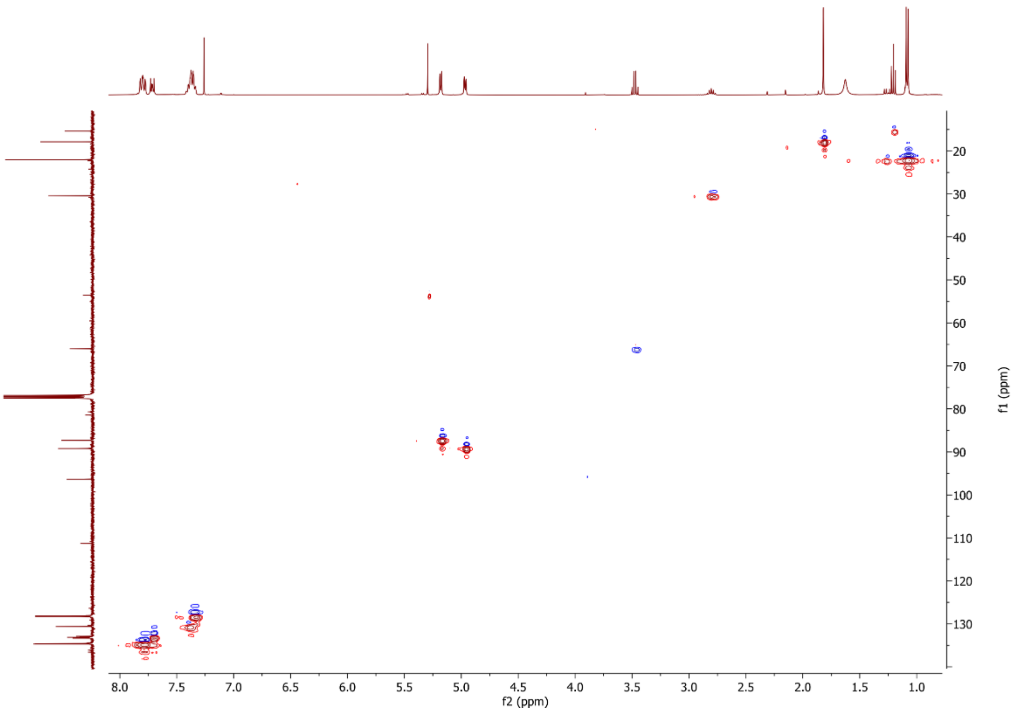


**Figure S26**. $^{1}$H-$^{13}$C HSQC NMR spectrum of **5** in $CDCl_3$ at 298 K

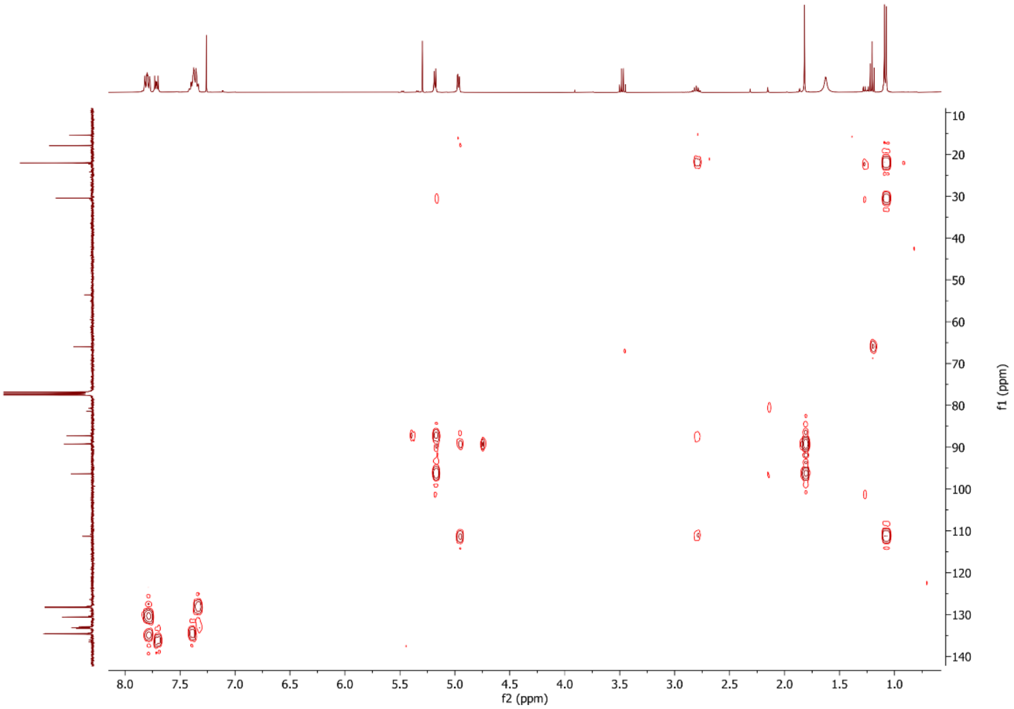


**Figure S27**. $^{1}H$-$^{13}C$ HMBC NMR spectrum of **5** in $CDCl_3$ at 298 K

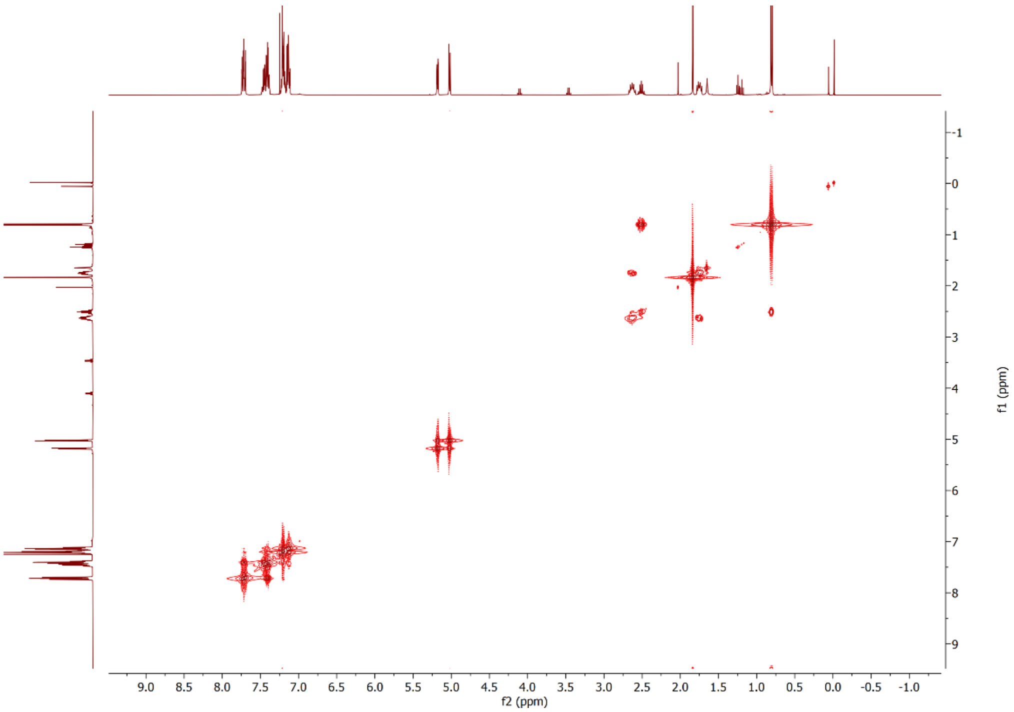


**Figure S28**. $^{1}H$-$^{1}H$ COSY NMR spectrum of **6** in $CDCl_3$ at 298 K

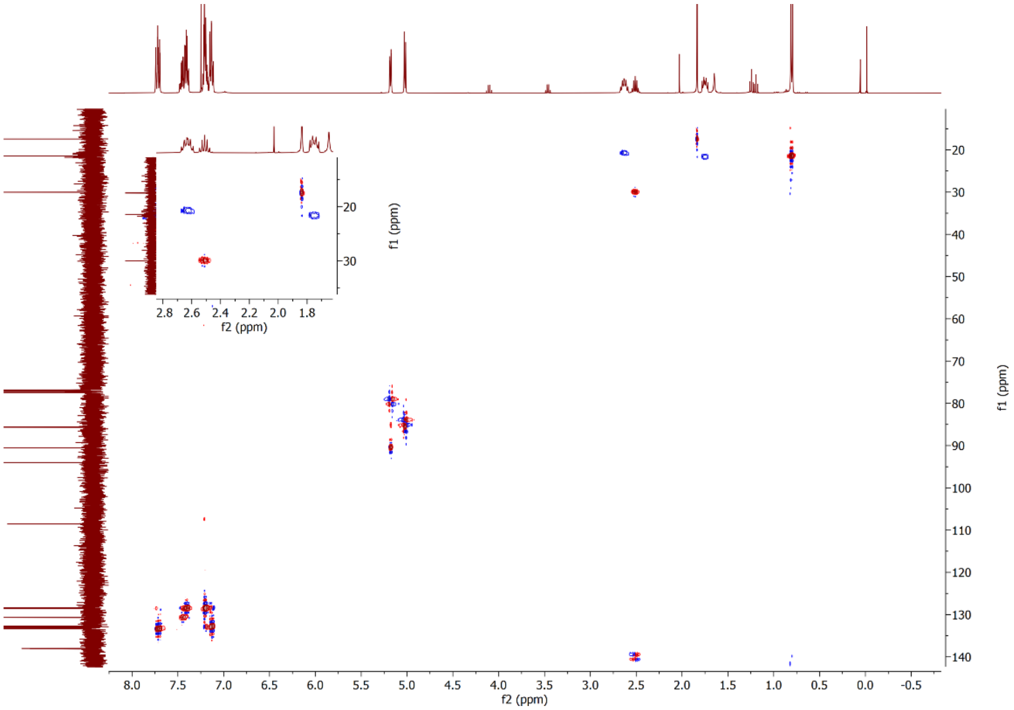


**Figure S29**. $^{1}H$-$^{13}C$ HSQC NMR spectrum of **6** in $CDCl_3$ at 298 K

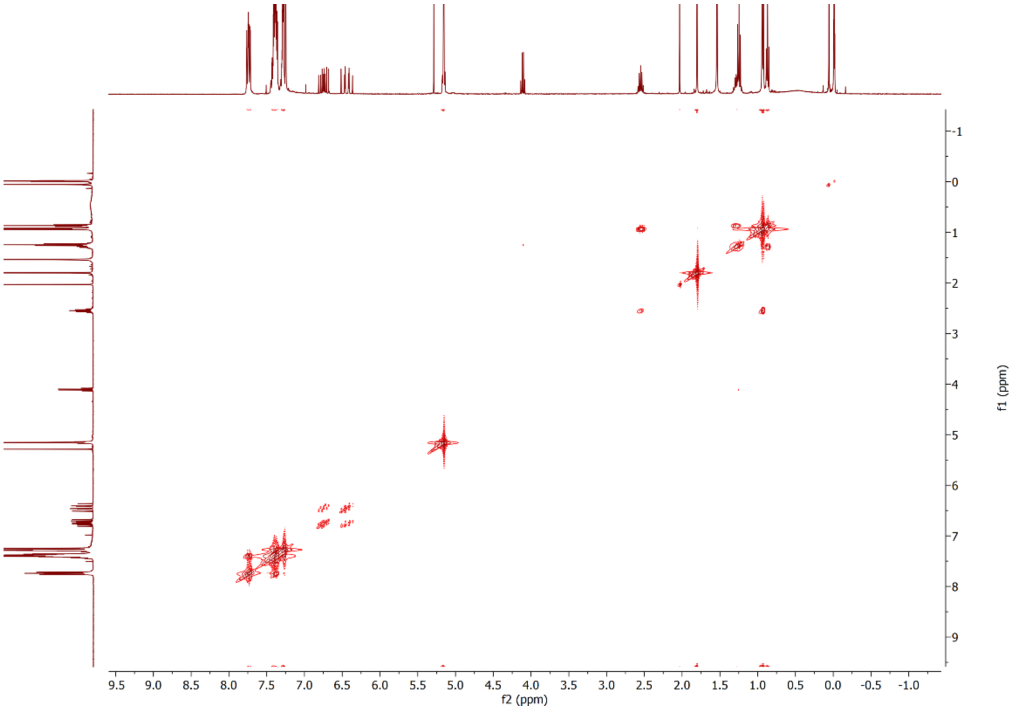


**Figure S30**. $^{1}H$-$^{1}H$ COSY NMR spectrum of **7** in $CDCl_3$ at 298 K

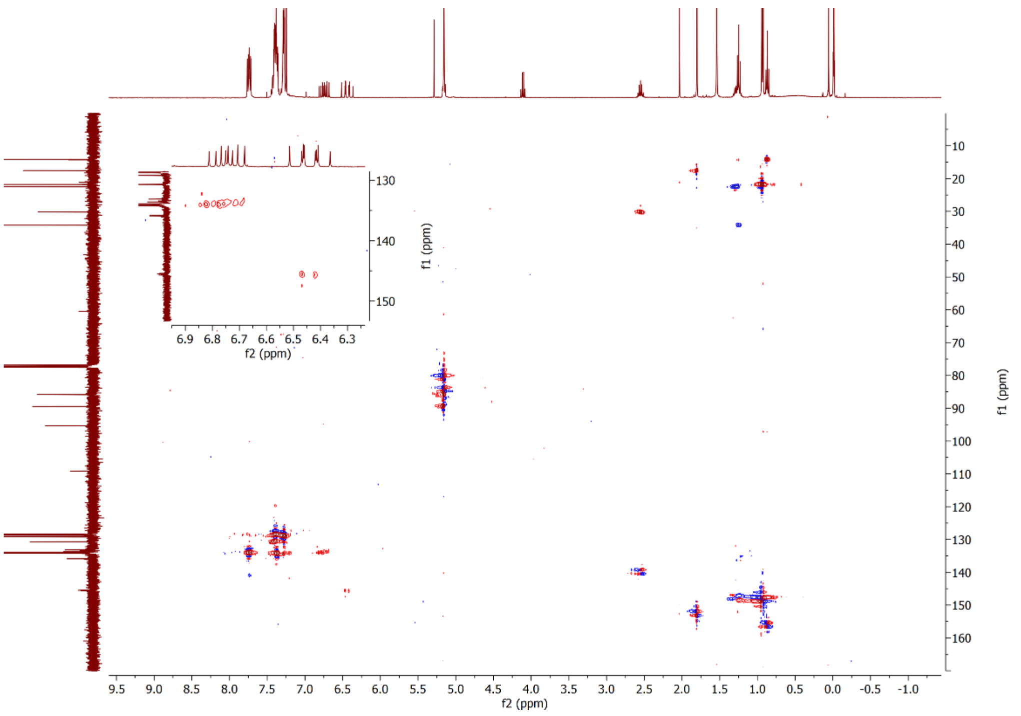


**Figure S31**. $^{1}H$-$^{13}C$ HSQC NMR spectrum of **7** in $CDCl_3$ at 298 K

6. Solution stability studies

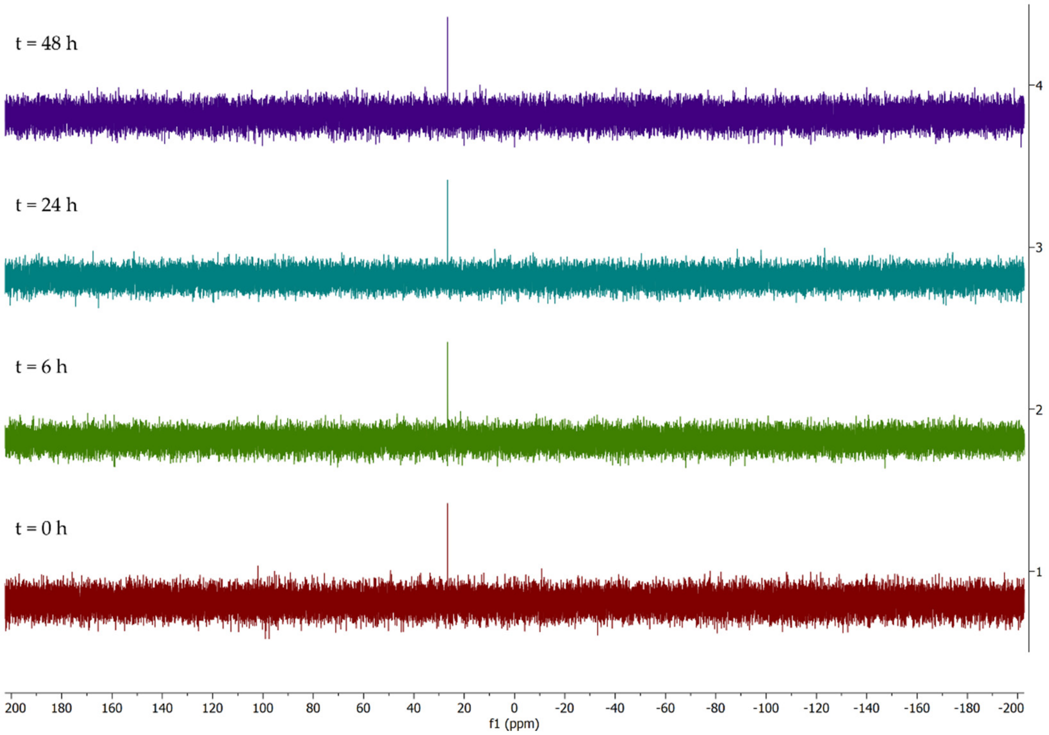


**Figure S32**. Time-dependant $^{31}P\{^{1}H\}$ NMR spectra of **1** in DMSO-$d_6$ at 298 K

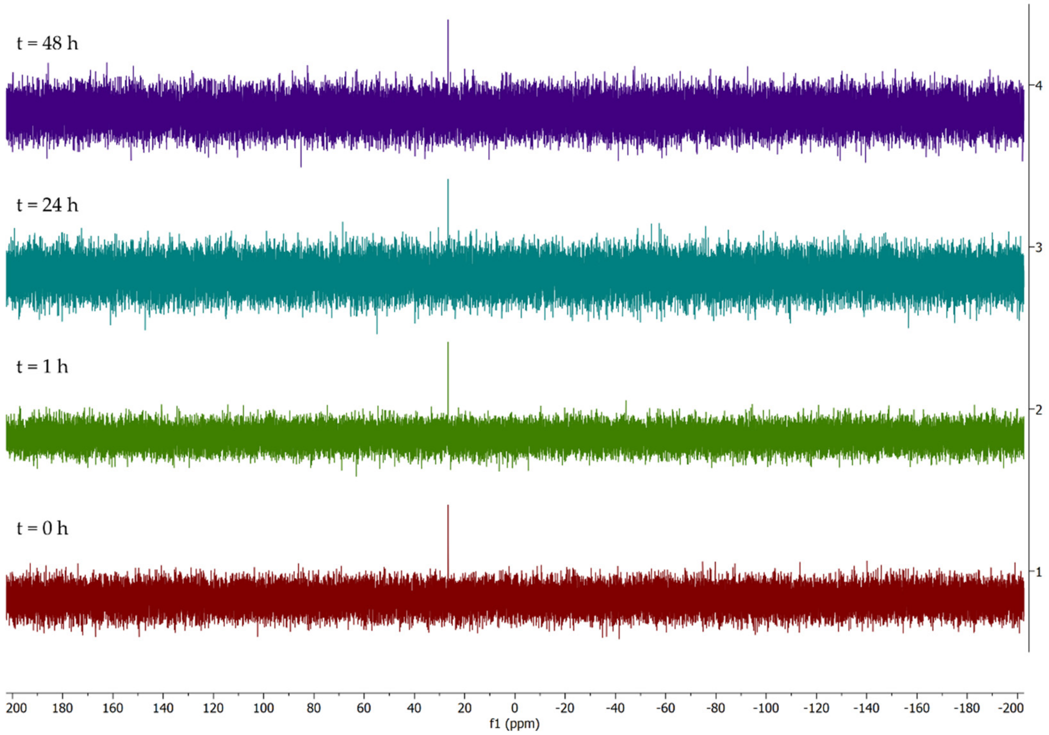


**Figure S33**. Time-dependant $^{31}P\{^{1}H\}$ NMR spectra of **1** in DMSO-$d_6$ at 298 K, after addition of one drop of PBS buffer solution in MilliQ water

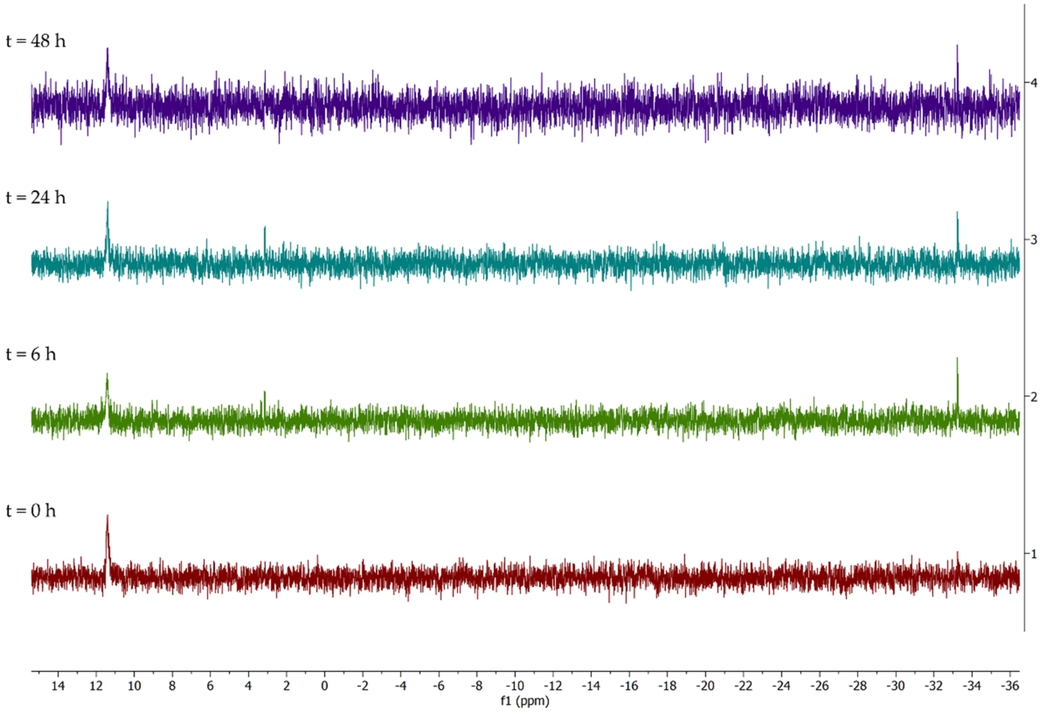


**Figure S34**. Time-dependant $^{31}P\{^{1}H\}$ NMR spectra of **3** in DMSO-$d_6$ at 298 K

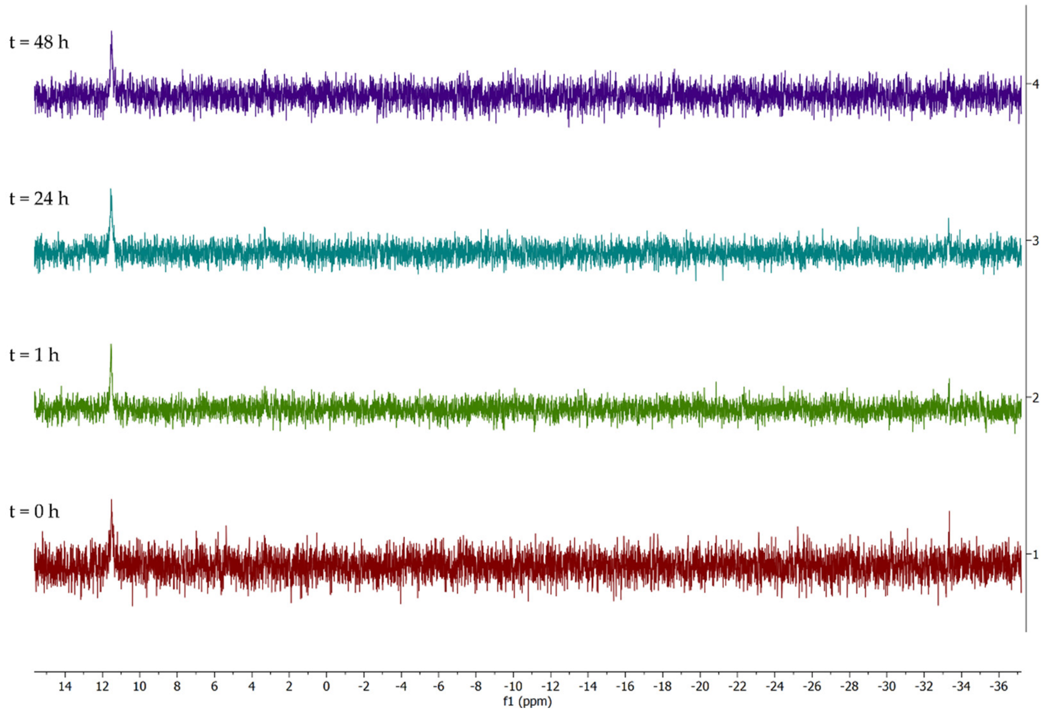


**Figure S35**. Time-dependant $^{31}P\{^{1}H\}$ NMR spectra of **3** in DMSO-$d_6$ at 298 K, after addition of one drop of PBS buffer solution in MilliQ water

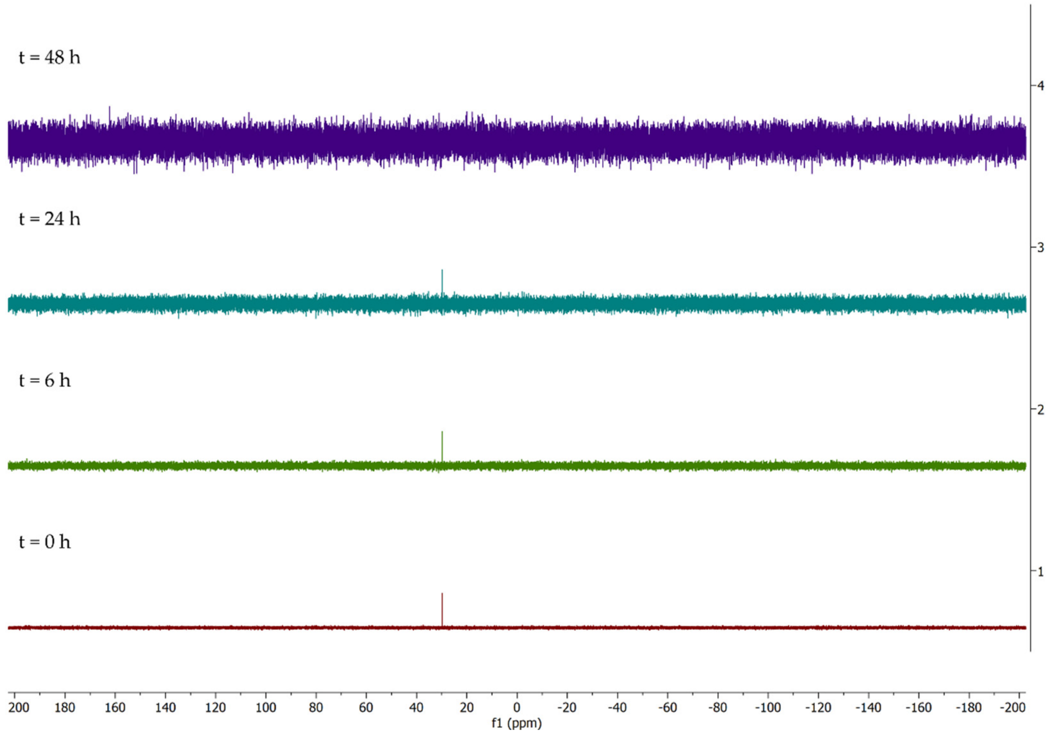


**Figure S36**. Time-dependant $^{31}P\{^{1}H\}$ NMR spectra of **4** in DMSO-$d_6$ at 298 K. It is worth noting that the complex seems to slowly crash out of solution over time, explaining the worse *s*/*n* ratio over time.

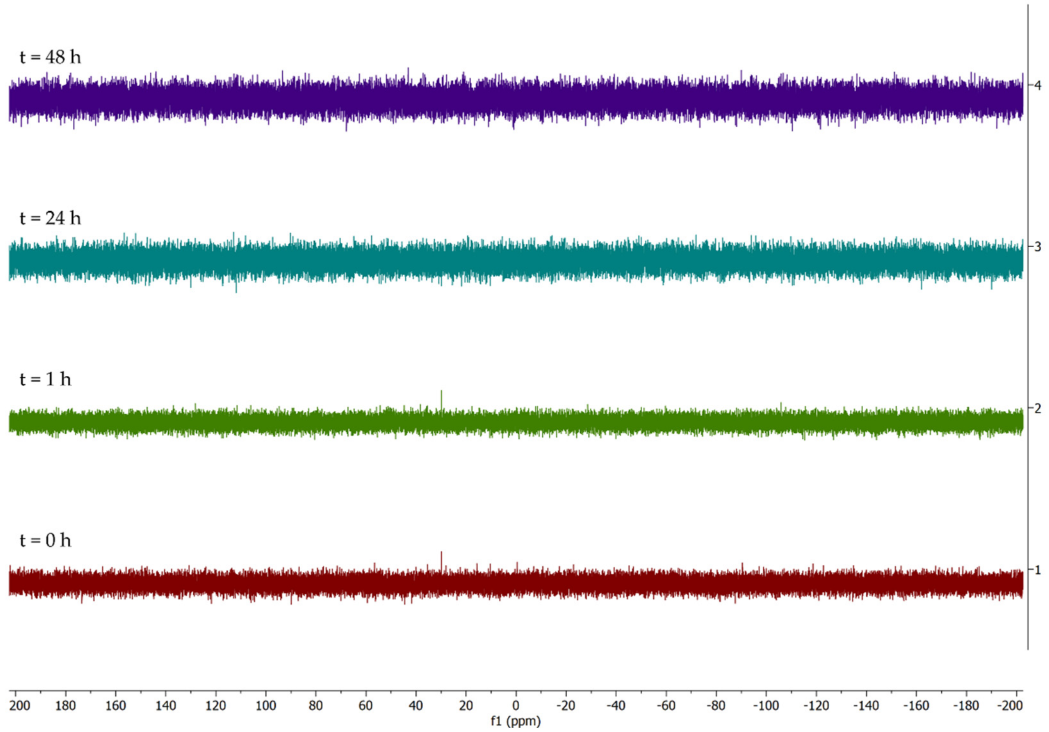


**Figure S37**. Time-dependant $^{31}P\{^{1}H\}$ NMR spectra of **4** in DMSO-$d_6$ at 298 K, after addition of one drop of PBS buffer solution in MilliQ water. The complex is fully crashed out of solution after 24 h, hence the disappearance of the signal.

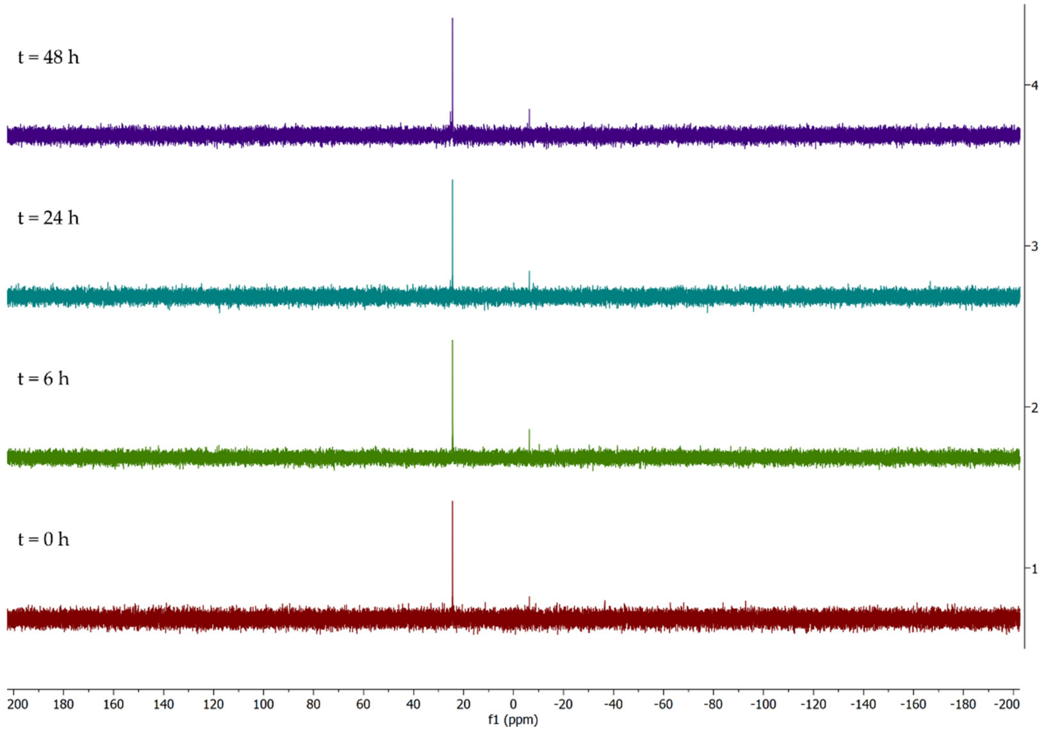


**Figure S38**. Time-dependant $^{31}P\{^{1}H\}$ NMR spectra of **5** in DMSO-$d_6$ at 298 K

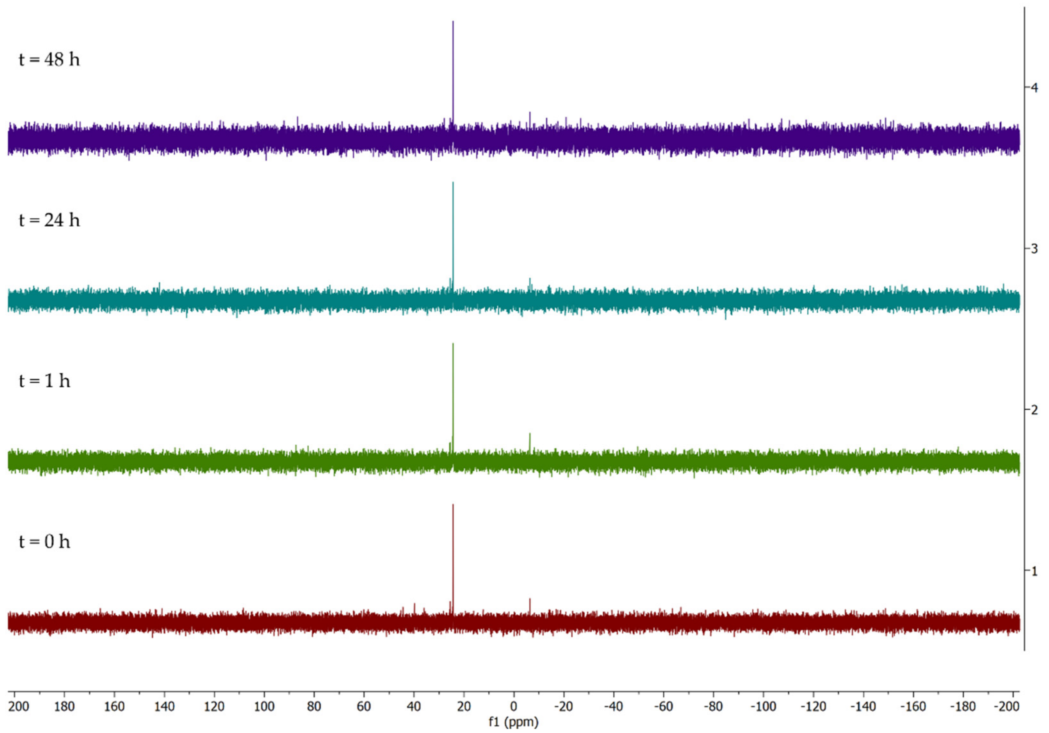


**Figure S39**. Time-dependant $^{31}P\{^{1}H\}$ NMR spectra of **5** in DMSO-$d_6$ at 298 K, after addition of one drop of PBS buffer solution in MilliQ water

## 7. FT-IR spectra

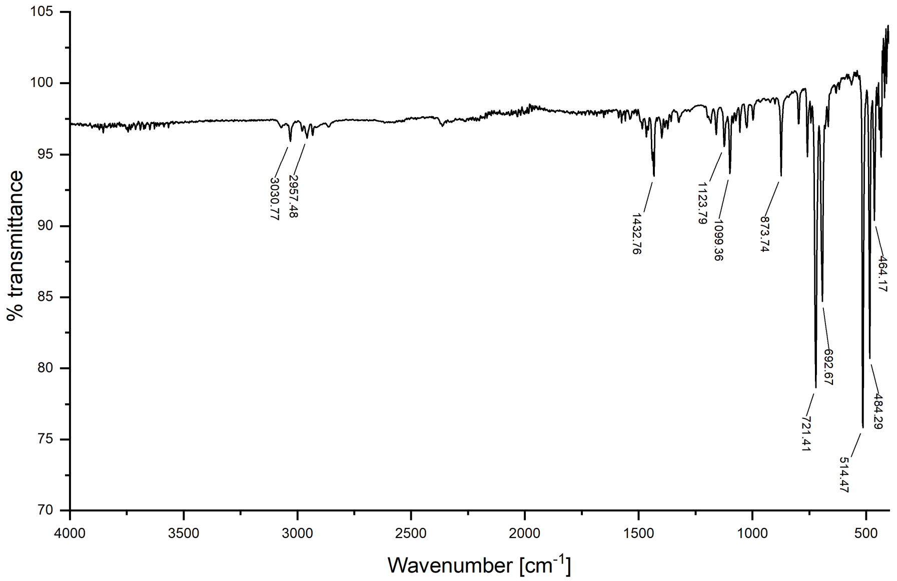


**Figure S40**. FT-IR spectrum of **1**

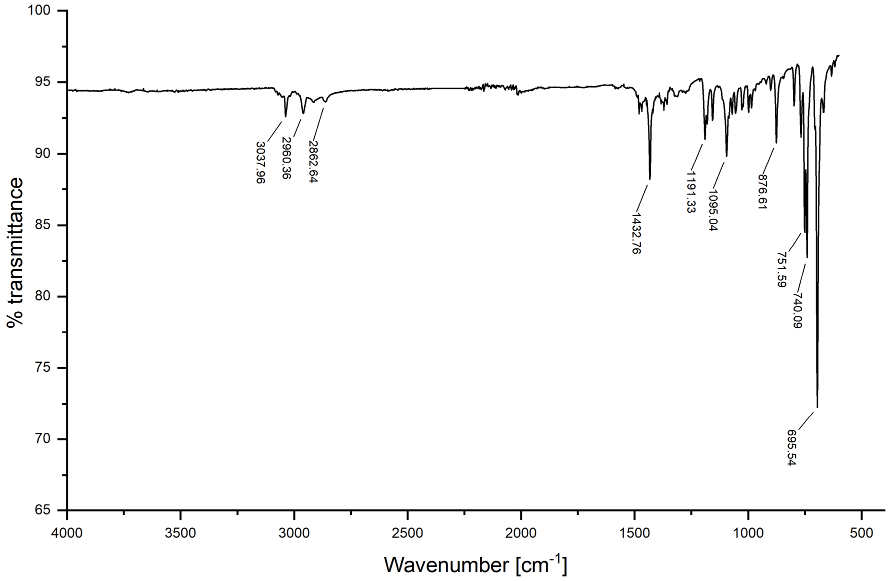


**Figure S41**. FT-IR spectrum of **2**

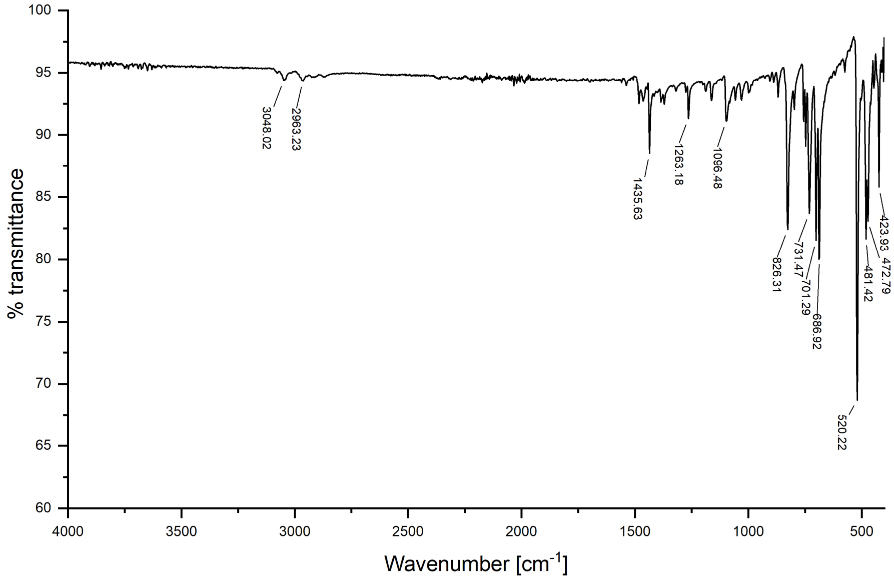


**Figure S42**. FT-IR spectrum of **3**

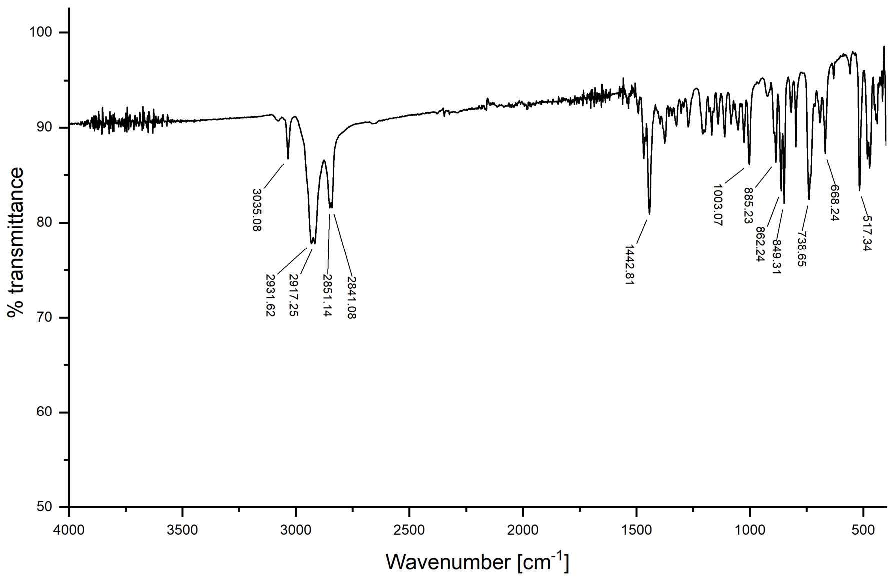


**Figure S43**. FT-IR spectrum of **4**

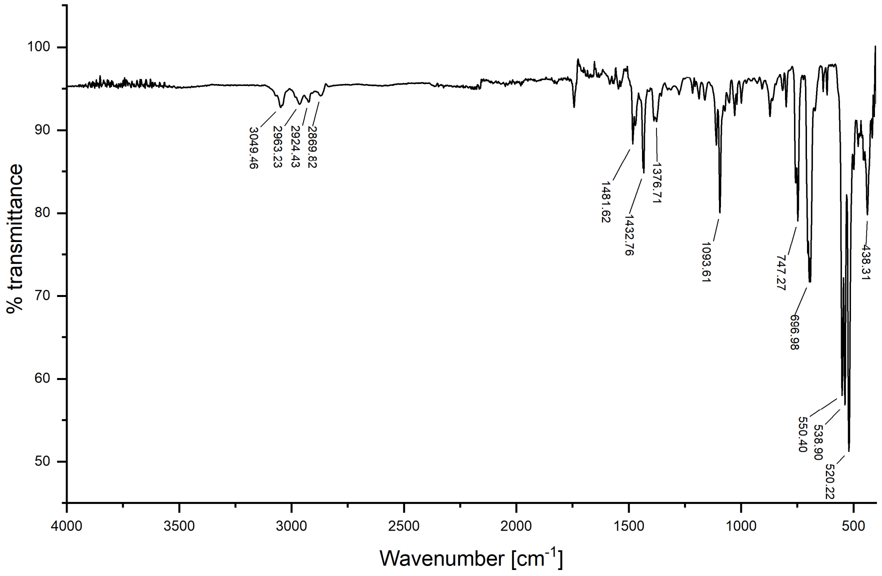


**Figure S44**. FT-IR spectrum of **5**

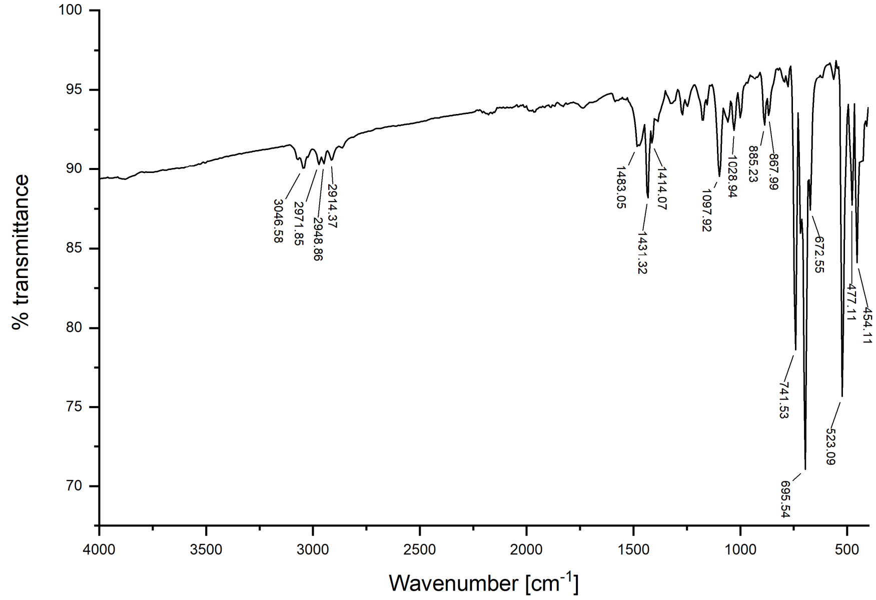


**Figure S45**. FT-IR spectrum of **6**

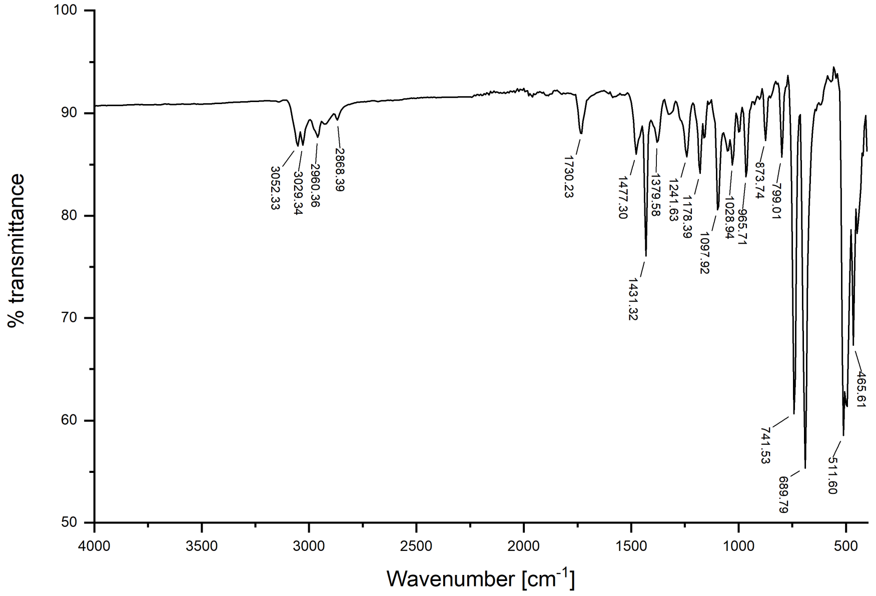


**Figure S46**. FT-IR spectrum of **7**

8. UV-Vis spectra

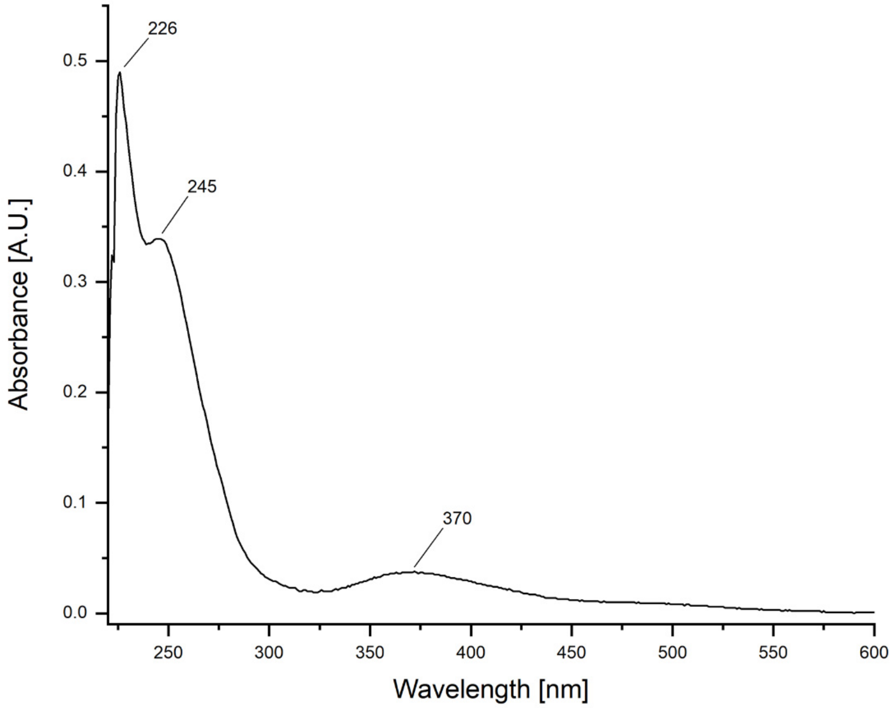


**Figure S47**. UV-Vis spectrum of **1** ($1.10^{-5}$ M, $CH_2Cl_2$, 293.15 K)

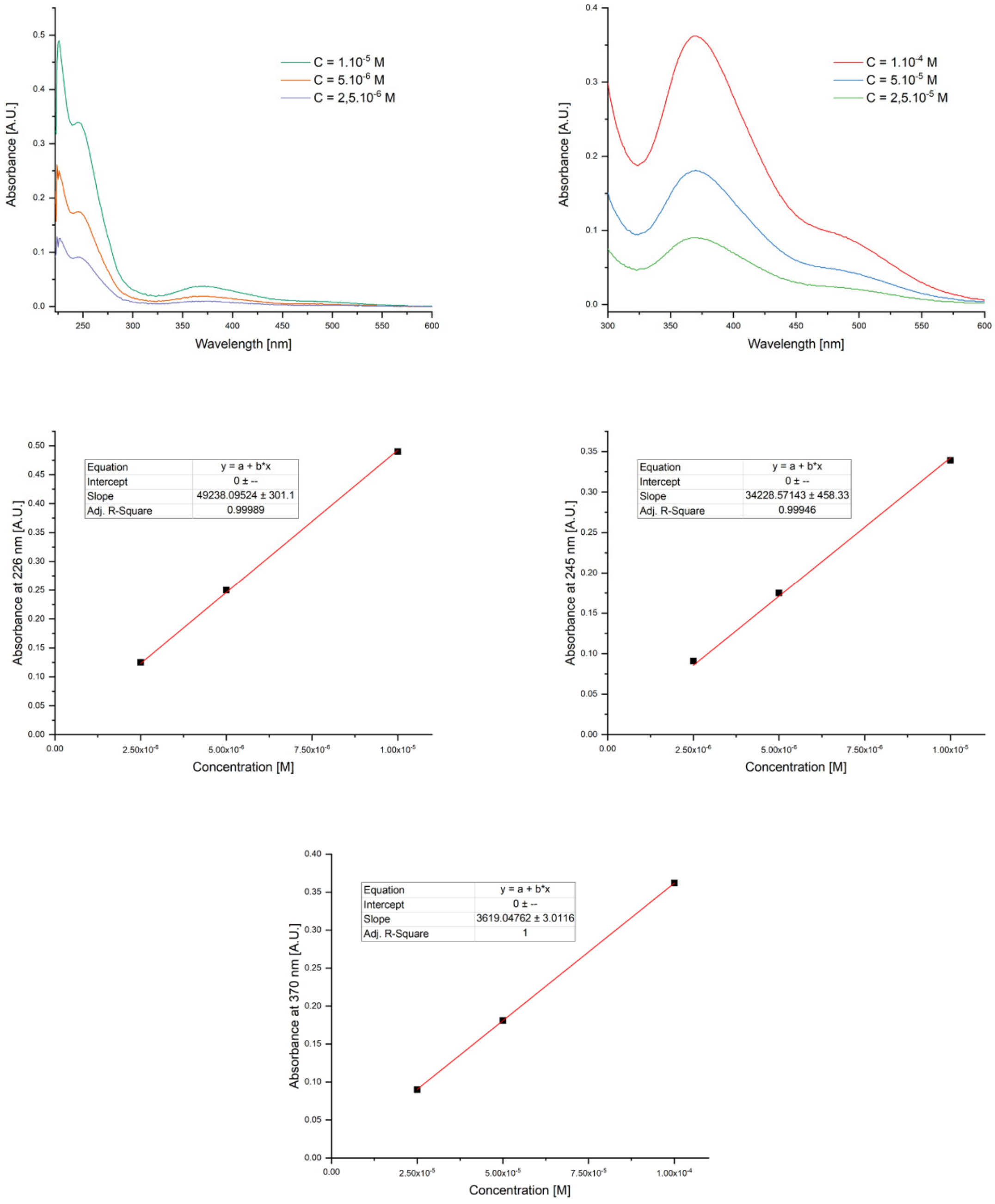


**Figure S48**. Variable concentration UV-Vis spectra of **1**, and plots of absorptions at the respective maxima for given concentrations.

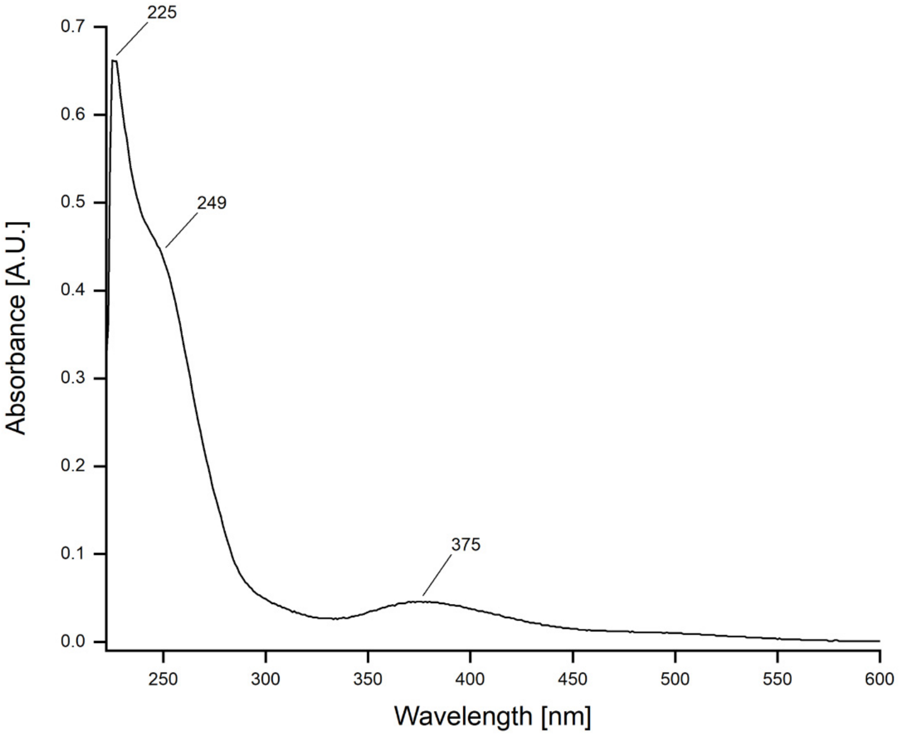


**Figure S49**. UV-Vis spectrum of **2** (1.10$^{-5}$ M, $CH_2Cl_2$, 293.15 K)

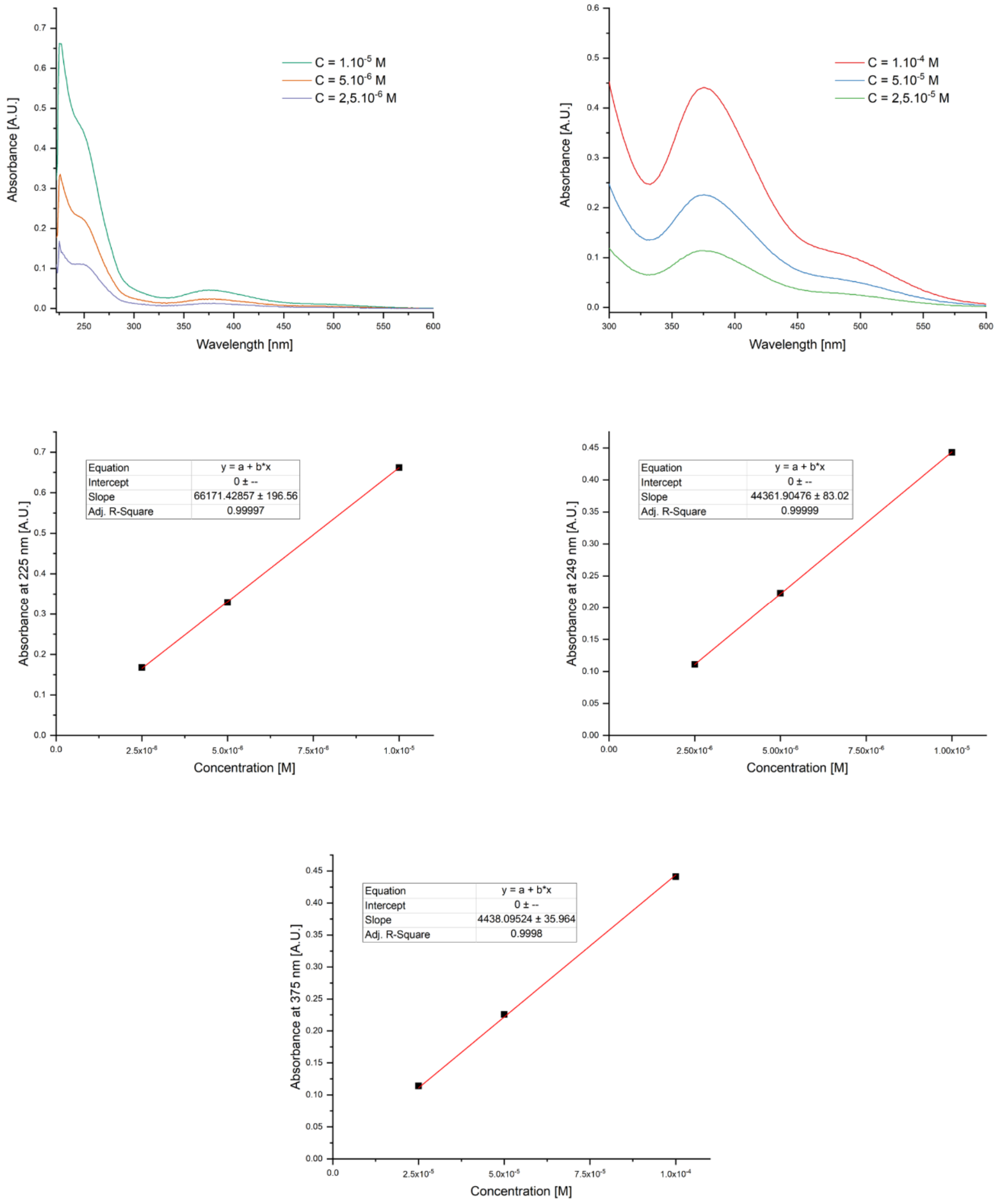


**Figure S50**. Variable concentration UV-Vis spectra of **2**, and plots of absorptions at the respective maxima for given concentrations.

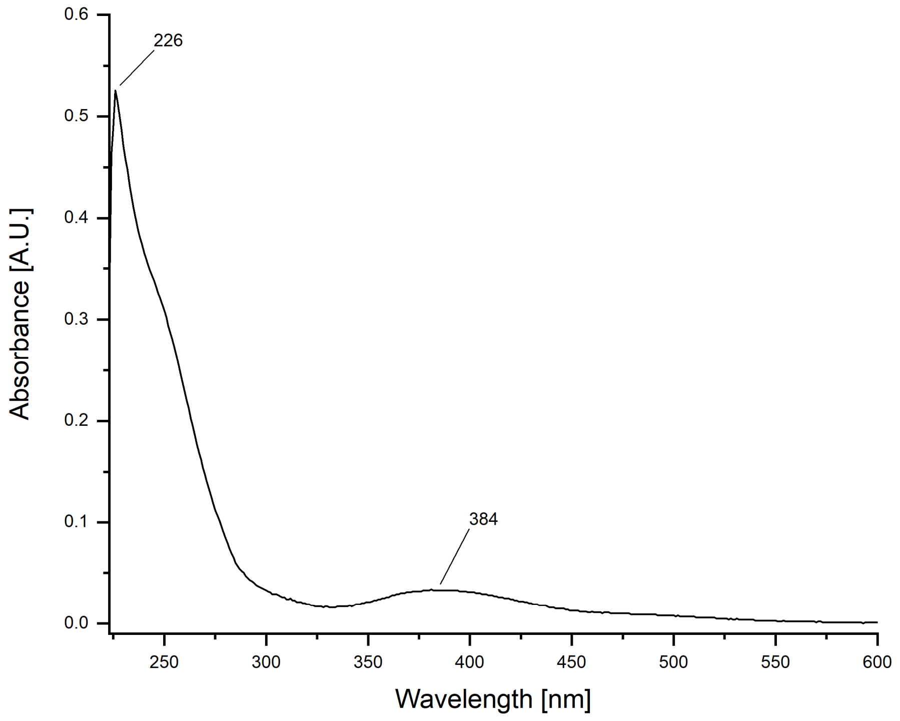


**Figure S51**. UV-Vis spectrum of **3** (1.10$^{-5}$ M, $CH_2Cl_2$, 293.15 K)

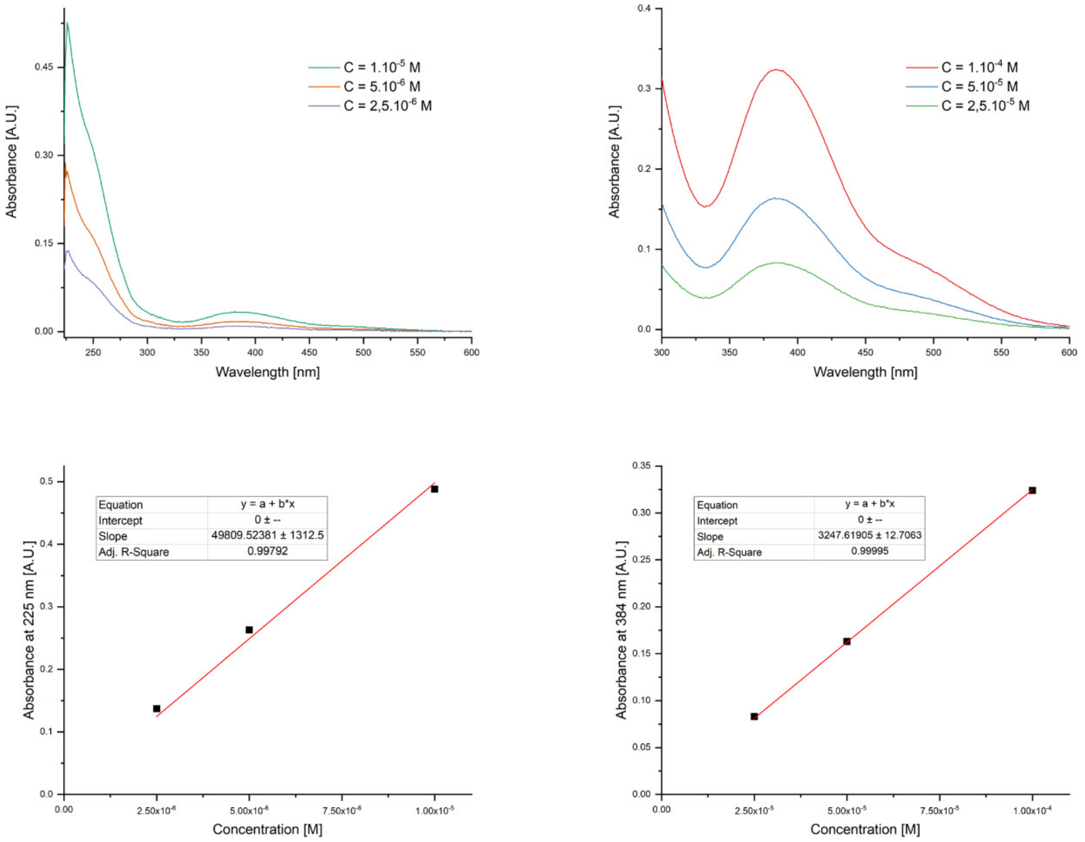


**Figure S52**. Variable concentration UV-Vis spectra of **3**, and plots of absorptions at the respective maxima for given concentrations.

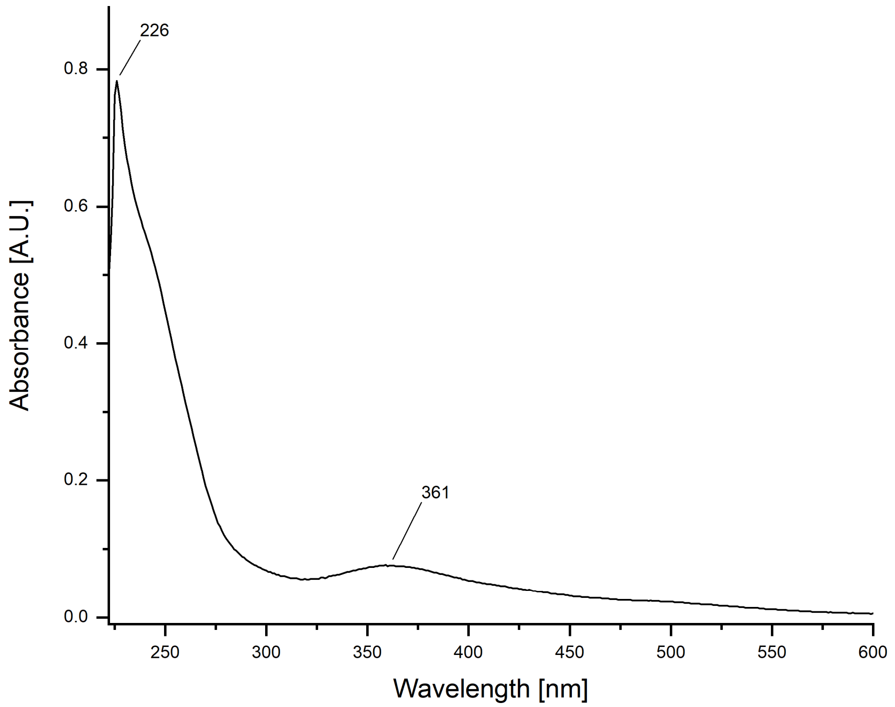


**Figure S53**. UV-Vis spectrum of **4** (2,5.10$^{-5}$ M, $CH_2Cl_2$, 293.15 K)

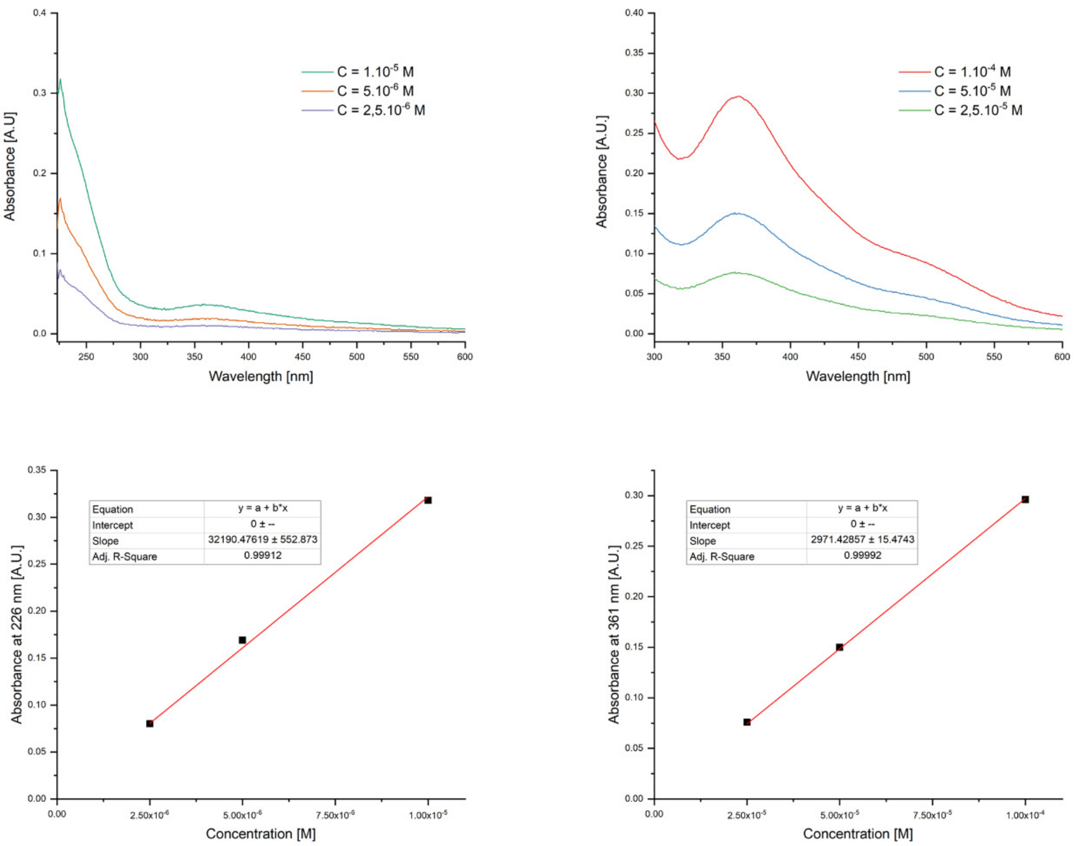


**Figure S54**. Variable concentration UV-Vis spectra of **4**, and plots of absorptions at the respective maxima for given concentrations.

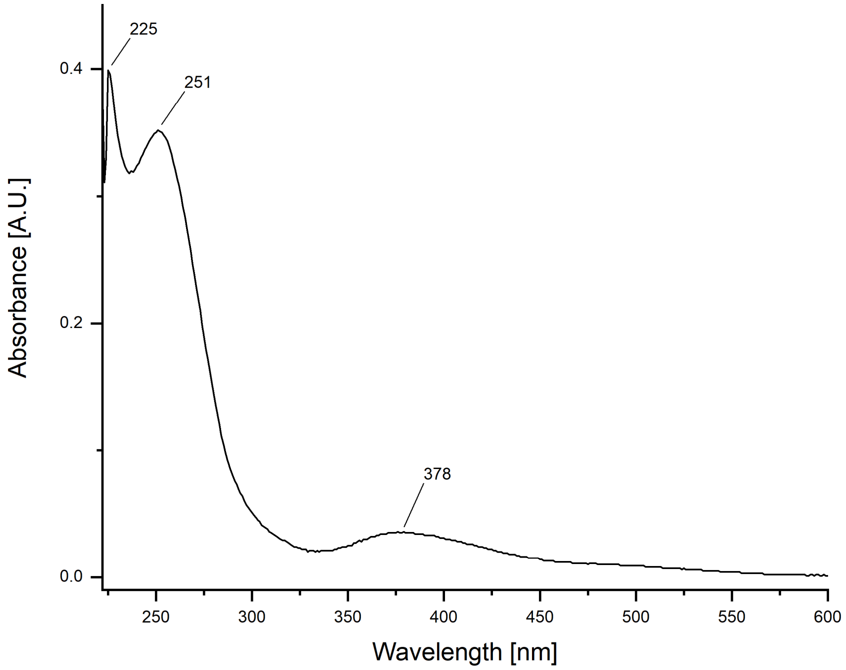


**Figure S55**. UV-Vis spectrum of **5** ($1.10^{-5}$ M, $CH_2Cl_2$, 293.15 K)

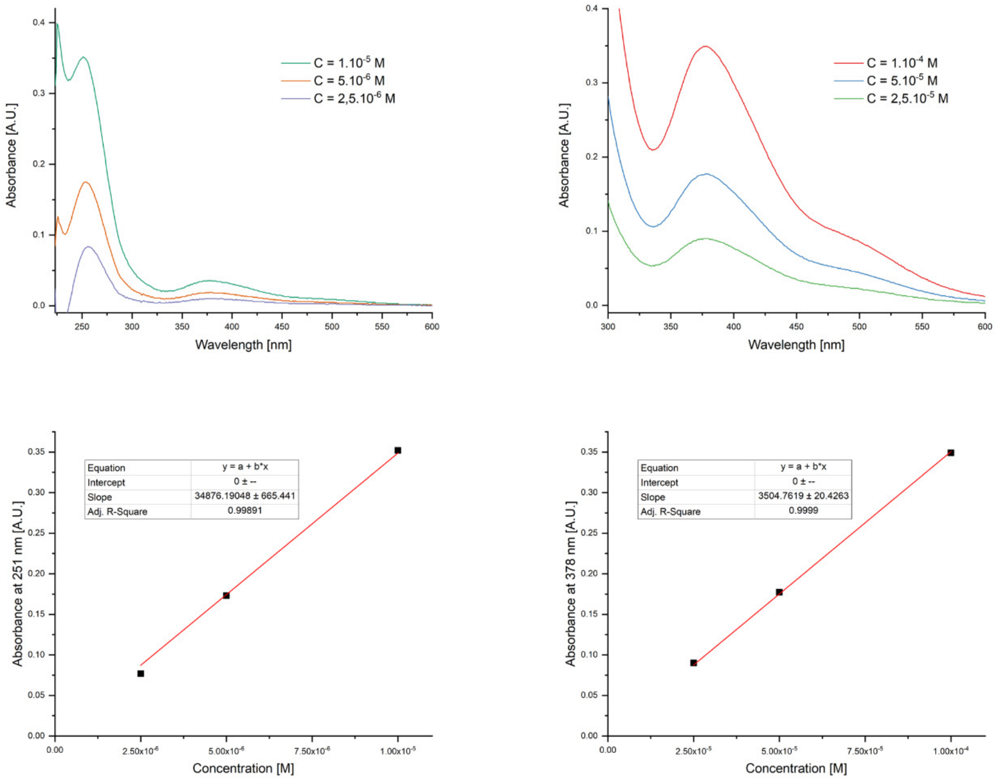


**Figure S56**. Variable concentration UV-Vis spectra of **5**, and plots of absorptions at the respective maxima for given concentrations.

9. Mass spectra

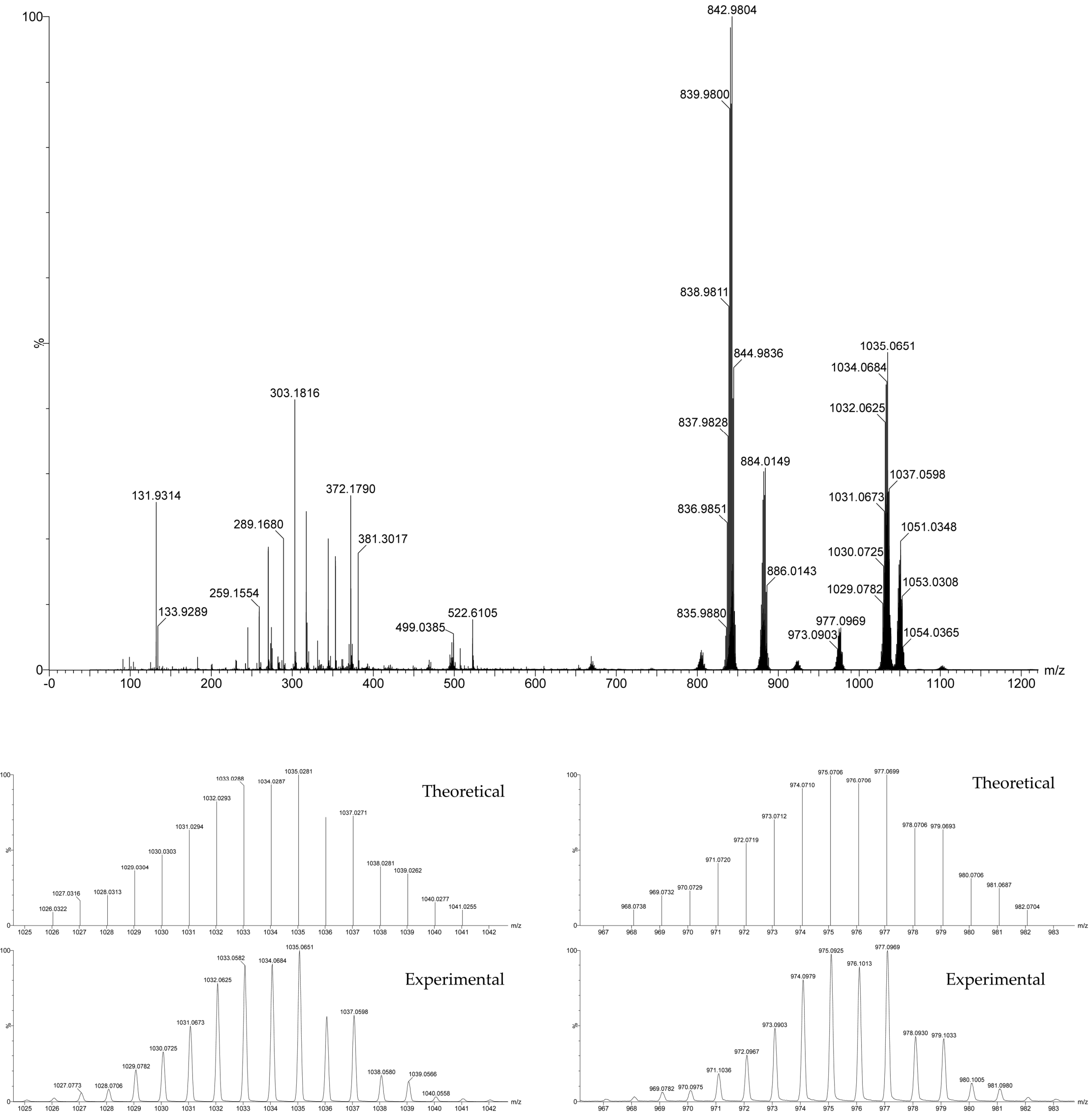


**Figure S57**. Mass spectrum of **1** (acetonitrile, top), and relevant fragments (bottom)

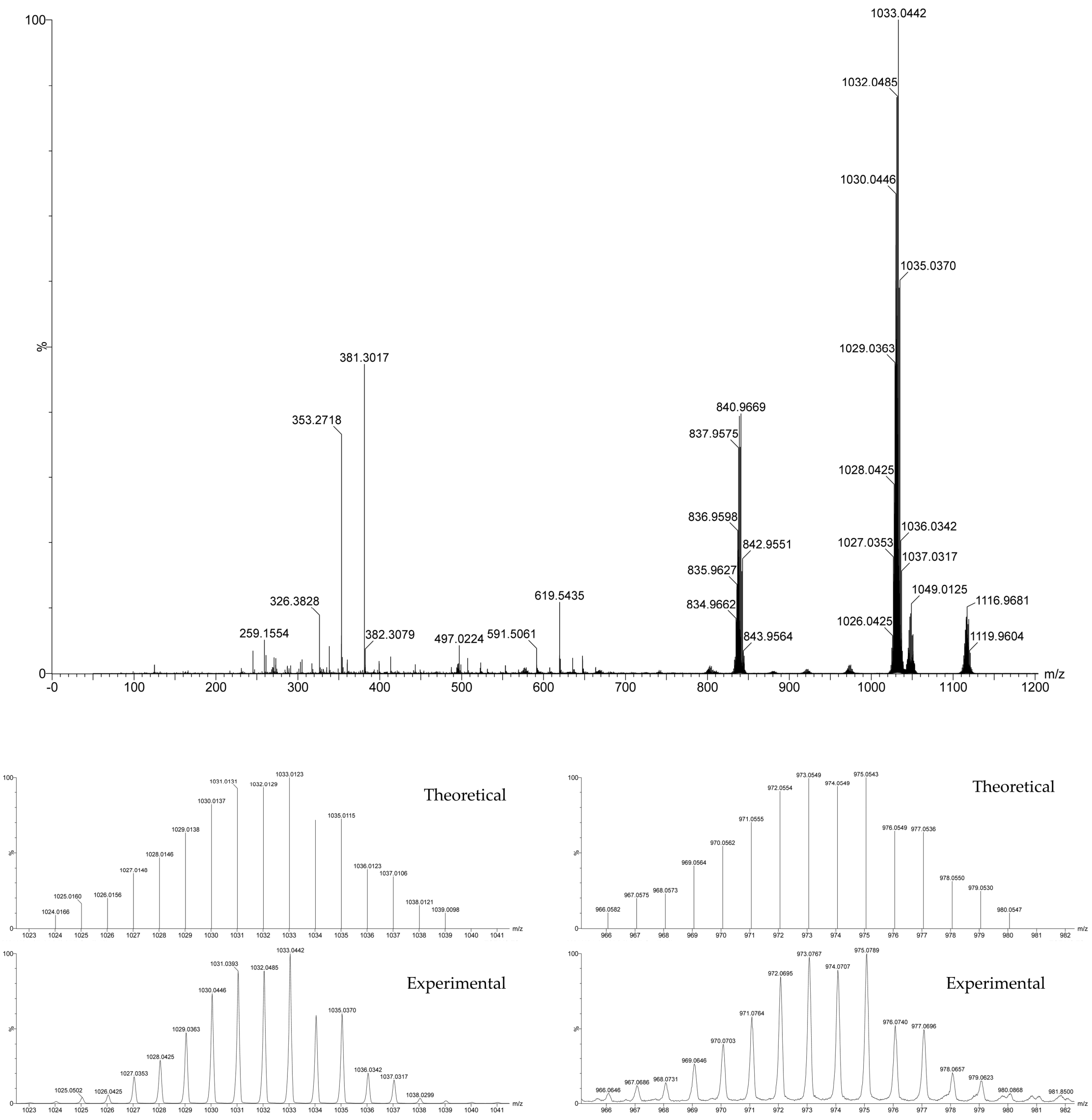


**Figure S58**. Mass spectrum of **2** (acetonitrile, top), and relevant fragments (bottom)

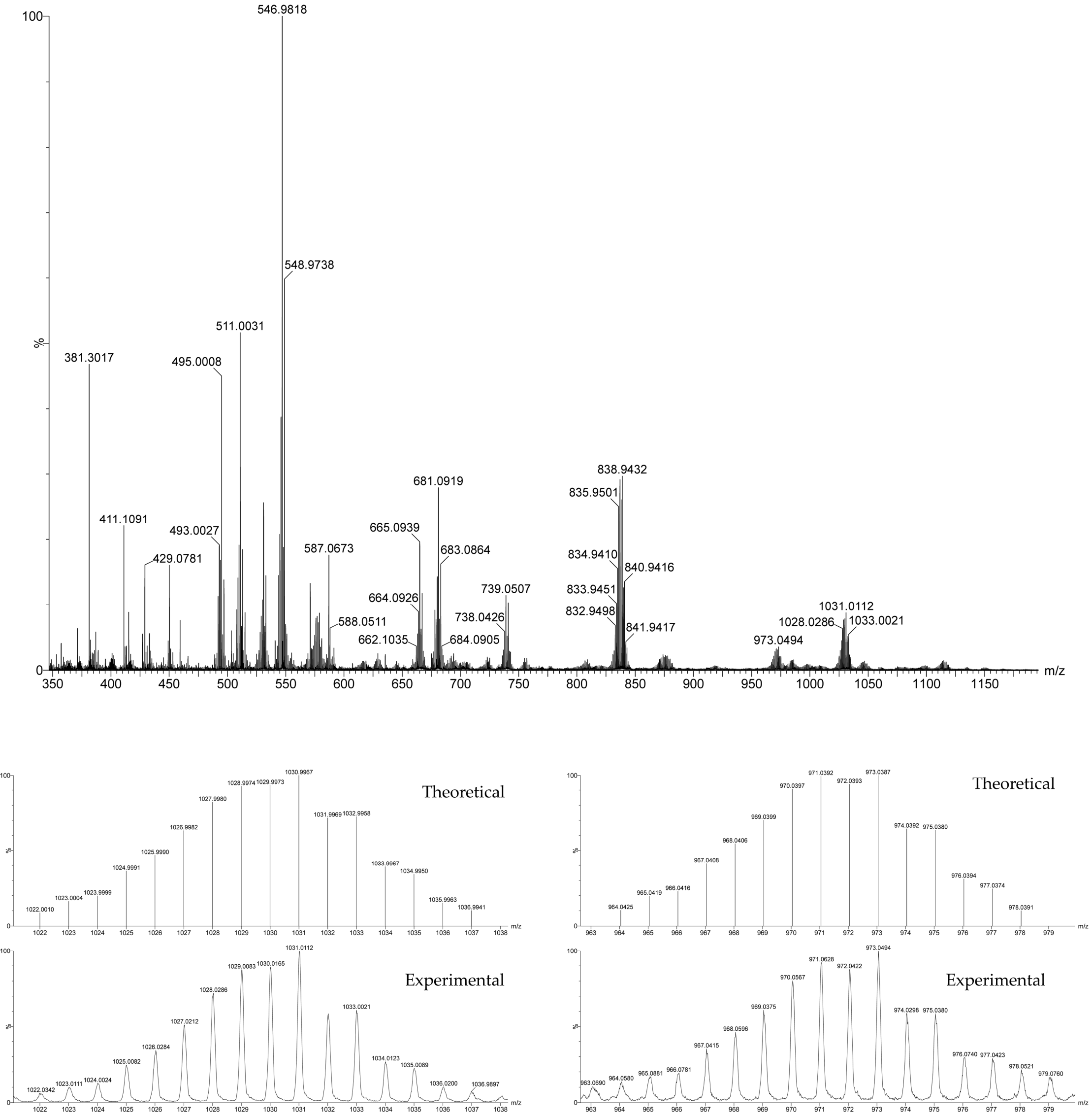


**Figure S59**. Mass spectrum of **3** (acetonitrile, top), and relevant fragments (bottom)

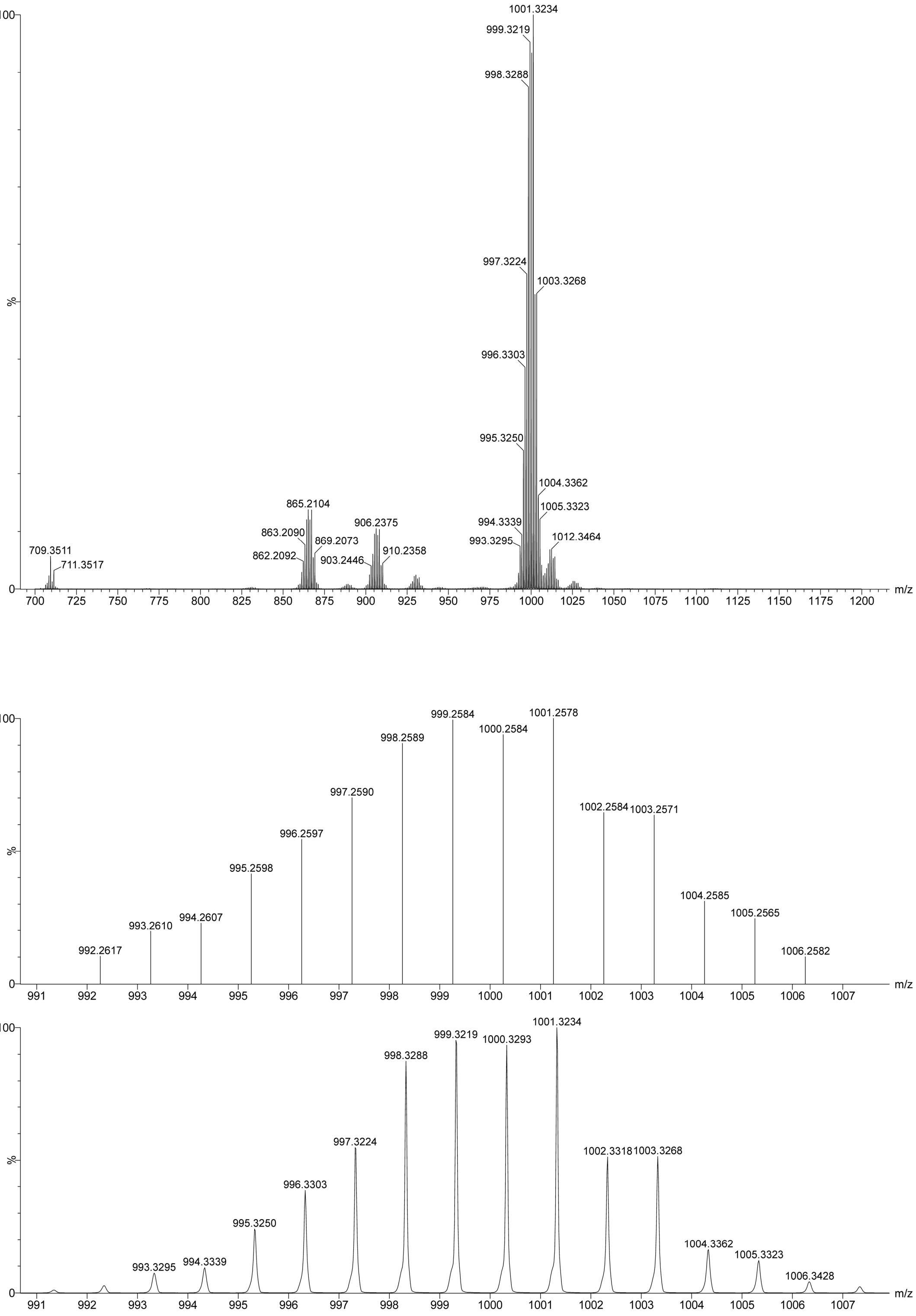


**Figure S60**. Mass spectrum of **4** (acetonitrile, top), and relevant fragments (bottom)

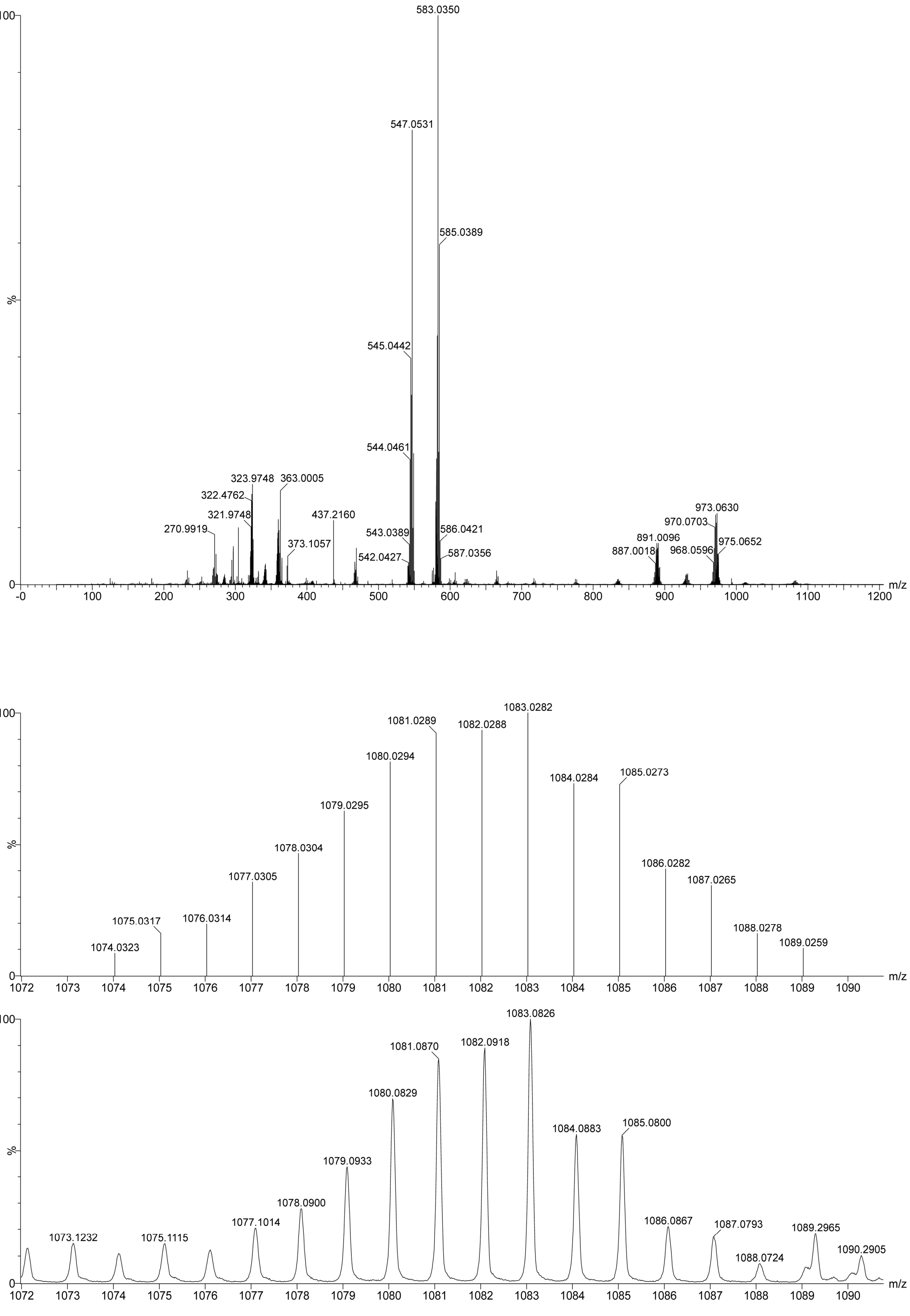


**Figure S61**. Mass spectrum of **5** (acetonitrile, top), and relevant fragments (bottom)

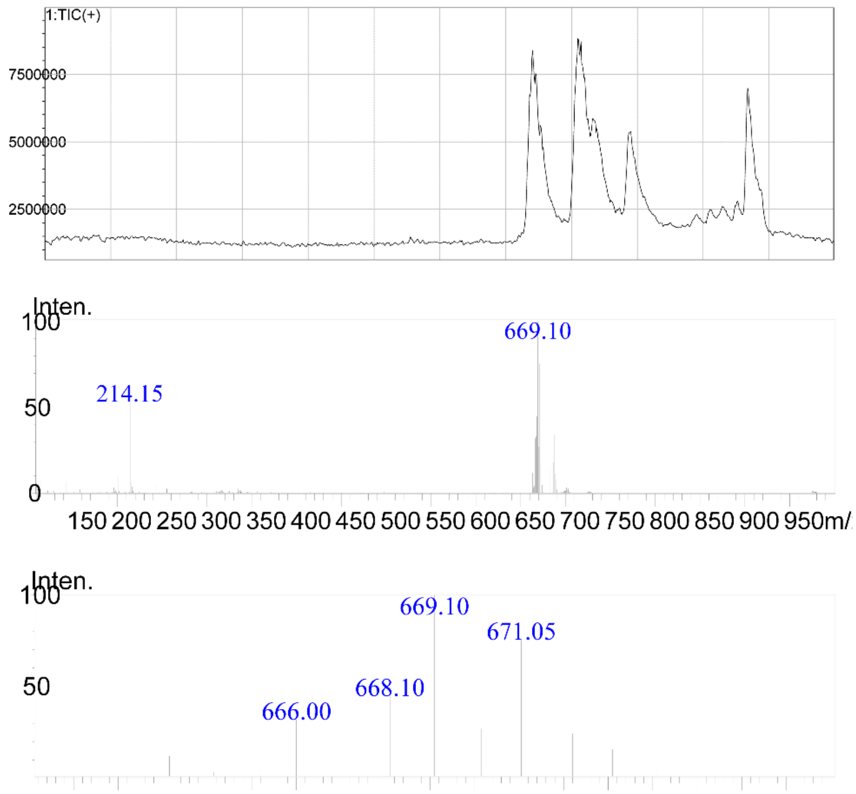


**Figure S62**. LC chromatogram (top), mass spectrum (middle) and [M – Cl]⁺ fragment (bottom) of **6**

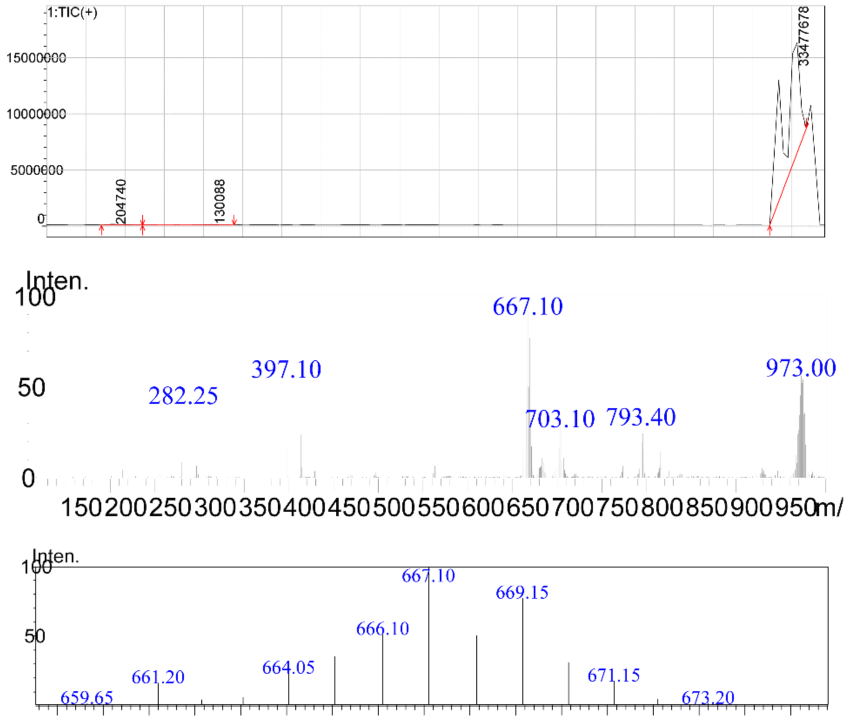


**Figure S63**. LC chromatogram (top), mass spectrum (middle) and [M – Cl]⁺ fragment (bottom) of **7**

10. Cell viability studies

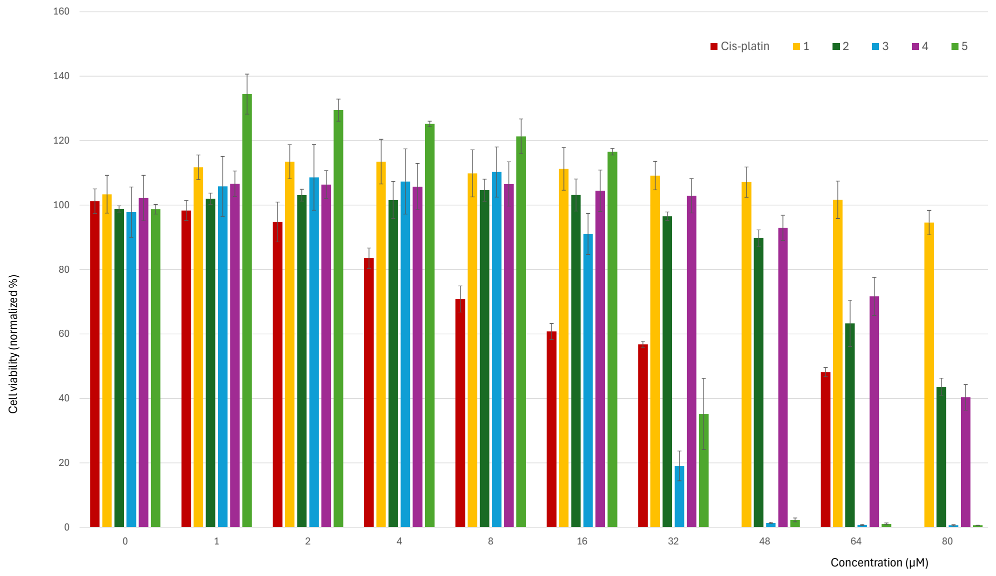


**Figure S64**. Presto blue cell cytotoxicity assay for HeLa cells in presence of either cisplatin (red column), complex **1** (yellow column), complex **2** (dark green column), complex **3** (blue column), complex **4** (purple column) or complex **5** (green column). Data are presented as mean ± SD (standard deviation) (n = 3). Note: cell viability of cisplatin was not measured at 48 and 80 μM, however the data from cis-platin with the range between 0 and 128 μM was used to calculate its corresponding $IC_{50}$ value (*Table 3*).

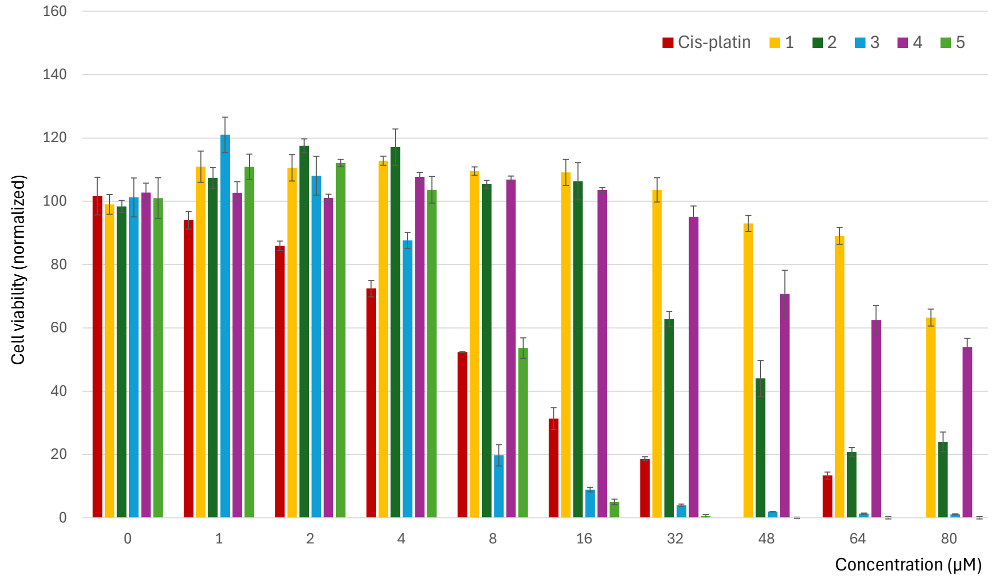


**Figure S65**. Presto blue cell cytotoxicity assay for Ect1/Ect6 cells in presence of either Cis-platin (red column), complex **1** (yellow column), complex **2** (dark green column), complex **3** (blue column), complex **4** (purple column) or complex **5** (green column). Data are presented as mean ± SD (standard deviation) (n = 3). Note: cell viability of cisplatin was not measured at 48 and 80 μM, however the data with the range between 0 and 128 μM was used to calculate its corresponding $IC_{50}$ value (*Table 3*).

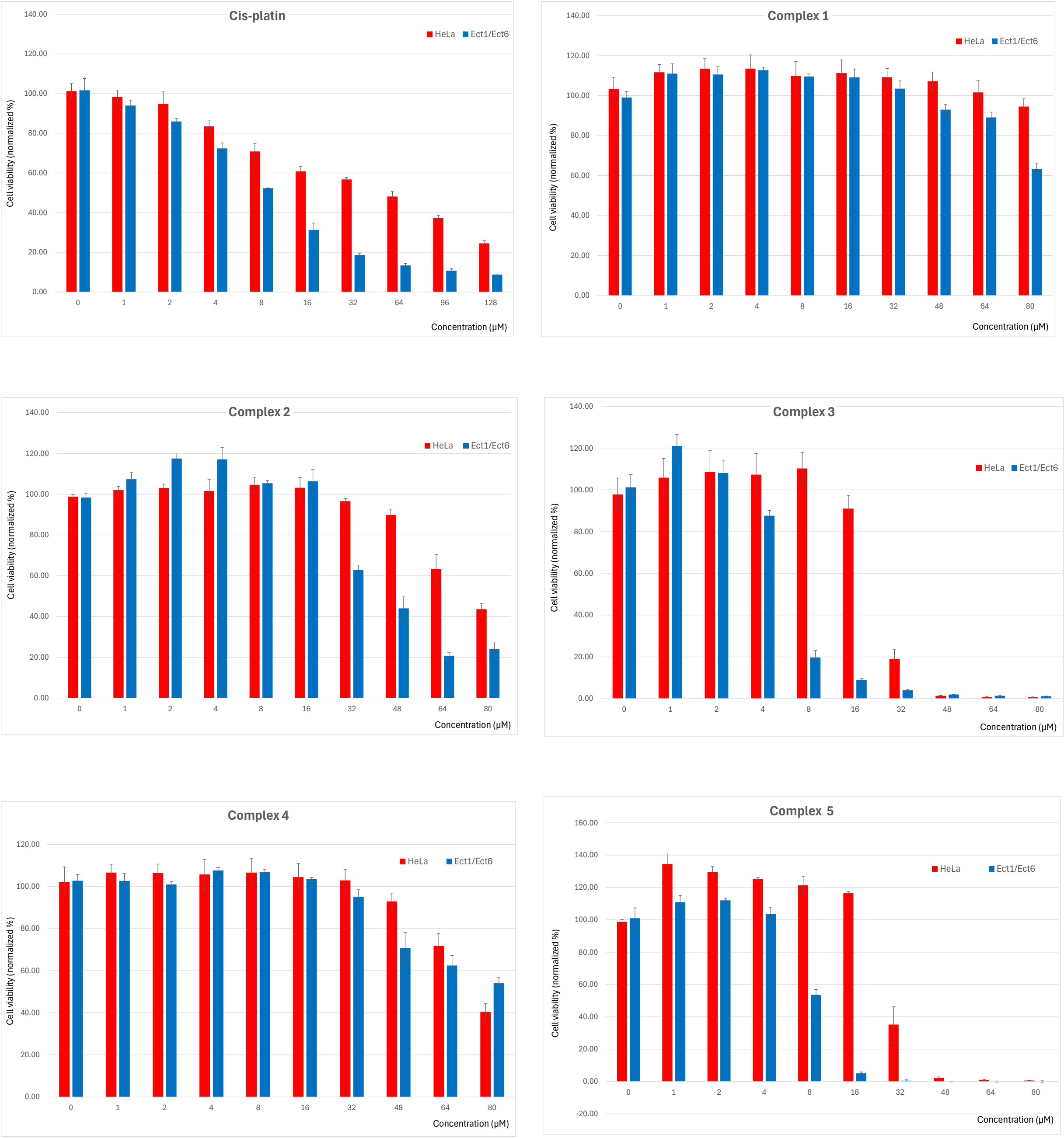


**Figure S66**. Presto blue cell cytotoxicity assay for Hela (red column) vs Ect1/Ect6 cells (blue column) in presence of cisplatin (control) and complexes **1** – **5**. Data are presented as mean ± SD (standard deviation) (n = 3).

## 11. IC50 plots

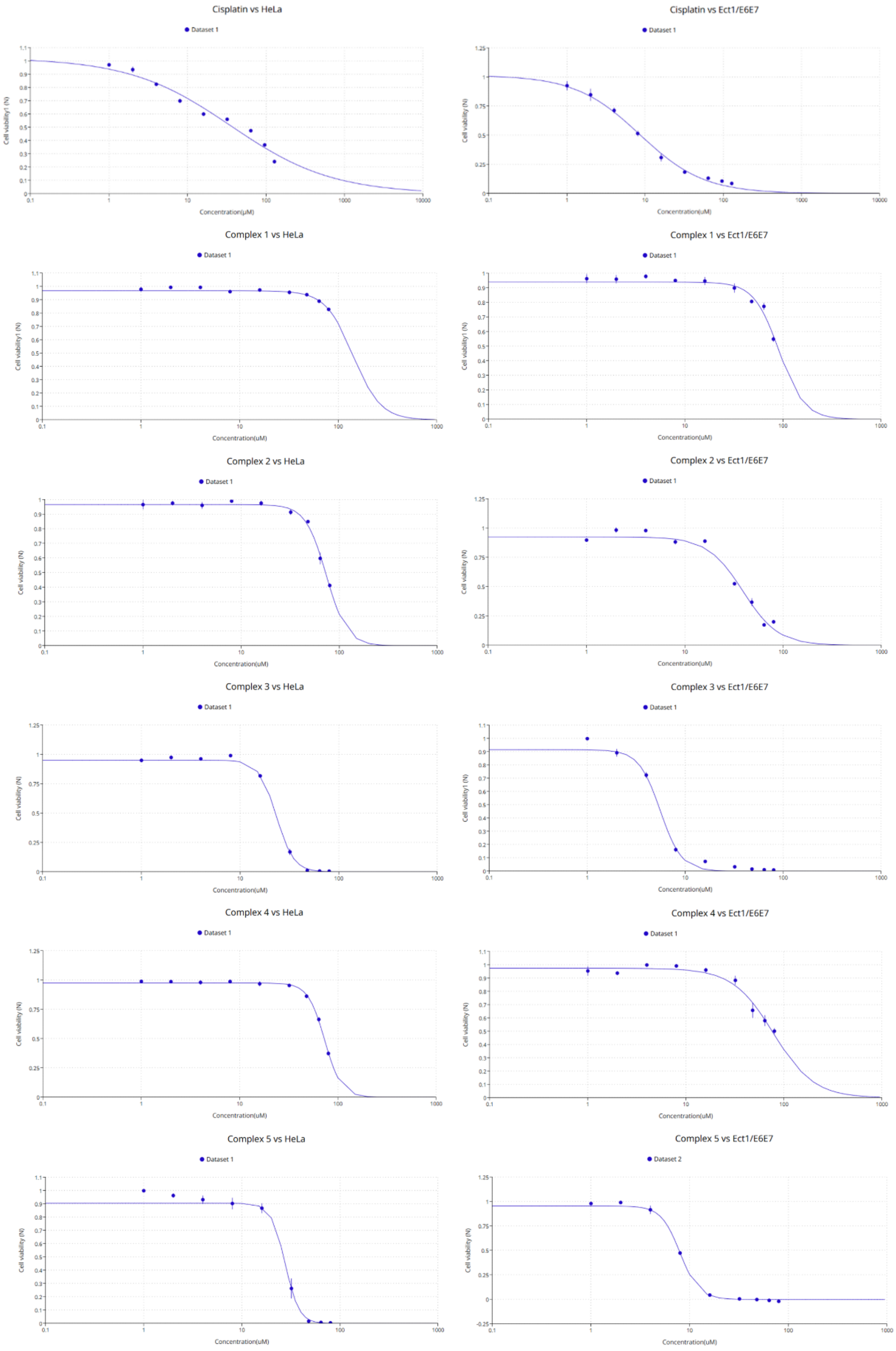


**Figure S67**. IC50 plots for all tested complexes against HeLa (left) and Ect1/E6E7 (right)

12. Single crystal X-ray diffraction analyses

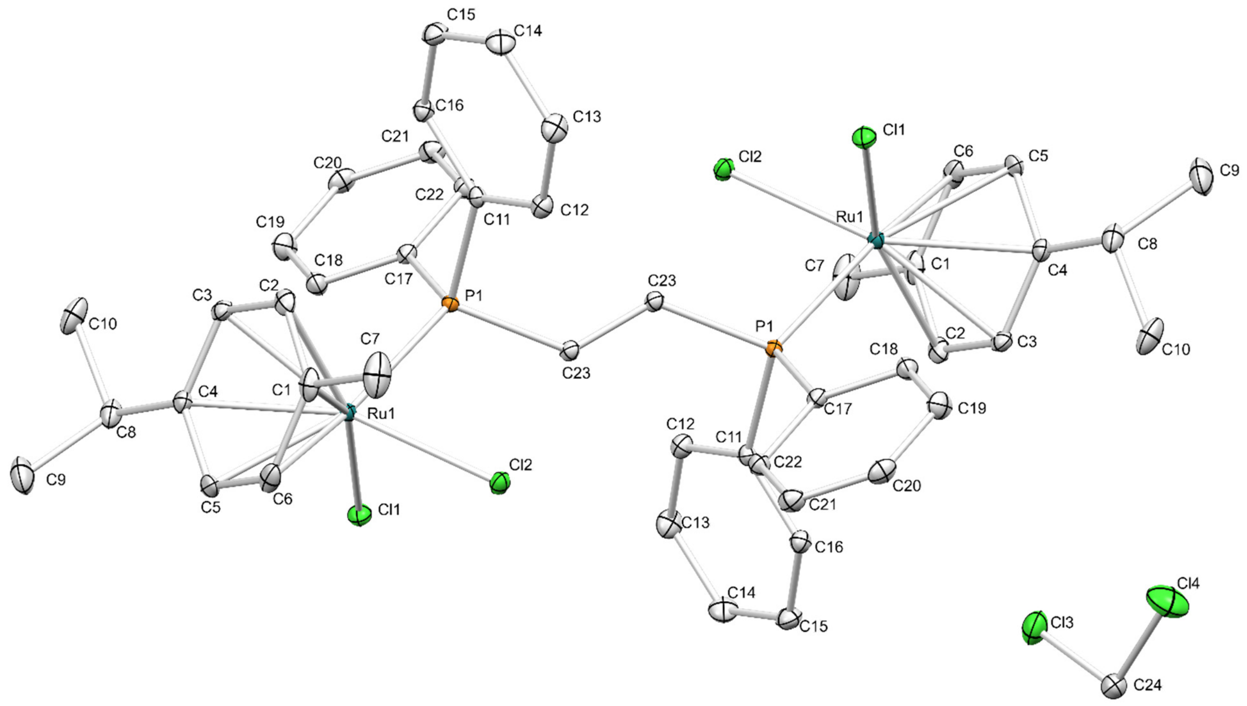


**Figure S68**. ORTEP representation of **2·CH2Cl2** with complete atom labellings, in the solid state determined by single crystal X-ray diffraction analysis. Thermal ellipsoids represented at the 30% probability level. H atoms omitted for clarity.

**Table S1. Crystal data and structure refinement for complex 2**

| | |
|---|---|
| Empirical formula | $C_{24}H_{27}Cl_4PRu$ |
| Formula weight | 589.29 |
| Temperature/K | 100.00(10) |
| Crystal system | triclinic |
| Space group | P-1 |
| a/Å | 11.0487(3) |
| b/Å | 11.3414(3) |
| c/Å | 11.9805(3) |
| α/° | 80.712(2) |
| β/° | 64.941(2) |
| γ/° | 63.925(2) |
| Volume/Å$^3$ | 1220.96(6) |

| | |
|---|---|
| Z | 2 |
| $\rho_{calc}$g/cm$^3$ | 1.603 |
| μ/mm$^{-1}$ | 1.156 |
| F(000) | 596.0 |
| Crystal size/mm$^3$ | 0.267 × 0.181 × 0.042 |
| Radiation | Mo K$\alpha$ ($\lambda$ = 0.71073) |
| 2Θ range for data collection/° | 5.404 to 66.184 |
| Index ranges | -16 ≤ h ≤ 16, -17 ≤ k ≤ 16, -18 ≤ l ≤ 17 |
| Reflections collected | 44250 |
| Independent reflections | 8266 [$R_{int}$ = 0.0391, $R_{sigma}$ = 0.0320] |
| Data/restraints/parameters | 8266/0/278 |
| Goodness-of-fit on F$^2$ | 1.051 |
| Final R indexes [I>=2σ (I)] | $R_1$ = 0.0273, w$R_2$ = 0.0586 |
| Final R indexes [all data] | $R_1$ = 0.0327, w$R_2$ = 0.0602 |
| Largest diff. peak/hole / e Å$^{-3}$ | 0.90/-0.73 |

**Table S2. Fractional Atomic Coordinates (×10$^4$) and Equivalent Isotropic Displacement Parameters (Å$^2$×10$^3$) for complex 2. $U_{eq}$ is defined as 1/3 of the trace of the orthogonalised $U_{IJ}$ tensor.**

| Atom | *x* | *y* | *z* | U(eq) |
|---|---|---|---|---|
| Ru1 | 4581.9(2) | 8589.9(2) | 3474.1(2) | 11.53(3) |
| Cl2 | 6056.0(4) | 9810.9(4) | 2447.6(3) | 17.04(7) |
| P1 | 4480.0(4) | 8628.0(3) | 1556.1(3) | 10.75(7) |
| Cl1 | 2442.2(4) | 10644.4(3) | 3917.4(3) | 18.54(7) |
| Cl3 | 4740.3(5) | 3784.9(5) | 2392.5(4) | 32.46(10) |
| Cl4 | 1634.4(5) | 4383.7(6) | 3780.5(5) | 45.32(13) |
| C23 | 4623.5(15) | 10024.0(14) | 608.6(12) | 12.7(2) |
| C17 | 2831.6(15) | 8641.7(13) | 1537.9(13) | 13.1(2) |
| C11 | 5994.1(15) | 7196.0(13) | 610.8(12) | 13.1(2) |
| C6 | 5548.8(18) | 7976.8(16) | 4872.2(14) | 21.9(3) |
| C4 | 3404.9(16) | 7619.9(14) | 5102.8(13) | 16.0(3) |
| C5 | 4065.1(18) | 8252.6(15) | 5470.6(13) | 19.4(3) |
| C3 | 4315.6(16) | 6738.1(14) | 4079.1(13) | 16.0(3) |
| C16 | 5779.3(16) | 6087.4(14) | 547.4(13) | 15.9(3) |

| | | | | |
|---|---|---|---|---|
| C1 | 6471.9(17) | 7068.8(16) | 3837.6(15) | 21.7(3) |
| C2 | 5834.4(16) | 6464.3(14) | 3454.1(14) | 18.8(3) |
| C22 | 2722.4(16) | 8648.1(14) | 415.7(13) | 16.1(3) |
| C18 | 1655.1(16) | 8684.1(15) | 2639.4(14) | 17.8(3) |
| C8 | 1791.1(17) | 7894.8(16) | 5840.3(15) | 22.6(3) |
| C12 | 7418.3(16) | 7138.1(15) | 81.7(14) | 16.8(3) |
| C15 | 6973.2(17) | 4937.9(15) | -40.4(15) | 20.9(3) |
| C19 | 390.5(17) | 8714.9(17) | 2620.6(15) | 22.3(3) |
| C24 | 3313.3(18) | 3296.8(16) | 2713.1(15) | 22.5(3) |
| C14 | 8379.5(17) | 4888.7(16) | -580.0(16) | 23.9(3) |
| C21 | 1453.9(17) | 8685.1(15) | 405.5(15) | 19.8(3) |
| C13 | 8599.7(16) | 5993.0(16) | -524.0(15) | 21.9(3) |
| C10 | 1338.3(19) | 6954.3(18) | 5494.6(19) | 31.1(4) |
| C20 | 289.2(16) | 8715.8(16) | 1507.4(15) | 20.7(3) |
| C7 | 8069.1(19) | 6788(2) | 3159.9(19) | 36.5(5) |
| C9 | 1453(2) | 7802(2) | 7226.9(17) | 36.3(4) |

**Table S3. Anisotropic Displacement Parameters (Å$^2$×10$^3$) for complex 2. The Anisotropic displacement factor exponent takes the form: $-2\pi^2[h^2a^{*2}U_{11}+2hka^*b^*U_{12}+\ldots]$.**

| Atom | $U_{11}$ | $U_{22}$ | $U_{33}$ | $U_{23}$ | $U_{13}$ | $U_{12}$ |
|---|---|---|---|---|---|---|
| Ru1 | 14.78(6) | 12.47(6) | 9.96(5) | 1.76(4) | -5.80(4) | -7.48(4) |
| Cl2 | 22.23(16) | 20.12(17) | 16.26(15) | 4.28(12) | -9.45(13) | -14.62(13) |
| P1 | 12.86(15) | 11.11(15) | 10.18(15) | 1.40(11) | -5.21(12) | -6.32(12) |
| Cl1 | 18.88(16) | 14.49(16) | 18.74(16) | -0.46(12) | -5.09(13) | -5.95(12) |
| Cl3 | 39.4(2) | 36.1(2) | 32.4(2) | 11.62(18) | -16.38(19) | -26.0(2) |
| Cl4 | 32.3(2) | 52.0(3) | 38.9(3) | -18.3(2) | -5.6(2) | -8.4(2) |
| C23 | 15.3(6) | 11.5(6) | 14.0(6) | 2.2(5) | -8.3(5) | -6.0(5) |
| C17 | 14.7(6) | 11.9(6) | 14.9(6) | 2.2(5) | -7.2(5) | -6.6(5) |
| C11 | 15.5(6) | 12.9(6) | 11.6(6) | 2.0(5) | -6.5(5) | -5.9(5) |
| C6 | 33.6(8) | 27.7(8) | 21.0(7) | 12.8(6) | -20.5(7) | -21.5(7) |
| C4 | 20.8(7) | 14.3(6) | 14.6(6) | 4.1(5) | -7.6(5) | -9.5(5) |
| C5 | 32.2(8) | 20.2(7) | 11.2(6) | 4.7(5) | -10.5(6) | -14.9(6) |
| C3 | 22.3(7) | 11.4(6) | 17.6(7) | 3.0(5) | -10.7(6) | -8.0(5) |

| | | | | | | |
|---|---|---|---|---|---|---|
| C16 | 17.3(6) | 14.8(6) | 15.9(6) | 1.5(5) | -7.1(5) | -6.8(5) |
| C1 | 19.8(7) | 25.6(8) | 22.5(7) | 12.6(6) | -13.5(6) | -11.0(6) |
| C2 | 21.2(7) | 13.6(6) | 16.7(7) | 4.4(5) | -8.2(6) | -3.7(5) |
| C22 | 18.1(6) | 17.2(7) | 14.9(6) | 1.7(5) | -7.3(5) | -8.7(5) |
| C18 | 18.7(7) | 21.4(7) | 14.5(6) | 0.7(5) | -5.6(5) | -10.2(6) |
| C8 | 19.6(7) | 19.0(7) | 22.5(8) | 4.8(6) | -3.6(6) | -8.3(6) |
| C12 | 17.0(6) | 16.5(7) | 18.6(7) | 2.1(5) | -8.1(5) | -7.8(5) |
| C15 | 25.7(7) | 14.1(7) | 23.3(7) | -0.3(5) | -10.3(6) | -7.8(6) |
| C19 | 16.5(7) | 27.5(8) | 22.6(8) | -1.4(6) | -3.7(6) | -12.0(6) |
| C24 | 27.2(8) | 21.6(8) | 20.7(7) | 1.0(6) | -8.7(6) | -12.7(6) |
| C14 | 20.1(7) | 16.9(7) | 26.4(8) | -2.8(6) | -6.9(6) | -1.9(6) |
| C21 | 23.6(7) | 19.4(7) | 22.2(7) | 2.4(6) | -14.1(6) | -9.8(6) |
| C13 | 14.0(6) | 21.2(7) | 26.6(8) | 1.2(6) | -6.6(6) | -5.6(6) |
| C10 | 25.4(8) | 27.4(9) | 44.6(11) | 12.8(8) | -14.3(8) | -18.1(7) |
| C20 | 16.7(7) | 20.6(7) | 29.6(8) | 0.4(6) | -11.3(6) | -9.6(6) |
| C7 | 20.4(8) | 44.4(11) | 42.5(11) | 20.9(9) | -16.0(8) | -14.8(8) |
| C9 | 32.7(9) | 37.5(10) | 22.4(9) | 6.0(7) | 0.7(7) | -13.9(8) |

**Table S4. Bond Lengths for complex 2.**

| Atom | Atom | Length/Å | Atom | Atom | Length/Å |
|---|---|---|---|---|---|
| Ru1 | Cl2 | 2.4121(4) | C6 | C5 | 1.387(2) |
| Ru1 | P1 | 2.3404(4) | C6 | C1 | 1.433(2) |
| Ru1 | Cl1 | 2.4070(4) | C4 | C5 | 1.441(2) |
| Ru1 | C6 | 2.2175(15) | C4 | C3 | 1.408(2) |
| Ru1 | C4 | 2.2637(14) | C4 | C8 | 1.524(2) |
| Ru1 | C5 | 2.2212(14) | C3 | C2 | 1.423(2) |
| Ru1 | C3 | 2.2123(14) | C16 | C15 | 1.390(2) |
| Ru1 | C1 | 2.2108(15) | C1 | C2 | 1.406(2) |
| Ru1 | C2 | 2.1875(14) | C1 | C7 | 1.500(2) |
| P1 | C23 | 1.8189(14) | C22 | C21 | 1.389(2) |
| P1 | C17 | 1.8239(14) | C18 | C19 | 1.392(2) |
| P1 | C11 | 1.8218(14) | C8 | C10 | 1.532(3) |

| | | | | | |
|---|---|---|---|---|---|
| Cl3 | C24 | 1.7679(17) | C8 | C9 | 1.537(2) |
| Cl4 | C24 | 1.7564(17) | C12 | C13 | 1.389(2) |
| C23 | C23[1] | 1.334(3) | C15 | C14 | 1.386(2) |
| C17 | C22 | 1.3985(19) | C19 | C20 | 1.384(2) |
| C17 | C18 | 1.3949(19) | C14 | C13 | 1.393(2) |
| C11 | C16 | 1.397(2) | C21 | C20 | 1.392(2) |
| C11 | C12 | 1.400(2) | | | |

[1]1-X,2-Y,-Z

**Table S5. Bond Angles for complex 2.**

| Atom | Atom | Atom | Angle/° | Atom | Atom | Atom | Angle/° |
|---|---|---|---|---|---|---|---|
| P1 | Ru1 | Cl2 | 83.734(12) | C18 | C17 | P1 | 120.45(11) |
| P1 | Ru1 | Cl1 | 85.914(13) | C18 | C17 | C22 | 119.48(13) |
| Cl1 | Ru1 | Cl2 | 87.967(13) | C16 | C11 | P1 | 120.35(11) |
| C6 | Ru1 | Cl2 | 90.21(4) | C16 | C11 | C12 | 119.50(13) |
| C6 | Ru1 | P1 | 157.50(5) | C12 | C11 | P1 | 119.59(11) |
| C6 | Ru1 | Cl1 | 115.60(5) | C5 | C6 | Ru1 | 71.93(9) |
| C6 | Ru1 | C4 | 66.90(6) | C5 | C6 | C1 | 120.58(14) |
| C6 | Ru1 | C5 | 36.43(6) | C1 | C6 | Ru1 | 70.86(8) |
| C4 | Ru1 | Cl2 | 154.84(4) | C5 | C4 | Ru1 | 69.67(8) |
| C4 | Ru1 | P1 | 121.41(4) | C5 | C4 | C8 | 119.62(13) |
| C4 | Ru1 | Cl1 | 92.61(4) | C3 | C4 | Ru1 | 69.69(8) |
| C5 | Ru1 | Cl2 | 117.39(4) | C3 | C4 | C5 | 117.34(13) |
| C5 | Ru1 | P1 | 158.61(4) | C3 | C4 | C8 | 123.02(14) |
| C5 | Ru1 | Cl1 | 91.23(4) | C8 | C4 | Ru1 | 133.27(10) |
| C5 | Ru1 | C4 | 37.46(5) | C6 | C5 | Ru1 | 71.64(9) |
| C3 | Ru1 | Cl2 | 152.41(4) | C6 | C5 | C4 | 121.73(14) |
| C3 | Ru1 | P1 | 96.53(4) | C4 | C5 | Ru1 | 72.87(8) |
| C3 | Ru1 | Cl1 | 119.61(4) | C4 | C3 | Ru1 | 73.66(8) |
| C3 | Ru1 | C6 | 79.02(6) | C4 | C3 | C2 | 121.05(14) |
| C3 | Ru1 | C4 | 36.65(5) | C2 | C3 | Ru1 | 70.18(8) |
| C3 | Ru1 | C5 | 66.58(6) | C15 | C16 | C11 | 120.09(14) |
| C1 | Ru1 | Cl2 | 88.23(4) | C6 | C1 | Ru1 | 71.37(9) |

| | | | | | | | |
|---|---|---|---|---|---|---|---|
| C1 | Ru1 | P1 | 120.11(5) | C6 | C1 | C7 | 121.21(16) |
| C1 | Ru1 | Cl1 | 153.07(5) | C2 | C1 | Ru1 | 70.46(8) |
| C1 | Ru1 | C6 | 37.77(6) | C2 | C1 | C6 | 118.23(14) |
| C1 | Ru1 | C4 | 79.94(5) | C2 | C1 | C7 | 120.55(16) |
| C1 | Ru1 | C5 | 67.13(6) | C7 | C1 | Ru1 | 128.44(11) |
| C1 | Ru1 | C3 | 67.68(6) | C3 | C2 | Ru1 | 72.07(8) |
| C2 | Ru1 | Cl2 | 114.67(4) | C1 | C2 | Ru1 | 72.25(9) |
| C2 | Ru1 | P1 | 95.73(4) | C1 | C2 | C3 | 121.04(14) |
| C2 | Ru1 | Cl1 | 157.35(4) | C21 | C22 | C17 | 119.92(14) |
| C2 | Ru1 | C6 | 67.18(6) | C19 | C18 | C17 | 120.22(14) |
| C2 | Ru1 | C4 | 67.23(5) | C4 | C8 | C10 | 113.04(14) |
| C2 | Ru1 | C5 | 79.01(6) | C4 | C8 | C9 | 110.13(14) |
| C2 | Ru1 | C3 | 37.74(5) | C10 | C8 | C9 | 108.74(15) |
| C2 | Ru1 | C1 | 37.29(6) | C13 | C12 | C11 | 119.92(14) |
| C23 | P1 | Ru1 | 116.33(5) | C14 | C15 | C16 | 120.32(15) |
| C23 | P1 | C17 | 101.12(6) | C20 | C19 | C18 | 120.11(14) |
| C23 | P1 | C11 | 104.72(6) | Cl4 | C24 | Cl3 | 111.48(9) |
| C17 | P1 | Ru1 | 117.76(5) | C15 | C14 | C13 | 119.83(14) |
| C11 | P1 | Ru1 | 110.26(4) | C22 | C21 | C20 | 120.31(14) |
| C11 | P1 | C17 | 105.26(6) | C12 | C13 | C14 | 120.31(14) |
| C23[1] | C23 | P1 | 125.69(15) | C19 | C20 | C21 | 119.95(14) |
| C22 | C17 | P1 | 120.05(10) | | | | |

[1]1-X,2-Y,-Z

**Table S6. Torsion Angles for complex 2.**

| A | B | C | D | Angle/° | A | B | C | D | Angle/° |
|---|---|---|---|---|---|---|---|---|---|
| Ru1 | P1 | C23 | C23[1] | 139.77(16) | C6 | C1 | C2 | Ru1 | 54.88(12) |
| Ru1 | P1 | C17 | C22 | -179.21(10) | C6 | C1 | C2 | C3 | -0.4(2) |
| Ru1 | P1 | C17 | C18 | 2.64(14) | C4 | C3 | C2 | Ru1 | -55.53(12) |
| Ru1 | P1 | C11 | C16 | 97.23(11) | C4 | C3 | C2 | C1 | -0.2(2) |
| Ru1 | P1 | C11 | C12 | -74.11(12) | C5 | C6 | C1 | Ru1 | 54.09(13) |
| Ru1 | C6 | C5 | C4 | 55.25(13) | C5 | C6 | C1 | C2 | -0.3(2) |
| Ru1 | C6 | C1 | C2 | -54.44(12) | C5 | C6 | C1 | C7 | 178.34(15) |

| | | | | | | | | | |
|---|---|---|---|---|---|---|---|---|---|
| Ru1 | C6 | C1 | C7 | 124.25(15) | C5 | C4 | C3 | Ru1 | -52.52(12) |
| Ru1 | C4 | C5 | C6 | -54.69(13) | C5 | C4 | C3 | C2 | 1.4(2) |
| Ru1 | C4 | C3 | C2 | 53.92(12) | C5 | C4 | C8 | C10 | -168.62(14) |
| Ru1 | C4 | C8 | C10 | 102.17(16) | C5 | C4 | C8 | C9 | -46.77(19) |
| Ru1 | C4 | C8 | C9 | -135.99(14) | C3 | C4 | C5 | Ru1 | 52.53(12) |
| Ru1 | C3 | C2 | C1 | 55.35(13) | C3 | C4 | C5 | C6 | -2.2(2) |
| Ru1 | C1 | C2 | C3 | -55.27(12) | C3 | C4 | C8 | C10 | 9.7(2) |
| P1 | C17 | C22 | C21 | -178.71(11) | C3 | C4 | C8 | C9 | 131.54(16) |
| P1 | C17 | C18 | C19 | 179.01(12) | C16 | C11 | C12 | C13 | 1.4(2) |
| P1 | C11 | C16 | C15 | -171.53(11) | C16 | C15 | C14 | C13 | 0.6(2) |
| P1 | C11 | C12 | C13 | 172.84(12) | C1 | C6 | C5 | Ru1 | -53.60(13) |
| C23 | P1 | C17 | C22 | 52.88(13) | C1 | C6 | C5 | C4 | 1.7(2) |
| C23 | P1 | C17 | C18 | -125.26(12) | C22 | C17 | C18 | C19 | 0.8(2) |
| C23 | P1 | C11 | C16 | -136.91(11) | C22 | C21 | C20 | C19 | 0.3(2) |
| C23 | P1 | C11 | C12 | 51.74(13) | C18 | C17 | C22 | C21 | -0.5(2) |
| C17 | P1 | C23 | C23$^{1}$ | -91.40(18) | C18 | C19 | C20 | C21 | 0.0(2) |
| C17 | P1 | C11 | C16 | -30.74(13) | C8 | C4 | C5 | Ru1 | -129.06(13) |
| C17 | P1 | C11 | C12 | 157.91(11) | C8 | C4 | C5 | C6 | 176.25(14) |
| C17 | C22 | C21 | C20 | 0.0(2) | C8 | C4 | C3 | Ru1 | 129.13(14) |
| C17 | C18 | C19 | C20 | -0.6(2) | C8 | C4 | C3 | C2 | -176.95(13) |
| C11 | P1 | C23 | C23$^{1}$ | 17.81(19) | C12 | C11 | C16 | C15 | -0.2(2) |
| C11 | P1 | C17 | C22 | -55.90(13) | C15 | C14 | C13 | C12 | 0.7(3) |
| C11 | P1 | C17 | C18 | 125.95(12) | C7 | C1 | C2 | Ru1 | -123.81(14) |
| C11 | C16 | C15 | C14 | -0.8(2) | C7 | C1 | C2 | C3 | -179.08(14) |
| C11 | C12 | C13 | C14 | -1.7(2) | | | | | |

$^{1}$1-X,2-Y,-Z

**Table S7. Hydrogen Atom Coordinates ($Å\times10^4$) and Isotropic Displacement Parameters ($Å^2\times10^3$) for complex 2.**

| Atom | *x* | *y* | *z* | U(eq) |
|---|---|---|---|---|
| H6 | 5956.45 | 8393.31 | 5150.63 | 26 |
| H5 | 3468.87 | 8875.01 | 6140.42 | 23 |
| H3 | 3909.69 | 6319.24 | 3801.14 | 19 |
| H16 | 4816.13 | 6118.91 | 906.48 | 19 |

| | | | | |
|---|---|---|---|---|
| H2 | 6426.83 | 5863.77 | 2767.07 | 23 |
| H22 | 3514.92 | 8627.13 | -338.77 | 19 |
| H18 | 1717.22 | 8692.02 | 3404.93 | 21 |
| H8 | 1182.87 | 8810.27 | 5677.34 | 27 |
| H12 | 7576.41 | 7881.28 | 136.86 | 20 |
| H15 | 6823.92 | 4182.65 | -72.31 | 25 |
| H19 | -404.47 | 8735.26 | 3372.82 | 27 |
| H24A | 3209.06 | 3265.22 | 1938.7 | 27 |
| H24B | 3571.21 | 2403.05 | 3046.61 | 27 |
| H14 | 9191.57 | 4103.88 | -987.15 | 29 |
| H21 | 1380.98 | 8689.43 | -357.42 | 24 |
| H13 | 9562.46 | 5962.56 | -901.92 | 26 |
| H10A | 1479.05 | 7053.44 | 4625.92 | 47 |
| H10B | 302 | 7159.57 | 6012.08 | 47 |
| H10C | 1944.09 | 6048.13 | 5624.64 | 47 |
| H20 | -574.73 | 8737.39 | 1495.94 | 25 |
| H7A | 8366.34 | 6644.76 | 2280.02 | 55 |
| H7B | 8653.22 | 5999.13 | 3484.43 | 55 |
| H7C | 8231.21 | 7535.43 | 3272.45 | 55 |
| H9A | 2033.21 | 6908.1 | 7406.14 | 54 |
| H9B | 405.1 | 8015.93 | 7693.7 | 54 |
| H9C | 1704.79 | 8423.88 | 7464.99 | 54 |
| H23 | 4065(19) | 10819(18) | 1072(17) | 17(4) |

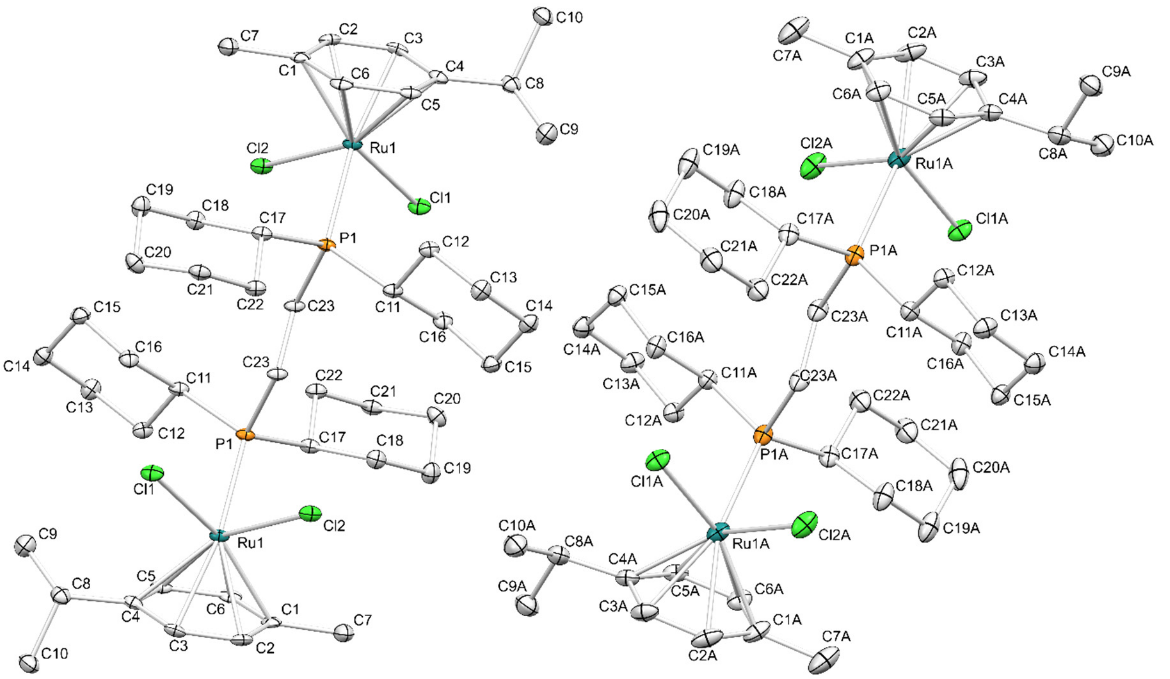


**Figure S69**. ORTEP representation of the two entities of **4** crystallised in the asymmetric unit, with complete atom labellings, in the solid state determined by single crystal X-ray diffraction analysis. Thermal ellipsoids represented at the 30% probability level. H atoms omitted for clarity.

**Table S8. Crystal data and structure refinement for complex 4.**

| | |
|---|---|
| Empirical formula | $C_{46}H_{76}Cl_4P_2Ru_2$ |
| Formula weight | 1034.94 |
| Temperature/K | 100.0(3) |
| Crystal system | monoclinic |
| Space group | $P2_1/c$ |
| a/Å | 21.3803(2) |
| b/Å | 15.64630(10) |
| c/Å | 13.87320(10) |
| α/° | 90 |
| β/° | 96.8390(10) |
| γ/° | 90 |
| Volume/Å$^3$ | 4607.88(6) |
| Z | 4 |
| $\rho_{calc}$g/cm$^3$ | 1.492 |

| | |
|---|---|
| $\mu/mm^{-1}$ | 8.325 |
| F(000) | 2152.0 |
| Crystal size/$mm^3$ | 0.12 × 0.101 × 0.048 |
| Radiation | Cu K$\alpha$ ($\lambda$ = 1.54184) |
| 2Θ range for data collection/° | 7.018 to 159.982 |
| Index ranges | -19 ≤ h ≤ 26, -19 ≤ k ≤ 19, -17 ≤ l ≤ 17 |
| Reflections collected | 80964 |
| Independent reflections | 9882 [$R_{int}$ = 0.0417, $R_{sigma}$ = 0.0231] |
| Data/restraints/parameters | 9882/0/493 |
| Goodness-of-fit on $F^2$ | 1.056 |
| Final R indexes [I>=2σ (I)] | $R_1$ = 0.0378, $wR_2$ = 0.0976 |
| Final R indexes [all data] | $R_1$ = 0.0401, $wR_2$ = 0.0989 |
| Largest diff. peak/hole / e $Å^{-3}$ | 1.09/-1.59 |

**Table S9. Fractional Atomic Coordinates (×$10^4$) and Equivalent Isotropic Displacement Parameters ($Å^2$×$10^3$) for complex 4. $U_{eq}$ is defined as 1/3 of the trace of the orthogonalised $U_{IJ}$ tensor.**

| **Atom** | ***x*** | ***y*** | ***z*** | **U(eq)** |
|---|---|---|---|---|
| Ru1 | 4404.3(2) | 7562.0(2) | 5128.8(2) | 16.76(7) |
| Ru1A | 9175.1(2) | 7439.1(2) | 5215.2(2) | 27.24(8) |
| Cl2 | 5251.3(4) | 7355.0(4) | 4133.0(5) | 23.17(14) |
| Cl1 | 3728.2(4) | 6876.5(4) | 3834.2(5) | 24.46(15) |
| P1 | 4646.0(3) | 6170.1(4) | 5788.0(5) | 16.47(14) |
| P1A | 9596.1(4) | 6100.9(5) | 5830.1(5) | 24.62(16) |
| Cl1A | 8559.6(4) | 6624.9(5) | 3970.6(5) | 32.86(17) |
| Cl2A | 9977.4(5) | 7433.4(6) | 4117.6(6) | 40.4(2) |
| C6 | 4546.2(15) | 8207.9(18) | 6554(2) | 22.1(6) |
| C11 | 4008.8(14) | 5533.9(18) | 6242(2) | 19.6(5) |
| C4 | 3599.9(14) | 8419.5(18) | 5377(2) | 19.5(5) |
| C23 | 4921.6(14) | 5460.6(17) | 4856.3(19) | 19.1(5) |
| C17 | 5283.0(13) | 6207.1(17) | 6831.7(19) | 18.3(5) |
| C3 | 3992.3(14) | 8842.6(17) | 4759(2) | 20.2(6) |
| C12 | 3684.9(14) | 6004.2(19) | 7018(2) | 21.1(6) |
| C21 | 5895.4(15) | 5544.9(19) | 8318(2) | 22.8(6) |

| | | | | |
|---|---|---|---|---|
| C5 | 3883.7(14) | 8124.8(17) | 6288.3(19) | 19.7(5) |
| C22 | 5345.5(14) | 5421.9(18) | 7512(2) | 21.0(6) |
| C2 | 4637.7(15) | 8928.8(17) | 5026(2) | 21.8(6) |
| C16 | 3510.5(14) | 5228(2) | 5429(2) | 23.5(6) |
| C13 | 3188.3(14) | 5437(2) | 7418(2) | 25.3(6) |
| C18 | 5913.0(14) | 6408(2) | 6451(2) | 22.7(6) |
| C14 | 2710.9(15) | 5089(2) | 6605(2) | 25.8(6) |
| C1 | 4933.6(14) | 8605.1(17) | 5937(2) | 21.4(6) |
| C12A | 8761.8(16) | 5679(2) | 7208(2) | 27.0(6) |
| C8 | 2899.6(15) | 8371(2) | 5052(2) | 25.1(6) |
| C11A | 9065.6(14) | 5329(2) | 6345(2) | 24.1(6) |
| C15 | 3045.6(15) | 4630(2) | 5843(2) | 27.3(6) |
| C4A | 8325.0(17) | 7991(2) | 5783(2) | 29.7(7) |
| C5A | 8801.0(17) | 7843(2) | 6562(2) | 28.6(7) |
| C16A | 8551.3(15) | 4986(2) | 5582(2) | 29.0(7) |
| C17A | 10248.8(15) | 6270(2) | 6822(2) | 29.0(7) |
| C20 | 6507.9(15) | 5698(2) | 7899(2) | 28.3(7) |
| C19 | 6452.8(15) | 6500(2) | 7270(2) | 27.5(6) |
| C6A | 9416.0(17) | 8183(2) | 6573(2) | 32.4(7) |
| C7 | 5629.9(16) | 8709(2) | 6206(3) | 32.2(7) |
| C14A | 7874.6(16) | 4609(2) | 6880(2) | 32.1(7) |
| C15A | 8176.0(16) | 4278(2) | 6015(2) | 29.6(7) |
| C13A | 8366.2(17) | 4991(2) | 7640(2) | 32.8(7) |
| C3A | 8484.1(19) | 8513(2) | 4999(3) | 36.6(8) |
| C23A | 9917.9(16) | 5471(2) | 4884(2) | 28.9(7) |
| C18A | 10769.8(17) | 6825(3) | 6464(3) | 39.2(9) |
| C10 | 2611.9(16) | 9250(2) | 5201(3) | 35.0(8) |
| C9 | 2558.6(17) | 7675(2) | 5552(3) | 36.2(8) |
| C22A | 10544.6(18) | 5490(3) | 7362(3) | 38.1(8) |
| C1A | 9573.5(19) | 8675(2) | 5786(3) | 39.0(9) |
| C2A | 9086(2) | 8846(2) | 5003(3) | 41.6(9) |
| C8A | 7662.5(18) | 7696(2) | 5854(3) | 36.1(8) |

| | | | | |
|---|---|---|---|---|
| C9A | 7385.0(19) | 8283(3) | 6582(3) | 42.8(9) |
| C10A | 7234(2) | 7668(3) | 4905(3) | 46.0(10) |
| C19A | 11227.1(18) | 7132(3) | 7333(3) | 45.4(10) |
| C21A | 11008.9(18) | 5785(3) | 8232(3) | 40.8(9) |
| C20A | 11511.4(18) | 6378(3) | 7913(3) | 49.1(11) |
| C7A | 10221(2) | 9040(3) | 5782(3) | 55.1(12) |

**Table S10. Anisotropic Displacement Parameters (Å$^2$×10$^3$) for complex 4. The Anisotropic displacement factor exponent takes the form: -2π$^2$[h$^2$a*$^2$U$_{11}$+2hka*b*U$_{12}$+…].**

| Atom | U$_{11}$ | U$_{22}$ | U$_{33}$ | U$_{23}$ | U$_{13}$ | U$_{12}$ |
|---|---|---|---|---|---|---|
| Ru1 | 27.10(12) | 12.23(11) | 11.04(11) | -0.67(7) | 2.58(8) | 1.22(7) |
| Ru1A | 36.97(15) | 29.05(14) | 15.55(12) | -0.36(8) | 2.55(9) | -10.22(10) |
| Cl2 | 35.7(4) | 17.6(3) | 17.8(3) | 1.1(2) | 9.7(3) | 0.6(3) |
| Cl1 | 36.5(4) | 20.5(3) | 15.4(3) | -3.4(2) | -1.4(3) | 1.2(3) |
| P1 | 25.4(3) | 12.2(3) | 12.4(3) | -0.8(2) | 4.4(2) | 1.4(2) |
| P1A | 25.0(4) | 32.9(4) | 16.4(3) | -5.7(3) | 4.6(3) | -4.9(3) |
| Cl1A | 41.9(4) | 35.4(4) | 20.1(3) | -3.6(3) | -1.1(3) | -9.0(3) |
| Cl2A | 49.2(5) | 51.8(5) | 21.9(4) | -1.6(3) | 11.8(3) | -19.5(4) |
| C6 | 37.8(16) | 14.8(13) | 12.7(12) | -6.0(10) | -0.4(11) | 3.0(11) |
| C11 | 28.3(14) | 14.7(13) | 16.5(12) | -0.9(10) | 5.4(11) | 0.6(11) |
| C4 | 26.1(14) | 15.1(13) | 17.5(13) | -3.9(10) | 3.0(11) | 5.3(10) |
| C23 | 30.4(15) | 13.6(13) | 14.1(12) | -2.0(10) | 5.2(10) | -0.2(11) |
| C17 | 28.1(14) | 13.9(12) | 13.2(12) | 0.5(10) | 3.5(10) | 2.1(10) |
| C3 | 34.2(15) | 11.5(12) | 15.3(12) | 1.5(10) | 4.1(11) | 7.4(11) |
| C12 | 28.7(15) | 18.8(13) | 16.3(13) | -1.1(11) | 4.9(11) | -1.7(11) |
| C21 | 34.2(16) | 17.4(13) | 16.6(13) | 3.5(11) | 2.5(11) | 3.5(11) |
| C5 | 35.0(15) | 12.8(12) | 12.5(12) | -2.8(10) | 7.8(11) | 1.6(11) |
| C22 | 31.0(15) | 17.0(13) | 15.3(12) | 1.3(10) | 4.0(11) | -0.1(11) |
| C2 | 35.5(16) | 9.0(12) | 21.9(14) | -1.4(10) | 7.8(12) | -1.8(11) |
| C16 | 26.9(15) | 24.3(15) | 19.6(13) | -5.5(11) | 3.9(11) | 0.6(12) |
| C13 | 27.8(15) | 29.9(16) | 18.3(13) | 1.9(12) | 3.9(11) | -3.4(12) |
| C18 | 27.0(15) | 23.9(15) | 17.8(13) | 1.7(11) | 5.0(11) | 0.5(11) |
| C14 | 27.4(15) | 26.9(15) | 23.1(14) | 3.0(12) | 2.9(12) | -3.8(12) |

| | | | | | | |
|---|---|---|---|---|---|---|
| C1 | 29.1(15) | 10.0(12) | 24.0(14) | -8.1(10) | -1.1(11) | -0.4(10) |
| C12A | 32.7(16) | 27.3(16) | 22.4(14) | -1.2(12) | 9.1(12) | -2.7(13) |
| C8 | 27.9(15) | 25.2(15) | 22.1(14) | -5.3(12) | 2.6(12) | 3.0(12) |
| C11A | 25.5(14) | 26.8(15) | 20.3(14) | -0.4(12) | 4.1(11) | -1.3(12) |
| C15 | 30.2(16) | 20.7(14) | 30.1(16) | -3.5(12) | -0.4(12) | -2.2(12) |
| C4A | 40.0(18) | 18.7(14) | 29.9(16) | -0.5(12) | 2.8(14) | 0.5(13) |
| C5A | 46.5(19) | 20.1(15) | 19.8(14) | 0.0(12) | 6.3(13) | -0.3(13) |
| C16A | 27.1(15) | 37.8(18) | 22.2(14) | -1.1(13) | 3.5(12) | -5.7(13) |
| C17A | 28.9(16) | 38.3(18) | 20.2(14) | -9.1(13) | 4.5(12) | -1.7(13) |
| C20 | 29.8(16) | 30.5(17) | 24.4(15) | 3.4(13) | 2.7(12) | 7.7(13) |
| C19 | 25.4(15) | 31.6(17) | 25.6(15) | 5.1(13) | 3.6(12) | -1.5(12) |
| C6A | 43.7(19) | 33.6(18) | 19.5(14) | -4.8(13) | 1.2(13) | -5.1(15) |
| C7 | 30.7(17) | 23.3(16) | 41.3(19) | -5.6(14) | -1.3(14) | 1.8(13) |
| C14A | 33.3(17) | 32.2(17) | 32.6(17) | 2.6(14) | 11.6(14) | -4.7(14) |
| C15A | 29.9(16) | 31.1(17) | 27.8(16) | -3.1(13) | 3.3(13) | -4.8(13) |
| C13A | 42.4(19) | 31.3(17) | 26.7(16) | 0.1(14) | 11.8(14) | -7.1(15) |
| C3A | 55(2) | 24.3(16) | 27.5(16) | 3.9(13) | -6.1(15) | -2.5(15) |
| C23A | 30.3(16) | 38.3(19) | 19.1(14) | -9.8(13) | 6.8(12) | -7.3(14) |
| C18A | 31.6(18) | 60(3) | 26.0(16) | -9.6(16) | 5.0(14) | -12.4(17) |
| C10 | 31.2(17) | 29.3(17) | 43(2) | -9.0(15) | -0.4(15) | 8.8(14) |
| C9 | 32.5(18) | 35.0(19) | 41(2) | -1.7(16) | 4.4(15) | -3.7(14) |
| C22A | 36.8(19) | 42(2) | 32.8(18) | -10.8(15) | -6.2(15) | 3.1(15) |
| C1A | 52(2) | 35.6(19) | 29.5(17) | -7.1(15) | 4.0(15) | -20.0(17) |
| C2A | 70(3) | 28.5(18) | 25.7(17) | 1.8(14) | 3.2(17) | -17.3(17) |
| C8A | 38.2(19) | 25.0(16) | 45(2) | 0.3(15) | 3.2(16) | 3.2(14) |
| C9A | 43(2) | 37(2) | 49(2) | -3.9(17) | 4.5(17) | 9.0(16) |
| C10A | 43(2) | 41(2) | 51(2) | -9.6(19) | -3.8(18) | 0.2(17) |
| C19A | 35.2(19) | 70(3) | 32.0(19) | -11.0(19) | 6.5(15) | -18.9(19) |
| C21A | 39(2) | 49(2) | 31.8(18) | -12.1(16) | -7.3(15) | 6.3(17) |
| C20A | 28.6(18) | 81(3) | 37(2) | -22(2) | -1.8(15) | 0.4(19) |
| C7A | 68(3) | 57(3) | 41(2) | -10(2) | 10(2) | -35(2) |

**Table S11. Bond Lengths for complex 4.**

| Atom | Atom | Length/Å | Atom | Atom | Length/Å |
|---|---|---|---|---|---|
| Ru1 | Cl2 | 2.4266(7) | C12 | C13 | 1.537(4) |
| Ru1 | Cl1 | 2.4181(7) | C21 | C22 | 1.535(4) |
| Ru1 | P1 | 2.3946(7) | C21 | C20 | 1.514(4) |
| Ru1 | C6 | 2.210(3) | C2 | C1 | 1.436(4) |
| Ru1 | C4 | 2.240(3) | C16 | C15 | 1.527(4) |
| Ru1 | C3 | 2.224(3) | C13 | C14 | 1.529(4) |
| Ru1 | C5 | 2.242(3) | C18 | C19 | 1.527(4) |
| Ru1 | C2 | 2.204(3) | C14 | C15 | 1.525(4) |
| Ru1 | C1 | 2.214(3) | C1 | C7 | 1.500(4) |
| Ru1A | P1A | 2.3955(9) | C12A | C11A | 1.531(4) |
| Ru1A | Cl1A | 2.4072(8) | C12A | C13A | 1.534(4) |
| Ru1A | Cl2A | 2.4256(9) | C8 | C10 | 1.531(4) |
| Ru1A | C4A | 2.239(4) | C8 | C9 | 1.523(5) |
| Ru1A | C5A | 2.211(3) | C11A | C16A | 1.530(4) |
| Ru1A | C6A | 2.222(3) | C4A | C5A | 1.413(5) |
| Ru1A | C3A | 2.234(4) | C4A | C3A | 1.433(5) |
| Ru1A | C1A | 2.220(3) | C4A | C8A | 1.504(5) |
| Ru1A | C2A | 2.226(4) | C5A | C6A | 1.417(5) |
| P1 | C11 | 1.857(3) | C16A | C15A | 1.532(5) |
| P1 | C23 | 1.852(3) | C17A | C18A | 1.541(5) |
| P1 | C17 | 1.867(3) | C17A | C22A | 1.529(5) |
| P1A | C11A | 1.857(3) | C20 | C19 | 1.525(4) |
| P1A | C17A | 1.859(3) | C6A | C1A | 1.409(5) |
| P1A | C23A | 1.839(3) | C14A | C15A | 1.518(5) |
| C6 | C5 | 1.426(4) | C14A | C13A | 1.521(5) |
| C6 | C1 | 1.405(4) | C3A | C2A | 1.388(6) |
| C11 | C12 | 1.536(4) | C23A | C23A[2] | 1.541(7) |
| C11 | C16 | 1.533(4) | C18A | C19A | 1.536(5) |
| C4 | C3 | 1.431(4) | C22A | C21A | 1.539(5) |
| C4 | C5 | 1.413(4) | C1A | C2A | 1.438(6) |

| | | | | | | |
|---|---|---|---|---|---|---|
| C4 | C8 | 1.513(4) | | C1A | C7A | 1.497(5) |
| C23 | C23[1] | 1.522(5) | | C8A | C9A | 1.537(5) |
| C17 | C22 | 1.545(4) | | C8A | C10A | 1.512(6) |
| C17 | C18 | 1.537(4) | | C19A | C20A | 1.515(7) |
| C3 | C2 | 1.392(4) | | C21A | C20A | 1.524(6) |

[1]1-X,1-Y,1-Z; [2]2-X,1-Y,1-Z

**Table S12. Bond Angles for complex 4.**

| Atom | Atom | Atom | Angle/° | Atom | Atom | Atom | Angle/° |
|---|---|---|---|---|---|---|---|
| Cl1 | Ru1 | Cl2 | 86.48(3) | C1 | C6 | C5 | 121.4(3) |
| P1 | Ru1 | Cl2 | 87.38(2) | C12 | C11 | P1 | 113.04(19) |
| P1 | Ru1 | Cl1 | 87.88(2) | C16 | C11 | P1 | 113.1(2) |
| C6 | Ru1 | Cl2 | 122.58(9) | C16 | C11 | C12 | 109.5(2) |
| C6 | Ru1 | Cl1 | 150.91(9) | C3 | C4 | Ru1 | 70.69(15) |
| C6 | Ru1 | P1 | 94.23(8) | C3 | C4 | C8 | 118.3(3) |
| C6 | Ru1 | C4 | 67.33(11) | C5 | C4 | Ru1 | 71.72(16) |
| C6 | Ru1 | C3 | 78.63(10) | C5 | C4 | C3 | 117.8(3) |
| C6 | Ru1 | C5 | 37.36(11) | C5 | C4 | C8 | 123.7(3) |
| C6 | Ru1 | C1 | 37.04(11) | C8 | C4 | Ru1 | 132.4(2) |
| C4 | Ru1 | Cl2 | 143.72(8) | C23[1] | C23 | P1 | 117.6(3) |
| C4 | Ru1 | Cl1 | 88.73(7) | C22 | C17 | P1 | 116.8(2) |
| C4 | Ru1 | P1 | 128.38(8) | C18 | C17 | P1 | 109.14(18) |
| C4 | Ru1 | C5 | 36.75(10) | C18 | C17 | C22 | 110.9(2) |
| C3 | Ru1 | Cl2 | 106.91(8) | C4 | C3 | Ru1 | 71.91(15) |
| C3 | Ru1 | Cl1 | 92.46(8) | C2 | C3 | Ru1 | 70.91(16) |
| C3 | Ru1 | P1 | 165.71(8) | C2 | C3 | C4 | 121.2(3) |
| C3 | Ru1 | C4 | 37.40(10) | C11 | C12 | C13 | 111.4(2) |
| C3 | Ru1 | C5 | 66.10(10) | C20 | C21 | C22 | 111.3(2) |
| C5 | Ru1 | Cl2 | 158.22(8) | C6 | C5 | Ru1 | 70.07(16) |
| C5 | Ru1 | Cl1 | 113.77(8) | C4 | C5 | Ru1 | 71.53(15) |
| C5 | Ru1 | P1 | 100.73(7) | C4 | C5 | C6 | 120.6(3) |
| C2 | Ru1 | Cl2 | 84.42(8) | C21 | C22 | C17 | 110.4(2) |
| C2 | Ru1 | Cl1 | 120.16(8) | C3 | C2 | Ru1 | 72.45(16) |

| | | | | | | | |
|---|---|---|---|---|---|---|---|
| C2 | Ru1 | P1 | 150.08(8) | C3 | C2 | C1 | 121.3(3) |
| C2 | Ru1 | C6 | 66.80(11) | C1 | C2 | Ru1 | 71.38(15) |
| C2 | Ru1 | C4 | 67.21(11) | C15 | C16 | C11 | 109.9(2) |
| C2 | Ru1 | C3 | 36.64(11) | C14 | C13 | C12 | 111.6(2) |
| C2 | Ru1 | C5 | 78.51(11) | C19 | C18 | C17 | 112.3(2) |
| C2 | Ru1 | C1 | 37.95(11) | C15 | C14 | C13 | 110.6(3) |
| C1 | Ru1 | Cl2 | 90.91(8) | C6 | C1 | Ru1 | 71.32(16) |
| C1 | Ru1 | Cl1 | 158.08(8) | C6 | C1 | C2 | 117.5(3) |
| C1 | Ru1 | P1 | 113.76(8) | C6 | C1 | C7 | 122.4(3) |
| C1 | Ru1 | C4 | 80.57(11) | C2 | C1 | Ru1 | 70.68(15) |
| C1 | Ru1 | C3 | 67.49(11) | C2 | C1 | C7 | 120.0(3) |
| C1 | Ru1 | C5 | 67.32(11) | C7 | C1 | Ru1 | 129.7(2) |
| P1A | Ru1A | Cl1A | 86.74(3) | C11A | C12A | C13A | 111.1(3) |
| P1A | Ru1A | Cl2A | 87.51(3) | C4 | C8 | C10 | 108.3(3) |
| Cl1A | Ru1A | Cl2A | 85.11(3) | C4 | C8 | C9 | 114.2(3) |
| C4A | Ru1A | P1A | 119.89(9) | C9 | C8 | C10 | 111.0(3) |
| C4A | Ru1A | Cl1A | 93.40(9) | C12A | C11A | P1A | 113.8(2) |
| C4A | Ru1A | Cl2A | 152.48(9) | C16A | C11A | P1A | 112.6(2) |
| C5A | Ru1A | P1A | 96.16(9) | C16A | C11A | C12A | 109.3(3) |
| C5A | Ru1A | Cl1A | 122.39(9) | C14 | C15 | C16 | 110.6(3) |
| C5A | Ru1A | Cl2A | 152.35(9) | C5A | C4A | Ru1A | 70.42(19) |
| C5A | Ru1A | C4A | 37.02(12) | C5A | C4A | C3A | 117.4(3) |
| C5A | Ru1A | C6A | 37.29(13) | C5A | C4A | C8A | 119.9(3) |
| C5A | Ru1A | C3A | 66.32(13) | C3A | C4A | Ru1A | 71.1(2) |
| C5A | Ru1A | C1A | 67.16(13) | C3A | C4A | C8A | 122.3(3) |
| C5A | Ru1A | C2A | 78.20(13) | C8A | C4A | Ru1A | 135.6(2) |
| C6A | Ru1A | P1A | 96.72(10) | C4A | C5A | Ru1A | 72.56(19) |
| C6A | Ru1A | Cl1A | 159.56(10) | C4A | C5A | C6A | 121.9(3) |
| C6A | Ru1A | Cl2A | 115.09(10) | C6A | C5A | Ru1A | 71.80(19) |
| C6A | Ru1A | C4A | 67.38(13) | C11A | C16A | C15A | 110.4(3) |
| C6A | Ru1A | C3A | 78.60(13) | C18A | C17A | P1A | 110.4(2) |
| C6A | Ru1A | C2A | 66.52(13) | C22A | C17A | P1A | 118.6(2) |

| | | | | | | | |
|---|---|---|---|---|---|---|---|
| C3A | Ru1A | P1A | 156.87(10) | C22A | C17A | C18A | 109.6(3) |
| C3A | Ru1A | Cl1A | 90.31(9) | C21 | C20 | C19 | 109.8(3) |
| C3A | Ru1A | Cl2A | 115.11(10) | C20 | C19 | C18 | 110.4(3) |
| C3A | Ru1A | C4A | 37.37(13) | C5A | C6A | Ru1A | 70.92(18) |
| C1A | Ru1A | P1A | 121.49(11) | C1A | C6A | Ru1A | 71.43(19) |
| C1A | Ru1A | Cl1A | 150.48(11) | C1A | C6A | C5A | 120.3(3) |
| C1A | Ru1A | Cl2A | 87.53(10) | C15A | C14A | C13A | 111.0(3) |
| C1A | Ru1A | C4A | 80.31(14) | C14A | C15A | C16A | 110.9(3) |
| C1A | Ru1A | C6A | 36.98(13) | C14A | C13A | C12A | 111.9(3) |
| C1A | Ru1A | C3A | 67.19(15) | C4A | C3A | Ru1A | 71.5(2) |
| C1A | Ru1A | C2A | 37.75(14) | C2A | C3A | Ru1A | 71.5(2) |
| C2A | Ru1A | P1A | 159.08(11) | C2A | C3A | C4A | 121.0(3) |
| C2A | Ru1A | Cl1A | 113.41(10) | C23A[2] | C23A | P1A | 117.3(3) |
| C2A | Ru1A | Cl2A | 88.64(11) | C19A | C18A | C17A | 109.9(3) |
| C2A | Ru1A | C4A | 66.71(14) | C17A | C22A | C21A | 109.6(3) |
| C2A | Ru1A | C3A | 36.25(15) | C6A | C1A | Ru1A | 71.6(2) |
| C11 | P1 | Ru1 | 118.91(9) | C6A | C1A | C2A | 117.9(3) |
| C11 | P1 | C17 | 104.52(13) | C6A | C1A | C7A | 121.1(4) |
| C23 | P1 | Ru1 | 110.53(9) | C2A | C1A | Ru1A | 71.3(2) |
| C23 | P1 | C11 | 102.86(13) | C2A | C1A | C7A | 120.9(4) |
| C23 | P1 | C17 | 107.25(13) | C7A | C1A | Ru1A | 130.2(3) |
| C17 | P1 | Ru1 | 111.86(9) | C3A | C2A | Ru1A | 72.2(2) |
| C11A | P1A | Ru1A | 118.98(10) | C3A | C2A | C1A | 121.4(3) |
| C11A | P1A | C17A | 104.30(15) | C1A | C2A | Ru1A | 70.9(2) |
| C17A | P1A | Ru1A | 110.89(12) | C4A | C8A | C9A | 107.4(3) |
| C23A | P1A | Ru1A | 111.75(11) | C4A | C8A | C10A | 115.4(3) |
| C23A | P1A | C11A | 102.93(15) | C10A | C8A | C9A | 110.4(3) |
| C23A | P1A | C17A | 107.04(15) | C20A | C19A | C18A | 110.6(4) |
| C5 | C6 | Ru1 | 72.57(15) | C20A | C21A | C22A | 111.5(3) |
| C1 | C6 | Ru1 | 71.63(16) | C19A | C20A | C21A | 112.1(3) |

[1]1-X,1-Y,1-Z; [2]2-X,1-Y,1-Z

**Table S13. Torsion Angles for complex 4.**

| A | B | C | D | Angle/° | A | B | C | D | Angle/° |
|---|---|---|---|---|---|---|---|---|---|
| Ru1 | P1 | C11 | C12 | -55.9(2) | C5 | C6 | C1 | C7 | 179.3(3) |
| Ru1 | P1 | C11 | C16 | 69.2(2) | C5 | C4 | C3 | Ru1 | 55.9(2) |
| Ru1 | P1 | C23 | C23[1] | -178.2(3) | C5 | C4 | C3 | C2 | 2.8(4) |
| Ru1 | P1 | C17 | C22 | 161.92(18) | C5 | C4 | C8 | C10 | 99.8(3) |
| Ru1 | P1 | C17 | C18 | -71.3(2) | C5 | C4 | C8 | C9 | -24.4(4) |
| Ru1 | C6 | C5 | C4 | -52.7(2) | C22 | C17 | C18 | C19 | -53.2(3) |
| Ru1 | C6 | C1 | C2 | 55.2(2) | C22 | C21 | C20 | C19 | 60.2(3) |
| Ru1 | C6 | C1 | C7 | -125.7(3) | C16 | C11 | C12 | C13 | 56.4(3) |
| Ru1 | C4 | C3 | C2 | -53.1(2) | C13 | C14 | C15 | C16 | -57.4(3) |
| Ru1 | C4 | C5 | C6 | 52.0(2) | C18 | C17 | C22 | C21 | 53.1(3) |
| Ru1 | C4 | C8 | C10 | -164.6(2) | C1 | C6 | C5 | Ru1 | 54.6(2) |
| Ru1 | C4 | C8 | C9 | 71.2(4) | C1 | C6 | C5 | C4 | 1.9(4) |
| Ru1 | C3 | C2 | C1 | -54.3(2) | C12A | C11A | C16A | C15A | -59.0(4) |
| Ru1 | C2 | C1 | C6 | -55.6(2) | C8 | C4 | C3 | Ru1 | -128.5(2) |
| Ru1 | C2 | C1 | C7 | 125.3(3) | C8 | C4 | C3 | C2 | 178.3(3) |
| Ru1A | P1A | C11A | C12A | -59.5(2) | C8 | C4 | C5 | Ru1 | 129.3(3) |
| Ru1A | P1A | C11A | C16A | 65.6(2) | C8 | C4 | C5 | C6 | -178.7(3) |
| Ru1A | P1A | C17A | C18A | -57.0(3) | C11A | P1A | C17A | C18A | 173.8(3) |
| Ru1A | P1A | C17A | C22A | 175.3(2) | C11A | P1A | C17A | C22A | 46.1(3) |
| Ru1A | P1A | C23A | C23A[2] | -168.0(3) | C11A | P1A | C23A | C23A[2] | -39.2(4) |
| Ru1A | C4A | C5A | C6A | 54.4(3) | C11A | C12A | C13A | C14A | -55.1(4) |
| Ru1A | C4A | C3A | C2A | -53.8(3) | C11A | C16A | C15A | C14A | 58.9(4) |
| Ru1A | C4A | C8A | C9A | 164.0(3) | C4A | C5A | C6A | Ru1A | -54.8(3) |
| Ru1A | C4A | C8A | C10A | -72.3(4) | C4A | C5A | C6A | C1A | -1.1(5) |
| Ru1A | C5A | C6A | C1A | 53.7(3) | C4A | C3A | C2A | Ru1A | 53.8(3) |
| Ru1A | C6A | C1A | C2A | 56.1(3) | C4A | C3A | C2A | C1A | 0.6(6) |
| Ru1A | C6A | C1A | C7A | -126.5(4) | C5A | C4A | C3A | Ru1A | 54.8(3) |
| Ru1A | C3A | C2A | C1A | -53.2(3) | C5A | C4A | C3A | C2A | 1.0(5) |
| Ru1A | C1A | C2A | C3A | 53.8(3) | C5A | C4A | C8A | C9A | 72.1(4) |
| P1 | C11 | C12 | C13 | -176.6(2) | C5A | C4A | C8A | C10A | -164.3(3) |

| | | | | | | | | | |
|---|---|---|---|---|---|---|---|---|---|
| P1 | C11 | C16 | C15 | 173.7(2) | C5A | C6A | C1A | Ru1A | -53.5(3) |
| P1 | C17 | C22 | C21 | 178.97(19) | C5A | C6A | C1A | C2A | 2.6(5) |
| P1 | C17 | C18 | C19 | 176.7(2) | C5A | C6A | C1A | C7A | -179.9(4) |
| P1A | C11A | C16A | C15A | 173.5(2) | C17A | P1A | C11A | C12A | 64.7(3) |
| P1A | C17A | C18A | C19A | 167.0(3) | C17A | P1A | C11A | C16A | -170.2(2) |
| P1A | C17A | C22A | C21A | -172.5(2) | C17A | P1A | C23A | $C23A^2$ | 70.4(4) |
| C11 | P1 | C23 | $C23^1$ | -50.3(3) | C17A | C18A | C19A | C20A | 57.7(4) |
| C11 | P1 | C17 | C22 | 32.0(2) | C17A | C22A | C21A | C20A | -56.3(4) |
| C11 | P1 | C17 | C18 | 158.79(19) | C20 | C21 | C22 | C17 | -57.4(3) |
| C11 | C12 | C13 | C14 | -54.3(3) | C6A | C1A | C2A | Ru1A | -56.2(3) |
| C11 | C16 | C15 | C14 | 60.4(3) | C6A | C1A | C2A | C3A | -2.4(6) |
| C4 | C3 | C2 | Ru1 | 53.6(2) | C15A | C14A | C13A | C12A | 54.0(4) |
| C4 | C3 | C2 | C1 | -0.7(4) | C13A | C12A | C11A | P1A | -176.2(2) |
| C23 | P1 | C11 | C12 | -178.4(2) | C13A | C12A | C11A | C16A | 57.0(4) |
| C23 | P1 | C11 | C16 | -53.3(2) | C13A | C14A | C15A | C16A | -55.8(4) |
| C23 | P1 | C17 | C22 | -76.7(2) | C3A | C4A | C5A | Ru1A | -55.2(3) |
| C23 | P1 | C17 | C18 | 50.1(2) | C3A | C4A | C5A | C6A | -0.8(5) |
| C17 | P1 | C11 | C12 | 69.7(2) | C3A | C4A | C8A | C9A | -100.3(4) |
| C17 | P1 | C11 | C16 | -165.2(2) | C3A | C4A | C8A | C10A | 23.4(5) |
| C17 | P1 | C23 | $C23^1$ | 59.6(3) | C23A | P1A | C11A | C12A | 176.3(2) |
| C17 | C18 | C19 | C20 | 56.0(3) | C23A | P1A | C11A | C16A | -58.6(3) |
| C3 | C4 | C5 | Ru1 | -55.4(2) | C23A | P1A | C17A | C18A | 65.1(3) |
| C3 | C4 | C5 | C6 | -3.3(4) | C23A | P1A | C17A | C22A | -62.6(3) |
| C3 | C4 | C8 | C10 | -75.5(3) | C18A | C17A | C22A | C21A | 59.4(4) |
| C3 | C4 | C8 | C9 | 160.3(3) | C18A | C19A | C20A | C21A | -54.8(4) |
| C3 | C2 | C1 | Ru1 | 54.8(2) | C22A | C17A | C18A | C19A | -60.5(4) |
| C3 | C2 | C1 | C6 | -0.8(4) | C22A | C21A | C20A | C19A | 54.4(4) |
| C3 | C2 | C1 | C7 | -179.9(3) | C8A | C4A | C5A | Ru1A | 132.1(3) |
| C12 | C11 | C16 | C15 | -59.3(3) | C8A | C4A | C5A | C6A | -173.5(3) |
| C12 | C13 | C14 | C15 | 54.2(3) | C8A | C4A | C3A | Ru1A | -132.6(3) |
| C21 | C20 | C19 | C18 | -58.7(3) | C8A | C4A | C3A | C2A | 173.5(3) |
| C5 | C6 | C1 | Ru1 | -55.0(2) | C7A | C1A | C2A | Ru1A | 126.3(4) |

| | | | | | | | | | |
|---|---|---|---|---|---|---|---|---|---|
| C5 | C6 | C1 | C2 | 0.2(4) | C7A | C1A | C2A | C3A | -179.9(4) |

[1]1-X,1-Y,1-Z; [2]2-X,1-Y,1-Z

**Table S14. Hydrogen Atom Coordinates (Å×10⁴) and Isotropic Displacement Parameters (Å²×10³) for complex 4.**

| Atom | *x* | *y* | *z* | U(eq) |
|---|---|---|---|---|
| H6 | 4729.42 | 7989.78 | 7162.39 | 26 |
| H11 | 4210.11 | 5011.36 | 6556.8 | 24 |
| H23A | 4592.76 | 5451.44 | 4290.09 | 23 |
| H23B | 5302.42 | 5721.82 | 4638.16 | 23 |
| H17 | 5186.72 | 6705.87 | 7239.98 | 22 |
| H3 | 3809 | 9068.76 | 4155.03 | 24 |
| H12A | 3480.17 | 6528.54 | 6733.19 | 25 |
| H12B | 4005.5 | 6177.17 | 7557.41 | 25 |
| H21A | 5806.02 | 6038.24 | 8726.52 | 27 |
| H21B | 5936.98 | 5030.1 | 8735.13 | 27 |
| H5 | 3630.99 | 7869.04 | 6727.82 | 24 |
| H22A | 4949.42 | 5342.18 | 7805.05 | 25 |
| H22B | 5418.04 | 4902.86 | 7132.42 | 25 |
| H2 | 4887.26 | 9207.08 | 4598.33 | 26 |
| H16A | 3282.29 | 5726.26 | 5119.43 | 28 |
| H16B | 3717.91 | 4924.44 | 4927.26 | 28 |
| H13A | 3401.02 | 4953.13 | 7782.44 | 30 |
| H13B | 2964.41 | 5773.69 | 7874.56 | 30 |
| H18A | 6014.31 | 5944.33 | 6009.15 | 27 |
| H18B | 5870.78 | 6945.48 | 6071.77 | 27 |
| H14A | 2421.73 | 4686.86 | 6880.2 | 31 |
| H14B | 2456.92 | 5566.12 | 6296.4 | 31 |
| H12C | 8489.86 | 6172.05 | 6993.94 | 32 |
| H12D | 9094.8 | 5882.41 | 7713.23 | 32 |
| H8 | 2842.68 | 8247.12 | 4339.42 | 30 |
| H11A | 9330.51 | 4829.77 | 6588.31 | 29 |
| H15A | 2730.68 | 4425.01 | 5312.48 | 33 |
| H15B | 3273.71 | 4126.69 | 6140.8 | 33 |

| | | | | |
|---|---|---|---|---|
| H5A | 8706.01 | 7504.99 | 7094.9 | 34 |
| H16C | 8745.84 | 4756.48 | 5022.4 | 35 |
| H16D | 8264.03 | 5456.45 | 5344.28 | 35 |
| H17A | 10066.2 | 6620.61 | 7322.68 | 35 |
| H20A | 6605.14 | 5199.75 | 7502.23 | 34 |
| H20B | 6855.57 | 5766.79 | 8431.77 | 34 |
| H19A | 6852.61 | 6595.45 | 6992.11 | 33 |
| H19B | 6375.93 | 7001.82 | 7674.78 | 33 |
| H6A | 9723.39 | 8079.98 | 7113.12 | 39 |
| H7A | 5762.92 | 8386.75 | 6800.9 | 48 |
| H7B | 5729.08 | 9315.54 | 6314.34 | 48 |
| H7C | 5852.71 | 8492.82 | 5678.31 | 48 |
| H14C | 7656.19 | 4133.36 | 7171.6 | 38 |
| H14D | 7556.89 | 5048.9 | 6658.94 | 38 |
| H15C | 7844.26 | 4064.68 | 5514.62 | 36 |
| H15D | 8460.02 | 3795.85 | 6222.95 | 36 |
| H13C | 8647.6 | 4531.91 | 7926.97 | 39 |
| H13D | 8152.86 | 5246.48 | 8166.73 | 39 |
| H3A | 8172.79 | 8632.82 | 4469 | 44 |
| H23C | 9608.81 | 5486.45 | 4293.15 | 35 |
| H23D | 10304.38 | 5760.42 | 4723.99 | 35 |
| H18C | 11002.07 | 6488.66 | 6017.92 | 47 |
| H18D | 10578.12 | 7324.71 | 6102.58 | 47 |
| H10A | 2165.61 | 9245.68 | 4938.59 | 53 |
| H10B | 2834.34 | 9684.3 | 4864.1 | 53 |
| H10C | 2651.57 | 9381.4 | 5896.28 | 53 |
| H9A | 2593.38 | 7791.79 | 6250.71 | 54 |
| H9B | 2749.7 | 7119.09 | 5444.02 | 54 |
| H9C | 2113.3 | 7666.07 | 5284.59 | 54 |
| H22C | 10769.49 | 5140.97 | 6917.16 | 46 |
| H22D | 10210.8 | 5131.78 | 7593.12 | 46 |
| H2A | 9177.83 | 9193.84 | 4476.5 | 50 |

| | | | | |
|---|---|---|---|---|
| H8A | 7685.66 | 7104.88 | 6130.82 | 43 |
| H9AA | 7400.79 | 8877.29 | 6363.32 | 64 |
| H9AB | 7630.67 | 8225.43 | 7221.87 | 64 |
| H9AC | 6946.53 | 8120.98 | 6626.3 | 64 |
| H10D | 6831.59 | 7405.85 | 5010.56 | 69 |
| H10E | 7433.13 | 7328.52 | 4431.14 | 69 |
| H10F | 7160.45 | 8250.06 | 4657.46 | 69 |
| H19C | 11567.12 | 7475.71 | 7097.22 | 54 |
| H19D | 10999.56 | 7501.44 | 7754.44 | 54 |
| H21C | 10774.46 | 6086.3 | 8703.3 | 49 |
| H21D | 11214.42 | 5278.7 | 8560.69 | 49 |
| H20C | 11779.9 | 6590.09 | 8494.83 | 59 |
| H20D | 11782.74 | 6051.78 | 7514.17 | 59 |
| H7AA | 10363.25 | 8946.02 | 5144.24 | 83 |
| H7AB | 10511.03 | 8757.31 | 6282.32 | 83 |
| H7AC | 10211.2 | 9654.12 | 5915.57 | 83 |

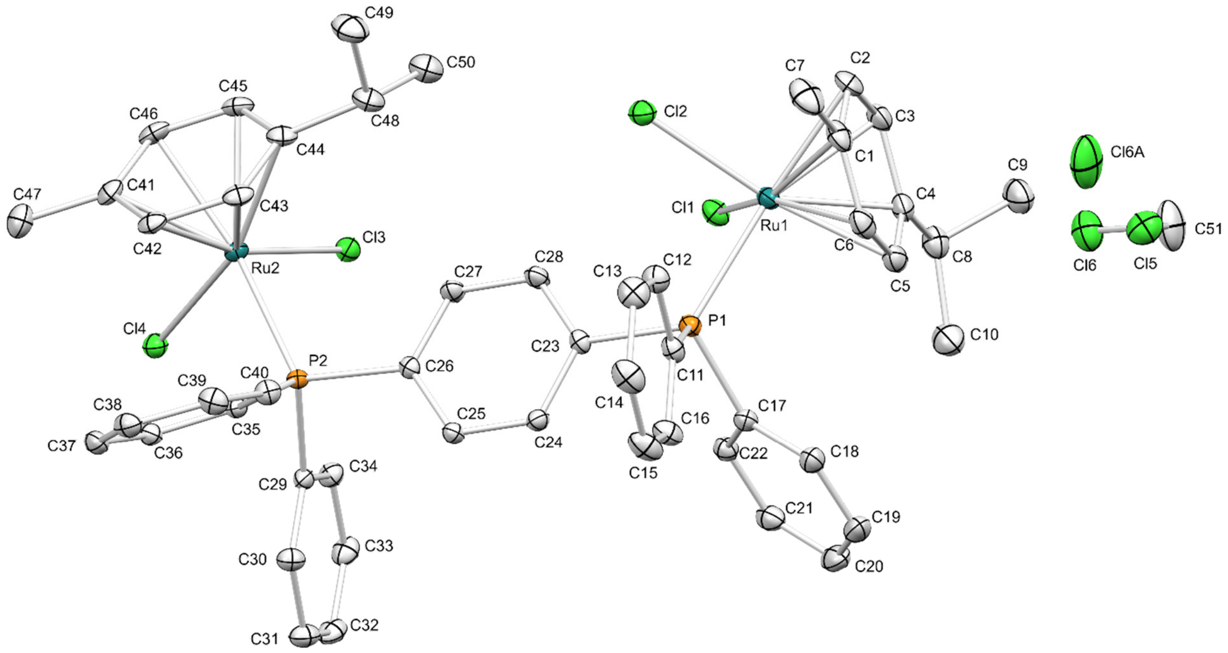


**Figure S70**. ORTEP representation of **5·CH2Cl2**, with complete atom labellings, in the solid state determined by single crystal X-ray diffraction analysis. Thermal ellipsoids represented at the 30% probability level. H atoms omitted for clarity. Cl6A is a disordered equivalent of Cl6.

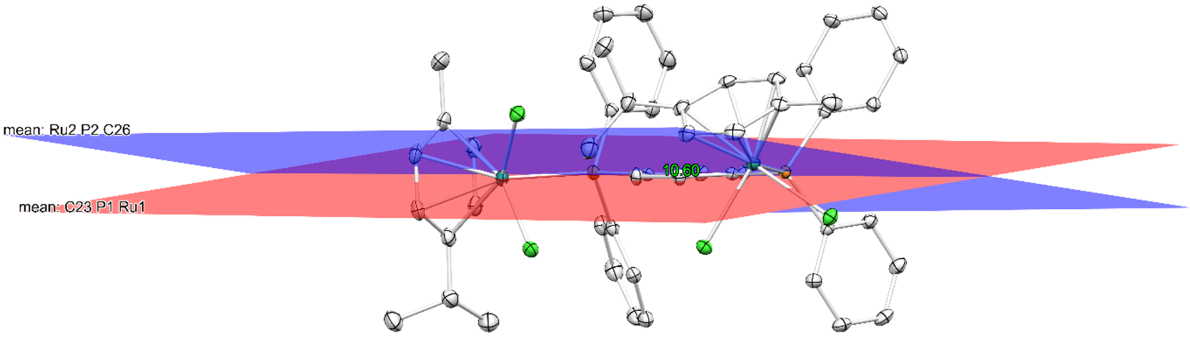


**Figure S71**. ORTEP representation of complex **5**, including representations of the Ru1-P1-C23 and Ru2-P2-C26 planes, and calculated dihedral angle between the two. Thermal ellipsoids represented at the 30% probability level. H atoms omitted for clarity. $CH_2Cl_2$ omitted for clarity.

**Table S15. Crystal data and structure refinement for complex 5.**

| | |
|---|---|
| Empirical formula | $C_{50.5}H_{53}Cl_{5.06}P_2Ru_2$ |
| Formula weight | 1103.38 |
| Temperature/K | 100.00(10) |
| Crystal system | triclinic |

| | |
|---|---|
| Space group | P-1 |
| a/Å | 10.09180(10) |
| b/Å | 14.2482(2) |
| c/Å | 18.8810(3) |
| α/° | 73.7490(10) |
| β/° | 85.0770(10) |
| γ/° | 87.0960(10) |
| Volume/Å$^3$ | 2595.80(6) |
| Z | 2 |
| $\rho_{calc}$g/cm$^3$ | 1.412 |
| μ/mm$^{-1}$ | 7.929 |
| F(000) | 1120.0 |
| Crystal size/mm$^3$ | 0.082 × 0.069 × 0.041 |
| Radiation | Cu Kα (λ = 1.54184) |
| 2Θ range for data collection/° | 6.464 to 160.496 |
| Index ranges | -12 ≤ h ≤ 12, -16 ≤ k ≤ 18, -23 ≤ l ≤ 24 |
| Reflections collected | 67005 |
| Independent reflections | 11124 [$R_{int}$ = 0.0396, $R_{sigma}$ = 0.0283] |
| Data/restraints/parameters | 11124/0/567 |
| Goodness-of-fit on F$^2$ | 1.045 |
| Final R indexes [I>=2σ (I)] | $R_1$ = 0.0360, w$R_2$ = 0.0914 |
| Final R indexes [all data] | $R_1$ = 0.0386, w$R_2$ = 0.0930 |
| Largest diff. peak/hole / e Å$^{-3}$ | 1.95/-1.08 |

**Table S16. Fractional Atomic Coordinates (×10$^4$) and Equivalent Isotropic Displacement Parameters (Å$^2$×10$^3$) for complex 5. $U_{eq}$ is defined as 1/3 of the trace of the orthogonalised $U_{IJ}$ tensor.**

| Atom | *x* | *y* | *z* | U(eq) |
|---|---|---|---|---|
| Ru2 | 3054.5(2) | 1504.4(2) | 9772.1(2) | 19.48(6) |
| Ru1 | 3190.9(2) | 6162.8(2) | 5688.7(2) | 24.32(6) |
| Cl3 | 5153.3(7) | 2255.1(5) | 9272.3(4) | 25.71(14) |
| P2 | 2204.7(6) | 3025.7(5) | 9907.3(4) | 16.86(13) |
| Cl4 | 3840.0(7) | 1240.4(5) | 10985.6(4) | 27.03(14) |
| Cl2 | 2308.5(7) | 4540.1(5) | 5994.3(4) | 30.28(15) |

| | | | | |
|---|---|---|---|---|
| Cl1 | 5035.7(7) | 5505.6(6) | 6418.0(4) | 30.34(15) |
| P1 | 2135.9(7) | 6393.5(5) | 6793.4(4) | 20.95(14) |
| Cl5 | 826(2) | 10227.6(15) | 2811.4(12) | 50.5(4) |
| Cl6 | 3193(3) | 9386(2) | 3583.8(16) | 56.0(11) |
| C26 | 2196(3) | 4008.2(19) | 9040.6(15) | 18.6(5) |
| C25 | 1606(3) | 4928(2) | 9000.0(15) | 20.7(5) |
| C29 | 2910(3) | 3636.3(19) | 10513.9(15) | 20.0(5) |
| C23 | 2204(3) | 5449(2) | 7679.8(15) | 20.3(5) |
| C40 | -576(3) | 3209(2) | 9783.2(17) | 24.5(6) |
| C39 | -1879(3) | 2931(2) | 10028.2(18) | 28.8(6) |
| C24 | 1611(3) | 5640(2) | 8324.3(16) | 22.3(5) |
| C35 | 456(3) | 2873.6(19) | 10245.9(15) | 19.2(5) |
| C22 | 3848(3) | 7299(2) | 7466.2(16) | 26.1(6) |
| C27 | 2816(3) | 3837(2) | 8405.4(16) | 24.2(6) |
| C30 | 2106(3) | 4143(2) | 10928.8(17) | 26.2(6) |
| C11 | 344(3) | 6630(2) | 6778.4(16) | 25.7(6) |
| C42 | 1130(3) | 787(2) | 9933.4(18) | 26.9(6) |
| C31 | 2678(3) | 4683(2) | 11330.3(18) | 31.6(7) |
| C43 | 1323(3) | 1394(2) | 9195.7(18) | 27.1(6) |
| C28 | 2815(3) | 4544(2) | 7724.5(16) | 25.5(6) |
| C34 | 4296(3) | 3678(2) | 10503.5(17) | 25.4(6) |
| C17 | 2797(3) | 7443(2) | 7010.0(16) | 24.4(6) |
| C36 | 157(3) | 2275(2) | 10962.3(16) | 23.8(6) |
| C41 | 2102(3) | 58(2) | 10212.3(18) | 28.7(6) |
| C38 | -2166(3) | 2321(2) | 10729.9(18) | 29.2(6) |
| C5 | 3288(4) | 7737(2) | 5128.5(17) | 32.0(7) |
| C21 | 4420(3) | 8089(3) | 7607.6(19) | 34.3(7) |
| C18 | 2331(3) | 8393(2) | 6686.5(18) | 31.0(6) |
| C12 | -361(3) | 6109(2) | 6415.5(18) | 29.9(6) |
| C46 | 3246(3) | -63(2) | 9739(2) | 31.5(7) |
| C44 | 2422(3) | 1270(2) | 8717.8(18) | 30.3(6) |
| C45 | 3395(3) | 532(2) | 9017.8(19) | 30.4(6) |

| | | | | |
|---|---|---|---|---|
| C33 | 4852(3) | 4229(2) | 10896.7(18) | 30.9(7) |
| C6 | 2070(4) | 7369(3) | 4988.9(17) | 34.5(7) |
| C37 | -1146(3) | 1998(2) | 11201.9(17) | 28.7(6) |
| C48 | 2461(4) | 1847(3) | 7906.2(19) | 37.5(7) |
| C32 | 4043(4) | 4729(2) | 11313.4(19) | 32.5(7) |
| C4 | 4523(4) | 7307(2) | 4973.3(17) | 32.1(7) |
| C47 | 1947(4) | -585(3) | 10991(2) | 38.6(8) |
| C16 | -365(3) | 7219(3) | 7173.1(18) | 32.6(7) |
| C3 | 4534(4) | 6504(3) | 4650.7(17) | 33.4(7) |
| C13 | -1739(3) | 6180(3) | 6445(2) | 36.7(7) |
| C8 | 5833(4) | 7665(3) | 5126(2) | 37.6(7) |
| C2 | 3346(4) | 6157(3) | 4506.3(17) | 36.2(7) |
| C14 | -2425(3) | 6765(3) | 6841(2) | 39.7(8) |
| C50 | 3848(4) | 2000(3) | 7527(2) | 44.9(9) |
| C1 | 2085(4) | 6578(3) | 4678.4(17) | 36.2(7) |
| C20 | 3948(4) | 9025(3) | 7292(2) | 41.2(8) |
| C7 | 831(4) | 6188(4) | 4513(2) | 50.6(10) |
| C19 | 2916(4) | 9184(2) | 6828(2) | 39.6(8) |
| C49 | 1573(5) | 1336(3) | 7516(2) | 48.9(10) |
| C15 | -1734(4) | 7282(3) | 7202(2) | 39.2(8) |
| C10 | 5679(5) | 8438(3) | 5542(2) | 49.1(9) |
| C9 | 6688(5) | 8031(3) | 4403(2) | 51.9(10) |
| C51 | 2529(10) | 10225(8) | 2863(6) | 66(3) |
| Cl6A | 3006(11) | 8953(8) | 3185(7) | 79(6) |

**Table S17. Anisotropic Displacement Parameters (Å²×10³) for complex 5. The Anisotropic displacement factor exponent takes the form: $-2\pi^2[h^2a^{*2}U_{11}+2hka^*b^*U_{12}+\ldots]$.**

| Atom | $U_{11}$ | $U_{22}$ | $U_{33}$ | $U_{23}$ | $U_{13}$ | $U_{12}$ |
|---|---|---|---|---|---|---|
| Ru2 | 19.62(10) | 17.17(10) | 23.21(11) | -8.35(8) | -2.22(8) | 1.57(7) |
| Ru1 | 26.15(12) | 27.80(12) | 19.51(11) | -8.09(8) | 1.08(8) | -1.48(8) |
| Cl3 | 22.3(3) | 25.4(3) | 28.8(3) | -7.3(3) | 0.6(2) | 0.2(2) |
| P2 | 16.6(3) | 17.0(3) | 17.9(3) | -6.5(2) | -1.5(2) | 0.6(2) |
| Cl4 | 33.6(4) | 23.3(3) | 24.5(3) | -6.4(2) | -6.4(3) | 2.3(3) |

| | | | | | | |
|---|---|---|---|---|---|---|
| Cl2 | 31.2(4) | 30.6(3) | 31.7(4) | -13.1(3) | -0.4(3) | -2.8(3) |
| Cl1 | 25.0(3) | 38.5(4) | 27.2(3) | -9.6(3) | 0.6(3) | -0.3(3) |
| P1 | 21.9(3) | 22.0(3) | 18.4(3) | -5.1(2) | -1.3(2) | 1.1(3) |
| Cl5 | 49.5(11) | 46.2(10) | 57.3(11) | -16.3(8) | -7.4(9) | 0.9(8) |
| Cl6 | 58.1(16) | 59.8(17) | 44.0(15) | -2.8(12) | -6.2(10) | -5.4(11) |
| C26 | 15.9(12) | 21.1(12) | 19.2(12) | -6.2(10) | -0.7(9) | -2.2(9) |
| C25 | 21.6(13) | 21.8(13) | 19.4(12) | -7.4(10) | 0.5(10) | -0.5(10) |
| C29 | 23.4(13) | 17.7(12) | 19.1(12) | -5.0(10) | -3.0(10) | -0.8(10) |
| C23 | 17.5(12) | 24.7(13) | 18.1(12) | -4.7(10) | -1.3(10) | -1.7(10) |
| C40 | 21.2(14) | 26.2(14) | 27.0(14) | -8.2(11) | -4.5(11) | 0.7(11) |
| C39 | 18.7(13) | 37.3(16) | 32.5(16) | -13.0(13) | -1.6(11) | 0.1(12) |
| C24 | 26.4(14) | 18.4(12) | 23.0(13) | -7.2(10) | -3.0(11) | 1.4(10) |
| C35 | 18.5(12) | 19.1(12) | 22.2(13) | -9.6(10) | 0.0(10) | -1.3(10) |
| C22 | 24.7(14) | 29.1(15) | 24.6(14) | -8.2(11) | 1.2(11) | -2.1(11) |
| C27 | 26.3(14) | 22.7(13) | 22.9(13) | -6.9(11) | 0.6(11) | 5.9(11) |
| C30 | 26.2(14) | 28.5(14) | 27.2(14) | -12.7(12) | -4.3(11) | 1.6(11) |
| C11 | 24.9(14) | 28.7(14) | 20.4(13) | -2.1(11) | -1.5(11) | 1.7(11) |
| C42 | 23.4(14) | 23.9(14) | 38.6(16) | -16.3(12) | -1.7(12) | -6.2(11) |
| C31 | 36.8(17) | 31.3(16) | 32.2(16) | -16.9(13) | -7.3(13) | 2.4(13) |
| C43 | 24.4(14) | 29.1(15) | 34.7(16) | -17.7(12) | -10.6(12) | 0.2(11) |
| C28 | 27.4(14) | 27.8(14) | 21.6(13) | -9.4(11) | 2.2(11) | 4.1(11) |
| C34 | 23.4(14) | 24.8(14) | 28.8(14) | -8.2(11) | -3.0(11) | -2.0(11) |
| C17 | 27.3(14) | 23.1(13) | 22.9(13) | -7.8(11) | 2.3(11) | -0.2(11) |
| C36 | 24.5(14) | 25.5(14) | 22.7(13) | -9.3(11) | 1.1(11) | -2.7(11) |
| C41 | 29.9(15) | 18.8(13) | 38.3(17) | -8.2(12) | -2.2(13) | -7.0(11) |
| C38 | 22.0(14) | 32.3(15) | 36.5(16) | -16.4(13) | 6.7(12) | -5.6(12) |
| C5 | 40.0(18) | 28.3(15) | 24.4(15) | -3.6(12) | -0.4(13) | 3.3(13) |
| C21 | 32.0(17) | 41.9(18) | 33.8(17) | -19.0(14) | 5.9(13) | -10.1(14) |
| C18 | 36.7(17) | 26.2(15) | 27.9(15) | -5.8(12) | 1.6(13) | 2.1(13) |
| C12 | 25.0(15) | 32.0(16) | 32.2(16) | -8.5(13) | -1.3(12) | 0.5(12) |
| C46 | 33.0(16) | 19.6(14) | 47.3(19) | -17.6(13) | -5.2(14) | 1.8(12) |
| C44 | 35.8(17) | 33.1(16) | 29.2(15) | -18.9(13) | -6.3(13) | -1.8(13) |

| | | | | | | |
|---|---|---|---|---|---|---|
| C45 | 30.1(16) | 31.0(15) | 38.0(17) | -23.8(13) | 0.1(13) | 1.1(12) |
| C33 | 29.6(16) | 29.0(15) | 35.5(16) | -8.1(13) | -10.7(13) | -6.3(12) |
| C6 | 37.5(18) | 37.5(17) | 23.1(15) | -1.0(13) | -1.9(13) | 7.1(14) |
| C37 | 31.5(16) | 30.0(15) | 25.6(14) | -11.4(12) | 7.2(12) | -6.4(12) |
| C48 | 46(2) | 43.3(19) | 28.1(16) | -17.4(14) | -3.9(14) | -1.8(15) |
| C32 | 42.0(18) | 27.0(15) | 33.7(16) | -12.7(13) | -15.7(14) | -2.7(13) |
| C4 | 37.9(18) | 32.6(16) | 22.4(14) | -2.7(12) | 3.5(12) | -7.0(13) |
| C47 | 38.3(19) | 30.7(16) | 43.3(19) | -3.3(14) | -2.7(15) | -7.6(14) |
| C16 | 33.1(17) | 40.8(18) | 25.0(15) | -11.7(13) | -3.5(12) | 4.6(14) |
| C3 | 36.4(17) | 37.6(17) | 23.3(14) | -6.3(12) | 8.2(13) | -1.9(14) |
| C13 | 26.7(16) | 44.8(19) | 39.4(18) | -11.7(15) | -7.2(13) | -0.9(14) |
| C8 | 35.0(18) | 40.7(18) | 34.0(17) | -5.0(14) | -0.2(14) | -6.9(14) |
| C2 | 51(2) | 41.7(18) | 17.2(14) | -11.1(13) | -0.3(13) | -3.0(15) |
| C14 | 23.9(16) | 56(2) | 37.2(18) | -11.1(16) | -1.2(13) | 4.4(15) |
| C50 | 54(2) | 48(2) | 36.6(19) | -18.2(16) | -0.6(17) | -2.0(18) |
| C1 | 40.2(19) | 47.4(19) | 18.8(14) | -4.3(13) | -4.8(13) | -4.8(15) |
| C20 | 48(2) | 35.6(18) | 45(2) | -20.7(15) | 10.0(16) | -16.5(16) |
| C7 | 47(2) | 75(3) | 32.0(18) | -14.6(19) | -8.5(16) | -14(2) |
| C19 | 52(2) | 24.4(15) | 40.6(19) | -10.3(14) | 10.2(16) | -2.3(14) |
| C49 | 56(2) | 61(2) | 38(2) | -25.5(18) | -7.2(18) | -6(2) |
| C15 | 31.0(17) | 54(2) | 33.3(17) | -14.4(15) | -2.4(14) | 11.2(15) |
| C10 | 52(2) | 53(2) | 46(2) | -17.4(18) | 0.2(18) | -15.6(19) |
| C9 | 53(2) | 58(2) | 44(2) | -14.8(19) | 14.3(18) | -20(2) |
| C51 | 59(6) | 69(6) | 51(5) | 17(5) | -9(4) | -1(5) |
| Cl6A | 71(7) | 58(7) | 77(9) | 23(6) | 0(5) | 21(5) |

**Table S18. Bond Lengths for complex 5.**

| Atom | Atom | Length/Å | Atom | Atom | Length/Å |
|---|---|---|---|---|---|
| Ru2 | Cl3 | 2.4171(7) | C22 | C21 | 1.391(4) |
| Ru2 | P2 | 2.3627(7) | C27 | C28 | 1.394(4) |
| Ru2 | Cl4 | 2.4122(7) | C30 | C31 | 1.395(4) |
| Ru2 | C42 | 2.199(3) | C11 | C12 | 1.395(4) |

| | | | | | |
|---|---|---|---|---|---|
| Ru2 | C43 | 2.171(3) | C11 | C16 | 1.403(4) |
| Ru2 | C41 | 2.228(3) | C42 | C43 | 1.422(5) |
| Ru2 | C46 | 2.251(3) | C42 | C41 | 1.414(4) |
| Ru2 | C44 | 2.257(3) | C31 | C32 | 1.379(5) |
| Ru2 | C45 | 2.245(3) | C43 | C44 | 1.405(5) |
| Ru1 | Cl2 | 2.4177(8) | C34 | C33 | 1.387(4) |
| Ru1 | Cl1 | 2.4043(8) | C17 | C18 | 1.396(4) |
| Ru1 | P1 | 2.3578(7) | C36 | C37 | 1.392(4) |
| Ru1 | C5 | 2.199(3) | C41 | C46 | 1.432(5) |
| Ru1 | C6 | 2.184(3) | C41 | C47 | 1.499(5) |
| Ru1 | C4 | 2.229(3) | C38 | C37 | 1.395(5) |
| Ru1 | C3 | 2.234(3) | C5 | C6 | 1.433(5) |
| Ru1 | C2 | 2.227(3) | C5 | C4 | 1.405(5) |
| Ru1 | C1 | 2.214(3) | C21 | C20 | 1.378(5) |
| P2 | C26 | 1.832(3) | C18 | C19 | 1.398(5) |
| P2 | C29 | 1.827(3) | C12 | C13 | 1.386(5) |
| P2 | C35 | 1.826(3) | C46 | C45 | 1.388(5) |
| P1 | C23 | 1.835(3) | C44 | C45 | 1.432(5) |
| P1 | C11 | 1.824(3) | C44 | C48 | 1.521(5) |
| P1 | C17 | 1.827(3) | C33 | C32 | 1.392(5) |
| Cl5 | C51 | 1.729(10) | C6 | C1 | 1.408(5) |
| Cl6 | C51 | 1.702(9) | C48 | C50 | 1.513(6) |
| Cl6 | Cl6A | 1.131(16) | C48 | C49 | 1.536(5) |
| C26 | C25 | 1.397(4) | C4 | C3 | 1.438(5) |
| C26 | C27 | 1.382(4) | C4 | C8 | 1.518(5) |
| C25 | C24 | 1.389(4) | C16 | C15 | 1.378(5) |
| C29 | C30 | 1.393(4) | C3 | C2 | 1.395(5) |
| C29 | C34 | 1.401(4) | C13 | C14 | 1.392(5) |
| C23 | C24 | 1.399(4) | C8 | C10 | 1.519(6) |
| C23 | C28 | 1.385(4) | C8 | C9 | 1.525(5) |
| C40 | C39 | 1.395(4) | C2 | C1 | 1.430(5) |
| C40 | C35 | 1.396(4) | C14 | C15 | 1.381(5) |

| | | | | | |
|---|---|---|---|---|---|
| C39 | C38 | 1.381(5) | C1 | C7 | 1.498(5) |
| C35 | C36 | 1.400(4) | C20 | C19 | 1.386(6) |
| C22 | C17 | 1.395(4) | C51 | Cl6A | 1.797(15) |

**Table S19. Bond Angles for complex 5.**

| Atom | Atom | Atom | Angle/° | Atom | Atom | Atom | Angle/° |
|---|---|---|---|---|---|---|---|
| P2 | Ru2 | Cl3 | 89.33(2) | C28 | C23 | P1 | 121.5(2) |
| P2 | Ru2 | Cl4 | 85.93(2) | C28 | C23 | C24 | 119.1(3) |
| Cl4 | Ru2 | Cl3 | 87.50(3) | C39 | C40 | C35 | 120.3(3) |
| C42 | Ru2 | Cl3 | 165.08(9) | C38 | C39 | C40 | 120.6(3) |
| C42 | Ru2 | P2 | 96.55(8) | C25 | C24 | C23 | 120.8(3) |
| C42 | Ru2 | Cl4 | 106.54(9) | C40 | C35 | P2 | 122.2(2) |
| C42 | Ru2 | C41 | 37.25(12) | C40 | C35 | C36 | 118.9(3) |
| C42 | Ru2 | C46 | 66.92(12) | C36 | C35 | P2 | 118.2(2) |
| C42 | Ru2 | C44 | 67.53(12) | C21 | C22 | C17 | 120.7(3) |
| C42 | Ru2 | C45 | 78.69(12) | C26 | C27 | C28 | 121.6(3) |
| C43 | Ru2 | Cl3 | 128.89(9) | C29 | C30 | C31 | 120.2(3) |
| C43 | Ru2 | P2 | 89.26(8) | C12 | C11 | P1 | 117.1(2) |
| C43 | Ru2 | Cl4 | 143.26(9) | C12 | C11 | C16 | 118.9(3) |
| C43 | Ru2 | C42 | 37.97(12) | C16 | C11 | P1 | 123.6(2) |
| C43 | Ru2 | C41 | 67.59(12) | C43 | C42 | Ru2 | 69.95(16) |
| C43 | Ru2 | C46 | 78.77(12) | C41 | C42 | Ru2 | 72.50(17) |
| C43 | Ru2 | C44 | 36.92(12) | C41 | C42 | C43 | 119.3(3) |
| C43 | Ru2 | C45 | 66.37(12) | C32 | C31 | C30 | 120.3(3) |
| C41 | Ru2 | Cl3 | 142.53(9) | C42 | C43 | Ru2 | 72.07(16) |
| C41 | Ru2 | P2 | 127.04(9) | C44 | C43 | Ru2 | 74.86(18) |
| C41 | Ru2 | Cl4 | 86.67(9) | C44 | C43 | C42 | 122.4(3) |
| C41 | Ru2 | C46 | 37.30(12) | C23 | C28 | C27 | 119.7(3) |
| C41 | Ru2 | C44 | 79.47(12) | C33 | C34 | C29 | 120.0(3) |
| C41 | Ru2 | C45 | 66.25(12) | C22 | C17 | P1 | 119.4(2) |
| C46 | Ru2 | Cl3 | 107.17(9) | C22 | C17 | C18 | 119.3(3) |
| C46 | Ru2 | P2 | 163.36(9) | C18 | C17 | P1 | 121.1(2) |

| | | | | | | | |
|---|---|---|---|---|---|---|---|
| C46 | Ru2 | Cl4 | 96.73(9) | C37 | C36 | C35 | 120.4(3) |
| C46 | Ru2 | C44 | 66.35(12) | C42 | C41 | Ru2 | 70.25(16) |
| C44 | Ru2 | Cl3 | 97.58(9) | C42 | C41 | C46 | 119.1(3) |
| C44 | Ru2 | P2 | 110.17(9) | C42 | C41 | C47 | 120.9(3) |
| C44 | Ru2 | Cl4 | 163.08(9) | C46 | C41 | Ru2 | 72.20(17) |
| C45 | Ru2 | Cl3 | 88.81(9) | C46 | C41 | C47 | 120.0(3) |
| C45 | Ru2 | P2 | 146.34(9) | C47 | C41 | Ru2 | 129.6(2) |
| C45 | Ru2 | Cl4 | 127.54(9) | C39 | C38 | C37 | 119.6(3) |
| C45 | Ru2 | C46 | 35.97(13) | C6 | C5 | Ru1 | 70.35(18) |
| C45 | Ru2 | C44 | 37.09(12) | C4 | C5 | Ru1 | 72.67(18) |
| Cl1 | Ru1 | Cl2 | 89.30(3) | C4 | C5 | C6 | 121.0(3) |
| P1 | Ru1 | Cl2 | 90.07(3) | C20 | C21 | C22 | 119.7(3) |
| P1 | Ru1 | Cl1 | 85.44(3) | C17 | C18 | C19 | 119.7(3) |
| C5 | Ru1 | Cl2 | 156.17(9) | C13 | C12 | C11 | 120.2(3) |
| C5 | Ru1 | Cl1 | 114.40(9) | C41 | C46 | Ru2 | 70.50(16) |
| C5 | Ru1 | P1 | 94.06(9) | C45 | C46 | Ru2 | 71.80(17) |
| C5 | Ru1 | C4 | 37.01(13) | C45 | C46 | C41 | 120.2(3) |
| C5 | Ru1 | C3 | 66.75(12) | C43 | C44 | Ru2 | 68.21(16) |
| C5 | Ru1 | C2 | 78.87(13) | C43 | C44 | C45 | 117.0(3) |
| C5 | Ru1 | C1 | 68.00(13) | C43 | C44 | C48 | 119.3(3) |
| C6 | Ru1 | Cl2 | 118.21(10) | C45 | C44 | Ru2 | 71.00(17) |
| C6 | Ru1 | Cl1 | 152.48(10) | C45 | C44 | C48 | 123.4(3) |
| C6 | Ru1 | P1 | 93.32(9) | C48 | C44 | Ru2 | 136.4(2) |
| C6 | Ru1 | C5 | 38.17(13) | C46 | C45 | Ru2 | 72.23(17) |
| C6 | Ru1 | C4 | 68.10(13) | C46 | C45 | C44 | 122.0(3) |
| C6 | Ru1 | C3 | 79.41(13) | C44 | C45 | Ru2 | 71.91(16) |
| C6 | Ru1 | C2 | 66.98(13) | C34 | C33 | C32 | 120.4(3) |
| C6 | Ru1 | C1 | 37.33(14) | C5 | C6 | Ru1 | 71.48(18) |
| C4 | Ru1 | Cl2 | 150.07(9) | C1 | C6 | Ru1 | 72.50(19) |
| C4 | Ru1 | Cl1 | 88.60(9) | C1 | C6 | C5 | 120.6(3) |
| C4 | Ru1 | P1 | 119.49(9) | C36 | C37 | C38 | 120.1(3) |
| C4 | Ru1 | C3 | 37.59(12) | C44 | C48 | C49 | 107.8(3) |

| | | | | | | | |
|---|---|---|---|---|---|---|---|
| C3 | Ru1 | Cl2 | 112.64(9) | C50 | C48 | C44 | 114.3(3) |
| C3 | Ru1 | Cl1 | 91.08(9) | C50 | C48 | C49 | 112.2(3) |
| C3 | Ru1 | P1 | 157.00(9) | C31 | C32 | C33 | 119.8(3) |
| C2 | Ru1 | Cl2 | 87.93(10) | C5 | C4 | Ru1 | 70.33(19) |
| C2 | Ru1 | Cl1 | 118.31(10) | C5 | C4 | C3 | 118.1(3) |
| C2 | Ru1 | P1 | 156.12(10) | C5 | C4 | C8 | 122.6(3) |
| C2 | Ru1 | C4 | 67.06(13) | C3 | C4 | Ru1 | 71.42(18) |
| C2 | Ru1 | C3 | 36.45(13) | C3 | C4 | C8 | 119.3(3) |
| C1 | Ru1 | Cl2 | 89.45(10) | C8 | C4 | Ru1 | 130.1(2) |
| C1 | Ru1 | Cl1 | 155.88(10) | C15 | C16 | C11 | 120.5(3) |
| C1 | Ru1 | P1 | 118.65(10) | C4 | C3 | Ru1 | 70.99(18) |
| C1 | Ru1 | C4 | 80.63(13) | C2 | C3 | Ru1 | 71.48(18) |
| C1 | Ru1 | C3 | 67.30(13) | C2 | C3 | C4 | 120.6(3) |
| C1 | Ru1 | C2 | 37.57(14) | C12 | C13 | C14 | 120.1(3) |
| C26 | P2 | Ru2 | 114.43(9) | C4 | C8 | C10 | 114.0(3) |
| C29 | P2 | Ru2 | 122.14(9) | C4 | C8 | C9 | 109.8(3) |
| C29 | P2 | C26 | 101.43(12) | C10 | C8 | C9 | 110.7(3) |
| C35 | P2 | Ru2 | 107.66(9) | C3 | C2 | Ru1 | 72.06(18) |
| C35 | P2 | C26 | 104.93(12) | C3 | C2 | C1 | 121.5(3) |
| C35 | P2 | C29 | 104.70(12) | C1 | C2 | Ru1 | 70.72(18) |
| C23 | P1 | Ru1 | 121.75(9) | C15 | C14 | C13 | 120.0(3) |
| C11 | P1 | Ru1 | 114.77(10) | C6 | C1 | Ru1 | 70.16(18) |
| C11 | P1 | C23 | 100.19(13) | C6 | C1 | C2 | 118.1(3) |
| C11 | P1 | C17 | 105.29(14) | C6 | C1 | C7 | 121.9(4) |
| C17 | P1 | Ru1 | 111.19(10) | C2 | C1 | Ru1 | 71.71(19) |
| C17 | P1 | C23 | 101.67(13) | C2 | C1 | C7 | 120.0(4) |
| Cl6A | Cl6 | C51 | 75.8(6) | C7 | C1 | Ru1 | 130.5(3) |
| C25 | C26 | P2 | 122.5(2) | C21 | C20 | C19 | 120.5(3) |
| C27 | C26 | P2 | 118.7(2) | C20 | C19 | C18 | 120.1(3) |
| C27 | C26 | C25 | 118.8(2) | C16 | C15 | C14 | 120.3(3) |
| C24 | C25 | C26 | 120.0(3) | Cl5 | C51 | Cl6A | 104.5(7) |
| C30 | C29 | P2 | 121.4(2) | Cl6 | C51 | Cl5 | 117.5(6) |

| | | | | | | | |
|---|---|---|---|---|---|---|---|
| C30 | C29 | C34 | 119.2(3) | Cl6 | C51 | Cl6A | 37.6(5) |
| C34 | C29 | P2 | 119.0(2) | Cl6 | Cl6A | C51 | 66.6(7) |
| C24 | C23 | P1 | 119.4(2) | | | | |

**Table S20. Torsion Angles for complex 5.**

| A | B | C | D | Angle/° | A | B | C | D | Angle/° |
|---|---|---|---|---|---|---|---|---|---|
| Ru2 | P2 | C26 | C25 | 173.26(19) | C35 | C36 | C37 | C38 | -0.4(4) |
| Ru2 | P2 | C26 | C27 | -7.8(3) | C22 | C17 | C18 | C19 | -0.5(5) |
| Ru2 | P2 | C29 | C30 | -142.2(2) | C22 | C21 | C20 | C19 | -0.7(5) |
| Ru2 | P2 | C29 | C34 | 44.4(3) | C27 | C26 | C25 | C24 | 2.2(4) |
| Ru2 | P2 | C35 | C40 | -102.7(2) | C30 | C29 | C34 | C33 | -0.7(4) |
| Ru2 | P2 | C35 | C36 | 67.6(2) | C30 | C31 | C32 | C33 | -0.3(5) |
| Ru2 | C42 | C43 | C44 | 58.0(3) | C11 | P1 | C23 | C24 | -55.1(3) |
| Ru2 | C42 | C41 | C46 | -55.2(2) | C11 | P1 | C23 | C28 | 124.0(2) |
| Ru2 | C42 | C41 | C47 | 125.1(3) | C11 | P1 | C17 | C22 | 142.6(2) |
| Ru2 | C43 | C44 | C45 | 53.4(2) | C11 | P1 | C17 | C18 | -41.9(3) |
| Ru2 | C43 | C44 | C48 | -132.1(3) | C11 | C12 | C13 | C14 | -0.5(5) |
| Ru2 | C41 | C46 | C45 | -53.9(3) | C11 | C16 | C15 | C14 | 0.0(5) |
| Ru2 | C46 | C45 | C44 | -54.2(3) | C42 | C43 | C44 | Ru2 | -56.7(2) |
| Ru2 | C44 | C45 | C46 | 54.3(3) | C42 | C43 | C44 | C45 | -3.3(4) |
| Ru2 | C44 | C48 | C50 | 66.9(4) | C42 | C43 | C44 | C48 | 171.3(3) |
| Ru2 | C44 | C48 | C49 | -167.6(3) | C42 | C41 | C46 | Ru2 | 54.2(2) |
| Ru1 | P1 | C23 | C24 | 177.19(18) | C42 | C41 | C46 | C45 | 0.3(4) |
| Ru1 | P1 | C23 | C28 | -3.7(3) | C43 | C42 | C41 | Ru2 | 53.9(2) |
| Ru1 | P1 | C11 | C12 | 38.6(3) | C43 | C42 | C41 | C46 | -1.2(4) |
| Ru1 | P1 | C11 | C16 | -148.3(2) | C43 | C42 | C41 | C47 | 179.0(3) |
| Ru1 | P1 | C17 | C22 | -92.5(2) | C43 | C44 | C45 | Ru2 | -52.0(2) |
| Ru1 | P1 | C17 | C18 | 83.0(3) | C43 | C44 | C45 | C46 | 2.3(4) |
| Ru1 | C5 | C6 | C1 | -55.5(3) | C43 | C44 | C48 | C50 | 155.3(3) |
| Ru1 | C5 | C4 | C3 | 54.9(3) | C43 | C44 | C48 | C49 | -79.1(4) |
| Ru1 | C5 | C4 | C8 | -125.7(3) | C28 | C23 | C24 | C25 | -1.3(4) |
| Ru1 | C6 | C1 | C2 | -55.2(3) | C34 | C29 | C30 | C31 | -0.2(4) |

| | | | | |
|---|---|---|---|---|
| Ru1 | C6 | C1 | C7 | 126.1(3) |
| Ru1 | C4 | C3 | C2 | 53.6(3) |
| Ru1 | C4 | C8 | C10 | -83.9(4) |
| Ru1 | C4 | C8 | C9 | 151.1(3) |
| Ru1 | C3 | C2 | C1 | 52.8(3) |
| Ru1 | C2 | C1 | C6 | 54.4(3) |
| Ru1 | C2 | C1 | C7 | -126.8(3) |
| P2 | C26 | C25 | C24 | -178.9(2) |
| P2 | C26 | C27 | C28 | 178.3(2) |
| P2 | C29 | C30 | C31 | -173.6(2) |
| P2 | C29 | C34 | C33 | 172.9(2) |
| P2 | C35 | C36 | C37 | -168.9(2) |
| P1 | C23 | C24 | C25 | 177.8(2) |
| P1 | C23 | C28 | C27 | -178.3(2) |
| P1 | C11 | C12 | C13 | 173.6(3) |
| P1 | C11 | C16 | C15 | -172.9(3) |
| P1 | C17 | C18 | C19 | -176.0(3) |
| Cl5 | C51 | Cl6A | Cl6 | -116.4(7) |
| C26 | P2 | C29 | C30 | 89.1(2) |
| C26 | P2 | C29 | C34 | -84.3(2) |
| C26 | P2 | C35 | C40 | 19.6(3) |
| C26 | P2 | C35 | C36 | -170.2(2) |
| C26 | C25 | C24 | C23 | -0.2(4) |
| C26 | C27 | C28 | C23 | 1.3(5) |
| C25 | C26 | C27 | C28 | -2.8(4) |
| C29 | P2 | C26 | C25 | -53.3(2) |
| C29 | P2 | C26 | C27 | 125.6(2) |
| C29 | P2 | C35 | C40 | 126.0(2) |
| C29 | P2 | C35 | C36 | -63.8(2) |
| C29 | C30 | C31 | C32 | 0.7(5) |
| C29 | C34 | C33 | C32 | 1.1(5) |
| C23 | P1 | C11 | C12 | -93.6(2) |

| | | | | |
|---|---|---|---|---|
| C34 | C33 | C32 | C31 | -0.6(5) |
| C17 | P1 | C23 | C24 | 53.0(2) |
| C17 | P1 | C23 | C28 | -127.9(2) |
| C17 | P1 | C11 | C12 | 161.2(2) |
| C17 | P1 | C11 | C16 | -25.7(3) |
| C17 | C22 | C21 | C20 | -0.3(5) |
| C17 | C18 | C19 | C20 | -0.4(5) |
| C41 | C42 | C43 | Ru2 | -55.1(2) |
| C41 | C42 | C43 | C44 | 2.8(4) |
| C41 | C46 | C45 | Ru2 | 53.3(3) |
| C41 | C46 | C45 | C44 | -0.9(5) |
| C5 | C6 | C1 | Ru1 | 55.0(3) |
| C5 | C6 | C1 | C2 | -0.2(5) |
| C5 | C6 | C1 | C7 | -178.9(3) |
| C5 | C4 | C3 | Ru1 | -54.4(3) |
| C5 | C4 | C3 | C2 | -0.8(5) |
| C5 | C4 | C8 | C10 | 7.0(5) |
| C5 | C4 | C8 | C9 | -117.9(4) |
| C21 | C22 | C17 | P1 | 176.4(2) |
| C21 | C22 | C17 | C18 | 0.8(5) |
| C21 | C20 | C19 | C18 | 1.0(5) |
| C12 | C11 | C16 | C15 | 0.0(5) |
| C12 | C13 | C14 | C15 | 0.5(6) |
| C45 | C44 | C48 | C50 | -30.5(4) |
| C45 | C44 | C48 | C49 | 95.0(4) |
| C6 | C5 | C4 | Ru1 | -53.2(3) |
| C6 | C5 | C4 | C3 | 1.7(5) |
| C6 | C5 | C4 | C8 | -178.9(3) |
| C48 | C44 | C45 | Ru2 | 133.7(3) |
| C48 | C44 | C45 | C46 | -172.0(3) |
| C4 | C5 | C6 | Ru1 | 54.3(3) |
| C4 | C5 | C6 | C1 | -1.2(5) |

| | | | | | | | | | |
|---|---|---|---|---|---|---|---|---|---|
| C23 | P1 | C11 | C16 | 79.5(3) | C4 | C3 | C2 | Ru1 | -53.3(3) |
| C23 | P1 | C17 | C22 | 38.5(3) | C4 | C3 | C2 | C1 | -0.6(5) |
| C23 | P1 | C17 | C18 | -146.0(3) | C47 | C41 | C46 | Ru2 | -126.0(3) |
| C40 | C39 | C38 | C37 | 1.3(5) | C47 | C41 | C46 | C45 | -179.9(3) |
| C40 | C35 | C36 | C37 | 1.7(4) | C16 | C11 | C12 | C13 | 0.2(5) |
| C39 | C40 | C35 | P2 | 168.7(2) | C3 | C4 | C8 | C10 | -173.6(3) |
| C39 | C40 | C35 | C36 | -1.5(4) | C3 | C4 | C8 | C9 | 61.4(4) |
| C39 | C38 | C37 | C36 | -1.1(5) | C3 | C2 | C1 | Ru1 | -53.4(3) |
| C24 | C23 | C28 | C27 | 0.8(4) | C3 | C2 | C1 | C6 | 1.1(5) |
| C35 | P2 | C26 | C25 | 55.5(2) | C3 | C2 | C1 | C7 | 179.8(3) |
| C35 | P2 | C26 | C27 | -125.6(2) | C13 | C14 | C15 | C16 | -0.3(6) |
| C35 | P2 | C29 | C30 | -19.8(3) | C8 | C4 | C3 | Ru1 | 126.2(3) |
| C35 | P2 | C29 | C34 | 166.7(2) | C8 | C4 | C3 | C2 | 179.8(3) |
| C35 | C40 | C39 | C38 | 0.0(5) | Cl6A | Cl6 | C51 | Cl5 | 77.7(9) |

**Table S21. Hydrogen Atom Coordinates (Å×10$^4$) and Isotropic Displacement Parameters (Å$^2$×10$^3$) for complex 5.**

| Atom | *x* | *y* | *z* | U(eq) |
|---|---|---|---|---|
| H25 | 1203.01 | 5067.88 | 9434.06 | 25 |
| H40 | -390.28 | 3628.26 | 9299.63 | 29 |
| H39 | -2574.92 | 3162.08 | 9709.58 | 35 |
| H24 | 1205.51 | 6263.62 | 8299.74 | 27 |
| H22 | 4176.63 | 6654.26 | 7682.49 | 31 |
| H27 | 3252.57 | 3222.64 | 8433.32 | 29 |
| H30 | 1165.36 | 4120.56 | 10938.27 | 31 |
| H42 | 355.5 | 871.1 | 10235.73 | 32 |
| H31 | 2124.64 | 5021.28 | 11616.53 | 38 |
| H43 | 685.3 | 1901.89 | 9019.64 | 33 |
| H28 | 3231.83 | 4405.55 | 7292.97 | 31 |
| H34 | 4854.3 | 3330.12 | 10227.1 | 30 |
| H36 | 846.72 | 2055.78 | 11287.18 | 29 |
| H38 | -3052.38 | 2123.99 | 10889.95 | 35 |
| H5 | 3259.58 | 8279.39 | 5329.21 | 38 |

| | | | | |
|---|---|---|---|---|
| H21 | 5133.28 | 7984.93 | 7920.61 | 41 |
| H18 | 1620.61 | 8501.37 | 6371.48 | 37 |
| H12 | 104 | 5705.5 | 6147.47 | 36 |
| H46 | 3904.76 | -552.92 | 9918.62 | 38 |
| H45 | 4166.34 | 446.49 | 8713.98 | 36 |
| H33 | 5792.19 | 4264.73 | 10881.69 | 37 |
| H6 | 1244.35 | 7661.71 | 5106.93 | 41 |
| H37 | -1340.01 | 1588.13 | 11687.99 | 34 |
| H48 | 2049.11 | 2507.61 | 7880.81 | 45 |
| H32 | 4428.83 | 5099.81 | 11585.26 | 39 |
| H47A | 2801.91 | -657.83 | 11213.09 | 58 |
| H47B | 1651.68 | -1228.98 | 10989.13 | 58 |
| H47C | 1284.23 | -288.41 | 11279.81 | 58 |
| H16 | 103.8 | 7576.72 | 7422.65 | 39 |
| H3 | 5357.49 | 6207.27 | 4534.78 | 40 |
| H13 | -2215.52 | 5828.58 | 6194.18 | 44 |
| H8 | 6315.49 | 7088.54 | 5440.15 | 45 |
| H2 | 3376.14 | 5629.81 | 4288.71 | 43 |
| H14 | -3369.88 | 6808.85 | 6864.17 | 48 |
| H50A | 3801.37 | 2449.4 | 7030.47 | 67 |
| H50B | 4241.31 | 1370.85 | 7489.36 | 67 |
| H50C | 4399.69 | 2277.95 | 7816.2 | 67 |
| H20 | 4331.57 | 9564.93 | 7392.45 | 49 |
| H7A | 868.6 | 5471.43 | 4679.94 | 76 |
| H7B | 736.85 | 6403.16 | 3978.86 | 76 |
| H7C | 66.42 | 6433.46 | 4771.91 | 76 |
| H19 | 2606.6 | 9832.26 | 6606.7 | 48 |
| H49A | 1939.56 | 678.23 | 7540.48 | 73 |
| H49B | 1539.94 | 1715.02 | 6996.61 | 73 |
| H49C | 672.56 | 1288.42 | 7760.19 | 73 |
| H15 | -2204.62 | 7683.29 | 7470.38 | 47 |
| H10A | 5266.42 | 9032.95 | 5233.65 | 74 |

| | | | | |
|---|---|---|---|---|
| H10B | 6556.25 | 8587.73 | 5662.11 | 74 |
| H10C | 5115.07 | 8193.91 | 6000.6 | 74 |
| H9A | 6218.84 | 8573.44 | 4068.92 | 78 |
| H9B | 6859.24 | 7497.57 | 4169.86 | 78 |
| H9C | 7534.09 | 8255.93 | 4504.5 | 78 |
| H51A | 2963.41 | 10123.63 | 2398.59 | 79 |
| H51B | 2767.07 | 10883.05 | 2881.37 | 79 |

**Table S22. Atomic Occupancy for complex 5.**

| **Atom** | ***Occupancy*** | **Atom** | ***Occupancy*** | **Atom** | ***Occupancy*** |
|---|---|---|---|---|---|
| Cl5 | 0.5 | Cl6 | 0.431(6) | C51 | 0.5 |
| H51A | 0.5 | H51B | 0.5 | Cl6A | 0.129(7) |

**Table S23. Solvent masks information for complex 5.**

| **Number** | **X** | **Y** | **Z** | **Volume** | **Electron count** | **Content** |
|---|---|---|---|---|---|---|
| 1 | 0.000 | 0.000 | 0.500 | 331.9 | 102.4 | 2 CH2Cl2 |

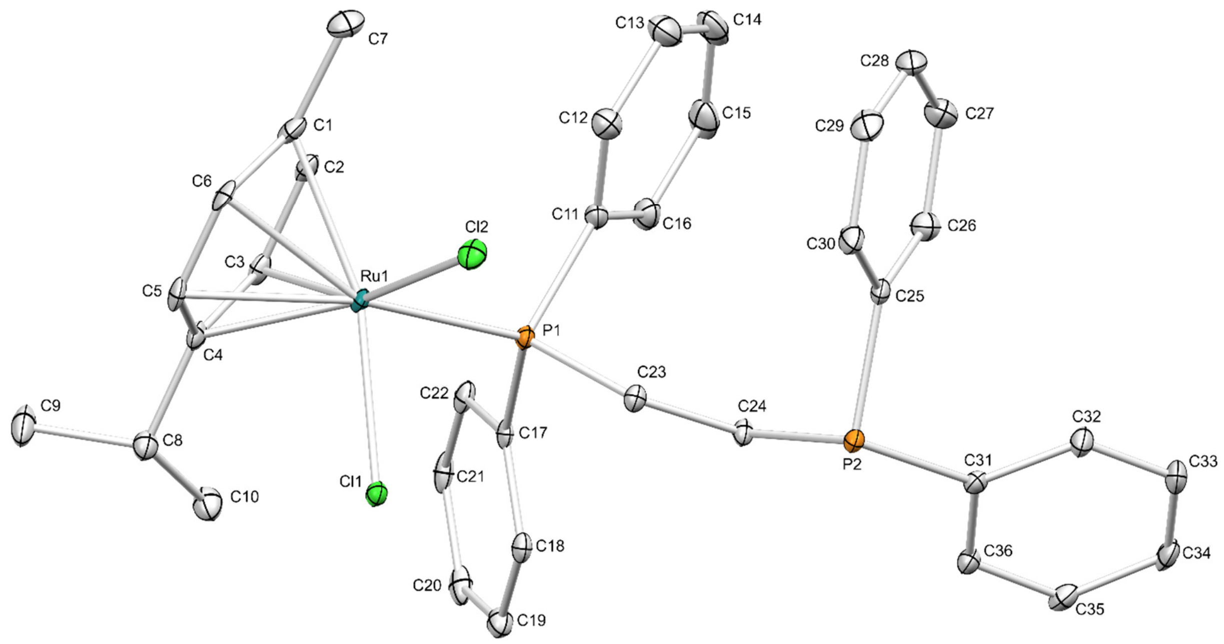


**Figure S72**. ORTEP representation of the two entities of **6**, with complete atom labellings, in the solid state determined by single crystal X-ray diffraction analysis. Thermal ellipsoids represented at the 30% probability level. H atoms omitted for clarity.

**Table S24. Crystal data and structure refinement for complex 6.**

| | |
|---|---|
| Empirical formula | $C_{72}H_{76}Cl_4P_4Ru_2$ |
| Formula weight | 1409.14 |
| Temperature/K | 100.00 |
| Crystal system | monoclinic |
| Space group | $P2_1/n$ |
| a/Å | 12.59299(10) |
| b/Å | 17.95904(15) |
| c/Å | 15.11860(15) |
| α/° | 90 |
| β/° | 106.4350(9) |
| γ/° | 90 |
| Volume/Å$^3$ | 3279.49(5) |
| Z | 2 |
| $\rho_{calc}$g/cm$^3$ | 1.427 |

| | |
|---|---|
| μ/mm$^{-1}$ | 6.465 |
| F(000) | 1448.0 |
| Crystal size/mm$^3$ | 0.39 × 0.26 × 0.17 |
| Radiation | Cu K$\alpha$ ($\lambda$ = 1.54184) |
| 2Θ range for data collection/° | 7.836 to 159.388 |
| Index ranges | -11 ≤ h ≤ 15, -22 ≤ k ≤ 22, -19 ≤ l ≤ 18 |
| Reflections collected | 70878 |
| Independent reflections | 7093 [$R_{int}$ = 0.0577, $R_{sigma}$ = 0.0250] |
| Data/restraints/parameters | 7093/0/374 |
| Goodness-of-fit on F$^2$ | 1.125 |
| Final R indexes [I>=2σ (I)] | $R_1$ = 0.0363, w$R_2$ = 0.0916 |
| Final R indexes [all data] | $R_1$ = 0.0367, w$R_2$ = 0.0919 |
| Largest diff. peak/hole / e Å$^{-3}$ | 1.30/-1.42 |

**Table S25. Fractional Atomic Coordinates (×10$^4$) and Equivalent Isotropic Displacement Parameters (Å$^2$×10$^3$) for complex 6. $U_{eq}$ is defined as 1/3 of the trace of the orthogonalised $U_{IJ}$ tensor.**

| Atom | *x* | *y* | *z* | U(eq) |
|---|---|---|---|---|
| Ru1 | 8202.6(2) | 1082.6(2) | 4654.1(2) | 12.21(7) |
| Cl1 | 9937.7(4) | 1544.3(3) | 4487.8(4) | 14.25(12) |
| Cl2 | 8911.3(5) | 1291.1(4) | 6294.6(4) | 21.17(14) |
| P1 | 7701.9(5) | 2348.2(3) | 4475.7(4) | 12.64(13) |
| P2 | 9195.3(5) | 4245.2(3) | 6304.0(4) | 13.72(13) |
| C17 | 7616(2) | 2706.1(13) | 3331.9(17) | 15.0(5) |
| C18 | 8535(2) | 3050.9(14) | 3164.0(18) | 16.6(5) |
| C24 | 8459(2) | 3800.4(13) | 5187.9(18) | 15.1(5) |
| C5 | 8505(2) | -84.6(14) | 4247.0(19) | 18.9(5) |
| C11 | 6390(2) | 2590.9(15) | 4704.0(19) | 18.7(5) |
| C22 | 6669(2) | 2593.6(14) | 2593.5(19) | 18.8(5) |
| C3 | 7017(2) | 726.5(14) | 3368.5(18) | 17.9(5) |
| C21 | 6636(2) | 2834.7(14) | 1713.1(19) | 22.1(6) |
| C1 | 7014(2) | 264.1(15) | 4910(2) | 21.9(5) |
| C19 | 8497(2) | 3285.1(15) | 2279.2(19) | 21.7(5) |
| C36 | 8853(2) | 5564.3(14) | 5229.9(18) | 17.2(5) |

| | | | | |
|---|---|---|---|---|
| C31 | 8867(2) | 5233.8(14) | 6070.4(18) | 15.5(5) |
| C32 | 8710(2) | 5697.7(15) | 6770(2) | 22.5(6) |
| C30 | 8545(3) | 3493.4(15) | 7682(2) | 24.0(6) |
| C20 | 7554(3) | 3175.5(15) | 1552.6(19) | 24.1(6) |
| C34 | 8511(2) | 6771.7(15) | 5791(2) | 23.6(6) |
| C6 | 8015(2) | -126.6(14) | 4955(2) | 20.6(5) |
| C25 | 8224(2) | 4000.2(14) | 6958.4(19) | 17.3(5) |
| C23 | 8672(2) | 2960.7(14) | 5289.0(17) | 15.1(5) |
| C2 | 6519(2) | 675.1(14) | 4101.3(19) | 19.0(5) |
| C35 | 8681(2) | 6326.3(15) | 5094(2) | 21.1(5) |
| C4 | 8036(2) | 363.9(14) | 3441.5(18) | 17.1(5) |
| C28 | 6757(3) | 3560.7(18) | 7948(2) | 33.8(7) |
| C29 | 7812(3) | 3276.9(17) | 8175(2) | 32.6(7) |
| C33 | 8522(3) | 6457.1(16) | 6626(2) | 26.2(6) |
| C10 | 8385(3) | 1101.8(16) | 2133(2) | 26.4(6) |
| C16 | 5627(2) | 3099.8(16) | 4181(2) | 24.9(6) |
| C26 | 7143(2) | 4282.8(16) | 6733(2) | 24.5(6) |
| C7 | 6511(3) | 231.2(18) | 5710(2) | 31.7(7) |
| C8 | 8632(2) | 399.7(15) | 2706.9(19) | 19.8(5) |
| C12 | 6201(2) | 2278.4(17) | 5494(2) | 26.1(6) |
| C13 | 5262(3) | 2468(2) | 5747(2) | 34.2(7) |
| C27 | 6418(3) | 4067.9(19) | 7225(2) | 31.8(7) |
| C14 | 4493(3) | 2959(2) | 5216(3) | 35.4(8) |
| C15 | 4675(3) | 3276.7(19) | 4439(3) | 33.4(7) |
| C9 | 8338(3) | -296.6(17) | 2099(2) | 36.2(8) |

**Table S26. Anisotropic Displacement Parameters (Å$^2$×10$^3$) for complex 6. The Anisotropic displacement factor exponent takes the form: $-2\pi^2[h^2a^{*2}U_{11}+2hka^*b^*U_{12}+\ldots]$.**

| **Atom** | **$U_{11}$** | **$U_{22}$** | **$U_{33}$** | **$U_{23}$** | **$U_{13}$** | **$U_{12}$** |
|---|---|---|---|---|---|---|
| Ru1 | 14.09(11) | 6.66(10) | 15.31(11) | 0.41(6) | 3.24(7) | 0.13(6) |
| Cl1 | 14.9(3) | 9.9(2) | 17.8(3) | -0.7(2) | 4.5(2) | 0.41(19) |
| Cl2 | 29.0(3) | 17.8(3) | 16.3(3) | 2.3(2) | 5.6(2) | -1.4(2) |
| P1 | 13.7(3) | 7.7(3) | 16.5(3) | -1.1(2) | 4.2(2) | 0.5(2) |

| | | | | | | |
|---|---|---|---|---|---|---|
| P2 | 16.9(3) | 9.4(3) | 15.5(3) | -1.1(2) | 5.7(2) | -0.6(2) |
| C17 | 18.7(11) | 7.3(10) | 18.0(12) | -1.3(9) | 3.7(9) | 3.9(9) |
| C18 | 19.6(12) | 10.4(11) | 19.0(12) | -1.7(9) | 4.4(10) | 2.5(9) |
| C24 | 20.4(12) | 9.8(11) | 15.1(11) | -0.8(9) | 5.2(9) | 0.9(9) |
| C5 | 22.4(13) | 6.8(11) | 24.2(13) | -3.9(9) | 1.3(10) | 1.6(9) |
| C11 | 16.7(12) | 14.8(12) | 26.1(13) | -5.3(10) | 8.6(10) | -1.2(9) |
| C22 | 19.8(12) | 9.3(11) | 24.8(13) | -2.1(10) | 2.1(10) | 1.9(9) |
| C3 | 17.1(12) | 12.5(11) | 19.9(12) | -2.6(9) | -1.7(10) | -2.8(9) |
| C21 | 27.9(14) | 10.7(11) | 20.6(13) | -2.8(10) | -4.8(10) | 6.8(10) |
| C1 | 24.0(13) | 12.3(12) | 29.1(14) | 1.8(10) | 7.2(11) | -7.0(10) |
| C19 | 29.9(14) | 15.1(12) | 21.6(13) | 0.7(10) | 9.9(11) | 1.5(10) |
| C36 | 18.7(12) | 11.8(11) | 21.6(13) | -1.3(9) | 6.4(10) | -2.0(9) |
| C31 | 14.9(11) | 12.8(11) | 19.2(12) | -1.9(9) | 5.5(9) | -2.9(9) |
| C32 | 32.1(14) | 13.3(12) | 24.6(14) | -3.4(10) | 12.3(11) | -2.4(10) |
| C30 | 35.9(15) | 13.2(12) | 26.9(14) | 0.7(10) | 15.5(12) | 0.2(11) |
| C20 | 40.7(16) | 12.4(12) | 18.2(13) | 2.5(10) | 6.6(11) | 9.0(11) |
| C34 | 20.6(12) | 8.9(11) | 40.5(16) | -1.8(11) | 7.1(11) | -0.9(9) |
| C6 | 28.6(14) | 5.6(11) | 26.8(14) | 3.4(10) | 6.5(11) | -3.9(9) |
| C25 | 24.9(13) | 9.7(11) | 19.7(13) | -3.9(9) | 10.3(10) | -2.6(9) |
| C23 | 17.6(11) | 11.3(11) | 15.9(11) | -1.0(9) | 3.8(9) | -0.2(9) |
| C2 | 15.0(11) | 12.0(11) | 28.0(14) | -1.9(10) | 2.9(10) | -3.7(9) |
| C35 | 20.6(12) | 14.9(12) | 26.9(14) | 2.8(10) | 5.2(11) | -4.1(10) |
| C4 | 22.3(12) | 11.0(11) | 17.1(12) | -5.4(9) | 4.0(10) | -3.8(9) |
| C28 | 49.2(19) | 21.8(15) | 43.4(19) | -2.8(13) | 34.4(16) | -8.6(13) |
| C29 | 53(2) | 19.1(14) | 32.6(16) | 6.8(12) | 24.1(15) | -1.7(13) |
| C33 | 32.6(15) | 14.1(13) | 35.4(16) | -8.7(11) | 15.3(13) | -1.7(11) |
| C10 | 38.3(17) | 20.5(14) | 22.5(14) | 2.7(11) | 12.0(12) | 4.1(11) |
| C16 | 21.0(13) | 17.1(13) | 37.1(16) | -1.1(11) | 9.1(12) | 3.0(10) |
| C26 | 24.9(14) | 22.0(14) | 29.3(15) | 1.2(11) | 12.0(11) | 0.3(11) |
| C7 | 37.7(17) | 26.4(15) | 36.2(17) | 7.0(13) | 19.0(14) | -5.6(13) |
| C8 | 25.4(13) | 14.8(12) | 19.5(12) | -0.9(10) | 6.5(10) | -0.1(10) |
| C12 | 26.3(14) | 23.9(14) | 30.8(15) | -1.0(12) | 12.4(12) | 0.1(11) |

| | | | | | | |
|---|---|---|---|---|---|---|
| C13 | 31.9(16) | 41.0(19) | 35.3(17) | -8.5(14) | 18.6(14) | -3.9(14) |
| C27 | 29.5(15) | 28.8(16) | 43.6(19) | -3.3(14) | 20.8(14) | -1.6(12) |
| C14 | 20.9(14) | 42.6(19) | 45.9(19) | -17.4(16) | 14.8(13) | 0.4(13) |
| C15 | 22.0(14) | 28.7(16) | 48(2) | -9.3(14) | 7.8(13) | 7.0(12) |
| C9 | 69(2) | 16.0(14) | 28.6(16) | -6.1(12) | 21.9(16) | -5.1(14) |

**Table S27. Bond Lengths for complex 6.**

| Atom | Atom | Length/Å | Atom | Atom | Length/Å |
|---|---|---|---|---|---|
| Ru1 | Cl1 | 2.4157(6) | C3 | C4 | 1.415(4) |
| Ru1 | Cl2 | 2.4157(6) | C21 | C20 | 1.388(4) |
| Ru1 | P1 | 2.3537(6) | C1 | C6 | 1.428(4) |
| Ru1 | C5 | 2.248(2) | C1 | C2 | 1.413(4) |
| Ru1 | C3 | 2.185(2) | C1 | C7 | 1.517(4) |
| Ru1 | C1 | 2.209(3) | C19 | C20 | 1.385(4) |
| Ru1 | C6 | 2.245(2) | C36 | C31 | 1.398(4) |
| Ru1 | C2 | 2.174(2) | C36 | C35 | 1.392(4) |
| Ru1 | C4 | 2.203(2) | C31 | C32 | 1.403(4) |
| P1 | C17 | 1.819(3) | C32 | C33 | 1.391(4) |
| P1 | C11 | 1.833(3) | C30 | C25 | 1.392(4) |
| P1 | C23 | 1.834(3) | C30 | C29 | 1.395(4) |
| P2 | C24 | 1.860(3) | C34 | C35 | 1.387(4) |
| P2 | C31 | 1.834(3) | C34 | C33 | 1.378(4) |
| P2 | C25 | 1.831(3) | C25 | C26 | 1.401(4) |
| C17 | C18 | 1.398(4) | C4 | C8 | 1.507(4) |
| C17 | C22 | 1.398(4) | C28 | C29 | 1.373(5) |
| C18 | C19 | 1.390(4) | C28 | C27 | 1.395(5) |
| C24 | C23 | 1.532(3) | C10 | C8 | 1.512(4) |
| C5 | C6 | 1.381(4) | C16 | C15 | 1.399(4) |
| C5 | C4 | 1.440(4) | C26 | C27 | 1.385(4) |
| C11 | C16 | 1.398(4) | C8 | C9 | 1.534(4) |
| C11 | C12 | 1.400(4) | C12 | C13 | 1.384(4) |
| C22 | C21 | 1.389(4) | C13 | C14 | 1.385(5) |

| | | | | | |
|---|---|---|---|---|---|
| C3 | C2 | 1.422(4) | C14 | C15 | 1.382(5) |

**Table S28. Bond Angles for complex 6.**

| Atom | Atom | Atom | Angle/° | Atom | Atom | Atom | Angle/° |
|---|---|---|---|---|---|---|---|
| Cl2 | Ru1 | Cl1 | 88.36(2) | C16 | C11 | P1 | 124.1(2) |
| P1 | Ru1 | Cl1 | 82.97(2) | C16 | C11 | C12 | 119.3(3) |
| P1 | Ru1 | Cl2 | 88.69(2) | C12 | C11 | P1 | 116.4(2) |
| C5 | Ru1 | Cl1 | 93.62(7) | C21 | C22 | C17 | 120.5(3) |
| C5 | Ru1 | Cl2 | 112.56(7) | C2 | C3 | Ru1 | 70.56(14) |
| C5 | Ru1 | P1 | 158.43(7) | C4 | C3 | Ru1 | 71.88(14) |
| C3 | Ru1 | Cl1 | 114.73(7) | C4 | C3 | C2 | 120.0(2) |
| C3 | Ru1 | Cl2 | 156.88(7) | C20 | C21 | C22 | 120.3(3) |
| C3 | Ru1 | P1 | 95.00(7) | C6 | C1 | Ru1 | 72.67(15) |
| C3 | Ru1 | C5 | 67.02(9) | C6 | C1 | C7 | 120.3(3) |
| C3 | Ru1 | C1 | 68.55(10) | C2 | C1 | Ru1 | 69.88(15) |
| C3 | Ru1 | C6 | 79.21(10) | C2 | C1 | C6 | 117.8(3) |
| C3 | Ru1 | C4 | 37.63(10) | C2 | C1 | C7 | 121.9(3) |
| C1 | Ru1 | Cl1 | 157.58(8) | C7 | C1 | Ru1 | 128.4(2) |
| C1 | Ru1 | Cl2 | 89.74(8) | C20 | C19 | C18 | 120.5(3) |
| C1 | Ru1 | P1 | 119.33(8) | C35 | C36 | C31 | 120.9(2) |
| C1 | Ru1 | C5 | 66.61(10) | C36 | C31 | P2 | 122.00(19) |
| C1 | Ru1 | C6 | 37.39(10) | C36 | C31 | C32 | 117.7(2) |
| C6 | Ru1 | Cl1 | 120.20(7) | C32 | C31 | P2 | 120.1(2) |
| C6 | Ru1 | Cl2 | 88.35(8) | C33 | C32 | C31 | 121.2(3) |
| C6 | Ru1 | P1 | 156.53(8) | C25 | C30 | C29 | 120.9(3) |
| C6 | Ru1 | C5 | 35.80(11) | C19 | C20 | C21 | 119.6(3) |
| C2 | Ru1 | Cl1 | 152.62(8) | C33 | C34 | C35 | 119.7(3) |
| C2 | Ru1 | Cl2 | 118.92(8) | C5 | C6 | Ru1 | 72.21(14) |
| C2 | Ru1 | P1 | 94.61(7) | C5 | C6 | C1 | 121.2(3) |
| C2 | Ru1 | C5 | 78.66(10) | C1 | C6 | Ru1 | 69.94(14) |
| C2 | Ru1 | C3 | 38.09(10) | C30 | C25 | P2 | 119.5(2) |
| C2 | Ru1 | C1 | 37.59(10) | C30 | C25 | C26 | 118.3(3) |

| | | | | | | | |
|---|---|---|---|---|---|---|---|
| C2 | Ru1 | C6 | 66.76(10) | C26 | C25 | P2 | 122.2(2) |
| C2 | Ru1 | C4 | 68.29(10) | C24 | C23 | P1 | 117.33(18) |
| C4 | Ru1 | Cl1 | 89.45(7) | C3 | C2 | Ru1 | 71.36(14) |
| C4 | Ru1 | Cl2 | 149.97(7) | C1 | C2 | Ru1 | 72.53(15) |
| C4 | Ru1 | P1 | 120.73(7) | C1 | C2 | C3 | 121.6(2) |
| C4 | Ru1 | C5 | 37.74(10) | C34 | C35 | C36 | 120.4(3) |
| C4 | Ru1 | C1 | 81.14(10) | C5 | C4 | Ru1 | 72.80(14) |
| C4 | Ru1 | C6 | 67.12(10) | C5 | C4 | C8 | 118.4(2) |
| C17 | P1 | Ru1 | 113.09(8) | C3 | C4 | Ru1 | 70.49(14) |
| C17 | P1 | C11 | 106.28(12) | C3 | C4 | C5 | 118.0(2) |
| C17 | P1 | C23 | 105.94(12) | C3 | C4 | C8 | 123.5(2) |
| C11 | P1 | Ru1 | 115.75(9) | C8 | C4 | Ru1 | 129.65(18) |
| C11 | P1 | C23 | 101.30(12) | C29 | C28 | C27 | 119.9(3) |
| C23 | P1 | Ru1 | 113.35(8) | C28 | C29 | C30 | 120.2(3) |
| C31 | P2 | C24 | 102.34(11) | C34 | C33 | C32 | 120.2(3) |
| C25 | P2 | C24 | 98.59(12) | C11 | C16 | C15 | 119.7(3) |
| C25 | P2 | C31 | 100.82(12) | C27 | C26 | C25 | 120.7(3) |
| C18 | C17 | P1 | 119.96(19) | C4 | C8 | C10 | 113.2(2) |
| C18 | C17 | C22 | 118.8(2) | C4 | C8 | C9 | 108.7(2) |
| C22 | C17 | P1 | 121.1(2) | C10 | C8 | C9 | 111.1(2) |
| C19 | C18 | C17 | 120.3(2) | C13 | C12 | C11 | 120.1(3) |
| C23 | C24 | P2 | 107.71(17) | C12 | C13 | C14 | 120.6(3) |
| C6 | C5 | Ru1 | 72.00(14) | C26 | C27 | C28 | 120.1(3) |
| C6 | C5 | C4 | 121.3(2) | C15 | C14 | C13 | 119.9(3) |
| C4 | C5 | Ru1 | 69.46(14) | C14 | C15 | C16 | 120.4(3) |

**Table S29. Torsion Angles for complex 6.**

| A | B | C | D | Angle/˚ | A | B | C | D | Angle/˚ |
|---|---|---|---|---|---|---|---|---|---|
| Ru1 | P1 | C17 | C18 | -93.16(19) | C31 | P2 | C24 | C23 | 179.81(17) |
| Ru1 | P1 | C17 | C22 | 82.0(2) | C31 | P2 | C25 | C30 | -143.9(2) |
| Ru1 | P1 | C11 | C16 | -140.0(2) | C31 | P2 | C25 | C26 | 38.8(2) |
| Ru1 | P1 | C11 | C12 | 44.7(2) | C31 | C36 | C35 | C34 | -0.5(4) |

| | | | | | | | | | |
|---|---|---|---|---|---|---|---|---|---|
| Ru1 | P1 | C23 | C24 | 176.19(16) | C31 | C32 | C33 | C34 | -1.5(4) |
| Ru1 | C5 | C6 | C1 | 52.0(2) | C30 | C25 | C26 | C27 | 0.6(4) |
| Ru1 | C5 | C4 | C3 | -55.9(2) | C6 | C5 | C4 | Ru1 | 52.1(2) |
| Ru1 | C5 | C4 | C8 | 126.3(2) | C6 | C5 | C4 | C3 | -3.8(4) |
| Ru1 | C3 | C2 | C1 | -54.6(2) | C6 | C5 | C4 | C8 | 178.4(2) |
| Ru1 | C3 | C4 | C5 | 57.1(2) | C6 | C1 | C2 | Ru1 | -56.5(2) |
| Ru1 | C3 | C4 | C8 | -125.3(2) | C6 | C1 | C2 | C3 | -2.5(4) |
| Ru1 | C1 | C6 | C5 | -53.0(2) | C25 | P2 | C24 | C23 | -77.04(19) |
| Ru1 | C1 | C2 | C3 | 54.1(2) | C25 | P2 | C31 | C36 | -141.9(2) |
| Ru1 | C4 | C8 | C10 | -64.3(3) | C25 | P2 | C31 | C32 | 43.5(2) |
| Ru1 | C4 | C8 | C9 | 171.7(2) | C25 | C30 | C29 | C28 | 0.2(5) |
| P1 | C17 | C18 | C19 | 176.07(19) | C25 | C26 | C27 | C28 | -0.5(5) |
| P1 | C17 | C22 | C21 | -176.53(19) | C23 | P1 | C17 | C18 | 31.6(2) |
| P1 | C11 | C16 | C15 | -176.7(2) | C23 | P1 | C17 | C22 | -153.3(2) |
| P1 | C11 | C12 | C13 | 176.2(2) | C23 | P1 | C11 | C16 | 96.9(2) |
| P2 | C24 | C23 | P1 | 159.47(13) | C23 | P1 | C11 | C12 | -78.4(2) |
| P2 | C31 | C32 | C33 | 176.3(2) | C2 | C3 | C4 | Ru1 | -53.6(2) |
| P2 | C25 | C26 | C27 | 178.0(2) | C2 | C3 | C4 | C5 | 3.4(3) |
| C17 | P1 | C11 | C16 | -13.6(3) | C2 | C3 | C4 | C8 | -178.9(2) |
| C17 | P1 | C11 | C12 | 171.1(2) | C2 | C1 | C6 | Ru1 | 55.1(2) |
| C17 | P1 | C23 | C24 | 51.6(2) | C2 | C1 | C6 | C5 | 2.1(4) |
| C17 | C18 | C19 | C20 | -0.5(4) | C35 | C36 | C31 | P2 | -175.3(2) |
| C17 | C22 | C21 | C20 | 1.5(4) | C35 | C36 | C31 | C32 | -0.5(4) |
| C18 | C17 | C22 | C21 | -1.3(4) | C35 | C34 | C33 | C32 | 0.4(4) |
| C18 | C19 | C20 | C21 | 0.6(4) | C4 | C5 | C6 | Ru1 | -51.0(2) |
| C24 | P2 | C31 | C36 | -40.5(2) | C4 | C5 | C6 | C1 | 1.0(4) |
| C24 | P2 | C31 | C32 | 144.9(2) | C4 | C3 | C2 | Ru1 | 54.3(2) |
| C24 | P2 | C25 | C30 | 111.7(2) | C4 | C3 | C2 | C1 | -0.3(4) |
| C24 | P2 | C25 | C26 | -65.6(2) | C29 | C30 | C25 | P2 | -177.9(2) |
| C5 | C4 | C8 | C10 | -155.0(2) | C29 | C30 | C25 | C26 | -0.5(4) |
| C5 | C4 | C8 | C9 | 81.0(3) | C29 | C28 | C27 | C26 | 0.2(5) |
| C11 | P1 | C17 | C18 | 138.8(2) | C33 | C34 | C35 | C36 | 0.5(4) |

| | | | | | | | | | |
|---|---|---|---|---|---|---|---|---|---|
| C11 | P1 | C17 | C22 | -46.1(2) | C16 | C11 | C12 | C13 | 0.6(4) |
| C11 | P1 | C23 | C24 | -59.1(2) | C7 | C1 | C6 | Ru1 | -124.9(3) |
| C11 | C16 | C15 | C14 | 0.9(5) | C7 | C1 | C6 | C5 | -177.9(3) |
| C11 | C12 | C13 | C14 | 0.8(5) | C7 | C1 | C2 | Ru1 | 123.5(3) |
| C22 | C17 | C18 | C19 | 0.8(4) | C7 | C1 | C2 | C3 | 177.5(3) |
| C22 | C21 | C20 | C19 | -1.1(4) | C12 | C11 | C16 | C15 | -1.5(4) |
| C3 | C4 | C8 | C10 | 27.3(4) | C12 | C13 | C14 | C15 | -1.4(5) |
| C3 | C4 | C8 | C9 | -96.7(3) | C13 | C14 | C15 | C16 | 0.5(5) |
| C36 | C31 | C32 | C33 | 1.4(4) | C27 | C28 | C29 | C30 | -0.1(5) |

**Table S30. Hydrogen Atom Coordinates (Å×10[4]) and Isotropic Displacement Parameters (Å[2]×10[3]) for complex 6.**

| Atom | *x* | *y* | *z* | U(eq) |
|---|---|---|---|---|
| H18 | 9188.05 | 3125.52 | 3656.66 | 20 |
| H24A | 7655.23 | 3902.93 | 5036.33 | 18 |
| H24B | 8738.6 | 4001.11 | 4686.22 | 18 |
| H5 | 9165.08 | -357.14 | 4292.01 | 23 |
| H22 | 6043.8 | 2350.42 | 2694.67 | 23 |
| H3 | 6666.03 | 1004.16 | 2830.32 | 22 |
| H21 | 5982.97 | 2766.1 | 1218.72 | 27 |
| H19 | 9124.05 | 3522.16 | 2171.99 | 26 |
| H36 | 8962.28 | 5264.7 | 4745.22 | 21 |
| H32 | 8732.77 | 5489.25 | 7352.52 | 27 |
| H30 | 9272.14 | 3292.4 | 7842.36 | 29 |
| H20 | 7534.69 | 3332.59 | 948.31 | 29 |
| H34 | 8387.92 | 7291 | 5694.66 | 28 |
| H6 | 8350.99 | -420.69 | 5482.55 | 25 |
| H23A | 8674.24 | 2815.39 | 5921.15 | 18 |
| H23B | 9423.78 | 2864.12 | 5231.95 | 18 |
| H2 | 5836.62 | 923.16 | 4045.83 | 23 |
| H35 | 8679.47 | 6542.76 | 4520.3 | 25 |
| H28 | 6259.04 | 3411.86 | 8283.97 | 41 |
| H29 | 8044.24 | 2931.98 | 8668.29 | 39 |

| | | | | |
|---|---|---|---|---|
| H33 | 8400.91 | 6759.8 | 7103.15 | 31 |
| H10A | 7587.68 | 1133.37 | 1830.01 | 40 |
| H10B | 8786.28 | 1090.64 | 1664.23 | 40 |
| H10C | 8620.49 | 1536.5 | 2532.03 | 40 |
| H16 | 5755.04 | 3324.77 | 3651.2 | 30 |
| H26 | 6904.97 | 4625.4 | 6237.55 | 29 |
| H7A | 7074.49 | 364.61 | 6282.11 | 47 |
| H7B | 6243.56 | -274.35 | 5764.28 | 47 |
| H7C | 5891.97 | 582.2 | 5602.02 | 47 |
| H8 | 9445.41 | 387.3 | 3020.1 | 24 |
| H12 | 6718.63 | 1935.95 | 5857.87 | 31 |
| H13 | 5142.96 | 2258.76 | 6287.97 | 41 |
| H27 | 5689.44 | 4266.32 | 7068.74 | 38 |
| H14 | 3842.32 | 3077.73 | 5386.48 | 42 |
| H15 | 4151.13 | 3616.89 | 4079.04 | 40 |
| H9A | 8566 | -742.24 | 2479.78 | 54 |
| H9B | 8722.38 | -280.83 | 1619.72 | 54 |
| H9C | 7536.23 | -311.69 | 1810.42 | 54 |

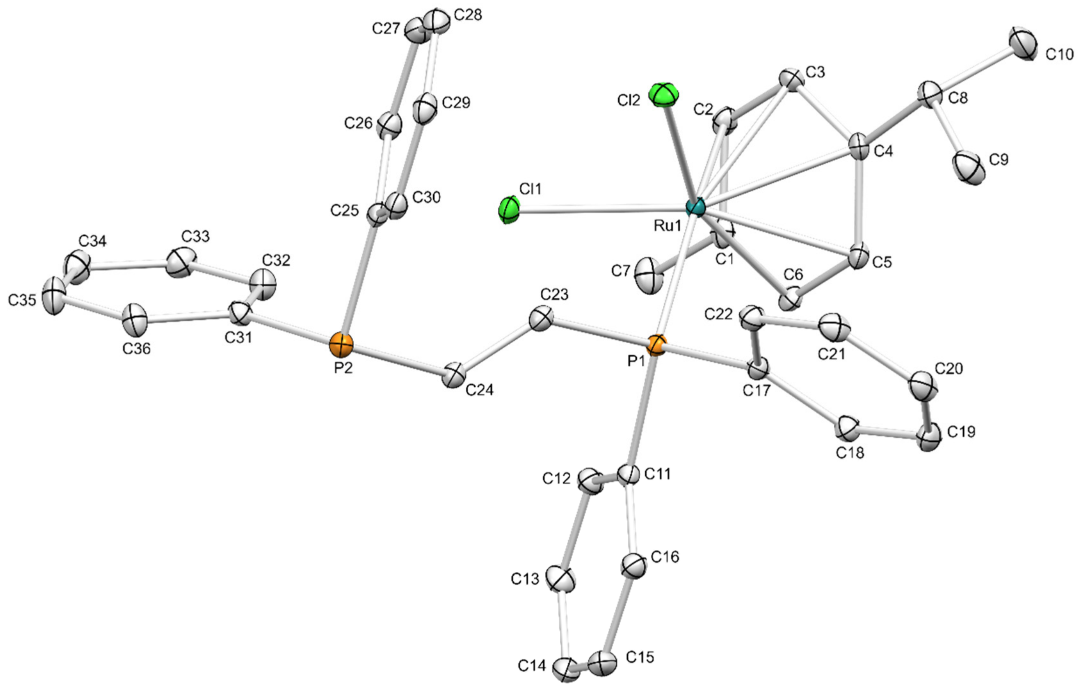


**Figure S73**. ORTEP representation of **7**, with complete atom labellings, in the solid state determined by single crystal X-ray diffraction analysis. Thermal ellipsoids represented at the 30% probability level. H atoms omitted for clarity.

**Table S31. Crystal data and structure refinement for complex 7.**

| | |
|---|---|
| Empirical formula | $C_{72}H_{72}Cl_4P_4Ru_2$ |
| Formula weight | 1405.11 |
| Temperature/K | 101(1) |
| Crystal system | monoclinic |
| Space group | $P2_1/c$ |
| a/Å | 9.93542(4) |
| b/Å | 15.54235(7) |
| c/Å | 20.93213(10) |
| α/° | 90 |
| β/° | 98.6808(4) |
| γ/° | 90 |
| Volume/Å$^3$ | 3195.31(3) |
| Z | 2 |
| $\rho_{calc}$g/cm$^3$ | 1.460 |
| μ/mm$^{-1}$ | 6.635 |

| | |
|---|---|
| F(000) | 1440.0 |
| Crystal size/mm$^3$ | 0.19 × 0.13 × 0.05 |
| Radiation | Cu K$\alpha$ ($\lambda$ = 1.54184) |
| 2Θ range for data collection/° | 8.546 to 160.628 |
| Index ranges | -11 ≤ h ≤ 12, -19 ≤ k ≤ 18, -26 ≤ l ≤ 26 |
| Reflections collected | 71850 |
| Independent reflections | 6989 [$R_{int}$ = 0.0473, $R_{sigma}$ = 0.0233] |
| Data/restraints/parameters | 6989/0/374 |
| Goodness-of-fit on F$^2$ | 1.057 |
| Final R indexes [I>=2σ (I)] | $R_1$ = 0.0261, w$R_2$ = 0.0649 |
| Final R indexes [all data] | $R_1$ = 0.0264, w$R_2$ = 0.0651 |
| Largest diff. peak/hole / e Å$^{-3}$ | 0.84/-0.49 |

**Table S32. Fractional Atomic Coordinates (×10$^4$) and Equivalent Isotropic Displacement Parameters (Å$^2$×10$^3$) for complex 7. $U_{eq}$ is defined as 1/3 of the trace of the orthogonalised $U_{IJ}$ tensor.**

| Atom | *x* | *y* | *z* | U(eq) |
|---|---|---|---|---|
| Ru1 | 5726.6(2) | 4757.3(2) | 3754.0(2) | 13.52(5) |
| Cl1 | 3641.5(4) | 4266.1(3) | 4081.3(2) | 19.19(9) |
| Cl2 | 6936.5(4) | 3694.6(3) | 4457.8(2) | 20.89(9) |
| P1 | 5350.7(4) | 3623.7(3) | 3006.2(2) | 14.14(9) |
| P2 | 3259.4(4) | 1205.2(3) | 3564.5(2) | 17.42(9) |
| C24 | 3693.9(18) | 2206.9(11) | 3189.7(8) | 17.9(3) |
| C22 | 7691.1(18) | 2629.3(12) | 2972.0(9) | 20.0(3) |
| C17 | 6871.6(17) | 3289.8(11) | 2677.2(8) | 16.4(3) |
| C4 | 7603.8(17) | 5540.3(12) | 3815.6(9) | 18.5(3) |
| C8 | 9096.6(19) | 5301.2(13) | 3983.2(10) | 23.7(4) |
| C12 | 2978.7(18) | 4339.6(12) | 2339.9(9) | 20.9(3) |
| C7 | 3314(2) | 6284.7(13) | 3462.3(11) | 26.9(4) |
| C1 | 4792.7(18) | 6040.0(11) | 3574.3(9) | 19.7(3) |
| C23 | 4839.5(18) | 2638.6(11) | 3364.7(8) | 17.7(3) |
| C5 | 6896.6(18) | 5566.9(11) | 3179.1(9) | 18.8(3) |
| C21 | 8915.8(19) | 2424.7(13) | 2760.6(9) | 23.9(4) |
| C16 | 4004.3(19) | 3249.5(12) | 1752.2(9) | 20.7(3) |

| | | | | |
|---|---|---|---|---|
| C31 | 1794.2(17) | 1544.9(12) | 3942.0(9) | 18.8(3) |
| C36 | 905.1(18) | 900.8(12) | 4088.9(10) | 23.1(4) |
| C25 | 4623.0(17) | 1132.1(11) | 4257.1(8) | 17.1(3) |
| C14 | 1879.9(19) | 3896.9(14) | 1284.9(9) | 25.3(4) |
| C6 | 5495.5(18) | 5818.4(11) | 3058.8(9) | 19.3(3) |
| C32 | 1516.6(19) | 2398.0(12) | 4080.1(9) | 23.1(4) |
| C19 | 8523.8(19) | 3535.8(14) | 1960.6(9) | 24.8(4) |
| C15 | 2926(2) | 3313.1(14) | 1246.9(9) | 25.1(4) |
| C35 | -214(2) | 1107.7(14) | 4385.1(11) | 28.3(4) |
| C3 | 6889.2(18) | 5789.5(12) | 4332.7(9) | 19.6(3) |
| C33 | 374(2) | 2600.5(13) | 4363.5(10) | 26.0(4) |
| C11 | 4037.8(17) | 3763.4(11) | 2300.8(8) | 16.8(3) |
| C30 | 5537.2(18) | 449.4(12) | 4265.1(9) | 19.4(3) |
| C18 | 7293.0(18) | 3743.7(12) | 2164.4(9) | 21.0(3) |
| C28 | 6763.9(19) | 961.4(14) | 5269.3(9) | 24.8(4) |
| C27 | 5869(2) | 1646.8(13) | 5262.3(9) | 24.7(4) |
| C2 | 5520.2(19) | 6030.9(11) | 4218.3(9) | 20.3(3) |
| C29 | 6602.5(19) | 360.5(13) | 4775.1(10) | 22.4(4) |
| C13 | 1907.6(19) | 4409.6(14) | 1829.8(10) | 25.8(4) |
| C26 | 4796.2(19) | 1732.2(12) | 4760.2(9) | 21.1(4) |
| C10 | 9930(2) | 6129.0(17) | 4119.4(12) | 36.0(5) |
| C34 | -484(2) | 1958.7(14) | 4521.0(10) | 27.7(4) |
| C20 | 9335.8(19) | 2880.7(14) | 2258.3(10) | 26.0(4) |
| C9 | 9646(2) | 4757.1(14) | 3476.0(12) | 30.9(5) |

**Table S33. Anisotropic Displacement Parameters (Å$^2$×10$^3$) for complex 7. The Anisotropic displacement factor exponent takes the form: $-2\pi^2[h^2a^{*2}U_{11}+2hka^*b^*U_{12}+\ldots]$.**

| Atom | $U_{11}$ | $U_{22}$ | $U_{33}$ | $U_{23}$ | $U_{13}$ | $U_{12}$ |
|---|---|---|---|---|---|---|
| Ru1 | 12.70(7) | 16.17(8) | 12.08(7) | 1.24(4) | 3.18(5) | -0.15(4) |
| Cl1 | 18.02(18) | 21.65(19) | 19.77(19) | -0.25(15) | 8.94(14) | -2.11(14) |
| Cl2 | 21.24(19) | 21.18(19) | 18.96(19) | 3.11(15) | -1.19(15) | 1.77(15) |
| P1 | 13.09(18) | 16.57(19) | 13.12(19) | 1.37(15) | 3.14(14) | 0.32(14) |
| P2 | 16.8(2) | 18.5(2) | 17.3(2) | 0.50(16) | 3.58(15) | -1.84(15) |

| | | | | | | |
|---|---|---|---|---|---|---|
| C24 | 18.3(8) | 19.0(8) | 17.1(8) | 1.7(6) | 4.9(6) | -0.2(6) |
| C22 | 18.1(8) | 23.1(9) | 18.5(8) | -0.3(7) | 1.7(6) | 0.9(7) |
| C17 | 14.3(7) | 19.9(8) | 15.1(8) | -2.1(6) | 2.8(6) | 0.1(6) |
| C4 | 15.5(8) | 20.6(8) | 20.0(8) | 0.9(7) | 4.3(6) | -6.0(6) |
| C8 | 16.5(8) | 33.3(10) | 21.4(9) | 1.8(7) | 3.0(7) | -2.8(7) |
| C12 | 17.6(8) | 24.6(9) | 20.2(8) | -0.1(7) | 1.6(7) | 1.1(7) |
| C7 | 20.9(9) | 23.0(9) | 37.6(11) | 4.5(8) | 6.3(8) | 3.5(7) |
| C1 | 19.5(8) | 14.1(8) | 26.2(9) | 3.5(7) | 5.9(7) | 0.6(6) |
| C23 | 18.9(8) | 19.0(8) | 15.9(8) | 2.5(6) | 4.5(6) | 1.2(6) |
| C5 | 19.5(8) | 20.0(8) | 18.4(8) | 2.3(7) | 7.2(6) | -4.4(6) |
| C21 | 18.6(8) | 27.6(9) | 24.8(9) | -2.8(7) | 0.8(7) | 5.7(7) |
| C16 | 20.0(8) | 24.7(9) | 17.5(8) | 0.1(7) | 3.6(7) | 0.6(7) |
| C31 | 15.0(8) | 21.6(8) | 19.5(8) | 1.6(7) | 1.6(6) | -0.7(6) |
| C36 | 17.4(8) | 20.4(9) | 31.8(10) | 3.2(7) | 4.4(7) | -0.5(7) |
| C25 | 16.4(8) | 19.0(8) | 16.7(8) | 3.6(6) | 5.1(6) | -2.4(6) |
| C14 | 18.7(8) | 36.5(10) | 19.4(9) | 4.8(8) | -1.6(7) | -4.5(8) |
| C6 | 21.6(8) | 18.9(8) | 17.4(8) | 5.7(6) | 3.1(6) | -2.1(6) |
| C32 | 21.4(9) | 22.7(9) | 25.9(9) | 1.1(7) | 6.0(7) | -1.9(7) |
| C19 | 19.2(9) | 36.2(10) | 20.4(9) | 0.6(8) | 7.0(7) | -1.7(7) |
| C15 | 24.3(9) | 33.9(10) | 16.9(8) | -2.5(7) | 2.7(7) | -5.3(8) |
| C35 | 19.3(9) | 28.4(10) | 38.8(11) | 7.6(8) | 9.6(8) | -2.5(7) |
| C3 | 21.5(8) | 19.9(8) | 17.4(8) | -2.8(7) | 3.0(6) | -5.5(7) |
| C33 | 27.4(9) | 25.8(9) | 25.6(9) | -0.7(8) | 6.7(8) | 4.4(8) |
| C11 | 15.2(7) | 19.0(8) | 16.2(8) | 3.3(6) | 2.3(6) | -1.8(6) |
| C30 | 19.3(8) | 21.0(8) | 19.5(8) | 0.6(7) | 8.2(7) | -1.5(7) |
| C18 | 17.1(8) | 26.0(9) | 19.9(8) | 2.4(7) | 2.6(6) | -0.2(7) |
| C28 | 19.8(8) | 34.1(10) | 19.9(9) | 5.8(8) | 1.4(7) | -1.9(7) |
| C27 | 28.4(9) | 27.2(9) | 18.6(8) | -1.6(7) | 4.1(7) | -3.4(8) |
| C2 | 23.9(9) | 17.0(8) | 21.7(9) | -2.1(7) | 9.2(7) | -1.6(7) |
| C29 | 17.4(8) | 25.4(9) | 25.8(9) | 5.1(7) | 7.3(7) | 1.9(7) |
| C13 | 17.4(8) | 32.5(10) | 26.5(10) | 2.7(8) | -0.5(7) | 3.4(7) |
| C26 | 22.2(8) | 21.7(9) | 20.6(8) | 0.3(7) | 6.8(7) | 0.2(7) |

| | | | | | | |
|---|---|---|---|---|---|---|
| C10 | 22.7(10) | 47.9(13) | 38.2(12) | -13.4(10) | 7.2(9) | -12.5(9) |
| C34 | 19.2(9) | 35.1(11) | 30.2(10) | 4.1(8) | 8.4(7) | 4.8(8) |
| C20 | 15.9(8) | 36.6(11) | 26.2(9) | -7.6(8) | 5.3(7) | 1.5(7) |
| C9 | 18.6(9) | 35.7(11) | 38.0(12) | -7.4(9) | 3.1(8) | 1.5(8) |

**Table S34. Bond Lengths for complex 7.**

| Atom | Atom | Length/Å | Atom | Atom | Length/Å |
|---|---|---|---|---|---|
| Ru1 | Cl1 | 2.4027(4) | C12 | C13 | 1.393(3) |
| Ru1 | Cl2 | 2.4103(4) | C7 | C1 | 1.502(3) |
| Ru1 | P1 | 2.3490(4) | C1 | C6 | 1.414(3) |
| Ru1 | C4 | 2.2144(17) | C1 | C2 | 1.430(3) |
| Ru1 | C1 | 2.2071(17) | C5 | C6 | 1.431(3) |
| Ru1 | C5 | 2.1920(17) | C21 | C20 | 1.384(3) |
| Ru1 | C6 | 2.1884(17) | C16 | C15 | 1.391(3) |
| Ru1 | C3 | 2.2254(17) | C16 | C11 | 1.395(3) |
| Ru1 | C2 | 2.2283(18) | C31 | C36 | 1.400(3) |
| P1 | C17 | 1.8278(17) | C31 | C32 | 1.394(3) |
| P1 | C23 | 1.8113(18) | C36 | C35 | 1.390(3) |
| P1 | C11 | 1.8296(18) | C25 | C30 | 1.395(3) |
| P2 | C24 | 1.8244(18) | C25 | C26 | 1.398(3) |
| P2 | C31 | 1.8356(18) | C14 | C15 | 1.391(3) |
| P2 | C25 | 1.8323(18) | C14 | C13 | 1.388(3) |
| C24 | C23 | 1.324(3) | C32 | C33 | 1.394(3) |
| C22 | C17 | 1.395(2) | C19 | C18 | 1.393(3) |
| C22 | C21 | 1.393(3) | C19 | C20 | 1.387(3) |
| C17 | C18 | 1.400(2) | C35 | C34 | 1.387(3) |
| C4 | C8 | 1.518(3) | C3 | C2 | 1.396(3) |
| C4 | C5 | 1.410(3) | C33 | C34 | 1.384(3) |
| C4 | C3 | 1.435(2) | C30 | C29 | 1.392(3) |
| C8 | C10 | 1.533(3) | C28 | C27 | 1.386(3) |
| C8 | C9 | 1.521(3) | C28 | C29 | 1.385(3) |
| C12 | C11 | 1.394(3) | C27 | C26 | 1.386(3) |

**Table S35. Bond Angles for complex 7.**

| Atom | Atom | Atom | Angle/° | Atom | Atom | Atom | Angle/° |
|---|---|---|---|---|---|---|---|
| Cl1 | Ru1 | Cl2 | 88.846(15) | C5 | C4 | C3 | 118.18(16) |
| P1 | Ru1 | Cl1 | 83.944(14) | C3 | C4 | Ru1 | 71.57(10) |
| P1 | Ru1 | Cl2 | 84.707(15) | C3 | C4 | C8 | 117.99(16) |
| C4 | Ru1 | Cl1 | 155.91(5) | C4 | C8 | C10 | 108.60(17) |
| C4 | Ru1 | Cl2 | 90.17(5) | C4 | C8 | C9 | 114.55(17) |
| C4 | Ru1 | P1 | 119.92(5) | C9 | C8 | C10 | 110.84(17) |
| C4 | Ru1 | C3 | 37.70(7) | C13 | C12 | C11 | 120.08(18) |
| C4 | Ru1 | C2 | 67.58(7) | C7 | C1 | Ru1 | 129.17(13) |
| C1 | Ru1 | Cl1 | 88.69(5) | C6 | C1 | Ru1 | 70.53(10) |
| C1 | Ru1 | Cl2 | 151.79(5) | C6 | C1 | C7 | 121.81(17) |
| C1 | Ru1 | P1 | 122.92(5) | C6 | C1 | C2 | 118.66(16) |
| C1 | Ru1 | C4 | 80.93(7) | C2 | C1 | Ru1 | 71.99(10) |
| C1 | Ru1 | C3 | 67.17(7) | C2 | C1 | C7 | 119.54(17) |
| C1 | Ru1 | C2 | 37.62(7) | C24 | C23 | P1 | 126.62(14) |
| C5 | Ru1 | Cl1 | 152.94(5) | C4 | C5 | Ru1 | 72.20(10) |
| C5 | Ru1 | Cl2 | 118.17(5) | C4 | C5 | C6 | 120.47(16) |
| C5 | Ru1 | P1 | 96.25(5) | C6 | C5 | Ru1 | 70.80(10) |
| C5 | Ru1 | C4 | 37.32(7) | C20 | C21 | C22 | 120.17(18) |
| C5 | Ru1 | C1 | 68.42(7) | C15 | C16 | C11 | 120.35(17) |
| C5 | Ru1 | C3 | 67.07(7) | C36 | C31 | P2 | 117.17(14) |
| C5 | Ru1 | C2 | 79.68(7) | C32 | C31 | P2 | 123.90(14) |
| C6 | Ru1 | Cl1 | 114.87(5) | C32 | C31 | C36 | 118.92(17) |
| C6 | Ru1 | Cl2 | 156.28(5) | C35 | C36 | C31 | 120.43(18) |
| C6 | Ru1 | P1 | 97.58(5) | C30 | C25 | P2 | 117.32(14) |
| C6 | Ru1 | C4 | 68.13(7) | C30 | C25 | C26 | 119.50(17) |
| C6 | Ru1 | C1 | 37.51(7) | C26 | C25 | P2 | 123.17(14) |
| C6 | Ru1 | C5 | 38.14(7) | C13 | C14 | C15 | 119.94(17) |
| C6 | Ru1 | C3 | 79.19(7) | C1 | C6 | Ru1 | 71.96(10) |
| C6 | Ru1 | C2 | 67.25(7) | C1 | C6 | C5 | 120.77(16) |
| C3 | Ru1 | Cl1 | 118.22(5) | C5 | C6 | Ru1 | 71.06(10) |

| | | | | | | | |
|---|---|---|---|---|---|---|---|
| C3 | Ru1 | Cl2 | 89.50(5) | C31 | C32 | C33 | 120.17(18) |
| C3 | Ru1 | P1 | 157.03(5) | C20 | C19 | C18 | 120.71(18) |
| C3 | Ru1 | C2 | 36.54(7) | C16 | C15 | C14 | 119.95(18) |
| C2 | Ru1 | Cl1 | 91.02(5) | C34 | C35 | C36 | 120.28(18) |
| C2 | Ru1 | Cl2 | 114.36(5) | C4 | C3 | Ru1 | 70.73(10) |
| C2 | Ru1 | P1 | 160.25(5) | C2 | C3 | Ru1 | 71.84(10) |
| C17 | P1 | Ru1 | 113.99(6) | C2 | C3 | C4 | 121.57(17) |
| C17 | P1 | C11 | 105.01(8) | C34 | C33 | C32 | 120.59(19) |
| C23 | P1 | Ru1 | 112.60(6) | C12 | C11 | P1 | 118.78(14) |
| C23 | P1 | C17 | 102.45(8) | C12 | C11 | C16 | 119.47(16) |
| C23 | P1 | C11 | 102.97(8) | C16 | C11 | P1 | 121.45(14) |
| C11 | P1 | Ru1 | 118.10(6) | C29 | C30 | C25 | 120.20(17) |
| C24 | P2 | C31 | 101.20(8) | C19 | C18 | C17 | 119.73(17) |
| C24 | P2 | C25 | 101.45(8) | C29 | C28 | C27 | 120.57(18) |
| C25 | P2 | C31 | 102.53(8) | C26 | C27 | C28 | 120.02(18) |
| C23 | C24 | P2 | 124.23(14) | C1 | C2 | Ru1 | 70.39(10) |
| C21 | C22 | C17 | 120.47(17) | C3 | C2 | Ru1 | 71.62(10) |
| C22 | C17 | P1 | 120.19(13) | C3 | C2 | C1 | 120.32(16) |
| C22 | C17 | C18 | 119.19(16) | C28 | C29 | C30 | 119.65(18) |
| C18 | C17 | P1 | 120.37(13) | C14 | C13 | C12 | 120.19(18) |
| C8 | C4 | Ru1 | 131.61(13) | C27 | C26 | C25 | 120.04(17) |
| C5 | C4 | Ru1 | 70.48(10) | C33 | C34 | C35 | 119.58(18) |
| C5 | C4 | C8 | 123.77(16) | C21 | C20 | C19 | 119.71(17) |

**Table S36. Torsion Angles for complex 7.**

| A | B | C | D | Angle/° | A | B | C | D | Angle/° |
|---|---|---|---|---|---|---|---|---|---|
| Ru1 | P1 | C17 | C22 | 92.12(14) | C5 | C4 | C8 | C10 | 101.1(2) |
| Ru1 | P1 | C17 | C18 | -82.09(15) | C5 | C4 | C8 | C9 | -23.4(3) |
| Ru1 | P1 | C23 | C24 | 120.74(16) | C5 | C4 | C3 | Ru1 | 54.68(14) |
| Ru1 | P1 | C11 | C12 | -24.67(16) | C5 | C4 | C3 | C2 | 1.7(3) |
| Ru1 | P1 | C11 | C16 | 161.56(13) | C21 | C22 | C17 | P1 | -174.10(14) |
| Ru1 | C4 | C8 | C10 | -165.66(15) | C21 | C22 | C17 | C18 | 0.2(3) |

| | | | | | | | | | |
|---|---|---|---|---|---|---|---|---|---|
| Ru1 | C4 | C8 | C9 | 69.8(2) | C31 | P2 | C24 | C23 | 114.03(16) |
| Ru1 | C4 | C5 | C6 | 53.87(15) | C31 | P2 | C25 | C30 | 140.70(14) |
| Ru1 | C4 | C3 | C2 | -52.96(16) | C31 | P2 | C25 | C26 | -40.71(16) |
| Ru1 | C1 | C6 | C5 | -53.76(15) | C31 | C36 | C35 | C34 | 1.9(3) |
| Ru1 | C1 | C2 | C3 | 53.40(15) | C31 | C32 | C33 | C34 | 1.1(3) |
| Ru1 | C5 | C6 | C1 | 54.17(15) | C36 | C31 | C32 | C33 | 0.4(3) |
| Ru1 | C3 | C2 | C1 | -52.84(15) | C36 | C35 | C34 | C33 | -0.4(3) |
| P1 | C17 | C18 | C19 | 173.70(15) | C25 | P2 | C24 | C23 | 8.62(18) |
| P2 | C24 | C23 | P1 | 178.83(10) | C25 | P2 | C31 | C36 | -96.52(15) |
| P2 | C31 | C36 | C35 | 178.93(16) | C25 | P2 | C31 | C32 | 84.31(17) |
| P2 | C31 | C32 | C33 | 179.52(15) | C25 | C30 | C29 | C28 | -1.1(3) |
| P2 | C25 | C30 | C29 | 179.53(13) | C6 | C1 | C2 | Ru1 | -54.75(14) |
| P2 | C25 | C26 | C27 | -178.59(14) | C6 | C1 | C2 | C3 | -1.3(3) |
| C24 | P2 | C31 | C36 | 158.93(15) | C32 | C31 | C36 | C35 | -1.9(3) |
| C24 | P2 | C31 | C32 | -20.24(18) | C32 | C33 | C34 | C35 | -1.1(3) |
| C24 | P2 | C25 | C30 | -114.95(14) | C15 | C16 | C11 | P1 | 173.98(14) |
| C24 | P2 | C25 | C26 | 63.65(16) | C15 | C16 | C11 | C12 | 0.3(3) |
| C22 | C17 | C18 | C19 | -0.6(3) | C15 | C14 | C13 | C12 | -0.2(3) |
| C22 | C21 | C20 | C19 | -0.8(3) | C3 | C4 | C8 | C10 | -76.1(2) |
| C17 | P1 | C23 | C24 | -116.38(17) | C3 | C4 | C8 | C9 | 159.45(17) |
| C17 | P1 | C11 | C12 | -153.02(14) | C3 | C4 | C5 | Ru1 | -55.21(14) |
| C17 | P1 | C11 | C16 | 33.21(17) | C3 | C4 | C5 | C6 | -1.3(3) |
| C17 | C22 | C21 | C20 | 0.5(3) | C11 | P1 | C17 | C22 | -137.10(15) |
| C4 | C5 | C6 | Ru1 | -54.52(15) | C11 | P1 | C17 | C18 | 48.69(16) |
| C4 | C5 | C6 | C1 | -0.3(3) | C11 | P1 | C23 | C24 | -7.54(19) |
| C4 | C3 | C2 | Ru1 | 52.47(15) | C11 | C12 | C13 | C14 | 1.0(3) |
| C4 | C3 | C2 | C1 | -0.4(3) | C11 | C16 | C15 | C14 | 0.5(3) |
| C8 | C4 | C5 | Ru1 | 127.62(17) | C30 | C25 | C26 | C27 | 0.0(3) |
| C8 | C4 | C5 | C6 | -178.51(17) | C18 | C19 | C20 | C21 | 0.4(3) |
| C8 | C4 | C3 | Ru1 | -127.99(16) | C28 | C27 | C26 | C25 | -0.6(3) |
| C8 | C4 | C3 | C2 | 179.05(17) | C27 | C28 | C29 | C30 | 0.4(3) |
| C7 | C1 | C6 | Ru1 | -124.68(17) | C2 | C1 | C6 | Ru1 | 55.46(14) |

| | | | | | | | | | |
|---|---|---|---|---|---|---|---|---|---|
| C7 | C1 | C6 | C5 | -178.44(16) | C2 | C1 | C6 | C5 | 1.7(3) |
| C7 | C1 | C2 | Ru1 | 125.39(16) | C29 | C28 | C27 | C26 | 0.4(3) |
| C7 | C1 | C2 | C3 | 178.79(17) | C13 | C12 | C11 | P1 | -174.90(15) |
| C23 | P1 | C17 | C22 | -29.81(16) | C13 | C12 | C11 | C16 | -1.0(3) |
| C23 | P1 | C17 | C18 | 155.98(15) | C13 | C14 | C15 | C16 | -0.6(3) |
| C23 | P1 | C11 | C12 | 100.08(15) | C26 | C25 | C30 | C29 | 0.9(3) |
| C23 | P1 | C11 | C16 | -73.69(16) | C20 | C19 | C18 | C17 | 0.3(3) |

**Table S37. Hydrogen Atom Coordinates (Å×10$^4$) and Isotropic Displacement Parameters (Å$^2$×10$^3$) for complex 7.**

| Atom | *x* | *y* | *z* | U(eq) |
|---|---|---|---|---|
| H24 | 3057.25 | 2433.59 | 2846.71 | 21 |
| H22 | 7411.95 | 2316.88 | 3319.44 | 24 |
| H8 | 9202.97 | 4960.2 | 4392.86 | 28 |
| H12 | 2987.06 | 4685.17 | 2714.89 | 25 |
| H7A | 2887.1 | 6067.54 | 3040.45 | 40 |
| H7B | 3229.67 | 6912.64 | 3471.09 | 40 |
| H7C | 2858.9 | 6033.45 | 3802.43 | 40 |
| H23 | 5455.91 | 2408.02 | 3713.9 | 21 |
| H5 | 7353.37 | 5416.73 | 2827.58 | 23 |
| H21 | 9464.06 | 1970.82 | 2961.78 | 29 |
| H16 | 4721.77 | 2853.89 | 1723.72 | 25 |
| H36 | 1067.49 | 318.53 | 3985.51 | 28 |
| H14 | 1147.14 | 3944.76 | 938.3 | 30 |
| H6 | 5032.33 | 5836.7 | 2627.39 | 23 |
| H32 | 2107.67 | 2842.77 | 3980.98 | 28 |
| H19 | 8810.42 | 3846.06 | 1613.9 | 30 |
| H15 | 2904.06 | 2958.17 | 875.95 | 30 |
| H35 | -796.88 | 664.61 | 4494.86 | 34 |
| H3 | 7358.44 | 5790.29 | 4763.14 | 24 |
| H33 | 181.94 | 3184.46 | 4449.27 | 31 |
| H30 | 5432.36 | 44.04 | 3921.53 | 23 |
| H18 | 6741.53 | 4191.69 | 1956.46 | 25 |

| | | | | |
|---|---|---|---|---|
| H28 | 7494.03 | 903.31 | 5616.14 | 30 |
| H27 | 5991.66 | 2058.14 | 5601.84 | 30 |
| H2 | 5069.84 | 6189.91 | 4569.96 | 24 |
| H29 | 7215.66 | -109.87 | 4784.11 | 27 |
| H13 | 1194.1 | 4809.38 | 1854.97 | 31 |
| H26 | 4178.01 | 2198.88 | 4757.93 | 25 |
| H10A | 9948.79 | 6440.13 | 3713.68 | 54 |
| H10B | 10861.84 | 5983.43 | 4313.86 | 54 |
| H10C | 9511.68 | 6492.52 | 4417.85 | 54 |
| H34 | -1252.27 | 2099.74 | 4720.84 | 33 |
| H20 | 10176.7 | 2745.65 | 2117.85 | 31 |
| H9A | 9103.56 | 4230.32 | 3398.87 | 46 |
| H9B | 10597.22 | 4606.36 | 3629.87 | 46 |
| H9C | 9590.1 | 5084.25 | 3072.65 | 46 |

## 13. References


1. C. Daguenet, P. J. Dyson, *Catal. Commun.* **2003**, *4*, 153-157.
2. I. Moldes, E. de la Encarnación, J. Ros, Á. Alvarez-Larena, J. F. Piniella, *J. Organomet. Chem.* **1998**, *566*, 165-174.
